# Sequential closed-loop Bayesian optimization as a guide for organic molecular metallophotocatalyst formulation discovery

**DOI:** 10.1038/s41557-024-01546-5

**Published:** 2024-06-11

**Authors:** Xiaobo Li, Yu Che, Linjiang Chen, Tao Liu, Kewei Wang, Lunjie Liu, Haofan Yang, Edward O. Pyzer-Knapp, Andrew I. Cooper

**Affiliations:** 1 Key Laboratory of the Ministry of Education for Advanced Catalysis Materials, Zhejiang Key Laboratory for Reactive Chemistry on Solid Surfaces, Zhejiang Normal University, Jinhua, China; 2 Materials Innovation Factory and Department of Chemistry, University of Liverpool, Liverpool, UK; 3 Leverhulme Research Centre for Functional Materials Design, University of Liverpool, Liverpool, UK; 4 School of Chemistry and School of Computer Science, University of Birmingham, Birmingham, UK; 5 Department of Chemistry and Chemical Engineering, Shanxi Datong University, Datong, China; 6 IBM Research, Daresbury, UK

**Keywords:** Photocatalysis, Organocatalysis, Cheminformatics, Computational chemistry, Photocatalysis

## Abstract

Conjugated organic photoredox catalysts (OPCs) can promote a wide range of chemical transformations. It is challenging to predict the catalytic activities of OPCs from first principles, either by expert knowledge or by using a priori calculations, as catalyst activity depends on a complex range of interrelated properties. Organic photocatalysts and other catalyst systems have often been discovered by a mixture of design and trial and error. Here we report a two-step data-driven approach to the targeted synthesis of OPCs and the subsequent reaction optimization for metallophotocatalysis, demonstrated for decarboxylative *sp*^3^–*sp*^2^ cross-coupling of amino acids with aryl halides. Our approach uses a Bayesian optimization strategy coupled with encoding of key physical properties using molecular descriptors to identify promising OPCs from a virtual library of 560 candidate molecules. This led to OPC formulations that are competitive with iridium catalysts by exploring just 2.4% of the available catalyst formulation space (107 of 4,500 possible reaction conditions).

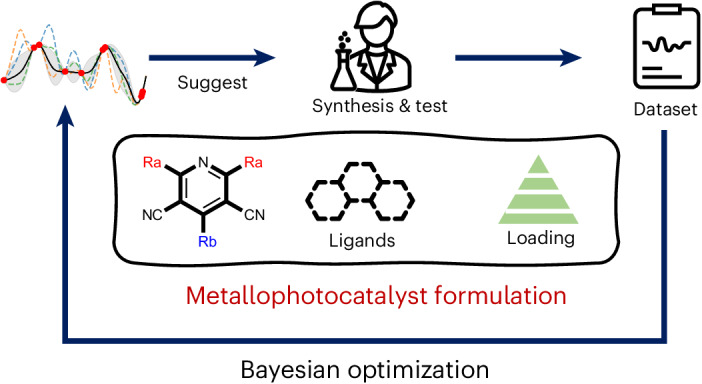

## Abstract (web summary)

Organic photoredox catalysts enable diverse chemical transformations, but predicting their activity is challenging due to complex properties. Now, a two-step data-driven approach is introduced for targeted organic photoredox catalysts synthesis and reaction optimization. Using Bayesian optimization, promising catalysts can be efficiently identified, yielding competitive results with iridium catalysts.

## Main

The activation of organic substrates via single-electron transfer (SET) using photoredox catalysts is a powerful tool in organic synthesis^1–7^. Metallophotocatalysis merges photoredox catalysis with transition-metal catalysis to allow organic reactions that are challenging with a single catalyst^8–12^. Metallophotocatalytic reactions can be complex, requiring several different components to function individually yet synergistically. The photoredox catalyst (PC) must exhibit suitable redox potentials in both the excited and ground states to allow for electron transfer to the substrates/transition-metal catalysts. This is essential for initiating the photocatalytic cycle and for regenerating the PC. Other crucial optoelectronic properties of PCs include light absorption, exciton lifetime and reorganization energy. So far, PCs have mostly been discovered through a mix of design, trial and error, and serendipity^13^. In some cases, high-throughput synthesis and testing have been used, particularly when the PCs can be generated in situ and do not require an elaborate purification procedure, as demonstrated for the discovery of transition-metal complexes as PCs^14^. Alternatively, photophysical properties can be used as design criteria to narrow the range of candidate PCs^15–19^. In such cases, it is often necessary to limit the complexity of the selection rules. However, photoredox catalysis is by its nature a multivariate problem, involving the intersection of many molecular and mesoscale factors. Also, the available a priori selection rules are only as good as our existing knowledge, which is far from complete.

Besides selecting the best photoredox catalysts, the optimization of reaction conditions—that is, the pairing of photoredox catalysts and transition-metal catalysts, reaction concentrations, and so on—can yield substantial improvements in metallophotocatalysis activity. However, experimental complexity scales exponentially with the number of variables, and the search for optimal experimental conditions requires the simultaneous variation of multiple components, often with little prior knowledge to draw upon. Factorial design of experiments (DOE) is a useful approach for such experimental search spaces, but this becomes ineffective as the dimensionality of the space increases, partly due to the use by DOE of a posteriori knowledge from previous experiments. DOE works less well for new multivariate systems where few constraints are known before experiment.

In this Article we report a data-driven approach to the targeted synthesis of organic photoredox catalysts (OPCs) and the subsequent reaction optimization for metallophotocatalysis, as demonstrated here for decarboxylative *sp*^3^–*sp*^2^ cross-coupling of amino acids with aryl halides. We adopted the established synergistic combination of photoredox catalysis and nickel catalysis but aimed to use an OPC in place of the more commonly used iridium photocatalysts^8^^,^^9^. OPCs offer potential advantages with respect to transition-metal-based photocatalysts, including lower cost, lower toxicity and high chemical diversity^2,20–22^. Although iridium-based photocatalysts are known for their versatility and high performance across a wide array of catalytic applications, recent advances have also highlighted the effectiveness of organic photocatalysts, notably CzIPN derivatives, in specific metallophotoredox reactions^20–22^. Nonetheless, optimizing these multicomponent systems, regardless of whether metal-based or organic photocatalysts are used, presents several challenges, including the labour-intensive nature of the process for multicomponent reactions.

**Fig. 1:**
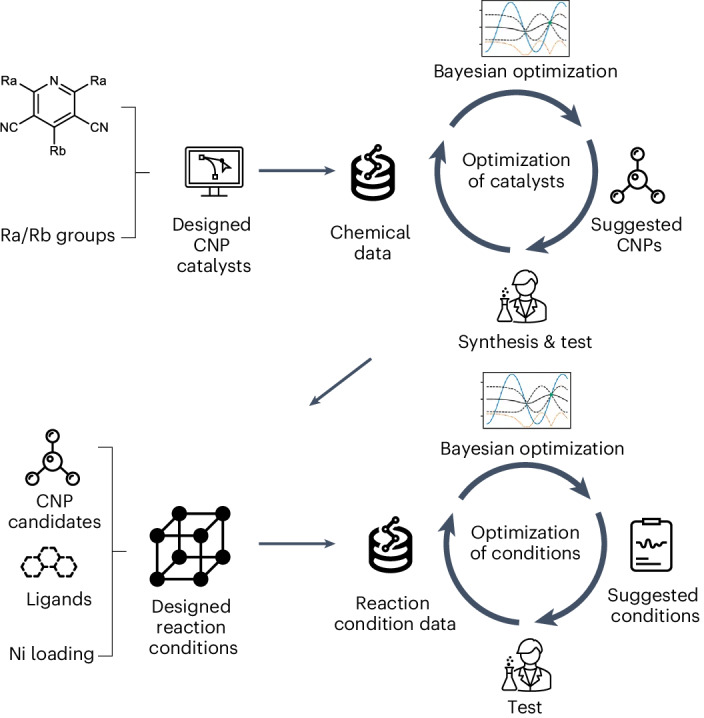
Workflow for sequential closed-loop BO for the discovery of OPCs in metallophotocatalysis formulations. The approach involves utilizing BO to guide catalyst selection, synthesis and test from a virtual library of organic CNP photocatalysts. Subsequently, an expanded optimization space, encompassing factors including nickel catalyst ligand and nickel loading, is explored using BO to further enhance the reaction efficiency.

**Fig. 2:**
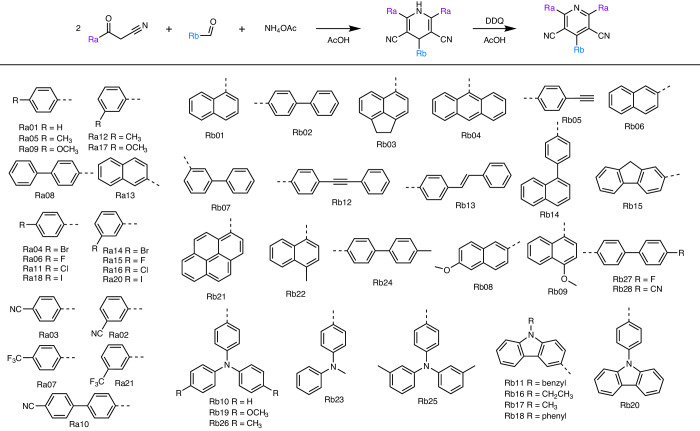
A virtual library of 560 candidate CNPs as potential photoredox catalysts. The reaction scheme for the Hantszch pyridine synthesis is shown, as well as the various chemical moieties that may be attached to the CNP core at the Ra or Rb positions. Different combinations of Ra and Rb moieties lead to 560 potential CNP molecules in this chemical space.

Our data-driven approach comprises two sequential closed-loop optimization workflows (Fig. 1), both integrating predictive machine learning (ML) with experiments under algorithmic control. The algorithm uses Bayesian optimization (BO) to explore the search space and to inform subsequent experiments^23–27^. First, we designed a virtual pool of 560 yet potentially synthesizable organic molecules using a common molecular scaffold based on the reliable and diversifiable Hantszch pyridine synthesis (Fig. 2). A batched BO was used to build a model that could be updated and queried to guide the experimental search for the most valuable catalysts. This iterative search strategy led us to synthesize 55 molecules out of the total library of 560 candidates, achieving reaction yields of 67% for the target reaction. In a second step, 18 of these synthesized molecules were selected for optimization of the reaction conditions, along with variation of the concentration of the nickel catalyst and its coordinating ligands. After evaluating 107 of the 4,500 possible sets of reaction conditions, guided by a second BO model, the highest reaction yield reached 88%.

## Results and discussion

### A library of candidate OPCs

The Hantzsch pyridine synthesis is a multicomponent organic reaction that produces pyridine compounds from an aldehyde, 2 equiv. of a β-ketoester, and a nitrogen donor, such as ammonia (Fig. 2)^28^. This reaction is metal-free and features high atom efficiency, using common reactants and facile reaction conditions. Here we combine 20 β-keto nitrile derivatives and 28 aromatic aldehydes (hereafter, these functional groups are denoted Ra and Rb, respectively) to construct a virtual library of 560 molecules that all share a common cyanopyridine (CNP) core, functionalized by different chemical moieties. All of these 560 CNP molecules are in principle synthesizable (Fig. 2). The 20 Ra groups comprise differently functionalized moieties: seven of them are electron-donating (ED), five are electron-withdrawing (EW), and eight are halogen-containing (X). The 28 Rb groups comprise 18 polyaromatic hydrocarbons (PAHs), five phenylamines (PAs) and five carbazole derivatives (CZs). These chemically and structurally diverse Ra and Rb moieties generate 560 different binary Ra/Rb combinations when combined into CNP molecules, offering the potential to tune the optoelectronic properties and redox potentials of the CNPs over a broad range. For instance, for D–A-type molecules, the ionization potential is mostly influenced by the donor part, and the electron affinity is controlled by the acceptor part. To our knowledge, the CNPs designed and evaluated in this study have not been reported previously in the literature for photocatalysis applications, apart from CNP-66. CNP-66, featuring the Ra03 and Rb11 groups, was reported recently by some of us for its photocatalytic hydrogen and hydrogen peroxide production, but it is not selected here by the BO algorithm for this reaction^29^. Synthetic considerations, particularly the availability of aldehydes and β-keto nitrile derivative precursors, also informed the initial selection of Ra and Rb groups. We ensured a broad coverage of molecular moieties in the ‘virtual’ chemical space, including electron-accepting/donating groups, halogens, polyaromatic hydrocarbons, phenylamines and carbazole derivatives. In other words, we aimed to prevent class imbalance biases in the design of the CNPs, while being mindful of the availability of starting materials.

**Fig. 3:**
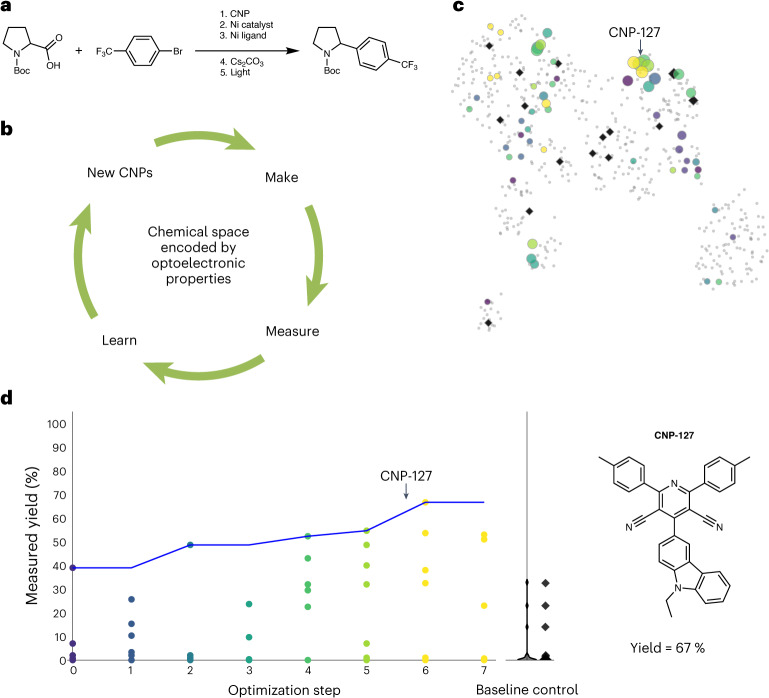
Targeted synthesis of CNPs for OPC discovery. **a**, The target reaction was a photoredox/Ni dual-catalytic C(*sp*^3^)–C(*sp*^2^) cross-coupling reaction. **b**, Catalyst discovery was driven by a closed-loop workflow using BO to link the experimental synthesis and measurement with predictive ML. **c**, Two-dimensional uniform manifold approximation and projection (UMAP) embedding of the chemical space (encoded by the calculated optoelectronic properties) of the 560 CNP molecules, where each point represents a molecule and the points are spatially arranged such that the closer the two points are on the plot, the more similar the two molecules are in the original high-dimensional chemical space. The CNP molecules synthesized here are colour-coded by experimental batch, using the colour scheme used in **d**. Molecules that were not synthesized are shown in grey. Symbol size denotes the experimentally measured reaction yield for the target reaction shown in **a**. **d**, Measured yield (average of three repeats) for the target cross-coupling reaction plotted against experiment batch (eight sequential batches). The highest yield attained after eight batches (optimization steps 0–7) was 67%. Black points refer to a baseline control experiment conducted for a set of 15 molecules chosen in a way that maximized the structural diversity of the set. The structure of the most active of the 55 molecules evaluated, CNP-127, is also shown. Source data

The cyanopyridine core of the CNPs is analogous to that of cyanoarenes, many of which are known to be active photocatalysts^2,30^. We expected that this diverse library of CNP molecules might contain promising OPCs for both reductive and oxidative photoredox reactions, as photoredox/nickel dual-catalysed cross-coupling reactions^21^. However, there were no clear, unambiguous physical principles to follow when selecting molecules from this virtual library for the target reaction shown in Fig. 3a. Synthesizing and testing all 560 CNP molecules was unrealistic. We therefore developed an active learning approach for the selection of CNPs for experiments, which made use of a closed-loop BO workflow with real-time feedback between experiment and prediction (Fig. 3b).

### Encoding the chemical space of CNP photocatalysts

The decarboxylative C(*sp*^3^)–C(*sp*^2^) cross-coupling reaction considered here (Fig. 3a) involves two interwoven catalytic cycles. One is photoredox catalysis and the other nickel catalysis. The commonly proposed mechanism for this dual catalysis has been discussed in detail previously^9,31,32^. In lieu of a simple predictive rule for selecting CNPs for experiments, we formulated the selection as an exploration of the available chemical space to maximize an objective metric, defined here as the reaction yield for the cross-coupling reaction. We encoded the 560 CNPs as a chemical space using 16 molecular descriptors that captured a range of thermodynamic, optoelectronic and excited-state properties (Methods section Molecular descriptors for CNP photocatalysts and Supplementary Section Computational details).

### Selecting synthetic targets using BO

For target selection from the virtual library of 560 CNPs (Fig. 2), we used a batched, constrained, discrete Bayesian optimization to explore the encoded chemical space of the CNPs (Fig. 3b), driving forward sequential experiments to improve the reaction yield. Starting from the complete, unexplored chemical space, we first selected a small set of six CNPs that were scattered across the space using the Kennard–Stone (KS) algorithm. These six CNPs were then synthesized and tested for the target reaction, forming step 0 in the optimization process (Fig. 3d; Supplementary Fig. 2 presents their molecular structures). All CNPs were tested under identical reaction conditions: 4 mol% CNP photocatalyst, 10 mol% NiCl_2_·glyme (glycol ether), 15 mol% dtbbpy (4,4′-di-*tert*-butyl-2,2′-bipyridine), 1.5 equiv. Cs_2_CO_3_ base, dimethylformamide (DMF) and blue light-emitting diode (LED) irradiation. All catalysis measurements were repeated three times, and the resulting average reaction yield is reported. The highest reaction yield achieved in step 0 was 39% for CNP-129, which combines Ra05 and Rb18. The yields achieved in step 0 gave us confidence that some but not all the CNPs in the virtual library had the potential to facilitate a synergistic combination of photoredox catalysis and nickel catalysis for the target C(*sp*^3^)–C(*sp*^2^) cross-coupling reaction.

Our BO started by building a Gaussian process (GP)-based surrogate model using the six data points in step 0. Subsequent sampling of 12 points per optimization step was carried out using sets of 12 upper confidence bound (UCB) functions: *α*_UCB_(*x*) = *μ*(*x*) + *βσ*(*x*), a weighted sum of the posterior mean *μ*(*x*) and uncertainty *σ*(*x*), controlled by hyperparameter *β*. For each step, the set of 12 *β* values was generated on a random exponential distribution, with small *β* values favouring predicted high performances *μ*(*x*), or exploitation, and large *β* values favouring high uncertainties *σ*(*x*), or exploration. From these 12 BO-proposed CNPs, we selected a subset of around six to eight CNPs per suggested batch for experiments, ensuring that the selected CNPs exploited a trade-off between exploitation and exploration, determined by their different *β* values. This protocol of joint decision-making for candidate PC selection combines both BO and insight from the chemist, including more prosaic factors such as the availability of starting materials for the CNPs. Seven such batches (Fig. 3) resulted in a total of 49 additional CNPs that were synthesized and tested (Supplementary Fig. 2 presents their molecular structures). The number of CNPs tested by experiment was 6, 6, 4, 8, 11, 6 and 8 in steps 1 to 7, respectively. The highest reaction yield attained increased from 39% at step 0 to 67% by step 7, which was achieved in step 6 using CNP-127 (Ra05 + Rb16). We decided to stop after these seven optimization steps because a yield of 67% was considered acceptable in the absence of reaction condition optimization.

**Extended Data Fig. 1:**
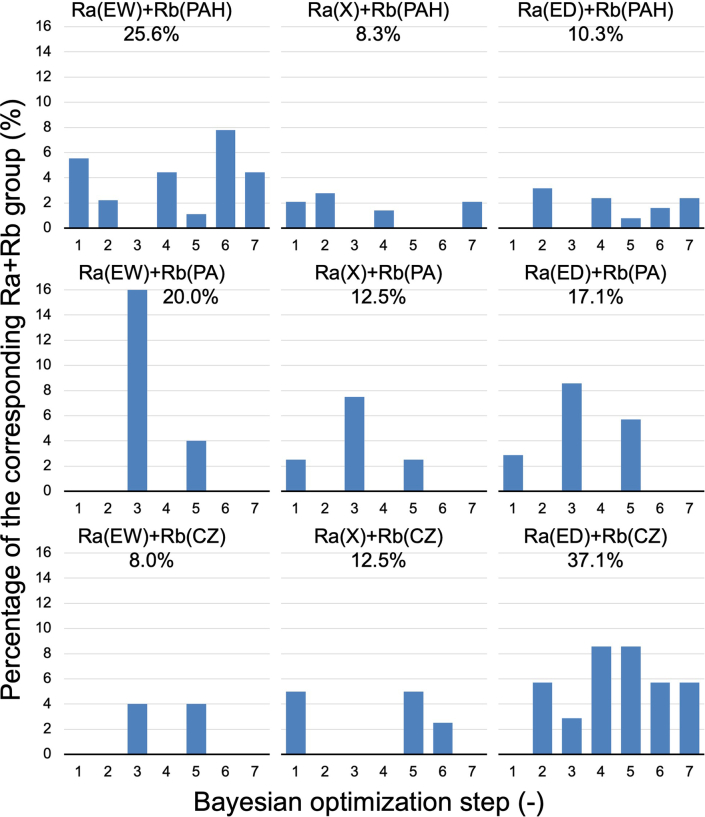
Selection and deselection of the binary combinations of Ra and Rb moieties for CNPs by BO over the seven optimization steps. EW = electron-withdrawing, X = halogen, ED = electron-donating, PAH = polyaromatic hydrocarbon, PA = phenylamine, and CZ = carbazole. The 560 CNPs cover 90 Ra(EW) + Rb(PAH) pairs, 144 Ra(X) + Rb(PAH) pairs, 126 Ra(ED) + Rb(PAH) pairs, 25 Ra(EW) + Rb(PA) pairs, 40 Ra(X) + Rb(PA) pairs, 35 Ra(ED) + Rb(PA) pairs, 25 Ra(EW) + Rb(CZ) pairs, 40 Ra(X) + Rb(CZ) pairs, and 35 Ra(ED) + Rb(CZ) pairs. At each BO step, 12 CNPs were suggested by the algorithm, and their number of suggestions per Ra+Rb pairing sub-group is shown in these histograms as a percentage. Source data

We can subdivide all the BO-recommended CNPs (12 molecules per step over seven steps) into a 3 × 3 grid of nine binary Ra + Rb groups (Extended Data Fig. 1) arising from the combination of three types of Ra group (EW, X, ED) with three types of Rb group (PAH, PA, CZ). This reveals how the BO algorithm selects and deselects certain Ra–Rb pairs to improve the reaction yield. From step 2 onward, the algorithm consistently suggested the pairing of ED Ra groups with CZ Rb groups in all batches (Extended Data Fig. 1, bottom right plot). After step 5, the BO algorithm deselected PA Rb groups in all batches. The selection of PAH Rb groups was scattered over the seven steps. Supplementary Fig. 3a shows the overall structure–activity relationship for the experimentally measured CNP samples. The CNPs consisting of CZ Rb moieties paired with donating Ra moieties provide optimal yields. CNPs with PA Rb groups exhibit low photoreaction yields, regardless of the Ra group. The yields of CNPs with PAH Rb groups are generally low, except for those with Ra09–Rb21, Ra12–Rb21 and Ra14–Rb09 pairs, which show modest yields. These experimental results validate the suggestions of Ra–Rb pairs made by the BO algorithm in the search for improved photocatalysis yields.

One might ask whether the ‘sweet spots’ discovered by the BO search within this virtual library of 560 CNPs constitute a global optimum, or at least close to one. Without synthesizing the entire library, which was impractical, we cannot be sure of this. However, to probe this further, a diverse set of 20 CNPs was picked from the two-dimensional (2D) structural space of the 560 CNPs encoded by Morgan fingerprints, using the KS algorithm, as used to pick the CNPs for step 0. Of the selected 20 CNP samples, three were unsynthesizable within the time available, and two (CNP-459 and CNP-244) were already picked up by the BO algorithm. As such, 15 additional CNPs were synthesized and tested for the photoredox reaction (their molecular structures are shown in Supplementary Fig. 2). The results obtained were in line with the structure–activity relationship summarized for the CNP samples arising from the BO search (Supplementary Fig. 3a). The CNPs with Rb moieties (Rb04, Rb06, Rb08, Rb13 and Rb15), which were not explored by the BO search, showed no or little activity (yield < 3%). The highest yield attained by the molecules in this structurally selected baseline control set was 32%.

**Extended Data Fig. 2:**
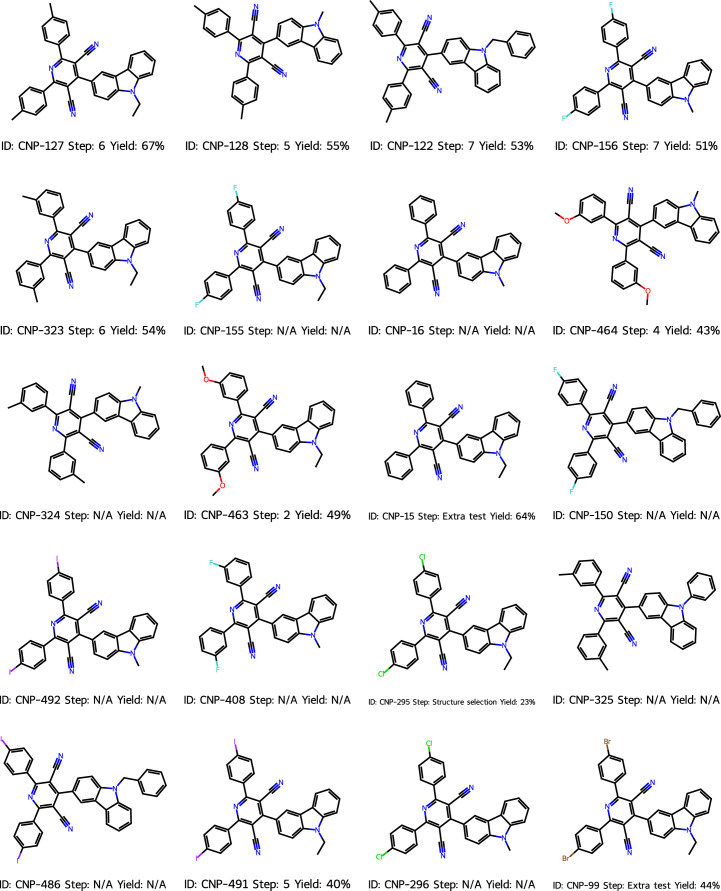
CNP-127 and its 19 closest neighbours in the chemical space depicted in Fig. 3c. Of the 19 CNPs, ten were measured experimentally with their corresponding reaction yields given in the figure.

Extended Data Fig. 2 shows the 19 closest neighbours to CNP-127 in the chemical space depicted in Fig. 3c, arranged in order of increasing distance from CNP-127. Of these 19 CNPs, eight were experimentally measured, with seven recommended by BO and one selected based on structural diversity. This approach is exploitative in that it focuses on the local chemical neighbourhood of CNP-127, so high performance was anticipated based on the model’s predictions. Two additional CNPs, CNP-15 and CNP-99, were synthesized and evaluated (Extended Data Fig. 2). This exploration of the ten CNPs in the vicinity of CNP-127 reaffirmed it to be the most active catalyst among those tested.

We also trained ML models on the 70 CNPs from our first BO workflow and used them to predict yields for 100 new CNPs (Supplementary Figs. 4–6), finding none likely to outperform CNP-127. We synthesized and tested three new CNPs (CNP-561, CNP-565 and CNP-577), all featuring the Rb16 carbazole group but varying in their Ra groups, achieving experimental yields close to their predicted values without optimizing the reaction conditions. After experimentally evaluating 75 CNPs, including those recommended by BO and those chosen for their diversity or for being in the vicinity of CNP-127, as well as these new ones, we are inclined to believe that CNP-127 is either the most active or among the top photocatalysts in our study. Furthermore, the structure–activity relationship for CNPs with carbazole Rb groups (CNP-C) was also investigated as they were prioritized by the BO algorithm and gave high photocatalytic yields compared to other Rb groups. A weak negative correlation between CNP-C materials with low reduction potentials and high photocatalysis yields was observed (Supplementary Fig. 3b,c). We note here that the correlation of reduction potential with yield does not apply to the whole set of CNP molecules (Supplementary Fig. 7). Utilizing single reduction potentials as the descriptor to suggest CNP candidates from 100 new CNPs was attempted. However, the suggested CNP-624 candidate exhibited no activity. Full details are given in Supplementary Section Searching for new active CNPs through machine learning prediction.

### Understanding the key molecular features for photocatalysis

**Fig. 4:**
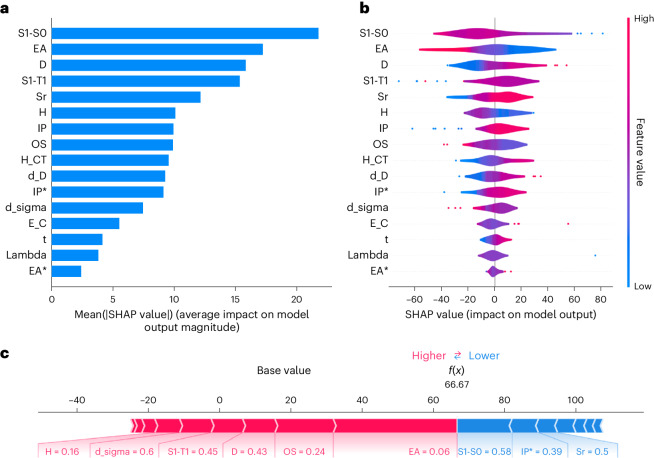
SHAP analysis identifies important molecular features for photocatalytic activity. ML model explanations for the prediction of experimentally measured reaction yields for the 55 CNPs evaluated during BO-driven photocatalyst discovery. The best ML model (using GPs) for predicting the reaction yields was used to obtain SHAP explainers and to calculate SHAP values. **a**, Bar plot of feature importance, showing the average absolute SHAP values of each input feature across all instances in the dataset (that is, all 55 CNPs). **b**, Beeswarm plot of feature importance, showing the distribution of SHAP values for each input feature across all instances in the dataset. **c**, Force plot explaining the ML model’s prediction for the best-forming catalyst, CNP-127, showing how each input feature contributes to the prediction. Each feature’s contribution is represented by an arrow, with the length of the arrow proportional to the magnitude of the SHAP value. Red arrows pointing to the right indicate positive contributions, and blue arrows pointing to the left indicate negative contributions.

To explain the underlying GP models that drove BO for the targeted synthesis of CNPs, we used the SHAP (Shapley additive explanations) explanation framework^33^. For the 55 CNPs that were synthesized and tested in the first BO workflow (Fig. 3), light absorption (Δ*E*_S1 → S0_; the optical gap), was identified as the most influential input feature. Small optical gaps (good light absorption) correlate broadly with positive contributions to the model’s predictions (Fig. 4a,b). The second most important feature was electron affinity (EA), which had strong, positive contributions when its values were low and strong, and negative contributions when its values were high. EA estimates the reducing ability of CNP^−^ in regenerating the intermediate nickel species, which subsequently undergoes an oxidative addition into the aryl-halide substrate. This is the key SET step connecting the photoredox catalytic cycle and the nickel catalytic cycle.

Excited-state charge separation was also identified to be influential, with the *D* index (a measure of the electron–hole distance) and the *S*_r_ index (a measure of the extent of electron–hole overlap) ranked as the third- and fifth-most important input features, respectively. Both high *D*-index values and low *S*_r_-index values result from better excited-state charge separation, which were both found to have strong positive contributions to the model’s predictions. Local explanations for the most active catalyst, CNP-127, were also constructed by SHAP analysis (Fig. 4c). The strong reducing ability (EA) of its CNP^−^ species, a large oscillator strength (OS) of *S*_1_, and a good excited-state charge separation (*D* index) are the key beneficial factors in its strong photocatalytic performance.

### Identifying the best reaction conditions using BO

**Fig. 5:**
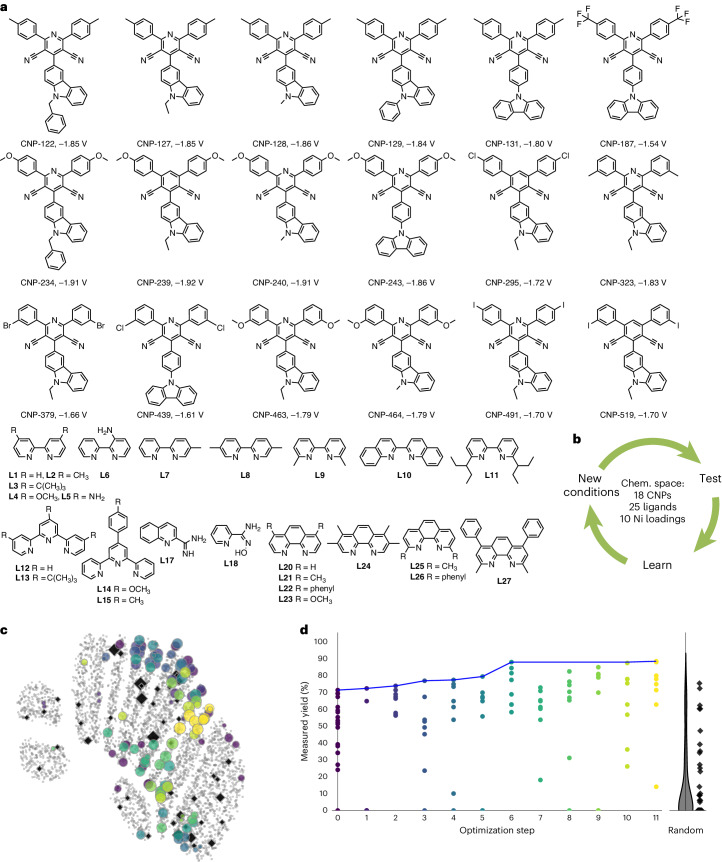
Reaction condition optimization. **a**, A total of 18 candidate carbazole CNPs and 25 candidate pyridyl ligands were considered in these experiments. **b**, Combinations of CNP photocatalyst, pyridyl ligand for Ni coordination and Ni concentration were screened in a closed-loop workflow using BO. **c**, 2D UMAP embedding of the chemical space of the 4,500 sets of reaction conditions. The tested set of conditions are colour-coded by experimental batch, adopting the same colouring scheme as in **d**, with all the untested conditions coloured in grey. Symbol size denotes the experimentally measured reaction yield. **d**, Measured yield for the target reaction plotted against experiment batch, optimization steps or baseline (that is, random selection). Number of samples: 19 samples at step 0; eight samples each at steps 1–11; 44 samples were included in a random selection as a baseline (black points). Source data

Next, we set out to use a similar Bayesian strategy to optimize the overall conditions of the reaction. The target decarboxylative C(*sp*^3^)–C(*sp*^2^) cross-coupling reaction requires a photoredox catalyst—in our case CNPs—and an organometallic nickel catalyst. As discussed above, these two catalysts must work synergistically in completing two interwoven catalytic cycles. The diffusional electron transfer between CNP^−^ and Ni intermediates not only depends on the thermodynamic driving force, but also on the concentration of Ni catalysts and Ni ligands. The maximum observed yield of 67% was achieved with fixed Ni loading and Ni ligands. Varying the concentration of the Ni catalyst (10 mol% or 1 mol%) markedly affected the reaction yields for the target cross-coupling reaction (Supplementary Table 3). To further optimize the reaction yield, three key variables were studied: (1) the choice of CNP photocatalyst, (2) the concentration of the Ni catalyst and (3) the choice of the Ni-coordinating pyridyl ligand. We used 18 carbazole-containing CNPs that were selected in the first BO workflow (Fig. 3), chosen to exhibit widely varying catalytic performance (0–71%; Supplementary Table 3). We chose this range of carbazole catalysts rather than simply the best material from the first BO selection (CNP-127), as it was initially unclear that this catalyst would also be optimal at all Ni concentrations and with all Ni ligands. A range of 25 pyridyl compounds were selected with different coordination environments and different molecular shape, size and degree of bulkiness (Fig. 5a). A total of 10 Ni concentration values were studied between 1 mol% and 10 mol%, with 1 mol% intervals. These three variables gave rise to a total of 4,500 (18 × 25 × 10) potential unique experiments.

We encoded the 4,500 sets of reaction conditions into a chemical space as follows. First, each combination of CNP, pyridyl ligand and Ni concentration was encoded by the concatenation of (1) the experimentally measured reduction potential *E*_1/2_^red^[CNP/CNP^−^], a measure of the reducing ability of the CNP^−^ species to regenerate intermediate nickel species; (2) the Morgan fingerprint of the CNP; (3) the Morgan fingerprint of the pyridyl ligand; (4) the concentration of the Ni(II) source. Second, the distance between two sets of reaction conditions was given as a combined distance from these four encoding elements, which is the summation of (1) the scalar difference between the reduction potentials; (2) the Tanimoto distance between the CNPs’ fingerprints; (3) the Tanimoto distance between the pyridyl ligand fingerprints, and (4) the scalar distance between the Ni concentrations. All four component distances were normalized before being added together to give the combined distance. Finally, the chemical space encoding the 4,500 sets of reaction conditions took the form of a 4,500 × 4,500 distance matrix, containing pairwise distances for all sets of reaction conditions. Figure 5c shows the resulting chemical space as a 2D uniform manifold approximation and projection (UMAP) embedding of the distance matrix.

The initial 19 sets of reaction conditions (step 0; Fig. 5d and Supplementary Table 3) were used to train GP-based surrogate models to suggest the first batch of experiments (step 1) in the optimization workflow. The same BO parallel sampling approach was used as for the synthetic candidate selection workflow. Eight samples were acquired at each BO step, covering a portfolio of UCB functions with varying degrees of balance between exploitation and exploration (Fig. 5d). From step 0 to step 6, the maximum reaction yield achieved at each step increased continuously from 71% to 88%. No further improvement in the maximum yield was attained in the subsequent five steps (40 reactions). The optimization was therefore terminated at step 11, having evaluated 88 sets of reaction conditions. The highest yield achieved during the 11 BO steps was 88%. This occurred when CNP-127 was used at Ni concentrations of 2, 4 or 5 mol%, in all cases with the 4,4′-dimethyl-2,2′-bipyridine ligand (L2). In step 0, CNP-239 was the highest-performing photocatalyst, reaching a yield of 71%, together with 4,4′-di-*tert*-butyl-2,2′-bipyridine (L3) and 1 mol% Ni concentration. As such, CNP-127 is effectively ‘rediscovered’ in this second BO search, but the reaction conditions are reoptimized to improve the photocatalytic yield from 71% to 88%.

Of the 88 samples requested by the BO algorithm and tested experimentally, 14 samples involved CNP-127, of which eight reactions also included L2. The eight samples involving both CNP-127 and L2 all gave high reaction yields (74–88%) for eight different Ni concentrations (2, 3, 4, 5, 6, 7, 8 or 10 mol%). For the CNP-127/L2 combination, lower Ni concentrations up to 5 mol% gave equivalent catalytic performances (87–88% yields), with decreasing performance at increasing Ni concentrations. Replacing L2 with a different pyridyl ligand also had a substantial impact on the catalytic performance of CNP-127. For example, 79% and 88% yields were measured for CNP-127 with L8 and L2, respectively (2 mol% Ni in both cases). Similarly, L4, L21 and L13 gave yields of 57%, 36% and zero, respectively, when combined with CNP-127 at low Ni concentrations (1, 2 and 4 mol%).

**Extended Data Fig. 3:**
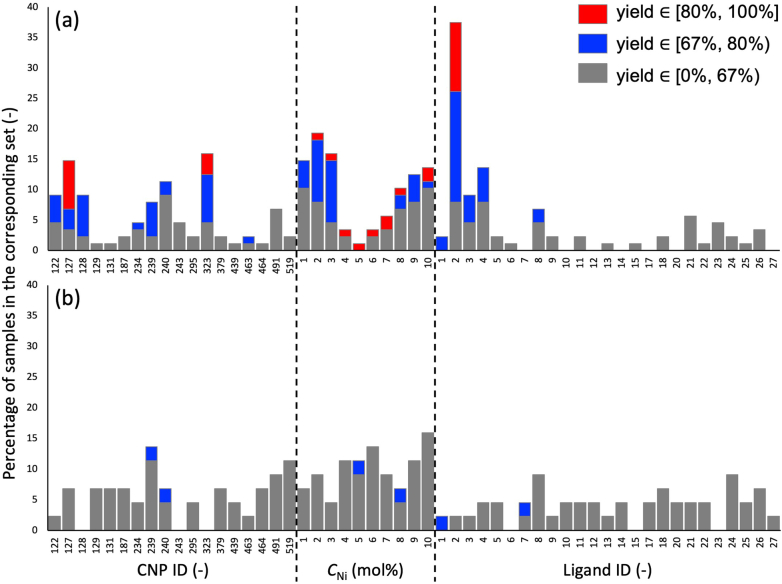
Histograms of measured samples (sets of reaction conditions) over three different variable ranges: candidate CNPs, Ni concentrations (CNi), and pyridyl ligands. (**a**) The 88 samples obtained during the BO search. (**b**) The 44 samples obtained by random selection. These histograms were calculated separately for each value range in either the BO set or the random set. The bars are coloured red, blue, or grey to indicate a yield greater than 80%, 67–80%, or lower than 67%, respectively. Source data

Overall, the 88 sets of conditions requested by the BO algorithm covered all 18 CNPs (that is, each was selected at least once), all 10 Ni concentrations and 18 out of the 25 available pyridyl ligands (Fig. 5a). Ten of the 88 sets of conditions gave a reaction yield greater than or equal to 80% (Extended Data Fig. 3, red bars), all of which involved CNP-127 or CNP-323 as the photocatalyst and L2 as the Ni-coordinating ligand. We note that CNP-323 is a structural isomer of CNP-127 (Fig. 5a).

For a baseline comparison, we randomly selected 44 sets of conditions for catalysis measurements (Fig. 5d). Only two sets of conditions attained a yield above 67% (Extended Data Fig. 3b, blue bars). By comparison with the BO-acquired samples (Extended Data Fig. 3), a more uniform sample distribution was generated by random sampling for the candidate CNPs, Ni concentrations and pyridyl ligands. The BO search markedly outperformed the random sampling, attaining a higher maximum reaction yield (88% versus 75%). Also, the BO method gave a much larger proportion of high-activity samples (Extended Data Fig. 3). For example, 39 of 88 reaction conditions (44%) gave yields of more than 67% for the BO search, whereas just 2 of 44 (4.5%) conditions gave a comparable yield in the random selection. This shows that BO explores the high-performing areas of the chemical space much more effectively than random sampling.

### Benchmarking the activity of CNP-127

**Fig. 6:**
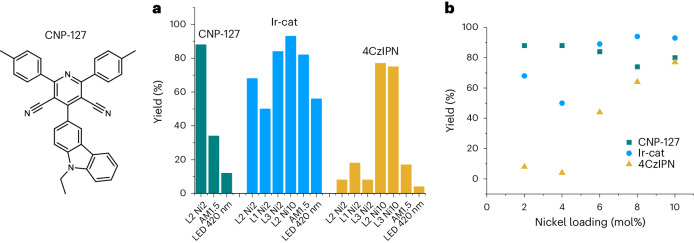
Benchmarking CNP-127 activity against established photoredox catalysts. **a**, C(*sp*^3^)–C(*sp*^2^) cross-coupling reaction performance comparison in the presence of CNP-127, Ir-cat or 4CzIPN with different ligands and irradiation sources. L1, 2,2′-bipyridine ligand; L2, 4,4′-dimethyl-2,2′-bipyridine ligand; L3, 4,4′-di-*tert*-butyl-2,2′-bipyridine. Condition: L2Ni2 for three photocatalysts using different irradiation sources (blue LED, 420-nm LED or solar simulator (350–1,000 nm)). **b**, Dependence of C(*sp*^3^)–C(*sp*^2^) cross-coupling reaction performance at different nickel loadings. Conditions: 4 mol% photocatalyst, *X* mol% NiCl_2_·glyme (glycol ether), *X* × 1.5 mol% L2, 1.5 equiv. Cs_2_CO_3_ base, DMF and blue-LED irradiation source. Source data

To benchmark the activity of the best-performing CNPs, two classical catalysts were considered: 4CzIPN^21^ and a transitional-metal catalyst, (Ir[dF(CF_3_)ppy]_2_(dtbbpy))PF_6_ (Ir-cat)^9^ (Fig. 6). We note that the activities obtained with 4CzIPN and Ir-cat were not as thoroughly optimized in terms of ligands and nickel loading as for the CNP case, above. Moreover, the concentration of catalysts may have additional impacts on their performance. Overall, CNP-127 exhibits comparable activity to Ir-cat, with higher yields at low nickel loadings and slightly lower yields at higher nickel loadings. CNP-127 also showed better activity than 4CzIPN under these conditions. Interestingly, the activity of 4CzIPN and Ir-cat decrease with a decrease in nickel catalyst concentration, whereas the activity of CNP-127 gradually increases with decreasing nickel catalyst concentration from 10 mol% to 2 mol%. We believe that the retention of high photoactivity at low nickel loadings for CNP-127 could be attributed to the high reducing power (*E*_1/2_^red^ cat/cat^−^) of the reduced CNP-127 (−1.85 V versus Fc^+^/Fc; cf. 4CzIPN, −1.68 V versus Fc^+^/Fc; Ir-cat: −1.72 V versus Fc^+^/Fc), which facilitates electron transfer. Reducing the nickel loadings in photoredox catalysis is appealing because it might improve sustainability while also mitigating the potential for metal-residue contamination. Further experimental investigations and discussions on the photocatalytic activity of CNPs, including its dependence on nickel concentration, are provided in Supplementary Section Additional discussions on benchmarking the photocatalytic activity of CNP-127 and Supplementary Figs. 8 and 9.

## Conclusions

An experimental BO strategy coupled with molecular descriptors has been used to identify promising OPCs from a virtual library of 560 candidate molecules while exploring a small fraction of the available chemical space (55 of 560 OPCs in the first BO search; 107 of 4,500 reaction conditions in the second reaction condition optimization). This search identified OPCs with reaction yields for a cross-coupling reaction of up to 88%, which is similar to that achieved by iridium catalysts. SHAP analysis was employed to generate both global and local explanations for the surrogate model used in the first BO campaign, thereby identifying the important molecular features of CNPs that contributed to their photocatalytic activity. BO is a promising approach for the discovery of metallophotocatalyst formulations, and by extension for other research challenges where there is a large search space and limited prior knowledge. The BO approach also has some limitations. For example, the Hanztsch synthesis is broadly generalizable, but it is not ubiquitous for all combinations of Ra and Rb functionalities. This was addressed by fusing BO algorithms with human decisions as to which molecules to pursue in each batch. It would be desirable in the future to fully automate such workflows using robots, but this would require additional technical developments, for example, to carry out automated trial syntheses for candidate OPCs and to make autonomous decisions about which OPCs to carry forward into catalysis testing.

## Methods

### Photoredox reaction experiments

#### CNP BO search

Aromatic halides (0.10 mmol, 1 equiv.), Boc–Pro-OH (0.15 mmol, 1.5 equiv.), Cs_2_CO_3_ (0.15 mmol, 1.5 equiv.), CNP (0.004 mmol, 0.04 equiv.), NiCl_2_ glyme (0.01 mmol, 0.1 equiv.), 4,4-di-*tert*-butyl-2,2-bipyridyl (0.015 mmol, 0.15 equiv.) and 4 ml of DMF were discharged into a glass vial (Fisherbrand, cat. no. 10504463) equipped with a screw cap (Supelco lot 134901) and magnetic stir bar. The reaction mixture was degassed by a bubbling nitrogen stream for 5 min, then irradiated under a blue LED (SynLED) for 3 h. The yield of product was calculated using biphenyl as the standard by gas chromatography–mass spectrometry (GC-MS).

### BO reaction optimization

Aromatic halides (0.10 mmol, 1 equiv.), Boc–Pro-OH (0.15 mmol, 1.5 equiv.), Cs_2_CO_3_ (0.15 mmol, 1.5 equiv.), the corresponding CNP (0.004 mmol, 0.04 equiv.), NiCl_2_ glyme, ligand and 4 ml of DMF were discharged into a glass vial (Fisherbrand, cat. no. 10504463) equipped with a screw cap (Supelco lot 134901) and magnetic stir bar. The reaction mixture was degassed by a bubbling nitrogen stream for 5 min, then irradiated under a blue LED (SynLED) for 21 h. The yield of product was calculated using biphenyl as the standard by GC-MS.

### Computational and ML details

All density functional theory (DFT) and time-dependent (TD) DFT calculations were performed with the CAM-B3LYP density functional, together with the 6–31G* basis set (LANL2DZ was used for bromine and iodine atoms), using Gaussian 16 software^34^. The effect of solvation by DMF was accounted for by using the polarizable continuum model/solvation model density (PCM/SMD) solvation model. Adiabatic IP, EA, EA* and IP* were calculated using fully optimized geometries for the respective charge states. All electron excitation analyses were performed using Multiwfn^35^. All calculations were conducted on an Intel(R) Xeon(R) Gold 6138 CPU @ 2.00 GHz. For each CNP molecule, the geometry optimization calculations (for charge states 0, +1 and −1) were completed in ~5 h using four CPU cores. The calculations for the singlet and triplet excited states via TD-DFT required ~2 h with four cores. Additionally, the optimization calculations for the singlet excited states were completed in around one day using eight cores. The time required for other DFT energy calculations and excited-state descriptor computations was relatively minimal. For the total of 660 CNP molecules examined in this study, the overall computational effort amounted to an estimated 145,200 core-hours.

The dimensionality reduction algorithm, UMAP^36^, was used to map high-dimensional data onto 2D representations, where each point represents a molecule (Fig. 3c) or a set of reaction conditions (Fig. 5c). Points on a 2D UMAP projection are spatially arranged such that the closer the two points are on the plot, the more similar the two points are in the original, high-dimensional space.

### Molecular descriptors for CNP photocatalysts

The photo-excited CNP (CNP*) oxidizes the α-amino-acid substrate via a SET event, generating an α-amino radical and the corresponding CNP^−^. Concurrently, the nickel catalytic cycle involves oxidative addition of the Ni(0) species into the aryl-halide substrate, producing a Ni(II)-aryl intermediate, which captures the α-amino radical and produces an alk-N(III)-aryl adduct. The desired C(*sp*^3^)–C(*sp*^2^) bond is subsequently forged via reductive elimination. A second SET event between the CNP^−^ species and the Ni(I) species expelled after the C(*sp*^3^)–C(*sp*^2^) bond formation completes both the photoredox cycle and the nickel catalytic cycle simultaneously, regenerating the CNP photocatalyst and the Ni(0) species.

Thermodynamically, the excited-state photocatalyst, CNP*, must be a strong oxidant for the α-amino-acid substrate, Boc–Pro–OH, which has a reduction potential, *E*^red^, of 1.19 V versus the standard hydrogen electrode (SHE)^37^. Using DFT and TD-DFT calculations, we determined the reduction potential for the CNP* → CNP^−^ half-reaction, *E*_1/2_^red^[CNP*/CNP^−^], for all 560 CNP molecules. All but four of these CNPs had a calculated value of *E*_1/2_^red^[CNP*/CNP^−^] that exceeded 1.19 V versus SHE, meaning that the oxidation of Boc–Pro–OH by CNP* should be thermodynamically favourable for most of the candidate CNPs. The resulting CNP^−^ from the oxidation process must then act as a strong reductant to regenerate the nickel intermediates (Ni(0) and/or Ni(I) species, for which *E*_1/2_^red^[Ni^I^/Ni^0^] = −1.17 V versus SHE and *E*_1/2_^red^[Ni^II^/Ni^I^] = −1.02 V versus SHE)^32,38^. All but one of the 560 CNPs were calculated to have *E*_1/2_^red^[CNP/CNP^−^] < −1.17 V versus SHE, suggesting that they should be able to drive the Ni reduction process. These computational results indicate that most of the candidate CNP molecules might in principle act as photocatalysts for the target reaction but, as will be seen, this also illustrates that it is not possible to use these redox potentials alone as selection criteria. This is because the catalytic activity is not defined by thermodynamic redox potential alone.

To be thermodynamically viable in the reaction, the EA (equivalent to *E*_1/2_^red^[CNP/CNP^−^]) and the exciton electron affinity (EA*, equivalent to *E*_1/2_^red^[CNP*/CNP^–^]) of the CNP molecule must straddle the Ni(I) reduction and Boc–Pro–OH oxidation potentials. Also, the optoelectronic and excited-state properties of the molecule may strongly influence its photocatalytic activity, so they were also used to encode the chemical space. The properties that we included were (1) light absorption (first singlet excited state, Δ*E*_S1 → S0_, together with the oscillator strength, *f*, of this transition); (2) excited-state charge distribution (change in dipole moment between *S*_1_ and *S*_0_, Δ*D*; degree of spatial extension of hole and electron distributions in the charge-transfer direction, *H*_CT_, *H* index, *t* index); (3) excited-state charge separation (difference in the extent of spatial distribution between electron and hole, Δ*σ*; electron–hole overlap, *S*_r_; distance between the centres of the electron and hole, *D* index; Coulomb attraction between the electron and hole, *E*_C_); (4) the energy gap between the first singlet state and the first triplet state (Δ*E*_S1 → T1_); and (5) the internal reorganization energy of CNP^–^ acting as a reductant for the Ni(I) compound. Definitions and calculation details for all 16 descriptors are given in Supplementary Section Computational details.

### Bayesian optimization

BO is a sequential hypothesis testing approach to the global optimization of ‘black-box’ functions, that is, functions that do not have a closed-form representation and do not provide function derivatives, thus only allowing for pointwise evaluation. Here, it equates to finding the highest reaction yield in the chemical space of the CNPs or reaction conditions.

Our surrogate models made use of GPs, together with the Matérn similarity kernel. A GP maintains a belief over the design space by simultaneously predicting the mean, *μ*(*x*_i_), and the uncertainty, *σ*(*x*_i_), at any point *x*_i_ in the input space, given existing observations. To hypothesize the most promising setting for the next experiment, based on the current, predicted mean and uncertainty, an acquisition function is required. Here, the UCB function was used, which is given by

1 $${\alpha }_{\rm{UCB}}{(x)}={\mu \left(x\right)+\beta \sigma (x)}$$

where *μ*(*x*) is the posterior mean, *σ*(*x*) is the uncertainty, and *β* is a hyperparameter. For each optimization step, the highest value of the acquisition function (equation (1)) was used as the next experimental suggestion.

The syntheses and photocatalytic measurements of CNPs were time-consuming, but were amenable to parallelization; that is, they could be made and tested in batches. To facilitate an efficient parallel search, a batched, discrete BO approach was adopted, where multiple BO instances were run in parallel, all using the same existing observations and contributing to the subsequent steps. Here, a set of 12 BO instances were run at each optimization step. This parallel sampling strategy allowed for intuitive biasing towards the exploitation or exploration of the search space by assigning different *β* values to the acquisition functions of different BO instances at each step. Small values of *β* prioritized areas where the mean was expected to be largest (that is, exploitation), and large values prioritized areas where the model was most uncertain (that is, exploration). A random exponential distribution function was used to generate *β* values within a batch.

For the selection of CNPs for synthesis, our BO scheme comprised two main steps. First, a surrogate model based on GPs was trained on all available observations, that is, the measured reaction yields for all synthesized CNPs at that point. Second, a new set of CNPs was proposed for subsequent experiments, using predictions by the surrogate model. This equates to the BO predicting the performances of candidate CNPs using available data and requesting new CNPs to be synthesized to verify its predictions. Our parallel sampling strategy is an intuitive and inexpensive approach to proposing multiple points (forming a batch) in the search space, using a portfolio of acquisition functions favouring exploitation or exploration of the search space. Our BO implementation follows that used previously in a robotic workflow used to find improved photocatalysts for hydrogen production^24^.

For optimization of the reaction conditions, we defined and used a customized radial basis function (RBF) kernel, given by

2 $${\mathscr{K}}\left({P}_{i},\,{P}_{j}\right)={\alpha \times {\rm{e}}}^{-\left({\theta }_{1}{D}_{\rm{EA}}^{2}\left({M}_{i},\,{M}_{j}\right)+{\theta }_{2}{D}_{\rm{fps}}^{2}\left({M}_{i},\,{M}_{j}\right){+\theta }_{3}{D}_{\rm{fps}}^{2}\left({L}_{i},\,{L}_{j}\right)+{\theta }_{4}{D}_{C}^{2}\left({C}_{i}^{\rm{Ni}},\,{C}_{j}^{\rm{Ni}}\right)\right)}$$

where *P*_*i*_ and *P*_*j*_ are two sets of reaction conditions, each involving a CNP molecule (*M*_*i*_ or *M*_*j*_), a Ni-coordinating ligand (*L*_*i*_ or *L*_*j*_) and a Ni concentration (${C}_{i}^{\rm{Ni}}$ or ${C}_{j}^{\rm{Ni}}$). For the CNP pair (that is, *M*_*i*_ and *M*_*j*_), we considered the Euclidean distance between the EA values of the two CNPs, *D*_EA_(*M*_*i*_, *M*_*j*_), and the Tanimoto distance between the two CNPs’ Morgan fingerprints (fps; radius = 2; 2,048 bits), *D*_fps_(*M*_*i*_, *M*_*j*_). For the ligand pair (that is, *L*_*i*_ and *L*_*j*_), we considered the Tanimoto distance between the two ligands’ Morgan fingerprints (radius = 2; 2,048 bits), *D*_fps_(*L*_*i*_, *L*_*j*_). For the Ni concentration pair (that is, ${C}_{i}^{\rm{Ni}}$ and ${C}_{j}^{\rm{Ni}}$), we consider the Euclidean distance between the values of Ni concentration of the two reaction condition sets, ${D}_{C}\left({C}_{i}^{\rm{Ni}},\,{C}_{j}^{\rm{Ni}}\right)$. Four scaling hyperparameters (*θ*_1_, *θ*_2_, *θ*_3_ and *θ*_4_) regulated the relative weighting of the four distances and were tuned during the training of GPs.

### Explaining the predictions of the best models

We used the SHAP algorithm to provide explanations for the ML model predictions. We used the Python implementation of SHAP, version 0.35.0, available via the conda-forge channel (https://anaconda.org/conda-forge/shap). SHAP combines game theory with local explanation, enabling accurate interpretations on how the model predicts a particular value for a given sample. The explanations are called local explanations and reveal subtle changes and interrelations that are otherwise missed when these differences are averaged out. Local explanations allow the inspection of samples that have extreme phenotype values (for example, a high or low photocatalytic reactivity).

We calculated SHAP values and built SHAP explainers for GP models trained on all 55 CNPs that were synthesized and tested in the first BO workflow (Fig. 3), obtaining insight into the overall model behaviours (Fig. 4a,b) and local explanations for individual predictions (Fig. 4c). Figure 4a,b shows the relative importance of the input features for the 55 CNPs. The features are sorted in descending order of importance from top to bottom. The average absolute SHAP value of an input feature (Fig. 4a) indicates the magnitude of the feature’s contribution to the model’s predictions; the larger the average absolute SHAP value, the greater the feature’s influence on the model’s predictions. Figure 4b reports the distribution of SHAP values for each feature, indicating the magnitude and direction of each feature’s contribution to the model’s prediction. Positive SHAP values indicate that the feature pushes the prediction higher, and negative SHAP values indicate that the feature pushes the prediction lower. Each data point in the plot represents the SHAP value of a specific feature for a single instance in the dataset. The points are arranged in a swarm manner along the *y* axis, with the density of the points giving a sense of the distribution of SHAP values for each feature.

In addition to global explanations, local explanations for individual predictions were also constructed by SHAP analysis (Fig. 4c). The force plot, shown in Fig. 4c, provide,s a detailed view of how the individual features contributed to the specific prediction for the best-performing catalyst, CNP-127 (Fig. 3d). The base value is the expected output of the model for the given instance, considering the average prediction across all instances in the dataset; that is, all 55 CNPs. The input features that pushed the prediction higher (above the base value) are indicated by the red arrows pointing to the right, with the length of the arrow proportional to the magnitude of the SHAP value. Similarly, the input features that pushed the prediction lower (below the base value) are indicated by the blue arrows pointing to the left.

Although SHAP analysis is valuable for determining the influence of different factors on a model’s predictions, it does not fully unravel the complex interactions between those factors, nor explain the internal workings of the model itself. Hence, while providing a degree of explainability, or interpretability, it does not by any means offer a complete explanation of the intricate chemistry underlying the performance of these CNPs. Indeed, the factor weightings are only useful when combined with expert chemical knowledge. Nevertheless, SHAP’s ability to offer both global and local explanations of a BO surrogate model’s predictions and uncertainties, as well as the resulting acquisition scores, can help us to gain insights into the BO algorithm’s decision-making process. This enhances the interaction between the optimization algorithm and human-in-the-loop knowledge integration. For instance, data requests that may initially appear non-obvious can be interpreted through SHAP analysis along three axes: ‘following positive contributions’, ‘avoiding negative contributions’ and ‘gaining knowledge contributions’. We acknowledge that this also presents some risk that such interpretations could introduce confirmation bias, but we feel that for many chemical problems, the benefits outweigh such risks.

## Online content

Any methods, additional references, Nature Portfolio reporting summaries, source data, extended data, supplementary information, acknowledgements, peer review information; details of author contributions and competing interests; and statements of data and code availability are available at 10.1038/s41557-024-01546-5.

### Supplementary information

Supplementary Information Materials and characterizations including NMR, UV–vis spectroscopy, figures and tables.

Supplementary Data Containing complete dataset for BO workflow, including dataset for BO training, molecular DFT descriptors and molecular structure_XYZ.

### Source data

Source Data Fig. 3 Data in excel.

Source Data Fig. 5 Data in excel.

Source Data Fig. 6 Data in excel.

Source Data Extended Data Fig./Table 1 Data in excel.

Source Data Extended Data Fig./Table 3 Data in excel.

## Data Availability

The data supporting the findings of this study are available within the paper and its Supplementary Information files. Source data are provided with this paper.

## Code availability

All Python scripts used in this study are available at https://github.com/Yuchees/bo_photocats.
