# Supplementary material for: Sequential closed-loop Bayesian optimization as a guide for organic molecular metallophotocatalyst formulation discovery
Source: Nat Chem. 2024 Jun 11;16(8):1286–94. doi: 10.1038/s41557-024-01546-5 (PMC11321994; doi:10.1038/s41557-024-01546-5)
Supplement: Supplementary file 1 — Materials and characterizations including NMR, UV–vis spectroscopy, figures and tables. [file 41557_2024_1546_MOESM1_ESM.pdf]

nature chemistry

Supplementary information https://doi.org/10.1038/s41557-024-01546-5

Sequential closed-loop Bayesian optimization as a guide for organic molecular metallophotocatalyst formulation discovery

In the format provided by the authors and unedited

Contents

Materials and characterizations 2

1. **Synthesis section** 2

General synthetic procedures for non-commercial Ra precursors 2

General synthetic procedures for CNP molecules 2

Synthetic procedure for CNP-624 3

2. **Photocatalytic tests** 4

Experimental setup for photoredox reactions 4

Procedures for photoredox reactions 5

3. **Computational details** 5

Structure generation 5

Molecular descriptors 6

4. **Searching for new active CNPs through machine learning prediction** 8

5. **Additional discussions on benchmarking the photocatalytic activity of CNP-127** 11

6. **Supplementary figures and tables** 12

7. **Characterization data for CNPs, provided in ID order.** 26

Cyclic voltammetry (CV) of CNPs 26

UV-vis absorption spectra of CNPs 39

NMR, elemental analysis, and mass spectrometry of CNPs 73

General methods

Materials and characterizations

Reagents and solvents used for the synthesis and photoredox reactions were obtained from Alfa Aesar, Sigma-Aldrich, Fluorochem, TCI Europe, Carbosynths, and used as received without further purification. The deionized water was produced using a Milli-Q System (ρ = 15 MΩ). ^{1}H and ^{13}C NMR spectra were measured on a Bruker Avance 400 NMR spectrometer at 400 MHz and 100 MHz, respectively. UV-visible absorption spectra were recorded on a Shimadzu UV-2550 UV-visible spectrometer at room temperature.

1. Synthesis section

General synthetic procedures for non-commercial Ra precursors

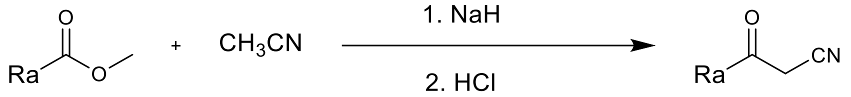

NaH (50-60% dispersion in mineral oil, 13 mmol) was carefully discharged into 250 mL RBF under N_2, then 100 mL dry CH_3CN was injected. The suspension was stirred at room temperature for 30 minutes. After that, the aryl methyl carboxylate (6.5 mmol) was charged into the reaction mixture. The reaction was heated to 80°C for 4 h, and the product precipitated from the reaction mixture as a sodium salt. Upon cooling, the salt product was filtered and washed with CH_3CN, and then dissolved in water. The final product was precipitated from the solution by adjusting the pH of solution to about 6 with HCl. After washing with water, the product was collected.

General synthetic procedures for CNP molecules

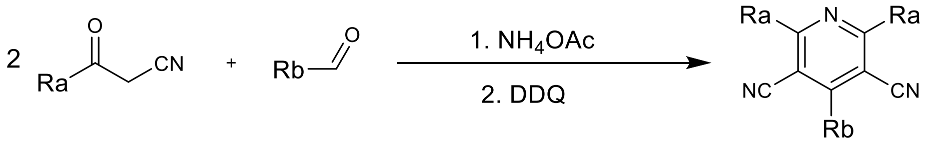

Scheme S1: General scheme of Hantszch pyridine synthesis reaction for CNP molecule synthesis. The CNP molecules were synthesised using classical Hantszch pyridine synthesis reaction: 3 mmol Ra, 1.5 mmol Rb, and 7.5 mmol ammonium acetate were charged into the reaction flask, then acetic acid (15 mL) was added. The reaction mixture was heated under reflux to 110 °C overnight. The resultant precipitate was filtered and washed with methanol. Then, the solids were oxidised with 2,3-dichloro-5,6-dicyano-*p*-benzoquinone (DDQ) in acetic acid solution under 110 °C for 1

hour, followed by filtration and methanol washing. The solids were purified by preparative column chromatogrpahy, giving the final product (the overall yield was greater than 50 % for most of the CNP molecules; the purity was confirmed by ^{1}H NMR spectroscopy and elemental analysis).

Synthetic procedure for CNP-624

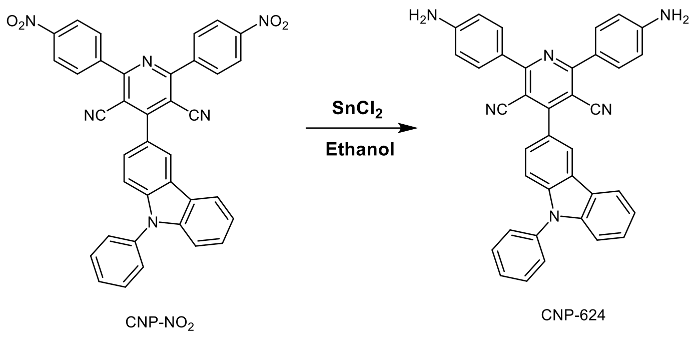

CNP-624 was synthesised as follows. CNP-NO_2 (1 mmol) and $SnCl_2$ (10 mmol) were charged into a solution of ethanol (50 mL) and ethyl acetate (50 mL). The solution was refluxed overnight. After reaction, the solution was removed by rotary evaporation, then saturated aqueous $NaHCO_3$ solution was added (100 mL), and the solid was filtered and washed with methanol. CH_2Cl_2 was added to extract the products from the solid. After purification by column chromatography, CNP-624 was collected (yield = 22%).

2. Photocatalytic tests

Experimental setup for photoredox reactions

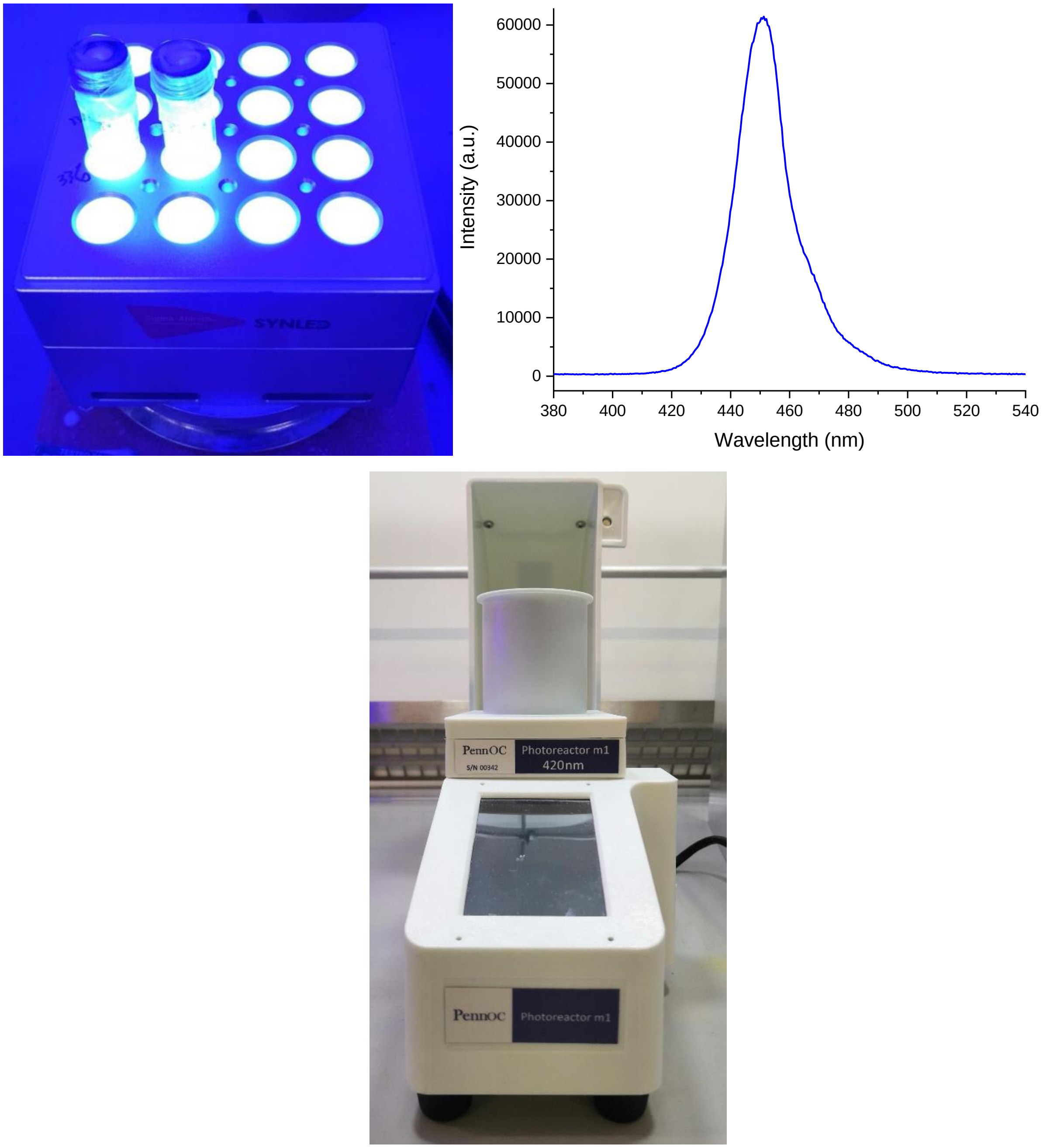

Supplementary Figure 1. Lamp source used for BO-guided CNP discovery and photoredox reaction optimisation experiments; also, the emission spectra of the Synled blue LED source (upper); experimental setup used for photoredox reactions under 420 LED illumination (lower).

Procedures for photoredox reactions

Condition for BO-led CNP search:

Aromatic halides (0.10 mmol, 1 equiv), Boc-Pro-OH (0.15 mmol, 1.5 equiv), Cs_2CO_3 (0.15 mmol, 1.5 equiv), CNP (0.004 mmol, 0.04 equiv), $NiCl_2$ glyme (0.01 mmol, 0.1 equiv), 4,4-di-tert-butyl-2,2-bipyridyl (0.015 mmol, 0.15 equiv) and 4 ml DMF were charged into a glass vial (Fisherbrand, Cat. No. 10504463) equipped with screw cap (SUPELCO Lot: 134901) and a magnetic stir bar. The reaction mixture was degassed by bubbling nitrogen stream for 5 min, then irradiated under Blue LED (SYNLED) for 3 h. The yield of product was calculated by GC-MS using biphenyl as a calibration standard.

Condition for BO reaction condition optimisation:

Aromatic halides (0.10 mmol, 1 equiv), Boc-Pro-OH (0.15 mmol, 1.5 equiv), Cs_2CO_3 (0.15 mmol, 1.5 equiv), the corresponding CNP (0.004 mmol, 0.04 equiv), $NiCl_2$ glyme, ligand and 4 ml DMF were charged into a glass vial (Fisherbrand, Cat. No. 10504463) equipped with screw cap (SUPELCO Lot: 134901) and magnetic stir bar. The reaction mixture was degassed by bubbling nitrogen stream for 5 min, then irradiated under Blue LED (SYNLED) for 21 h. Again, the yield of product was calculated by GC-MS using biphenyl as standard.

3. Computational details

Structure generation

To generate atomistic structures for the molecules, the simplified molecular-input line-entry system (SMILES) representation of each molecule was converted to a 3D structure using Open Babel. The gen3d operation was used, which starts with 250 steps of steepest-descent geometry optimization with the MMFF94 forcefield, followed by 200 iterations of a Weighted Rotor conformational search, before a final 250-step conjugated-gradient geometry optimization. The resulting 3D structure was subjected to a further conformer search, generating 50 conformers. The lowest-energy conformer from the search was finally geometry-optimized at the CAM-B3LYP level of theory and in n,n-DiMethylFormamide with the SMD solvation model. The 6-31G* basis set was used for all atoms, except for Br and I atoms the “double-ζ” quality LANL2DZ basis set was used. All (TD-)DFT calculations were performed using Gaussian 16 software.

Molecular descriptors

A total of 16 descriptors were calculated for all the 560 CNPs: IP, EA, EA*, IP*, $\Delta E_{S1\rightarrow S0}$, OS, $\Delta E_{S1\rightarrow T1}$, S_r, D index, ΔD, Δσ, H_{CT}, H index, t index, E_c, and λ_{int}.

IP, EA, EA* and IP* are standard reduction potentials of half-reactions for free electrons/holes and excitons and were calculated using (TD-)DFT.

The first singlet excited state, S_1, and the first triplet excited state, T_1, were determined by TD-DFT, with n,n-DiMethylFormamide as the solvent using the SMD solvation model. $\Delta E_{S1\rightarrow S0}$ is the energy difference between S_1 and the ground state, S_0; OS is the oscillator strength of S_1. $\Delta E_{S1\rightarrow S0}$ is also referred to as the calculated optical gap in this study. $\Delta E_{S1\rightarrow T1}$ is the energy difference between S_1 and T_1. The smaller the $\Delta E_{S1\rightarrow T1}$ value, the larger the spin-orbital coupling, and ultimately the more probable and faster the intersystem crossing.

S_r, D, ΔD, Δσ, H_{CT}, H, and E_c are descriptors from quantitative characterization of hole and electron distributions in real space, performed for S_1 on the optimized, ground-state geometry, using the Multiwfn software. Briefly:

S_r index quantifies the overlap between the hole distribution ($\rho^{hole}(\boldsymbol{r})$) and the electron distribution ($\rho^{electron}(\boldsymbol{r})$). S_r varies between 0 (no overlap) and 1 (complete overlap); the larger the value is, the greater the extent of overlap is.

D index quantifies the distance between the centres of the hole and the electron, which characterizes the charge transfer length.

ΔD is the difference in dipole moment between the excited-state and the ground-state of the molecule—*i.e.*, $\Delta D = D_{excited\text{-}state} - D_{ground\text{-}state}$—a measure of the extent of charge redistribution between the two states.

Δσ index is the difference between $\boldsymbol{\sigma}_{electron}$ and $\boldsymbol{\sigma}_{hole}$, given by

$\Delta\sigma$ index = | $\boldsymbol{\sigma}_{electron}$ | - | $\boldsymbol{\sigma}_{hole}$ |,

where $\boldsymbol{\sigma}_{electron}$ and $\boldsymbol{\sigma}_{hole}$ are a measure of the sparsity of $\rho^{electron}(\boldsymbol{r})$ and $\rho^{hole}(\boldsymbol{r})$, respectively. $\Delta\sigma$ index can be positive or negative for different molecules.

H_{CT} is the average of $\boldsymbol{\sigma}_{electron}$ and $\boldsymbol{\sigma}_{hole}$ in the charge-transfer (CT) direction, given by

H_{CT} = | $\mathbf{H} \cdot \mathbf{u}_{CT}$ |,

where $\mathbf{H} = (\boldsymbol{\sigma}_{electron} + \boldsymbol{\sigma}_{hole})/2$ and $\mathbf{u}_{CT}$ is the unit vector along the CT direction.

H index is simply taken as $(|\boldsymbol{\sigma}_{electron}| + |\boldsymbol{\sigma}_{hole}|)/2$.

The t index is a measure of the degree of separation between hole and electron along the CT direction and is given by t index = D index – H_{CT}.

E_c is the Coulomb attraction between hole and electron, which can be considered a measure of exciton binding energy, given by $E_c = \int\int \frac{\rho^{hole}(\boldsymbol{r_1})\rho^{electron}(\boldsymbol{r_2})}{|\boldsymbol{r_1}-\boldsymbol{r_2}|} d\boldsymbol{r_1} d\boldsymbol{r_2}$.

λ_{int}, internal reorganization energy, is given by

$\lambda_{int} = \frac{1}{2}(E_0^- - E_0 + E_-^0 - E_-)$

where E_0 is the energy of the charge-neutral state of the charge-neutral geometry, E_- is the energy of the -1 charged state of the -1 charged geometry, E_0^- is the energy of the charge-neutral state of the -1 charged geometry, and E_-^0 is the energy of the -1 charged state of the charge-neutral geometry.

4. Searching for new active CNPs through machine learning prediction

The structure-activity relationship for CNPs with carbazole Rb groups (CNP-C) was further investigated since they were prioritized by the BO algorithm and gave high photocatalytic yields compared to other Rb groups. Supplementary Figure 3a and 3b shows that the Ra group has a significant effect on the yields for the CNP-C materials, with electron donating groups giving the highest yields, followed by halogen-bearing Ra groups, then electron withdrawing groups. Rb variations in the carbazole groups had a relatively small effect on the reaction yield. For D-A type molecules, the energy of ionization potential is dominated by donating part, while $E_{1/2}^{red}(CNP/CNP^-)$ (electron affinity, EA) is controlled by accepting part. Thus, by varying Ra groups, the tunability of $E_{1/2}^{red}(CNP/CNP^-)$ in the CNP can be achieved. Supplementary Figure 3b and Supplementary Table 1 show the photoredox reaction yields of CNP-C samples as a function of the experimentally measured $E_{1/2}^{red}(CNP/CNP^-)$ and their corresponding Ra groups. In general, donating groups raise $E_{1/2}^{red}(CNP/CNP^-)$ to more negative energy level; conversely withdrawing groups lower $E_{1/2}^{red}(CNP/CNP^-)$, and CNP-C with halogen Ra have $E_{1/2}^{red}(CNP/CNP^-)$ values that fall in between. We observed a strong linear correlation between calculated EA values and experimentally measured reduction potentials, $E_{1/2}^{red}(CNP/CNP^-)$, of CNP-C (Supplementary Figure 3c), validating our use of predicted energy levels in the BO selection. There seemed to be a weak negative correlation between CNP-C materials with low reduction potentials and high photocatalysis yields, except for two outliers with naphthalene Ra groups (CNP-346, Ra13-Rb011; CNP-351, Ra13-Rb016), which showed low activity (Supplementary Figure 3a,b). The reduction potential for the CNP estimates the driving force of the second electron transfer from the reduced CNP^- to Ni catalyst, a key step that couples the PC and nickel catalyst catalytic cycles.[1, 2]

The weak experimental correlation between reduction potentials of CNPs with carbazole groups and photocatalytic yield (Supplementary Figure 3b) inspired us to search for CNPs with more negative calculated reduction potentials in the virtual library of 560 CNPs. CNP-239 and CNP-234, were both explored experimentally by BO but they showed less good performance, despite having the strongest reduction potentials in the library (-1.92 V vs Fc^+/Fc and -1.91 V vs Fc^+/Fc, respectively). Then, we designed an expanded virtual library consisting of 100 additional CNP molecules (Supplementary Figure 4), which comprises donating Ra groups and carbazole Rb groups to ensure that the designed CNPs have proper electron potentials to proceed the photocatalytic process. Noted that synthesisability was not prioritized in this design to allow a greedy search. It was found that CNP-624 has a calculated reduction potential of -2.17 V vs SHE; the experimentally measured reduction potential was -2.07 V vs Fc^+/Fc. However, no activity was observed for the cross-coupling reaction using CNP-624. We note here that the correlation of reduction potential with yield fails to apply to the whole set of CNP molecules (Supplementary

Figure 7). Again, these observations indicate that the performance of CNPs is determined by multiple factors, rather than a single photophysical feature, rationalizing the use of a BO-led search strategy rather than more classical computational design.

We then conducted an evaluation of multiple ML models to predict the reaction yields of the 70 CNPs synthesized during the first BO workflow. The models we assessed included Gaussian Processes (GP), Kernel Ridge Regression (KRR), Gradient-Boosted Decision Trees (GBDT), Random Forests (RF), and Support Vector Machines (SVM). Our model assessment process involved training these ML models on the 55 CNPs recommended by BO and then testing their performance on the 15 CNPs selected based on structural diversity. For input to the ML models, we used the 16 molecular descriptors capturing various optoelectronic properties of the CNPs. We employed a 5-fold cross-validation approach for training these models. The GP-based models emerged as the most performant among the tested models. The performance details of all evaluated ML models are summarized in Supplementary Table 2. Additionally, Supplementary Figure 5 offers a visual comparison between the GP models' predicted reaction yields and their actual experimental values. This comparison demonstrates that the GP models accurately predicted the reaction yields for both the set of 55 CNPs recommended by BO and the set of 15 molecules selected for structural diversity.

Furthermore, we trained GP regression models using all 70 CNPs synthesized during the first BO workflow. These models were subsequently employed to predict the reaction yields for 100 newly designed CNPs (Supplementary Figure 4). The top 12 predictions made by the GP model are presented in Supplementary Figure 6. Notably, none of the 100 new CNPs were predicted to surpass CNP-127 in terms of photocatalytic activity. We synthesized and tested three CNPs from this new set—specifically, CNP-561 (predicted to be the most active), CNP-565, and CNP-577. All three CNPs shared the Rb16 carbazole group but differed in their Ra groups. The experimental yields for CNP-561, CNP-565, and CNP-577 were 55%, 59%, and 60%, respectively, compared to their predicted yields of 62%, 59%, and 59%. We note that these three CNPs were not subjected to any optimization of reaction conditions. Having experimentally evaluated a total of 75 CNPs—55 recommended by BO, 15 selected for structural diversity, 2 additional neighbours of CNP-127, and 3 from the new set—we are inclined to believe that CNP-127 is either the most active or one

of the top photocatalysts among all CNPs considered in this study. This outcome indicates that the initial BO workflow was highly effective in identifying CNP-127 as a leading candidate.

5. Additional discussions on benchmarking the photocatalytic activity of CNP-127

To understand the dependence of reaction yield on nickel concentration for CNP-127, Ir-cat, and 4CzIPN, we compiled their reaction yields and conversion rates across various nickel concentrations in Supplementary Table 4. Our analysis revealed that Ir-cat exhibited a lower rate of the $C(sp^3)$-$C(sp^2)$ cross-coupling reaction compared to CNP-127 at lower Ni concentrations, as evidenced by kinetic curves in Supplementary Figure 8. These curves compare the reaction progression at a 2 mol% nickel concentration for Ir-cat and CNP-127. For 4CzIPN, we observed a decrease in selectivity for the $C(sp^3)$-$C(sp^2)$ cross-coupling reaction as nickel loading decreased. No significant by-products were detected in the GC-MS spectrum examined in our study.

Supplementary Figure 9 further illustrates the relationship between reaction yields and the reduction potential Exp-$E_{1/2}^{red}$(CNP/CNP$^-$) for CNPs with carbazole Rb groups at a 1 mol% nickel concentration, with data given in Supplementary Table 3. Mirroring the trends observed at 10 mol% nickel concentration (Supplementary Figure 3b), we noted a weak negative correlation between CNP-C molecules with low reduction potentials and high reaction yields. When nickel concentration was further reduced to 0.5 mol% (Supplementary Figure 9b), only CNP-239 and CNP-234, with the strongest reduction potentials among the 560 CNPs, showed substantial photocatalytic activities, with reaction yields of 75% and 73%, respectively. While conducted in a glovebox or in air, trace yields (< 1%) were observed on CNP-122 and CNP-127 at 0.5 mol% nickel concentrations. This indicates the existence of threshold reduction potential values necessary to initiate the photocatalytic process at lower nickel concentrations ($\leqslant$ 1 mol%), a prerequisite not required at higher nickel concentrations. These observations underscore that the performance of organic molecular photocatalysts in nickel/photoredox dual cross-coupling reactions is closely tied to specific reduction potential thresholds, which are in turn influenced by nickel concentrations. Note that the nickel loading concentration dependence is influenced by the structure of organic molecular photocatalysts, as seen in the case of CNP-519 and CNP-419, with a reduction potential of -1.70 V similar to 4CzIPN. However, they exhibit less dependence on nickel loading, as shown in Supplementary Table 5.

6. Supplementary figures and tables

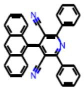
ID: 3 Step: 0

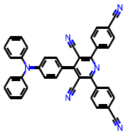
ID: 65 Step: 0

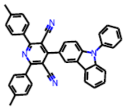
ID: 129 Step: 0

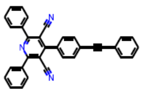
ID: 11 Step: 0

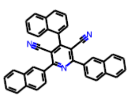
ID: 336 Step: 0

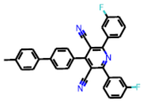
ID: 415 Step: 0

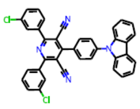
ID: 439 Step: 1

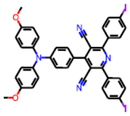
ID: 494 Step: 1

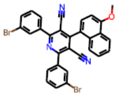
ID: 372 Step: 1

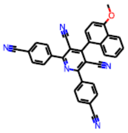
ID: 64 Step: 1

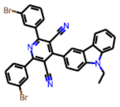
ID: 379 Step: 1

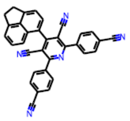
ID: 58 Step: 1

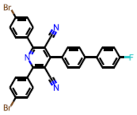
ID: 110 Step: 2

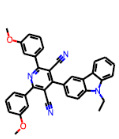
ID: 463 Step: 2

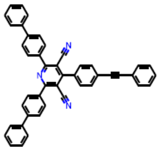
ID: 207 Step: 2

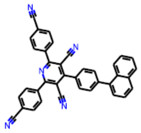
ID: 69 Step: 2

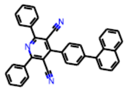
ID: 13 Step: 2

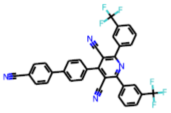
ID: 559 Step: 2

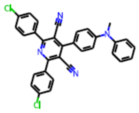
ID: 302 Step: 3

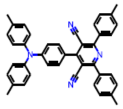
ID: 137 Step: 3

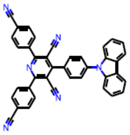
ID: 75 Step: 3

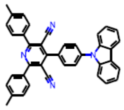
ID: 131 Step: 3

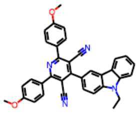
ID: 239 Step: 4

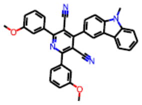
ID: 464 Step: 4

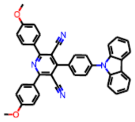
ID: 243 Step: 4

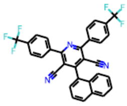
ID: 168 Step: 4

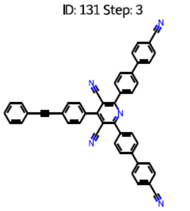
ID: 263 Step: 4

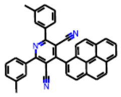
ID: 328 Step: 4

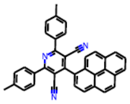
ID: 132 Step: 4

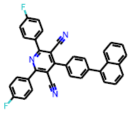
ID: 153 Step: 4

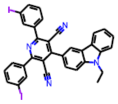
ID: 519 Step: 5

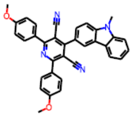
ID: 240 Step: 5

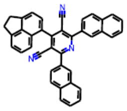
ID: 338 Step: 5

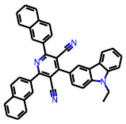
ID: 351 Step: 5

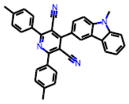
ID: 128 Step: 5

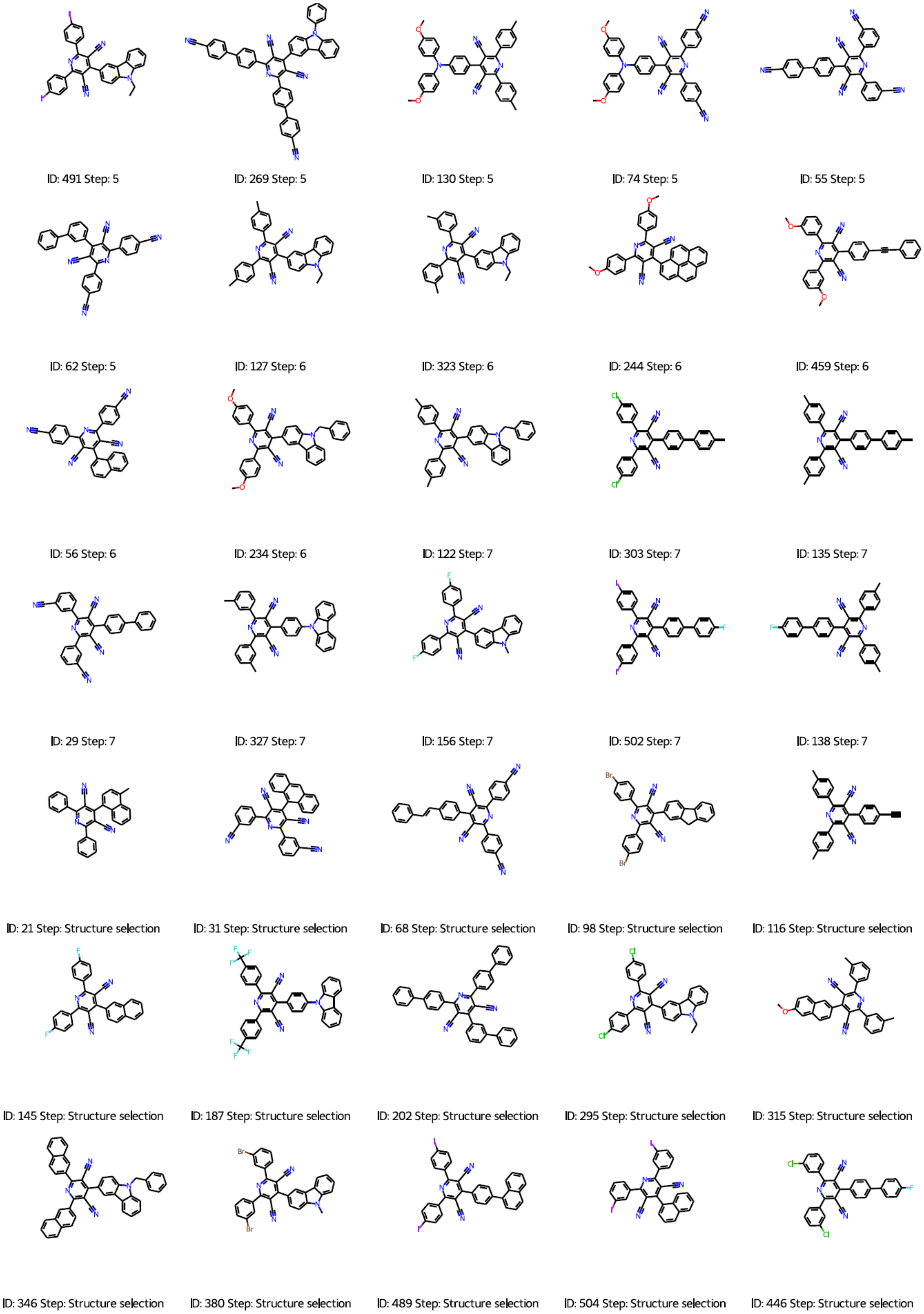

Supplementary Figure 2. Molecular structures of the experimentally tested CNPs-ID, shown in BO step order.

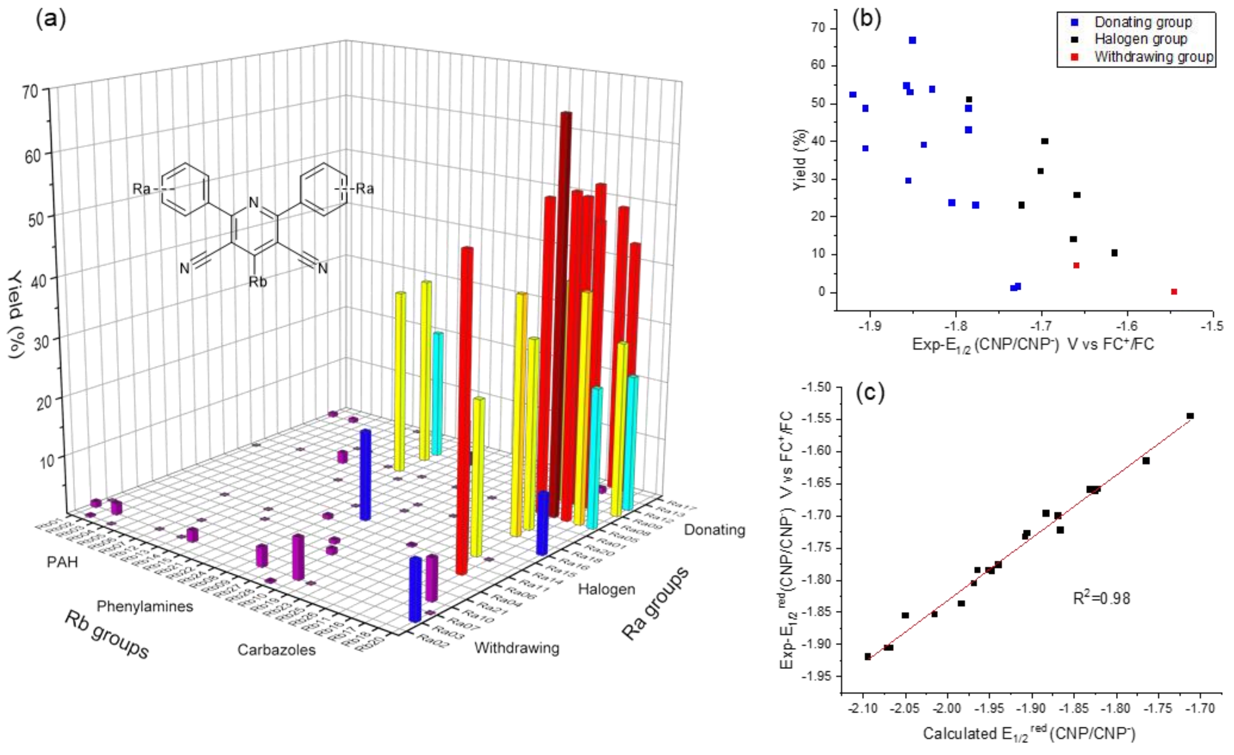

Supplementary Figure 3. Structure-activity relationships for CNP photoredox catalysts. (a) Plot showing the yield for the experimentally measured CNPs as a function of their Ra and Rb components (containing the structurally selected control set). (b) Plot showing the dependence of yield as a function of Exp-$E_{1/2}^{red}$(CNP/CNP$^-$) for CNPs bearing carbazole Rb groups. The colour of the points in this plot represent different Ra groups (electron donating, electron withdrawing, and halogen-bearing), which shows how the Ra groups modulate Exp-$E_{1/2}^{red}$(CNP/CNP$^-$). These data are also listed in Supplementary Table S1. (c) Linear correlation between calculated $E_{1/2}^{red}$(CNP/CNP$^-$) and experimentally measured Exp-$E_{1/2}^{red}$(CNP/CNP$^-$) for CNPs with carbazole Rb groups.

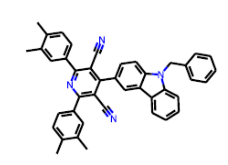
ID: 560

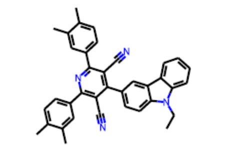
ID: 561

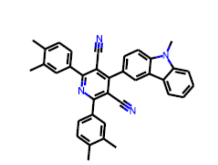
ID: 562

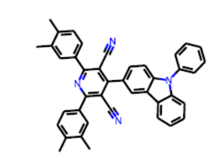
ID: 563

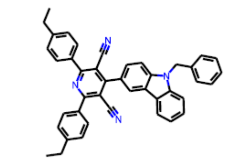
ID: 564

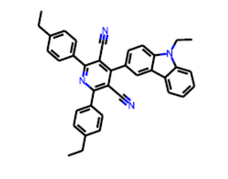
ID: 565

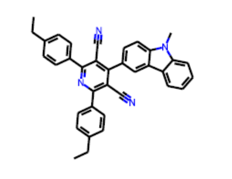
ID: 566

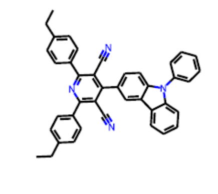
ID: 567

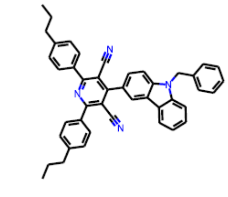
ID: 568

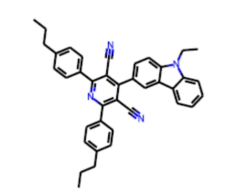
ID: 569

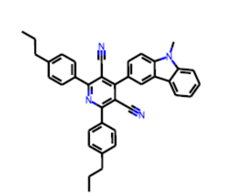
ID: 570

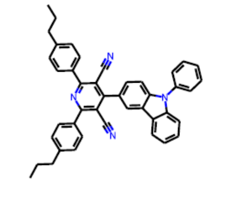
ID: 571

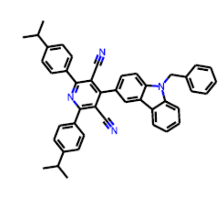
ID: 572

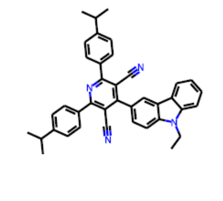
ID: 573

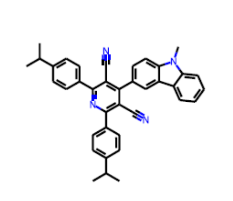
ID: 574

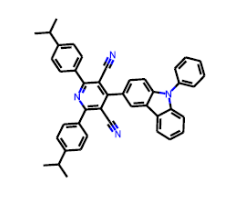
ID: 575

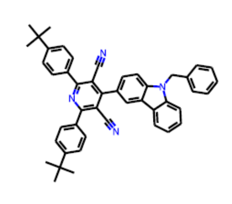
ID: 576

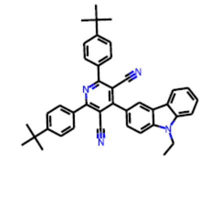
ID: 577

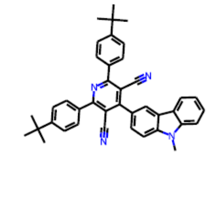
ID: 578

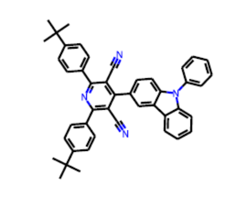
ID: 579

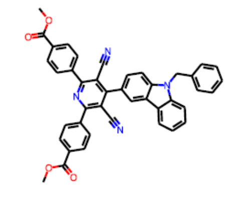
ID: 580

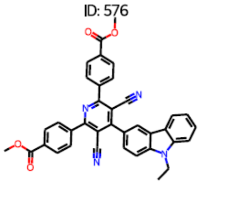
ID: 581

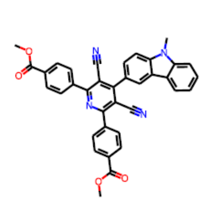
ID: 582

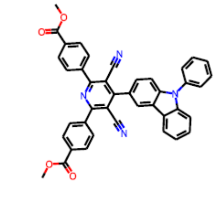
ID: 583

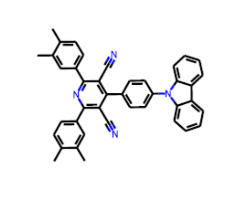
ID: 590

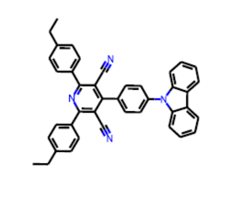
ID: 591

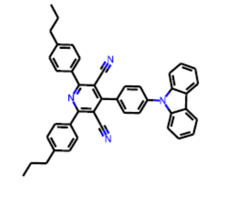
ID: 592

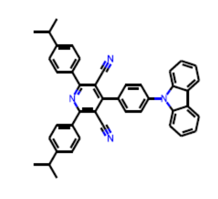
ID: 593

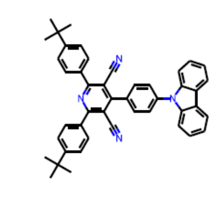
ID: 594

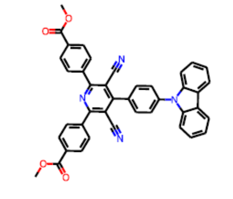
ID: 595

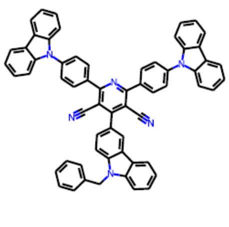
ID: 596

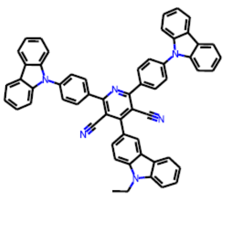
ID: 597

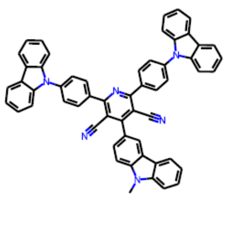
ID: 598

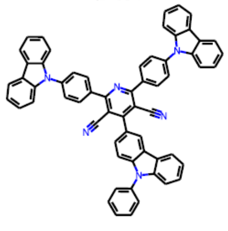
ID: 599

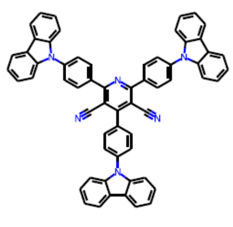
ID: 600

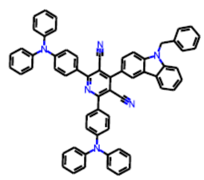
ID: 601

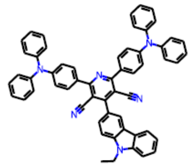
ID: 602

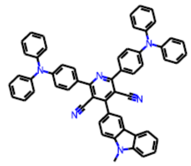
ID: 603

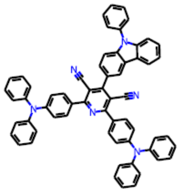
ID: 604

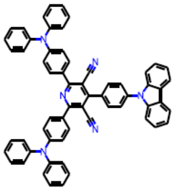
ID: 605

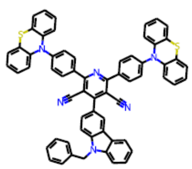
ID: 606

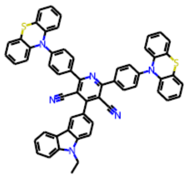
ID: 607

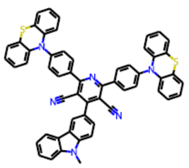
ID: 608

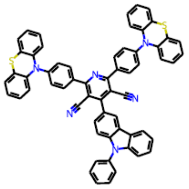
ID: 609

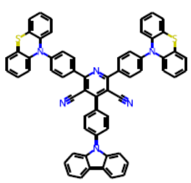
ID: 610

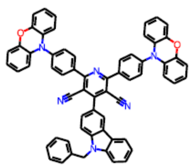
ID: 611

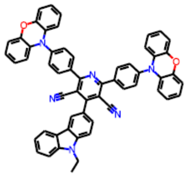
ID: 612

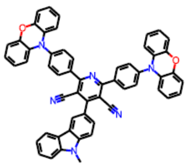
ID: 613

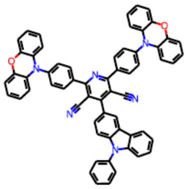
ID: 614

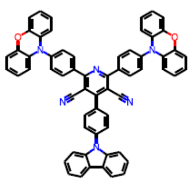
ID: 615

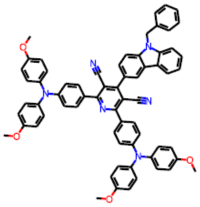
ID: 616

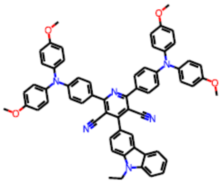
ID: 617

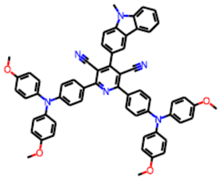
ID: 618

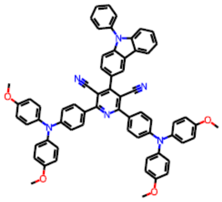
ID: 619

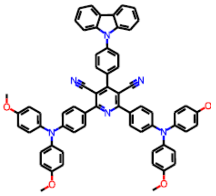
ID: 620

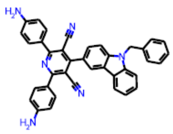
ID: 621

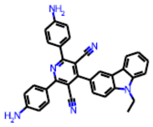
ID: 622

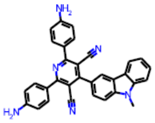
ID: 623

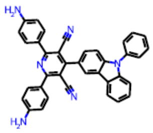
ID: 624

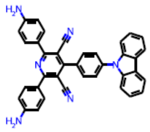
ID: 625

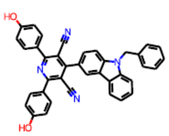
ID: 626

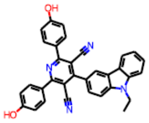
ID: 627

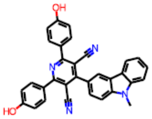
ID: 628

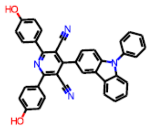
ID: 629

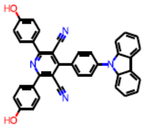
ID: 630

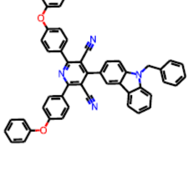
ID: 631

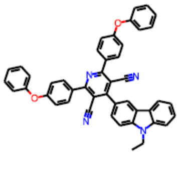
ID: 632

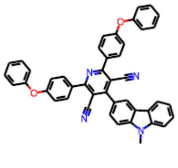
ID: 633

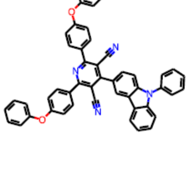
ID: 634

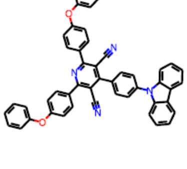
ID: 635

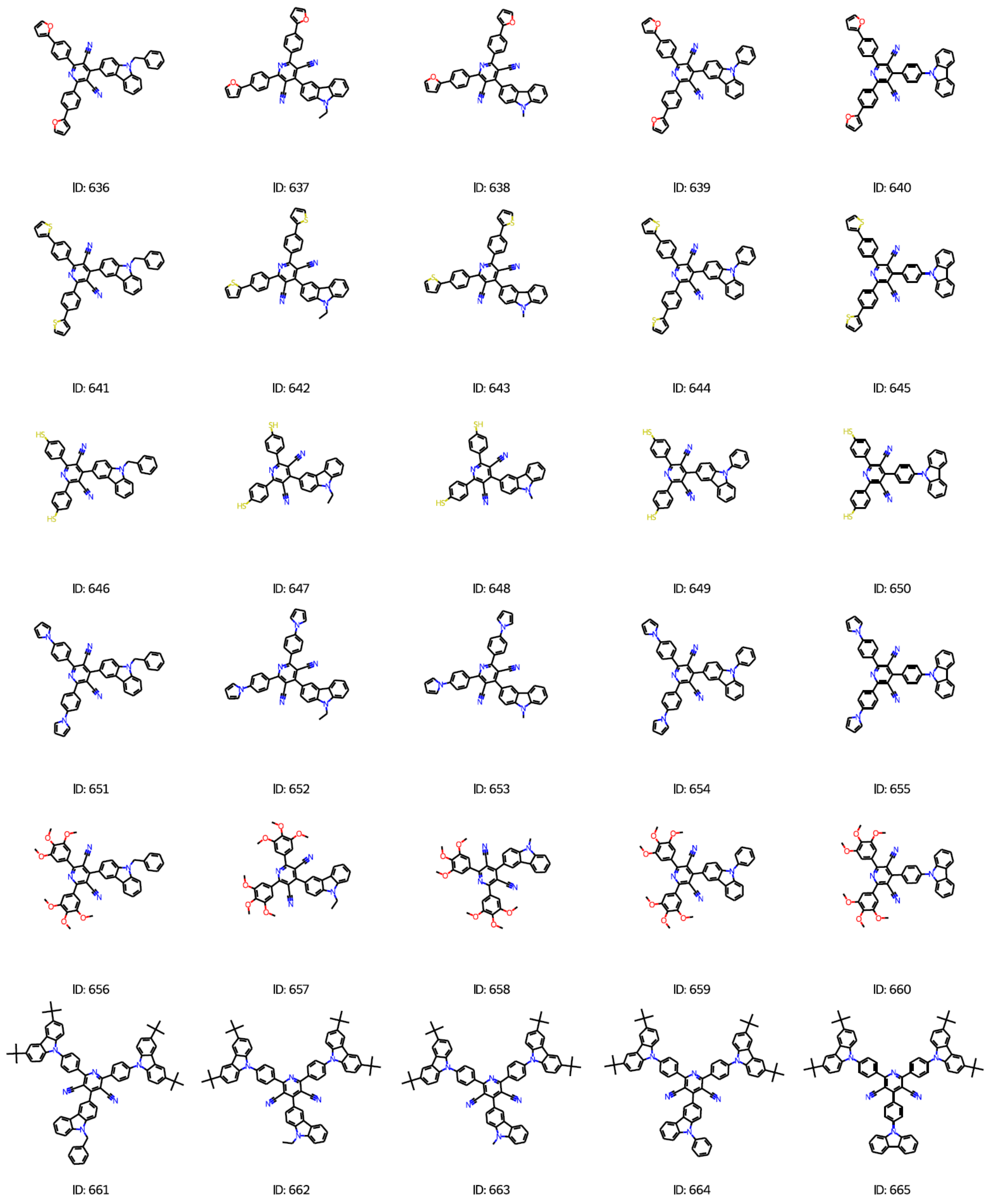

Supplementary Figure 4. Molecular structures of the 100 additional CNPs-ID designed for machine learning screening.

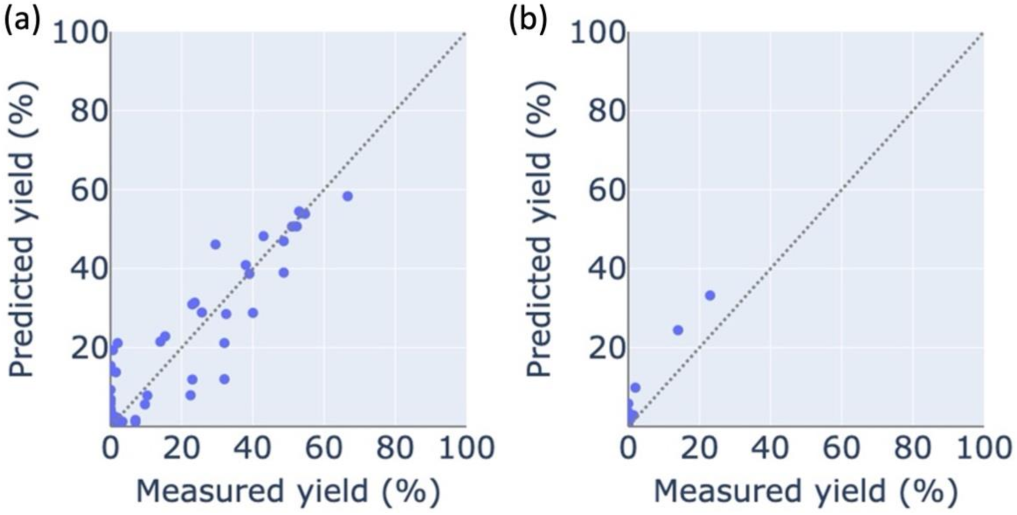

Supplementary Figure 5. Prediction performance of GP models on the experimental data from the first BO optimization step. (a) GP models trained by 5-fold cross-validation using the 55 CNPs synthesized on BO recommendation. (b) Prediction of reaction yields for the 15 CNPs selected for structural diversity by the GP models trained on the 55 CNPs from the BO workflow.

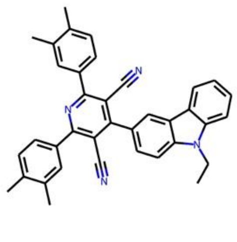

ID: 561 name: Ra22_Rb016 Yield: 62%

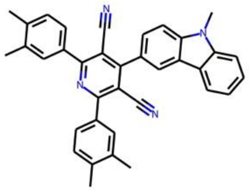

ID: 562 name: Ra22_Rb017 Yield: 60%

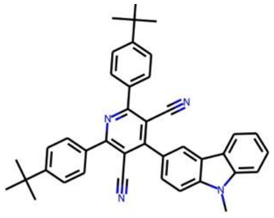

ID: 578 name: Ra26_Rb017 Yield: 59%

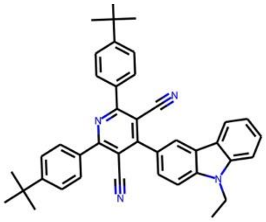

ID: 577 name: Ra26_Rb016 Yield: 59%

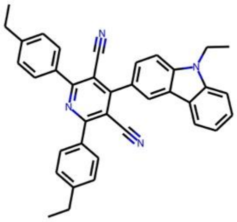

ID: 565 name: Ra23_Rb016 Yield: 59%

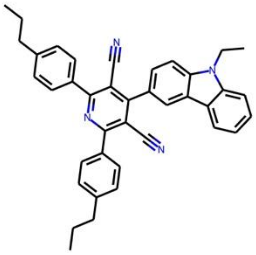

ID: 569 name: Ra24_Rb016 Yield: 58%

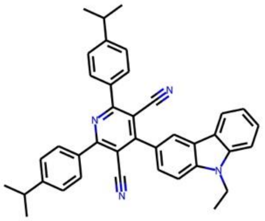

ID: 573 name: Ra25_Rb016 Yield: 57%

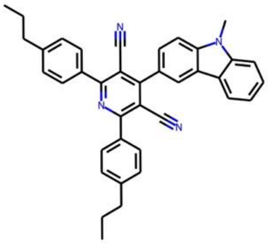

ID: 570 name: Ra24_Rb017 Yield: 57%

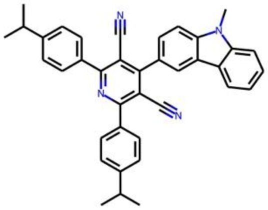

ID: 574 name: Ra25_Rb017 Yield: 56%

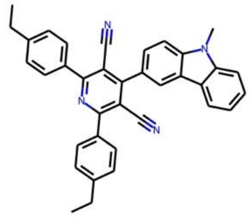

ID: 566 name: Ra23_Rb017 Yield: 56%

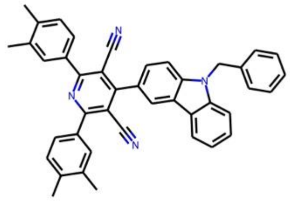

ID: 560 name: Ra22_Rb011 Yield: 54%

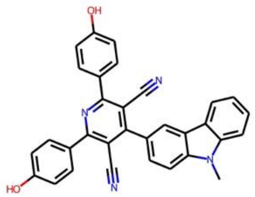

ID: 628 name: Ra34_Rb017 Yield: 53%

Supplementary Figure 6. Additional CNPs-ID screened by machine learning models. The top 12 predictions per the ML models trained on the 70 CNPs experimentally evaluated in the first BO campaign are shown here, with their predicted yields. CNP-561, CNP-565, and CNP-577 were evaluated experimentally, achieving reaction yields of 55%, 59%, and 60%, respectively.

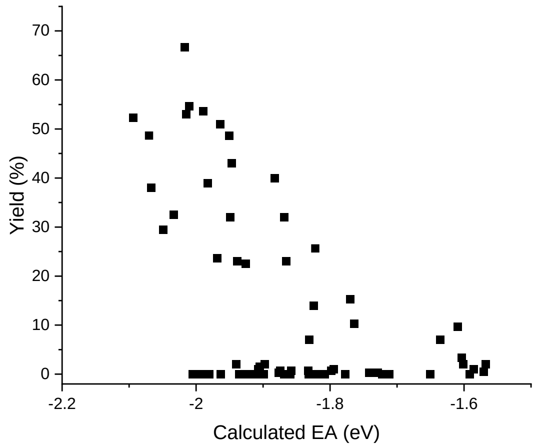

Supplementary Figure 7. Plot of reaction yield versus calculated reduction potential for the experimentally measured CNPs.

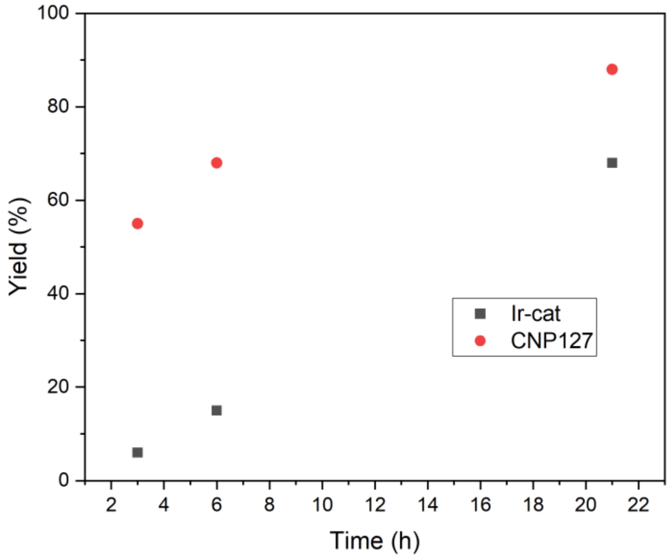

Supplementary Figure 8. Kinetic curves of reaction yields over time at 2 mol% Ni concentration for Ir-cat and CNP-127. Conditions: 2 mol% $NiCl_2$·glyme (glycol ether), 3 mol% L2 ligand, 1.5 equivalents Cs_2CO_3 base, DMF, and blue LED irradiation source.

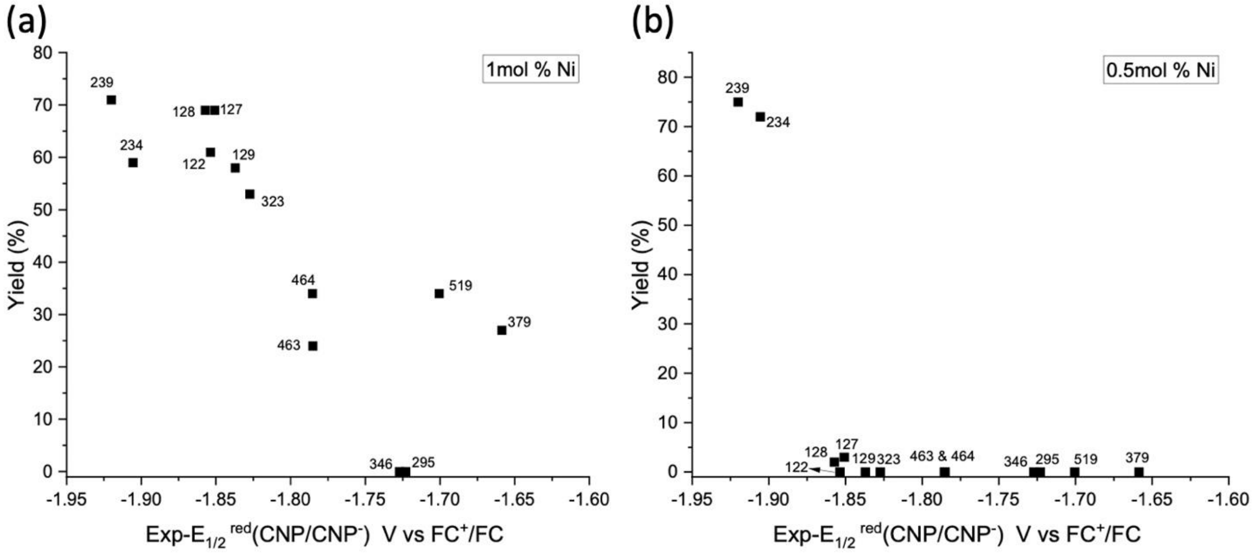

Supplementary Figure 9. Reaction yields plotted as a function of the experimentally measured reduction potentials, Exp-$E^{1/2}$red(CNP/CNP$^-$) for CNPs bearing carbazole Rb groups at nickel concentrations of 1 mol% (a) and 0.5 mol% (b). Conditions: 4 mol% photocatalyst, X mol% $NiCl_2$·glyme (glycol ether), X×1.5 mol% L3 ligand, 1.5 equivalents Cs_2CO_3 base, DMF, and blue LED irradiation source with irradiation time 21 hours.

Supplementary Table 1. Summary of data in Figure 4b.

Ra - Rb pair	CNP-ID	Exp-$E_{1/2}^{red}$(CNP/CNP-) V vs FC^+/FC	Yield (%)
Ra05_Rb016	127	-1.85	67
Ra05_Rb017	128	-1.86	55
Ra12_Rb016	323	-1.83	54
Ra05_Rb011	122	-1.85	53
Ra09_Rb016	239	-1.92	52
Ra06_Rb017	156	-1.78	51
Ra17_Rb016	463	-1.79	49
Ra09_Rb017	240	-1.91	49
Ra17_Rb017	464	-1.79	43
Ra18_Rb016	491	-1.70	40
Ra05_Rb018	129	-1.84	39
Ra09_Rb011	234	-1.91	38
Ra20_Rb016	519	-1.70	32
Ra09_Rb020	243	-1.86	30
Ra14_Rb016	379	-1.66	26
Ra05_Rb020	131	-1.80	24
Ra12_Rb020	327	-1.78	23
Ra11_Rb016	295	-1.72	23
Ra14_Rb017	380	-1.66	14
Ra16_Rb020	439	-1.61	10
Ra10_Rb018	269	-1.66	7
Ra13_Rb011	346	-1.73	2
Ra13_Rb016	351	-1.73	1
Ra07_Rb020	187	-1.54	0

Supplementary Table 2. Performance metrics of various machine-learning models for the experimental data points from the first BO workflow.

Model	5-fold cross-validation on the 55 CNPs from the BO workflow		Prediction of the 15 CNPs selected for structural diversity by the models trained on the 55 CNPs from the BO workflow
	R2	MAE [a]	MAE
GP	0.89	0.04	0.043
KRR	0.89	0.041	0.047
GBDT	0.86	0.047	0.043
RF	0.75	0.071	0.065
SVR	0.68	0.086	0.135

[a] Computed as the average of 5 MAE values, each derived from its respective test set in the 5 iterations of the cross-validation loop.

Supplementary Table 3. Summary of yields of CNPs at different nickel loadings.

CNP-ID	Nickel loading (mol%)	Yield (%)
295	1	0
463	1	24
379	1	27
519	1	34
464	1	34
323	1	53
129	1 (10)	58 (39)
234	1 (10)	59 (38)
122	1	61
128	1 (10)	69 (55)
127	1 (10)	69 (67)
239	1 (10)	71 (52)
491	1	51
240	10	49

Supplementary Table 4. Reaction yields and corresponding conversion rates (in parentheses) for CNP-127, Ir-cat, and 4CzIPN at various nickel concentrations.

Nickel loading / mol%	Ir-cat / % (%)	4CzIPN / % (%)	CNP-127 / % (%)
10	93 (>99)	77 (>99)	80 (>99)
8	94 (>99)	64 (>99)	74 (>99)
6	89 (>99)	44 (>99)	84 (>99)
4	50 (55)	4 (>99)	88 (>99)
2	68 (71)	8 (61)	88 (>99)
1	59 (62)	< 1 (3)	

Conditions: 4 mol% photocatalyst, X mol% $NiCl_2$·glyme (glycol ether), X×1.5 mol% L2 ligand, 1.5 equivalents Cs_2CO_3 base, DMF, and blue LED irradiation source with irradiation time 21h. The conversion rates were calculated based on aromatic halides.

Supplementary Table 5. Dependence of reaction yield on nickel loading for CNP-491, CNP-519, and 4CzIPN.

Nickel loading / mol%	4CzIPN / % (%)	CNP-491 / % (%)	CNP-519 / % (%)
10	75 (> 99)	61 (> 99)	52 (> 99)
1	< 1 (3)	51 (> 99)	34 (> 99)

Conditions: 4 mol% photocatalyst, X mol% $NiCl_2$·glyme (glycol ether), X×1.5 mol% L3 ligand, 1.5 equivalents Cs_2CO_3 base, DMF, and blue LED irradiation source with irradiation time 21h. Value in the parentheses are conversion rates calculated based on aromatic halides.

7. **Characterization data for CNPs, provided in CNP-ID order.**

Cyclic voltammetry (CV) of CNPs

Condition: 5 mg in 20 mL DMF with 0.1 M $TPABF_6$, Ag/AgCl-reference electrode, Pt wire-counter electrode, Glass carbon- working electrode.

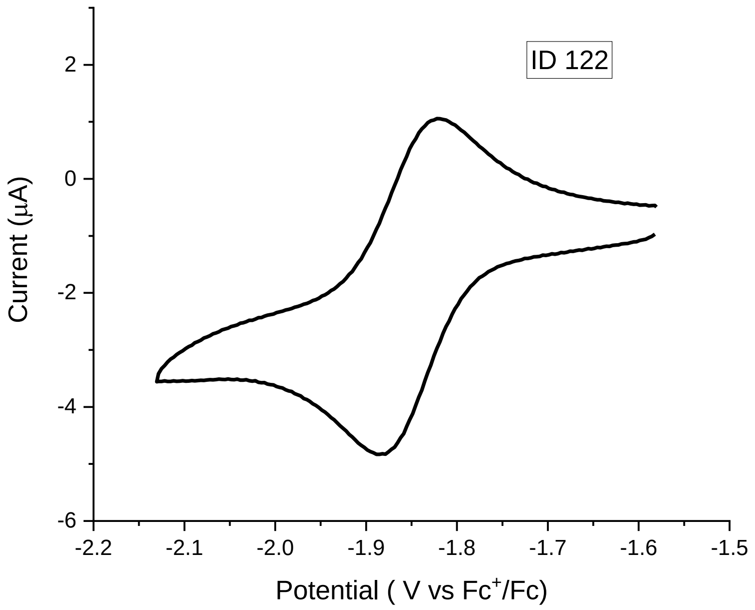

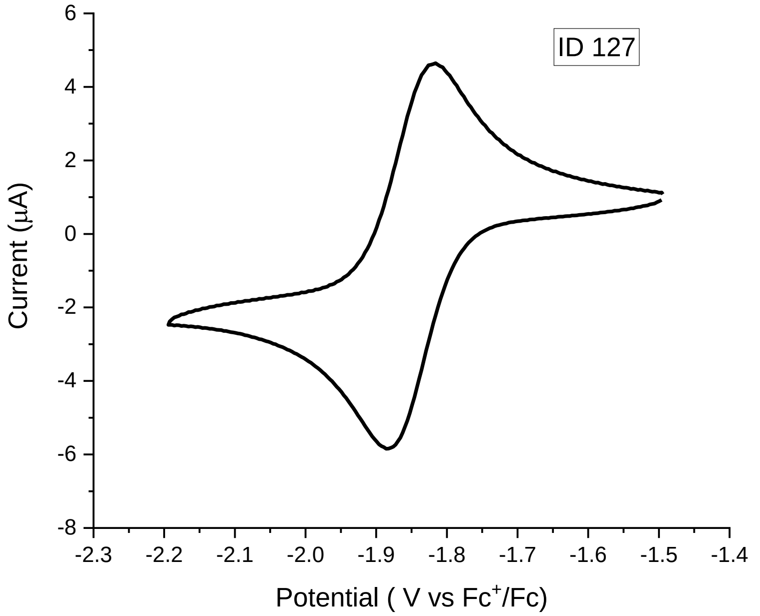

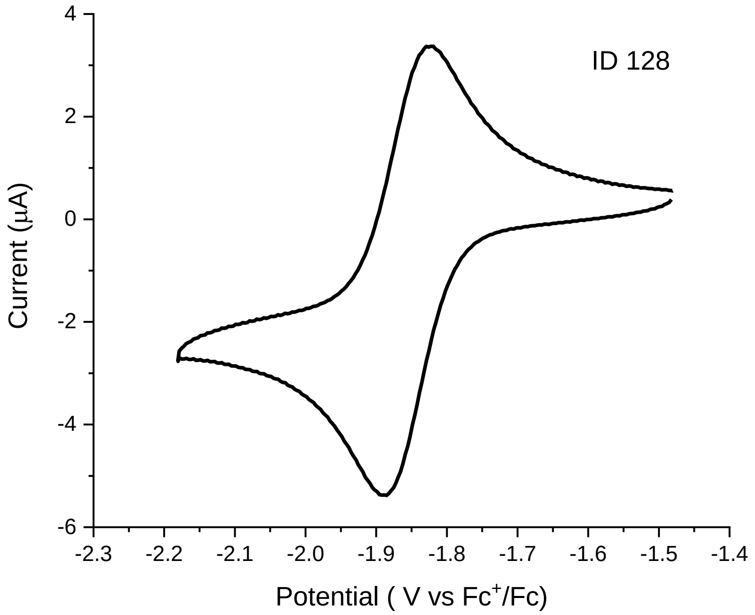
ID 128
Current (μA)
4
2
0
-2
-4
-6
-2.3
-2.2
-2.1
-2.0
-1.9
-1.8
-1.7
-1.6
-1.5
-1.4
Potential (V vs Fc+/Fc)

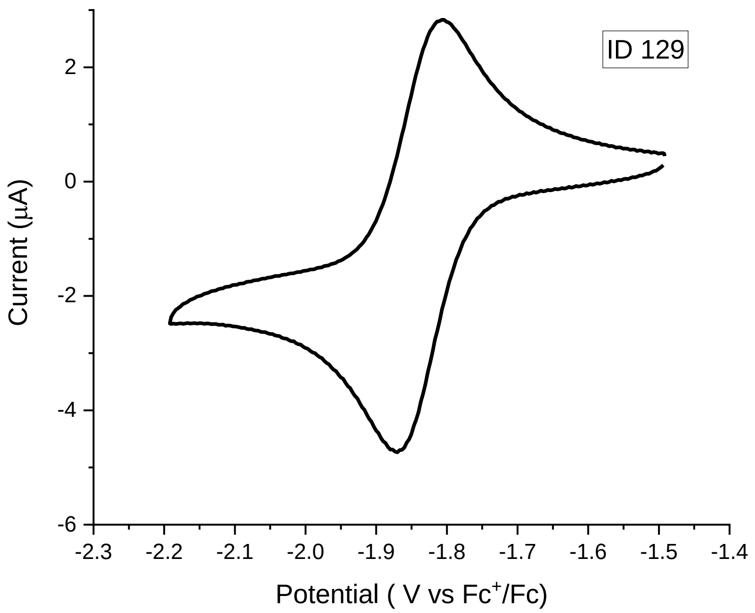
ID 129
Current (μA)
2
0
-2
-4
-6
-2.3
-2.2
-2.1
-2.0
-1.9
-1.8
-1.7
-1.6
-1.5
-1.4
Potential (V vs Fc+/Fc)

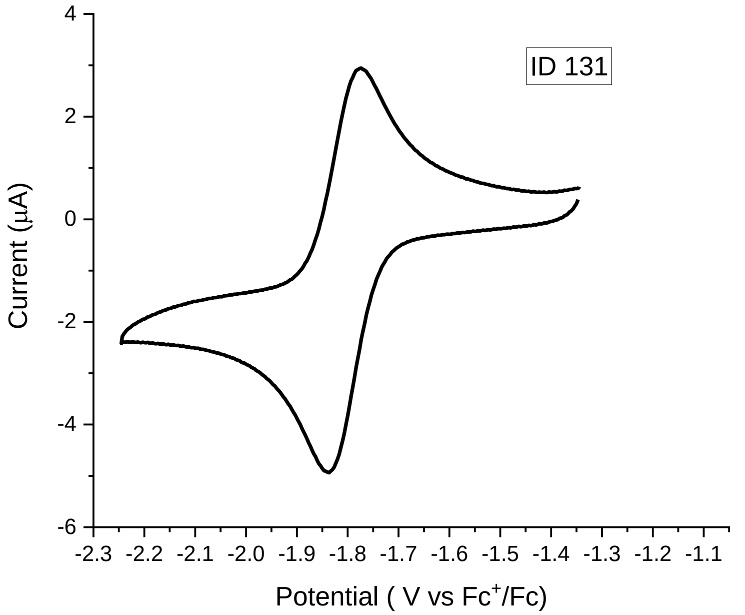
ID 131
Current (μA)
4
2
0
-2
-4
-6
-2.3
-2.2
-2.1
-2.0
-1.9
-1.8
-1.7
-1.6
-1.5
-1.4
-1.3
-1.2
-1.1
Potential (V vs Fc+/Fc)

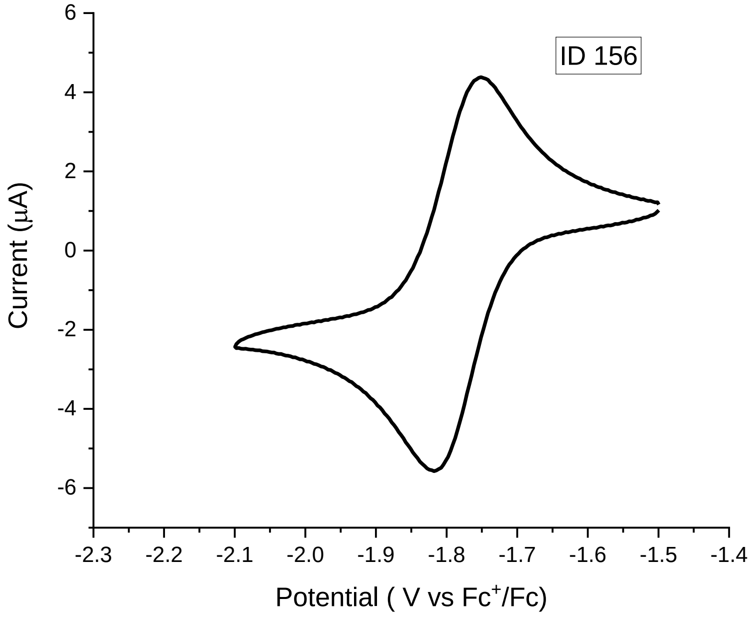
ID 156
Current (μA)
6
4
2
0
-2
-4
-6
-2.3
-2.2
-2.1
-2.0
-1.9
-1.8
-1.7
-1.6
-1.5
-1.4
Potential (V vs Fc+/Fc)

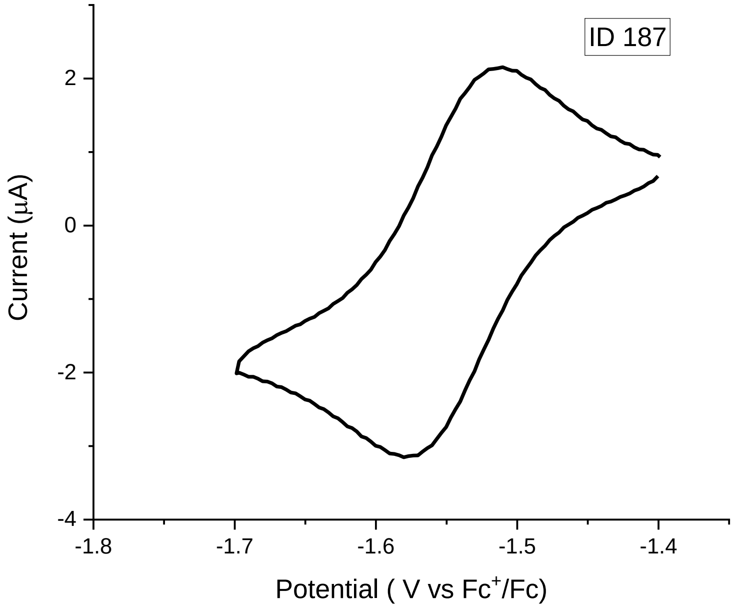
ID 187
Current (μA)
Potential (V vs Fc+/Fc)
2
0
-2
-4
-1.8
-1.7
-1.6
-1.5
-1.4

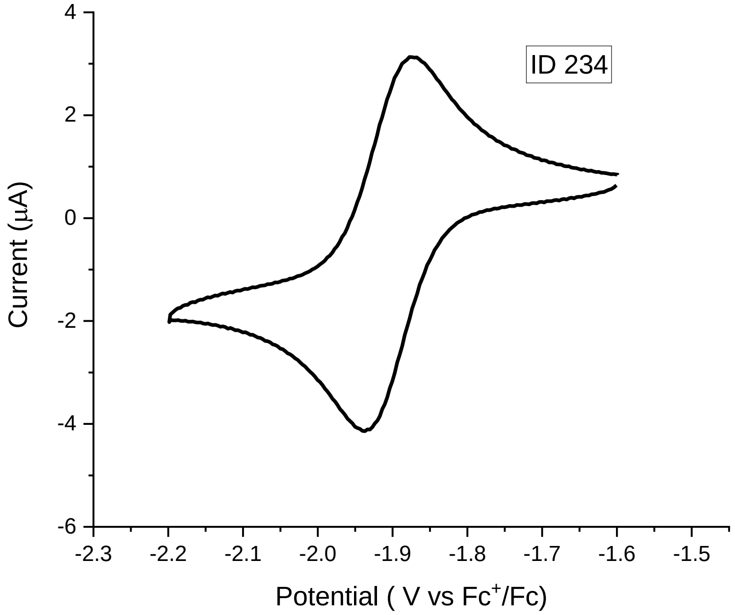
ID 234
Current (μA)
Potential (V vs Fc+/Fc)
4
2
0
-2
-4
-6
-2.3
-2.2
-2.1
-2.0
-1.9
-1.8
-1.7
-1.6
-1.5

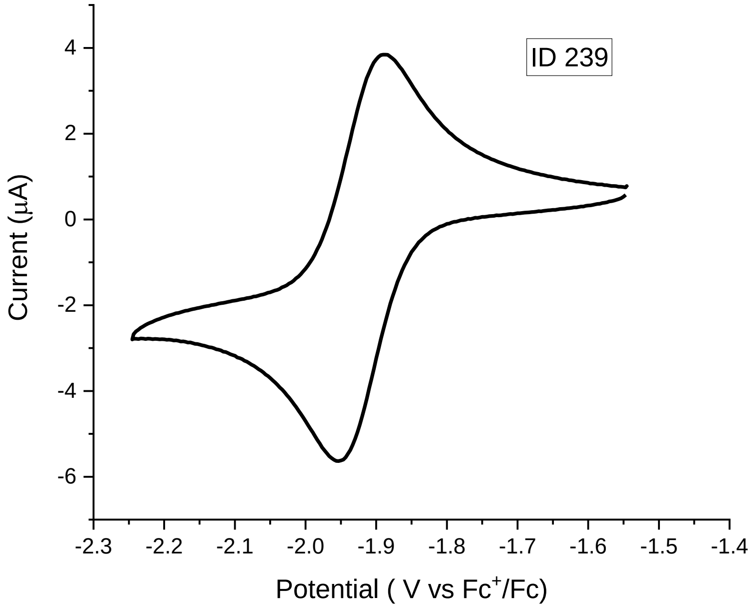
ID 239
Current (μA)
4
2
0
-2
-4
-6
-2.3
-2.2
-2.1
-2.0
-1.9
-1.8
-1.7
-1.6
-1.5
-1.4
Potential (V vs Fc+/Fc)

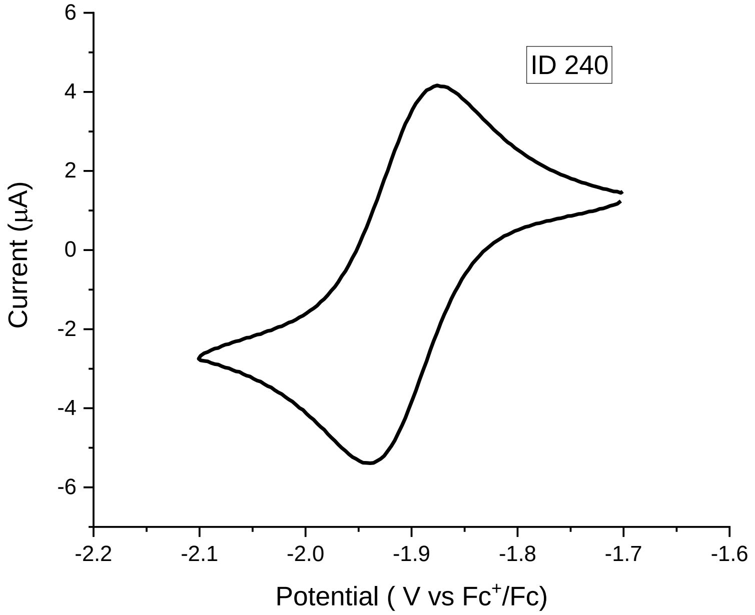
ID 240
Current (μA)
6
4
2
0
-2
-4
-6
-2.2
-2.1
-2.0
-1.9
-1.8
-1.7
-1.6
Potential (V vs Fc+/Fc)

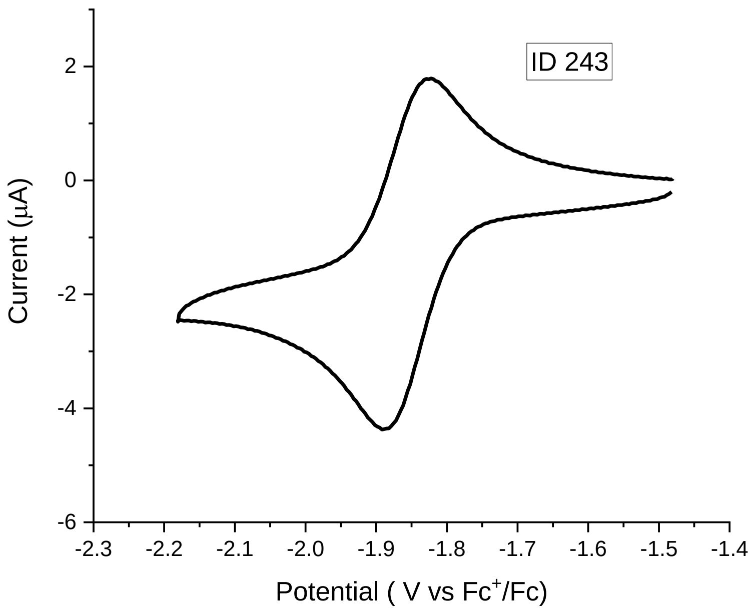

ID 243
Current (μA)
Potential (V vs Fc+/Fc)
2
0
-2
-4
-6
-2.3
-2.2
-2.1
-2.0
-1.9
-1.8
-1.7
-1.6
-1.5
-1.4

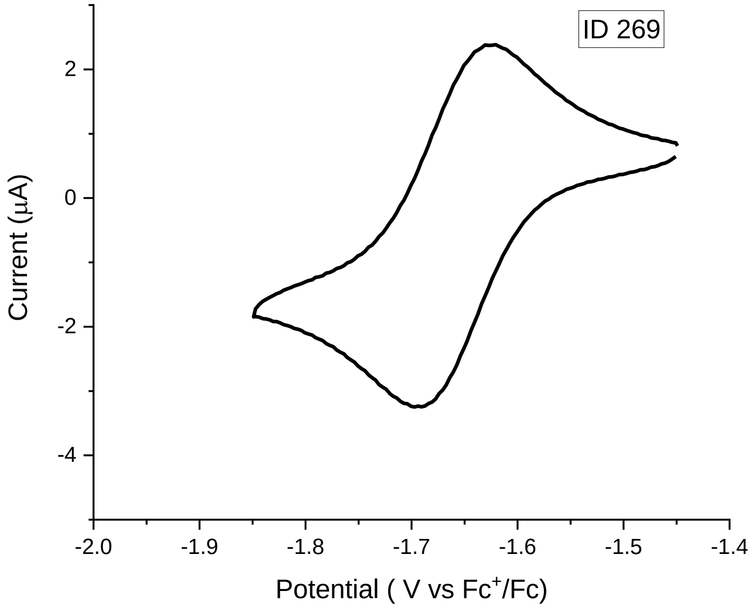

ID 269
Current (μA)
Potential (V vs Fc+/Fc)
2
0
-2
-4
-2.0
-1.9
-1.8
-1.7
-1.6
-1.5
-1.4

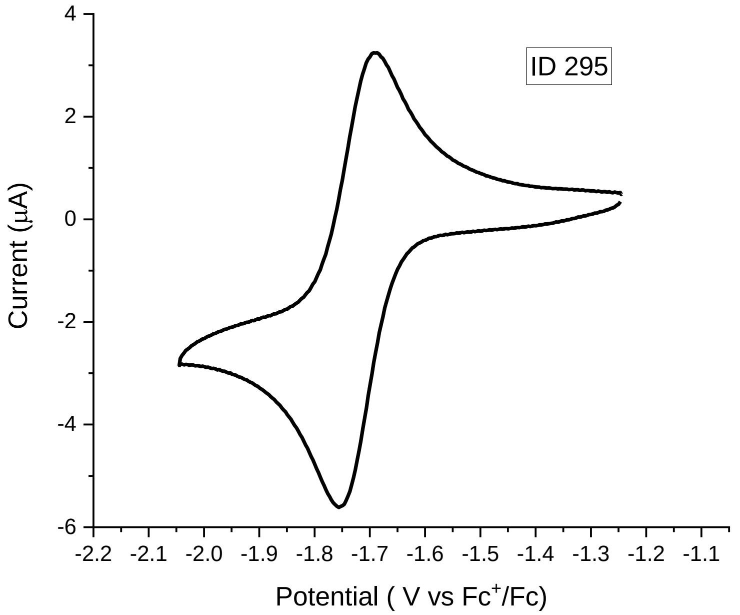
ID 295
Current (μA)
Potential (V vs Fc+/Fc)
4
2
0
-2
-4
-6
-2.2
-2.1
-2.0
-1.9
-1.8
-1.7
-1.6
-1.5
-1.4
-1.3
-1.2
-1.1

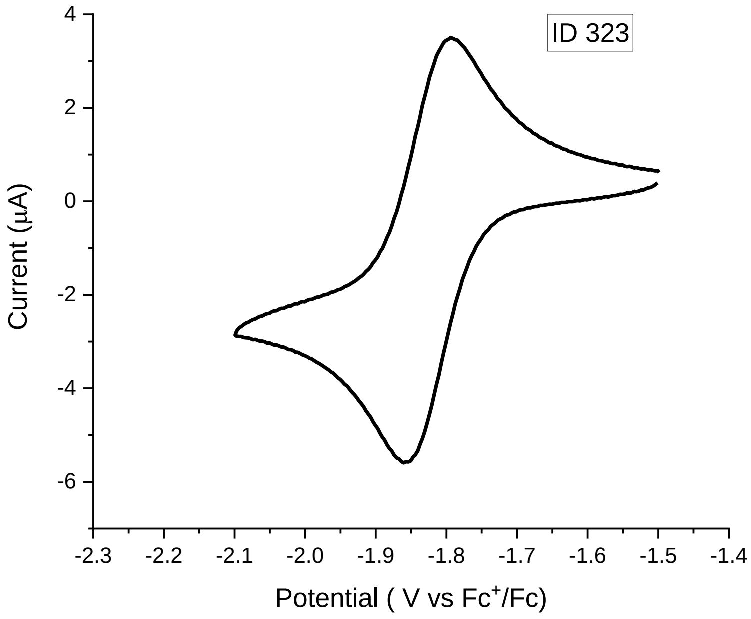
ID 323
Current (μA)
Potential (V vs Fc+/Fc)
4
2
0
-2
-4
-6
-2.3
-2.2
-2.1
-2.0
-1.9
-1.8
-1.7
-1.6
-1.5
-1.4

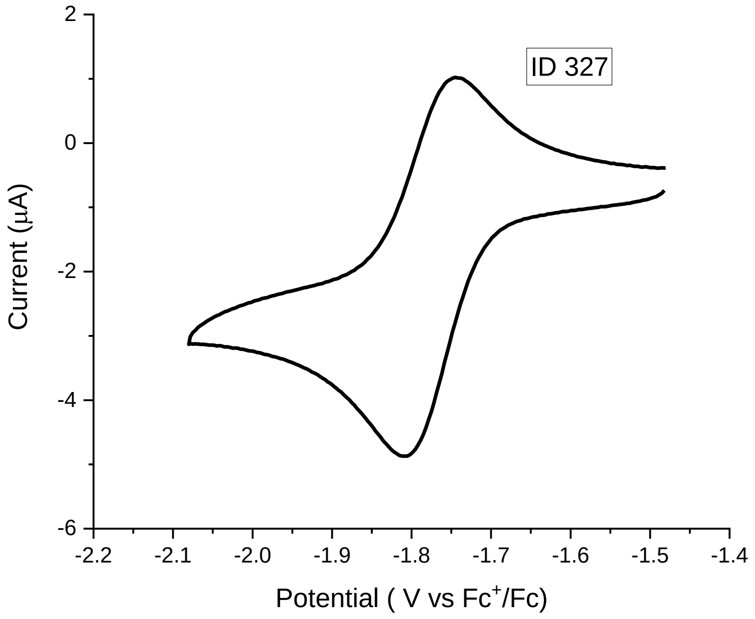

ID 327
Current (μA)
Potential (V vs Fc+/Fc)
2
0
-2
-4
-6
-2.2
-2.1
-2.0
-1.9
-1.8
-1.7
-1.6
-1.5
-1.4

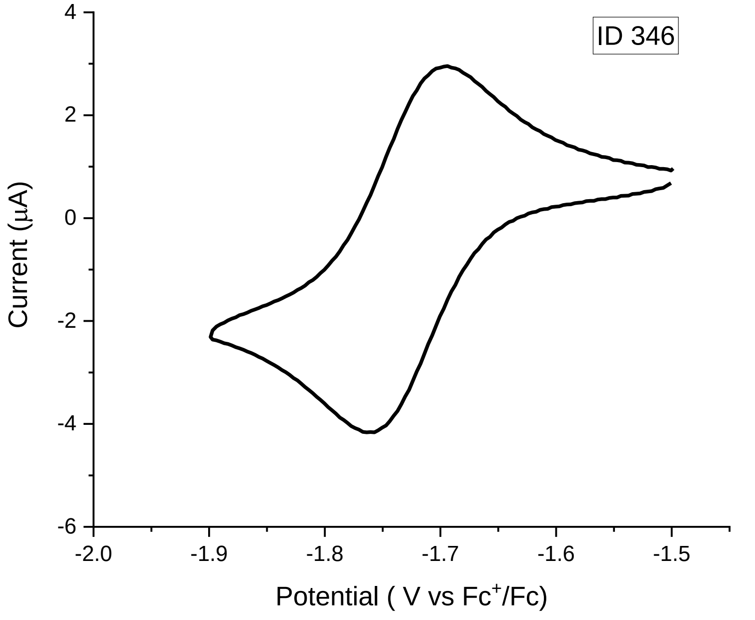

ID 346
Current (μA)
Potential (V vs Fc+/Fc)
4
2
0
-2
-4
-6
-2.0
-1.9
-1.8
-1.7
-1.6
-1.5

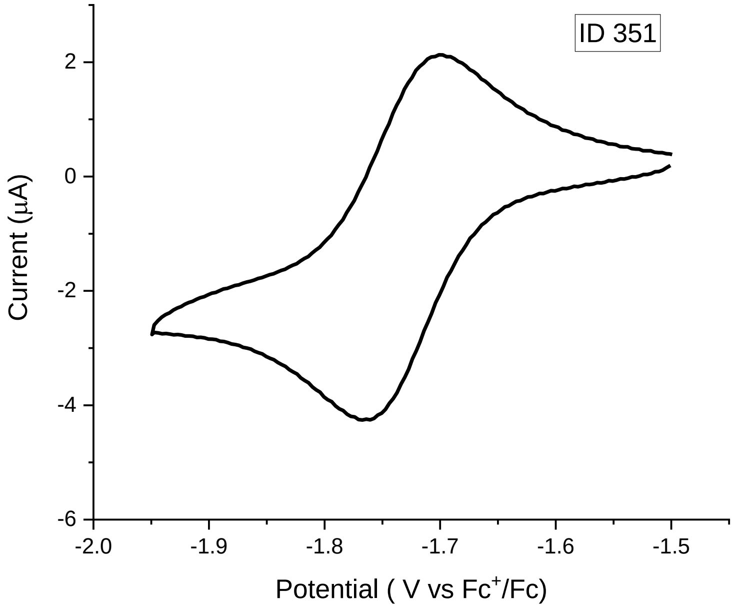
ID 351
Current (μA)
2
0
-2
-4
-6
-2.0
-1.9
-1.8
-1.7
-1.6
-1.5
Potential (V vs Fc+/Fc)

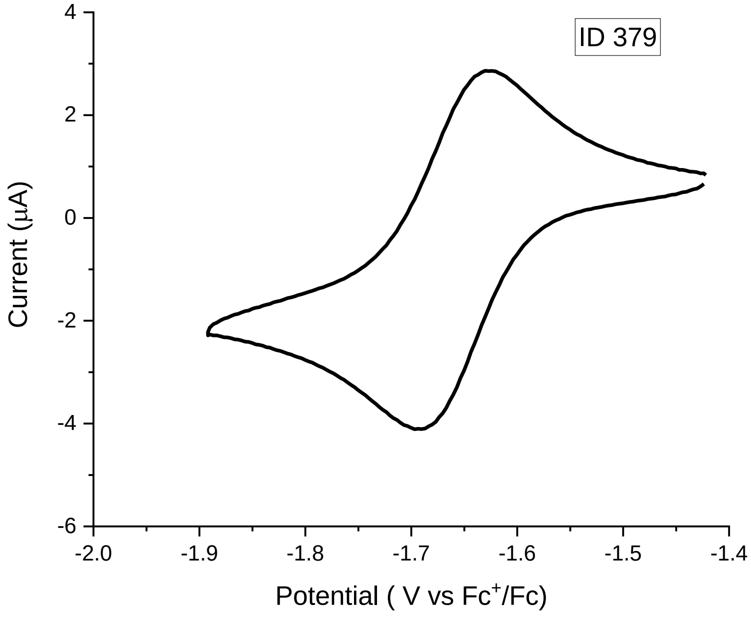
ID 379
Current (μA)
4
2
0
-2
-4
-6
-2.0
-1.9
-1.8
-1.7
-1.6
-1.5
-1.4
Potential (V vs Fc+/Fc)

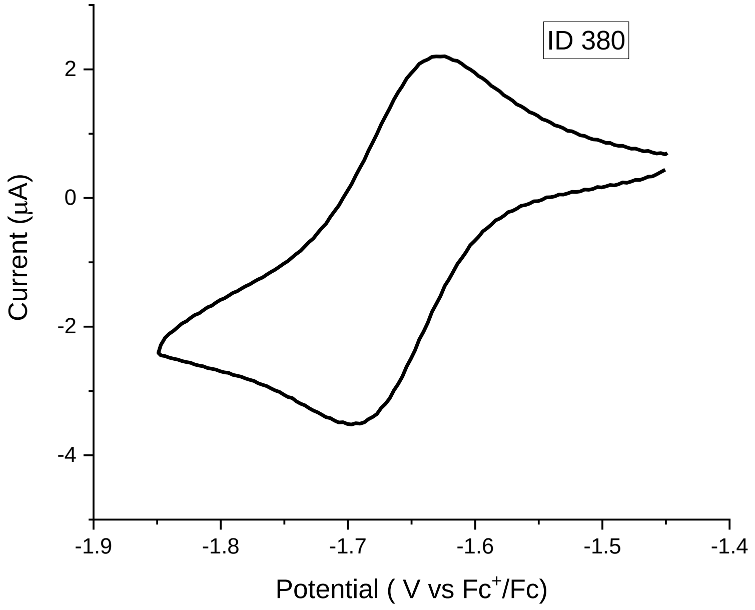
ID 380
Current (μA)
2
0
-2
-4
-1.9
-1.8
-1.7
-1.6
-1.5
-1.4
Potential (V vs Fc+/Fc)

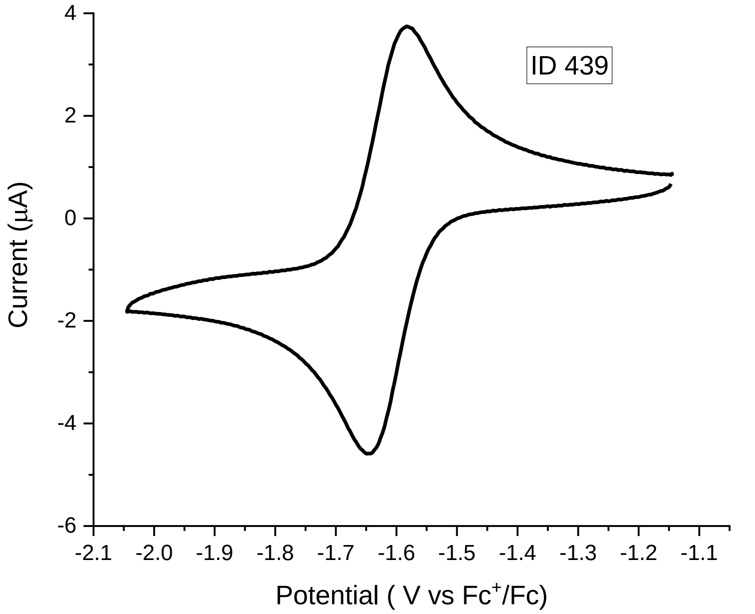
ID 439
Current (μA)
4
2
0
-2
-4
-6
-2.1
-2.0
-1.9
-1.8
-1.7
-1.6
-1.5
-1.4
-1.3
-1.2
-1.1
Potential (V vs Fc+/Fc)

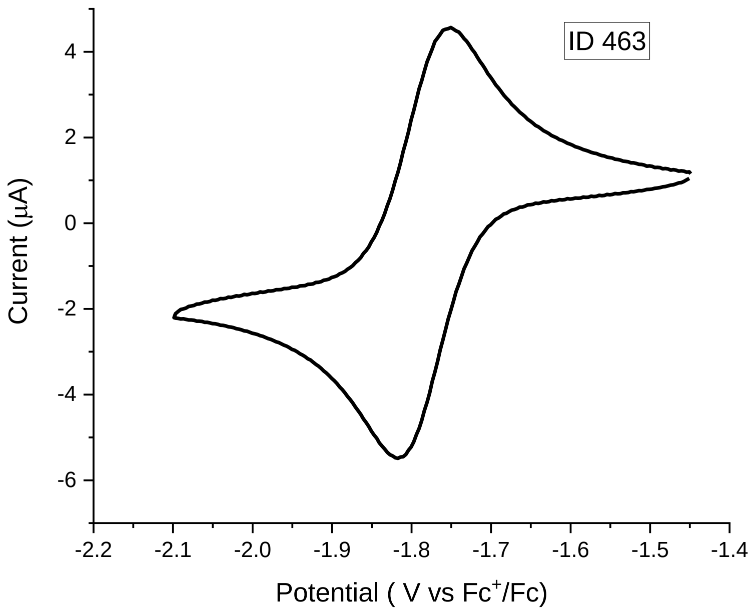
ID 463
Current (μA)
4
2
0
-2
-4
-6
-2.2
-2.1
-2.0
-1.9
-1.8
-1.7
-1.6
-1.5
-1.4
Potential (V vs Fc+/Fc)

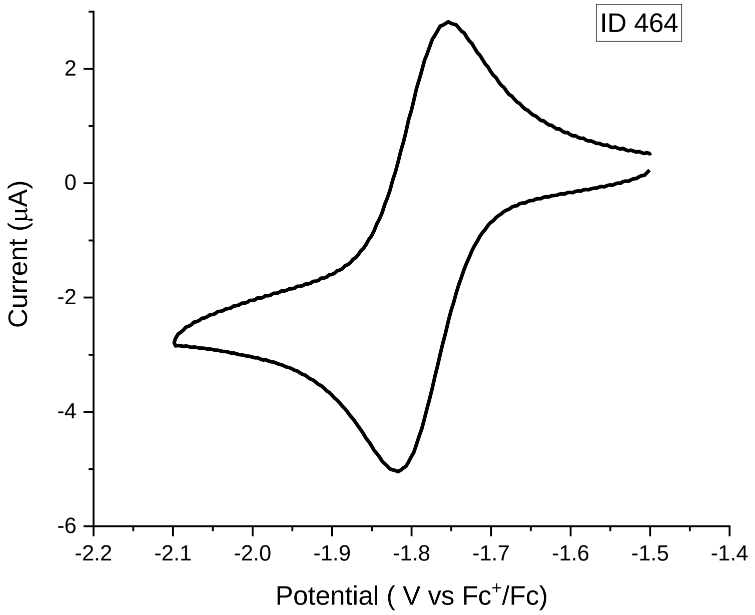
ID 464
Current (μA)
2
0
-2
-4
-6
-2.2
-2.1
-2.0
-1.9
-1.8
-1.7
-1.6
-1.5
-1.4
Potential (V vs Fc+/Fc)

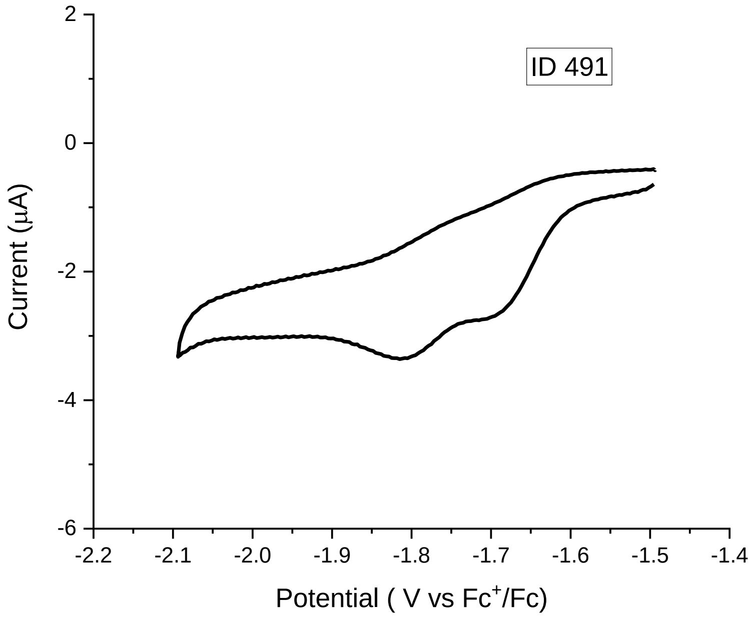

ID 491
Current (μA)
Potential (V vs Fc+/Fc)
2
0
-2
-4
-6
-2.2
-2.1
-2.0
-1.9
-1.8
-1.7
-1.6
-1.5
-1.4

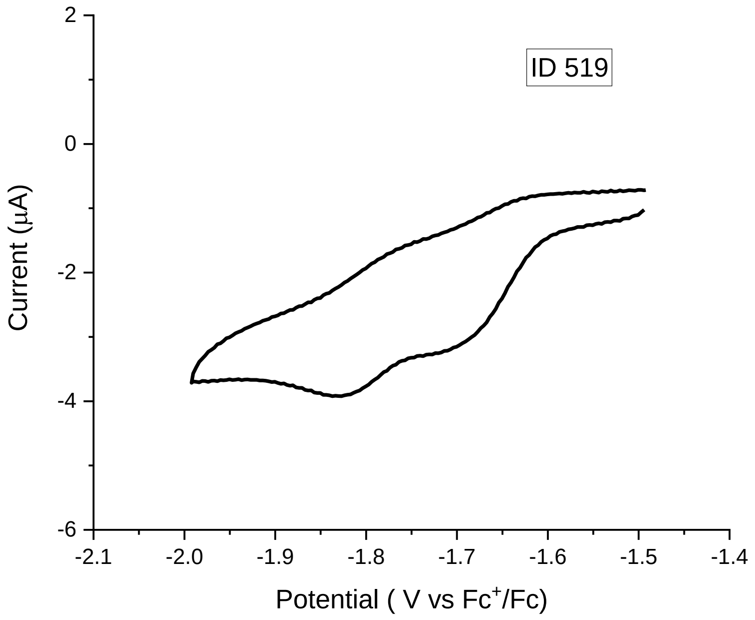

ID 519
Current (μA)
Potential (V vs Fc+/Fc)
2
0
-2
-4
-6
-2.1
-2.0
-1.9
-1.8
-1.7
-1.6
-1.5
-1.4

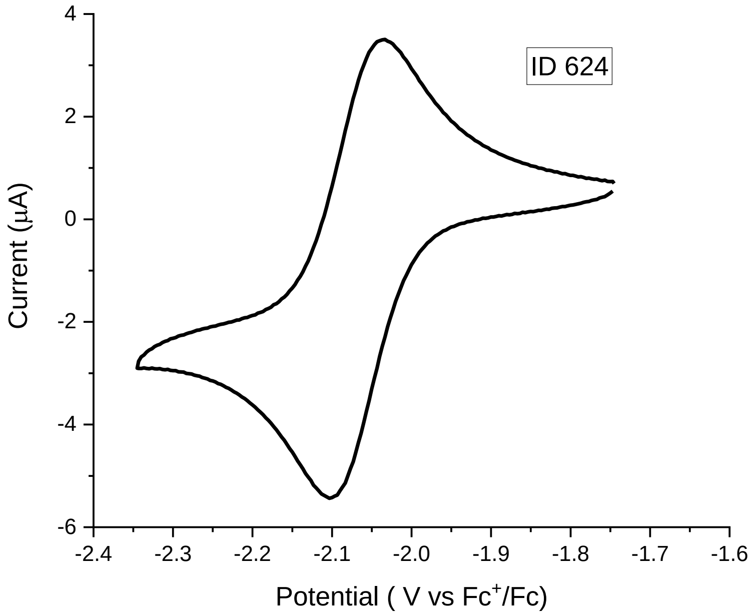
ID 624
Current (μA)
Potential (V vs Fc+/Fc)
4
2
0
-2
-4
-6
-2.4
-2.3
-2.2
-2.1
-2.0
-1.9
-1.8
-1.7
-1.6

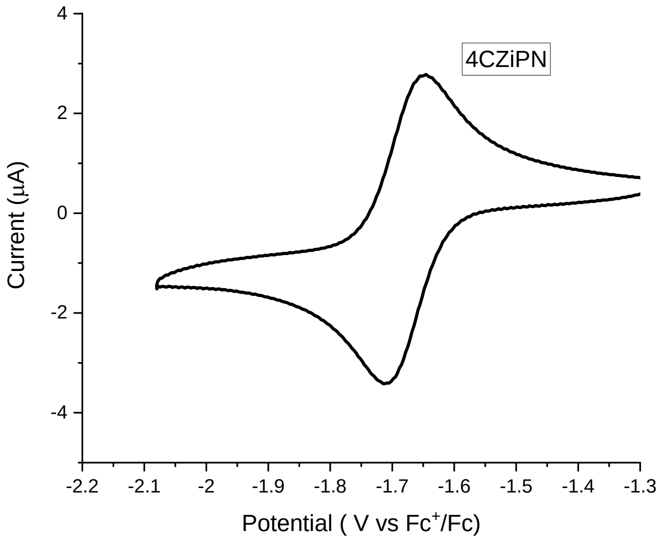
4CZiPN
Current (μA)
Potential (V vs Fc+/Fc)
4
2
0
-2
-4
-2.2
-2.1
-2
-1.9
-1.8
-1.7
-1.6
-1.5
-1.4
-1.3

UV-vis absorption spectra of CNPs

Condition: 20 μM in DMF.

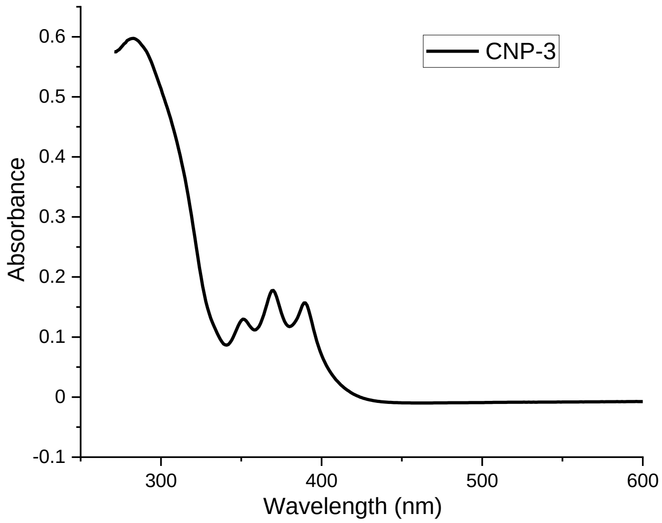

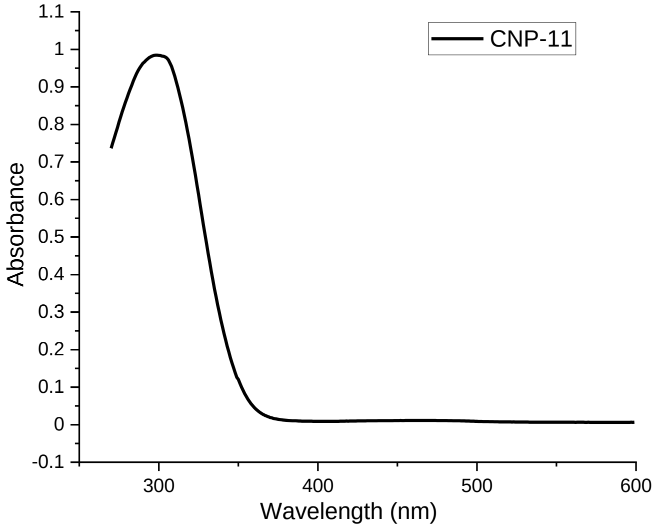

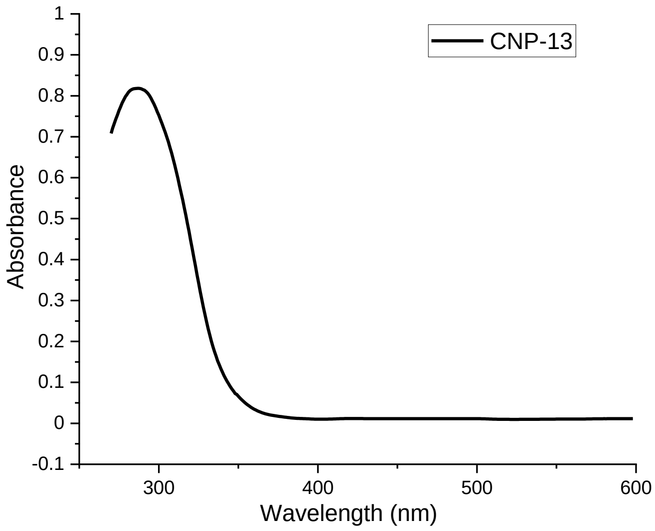
CNP-13
Absorbance
Wavelength (nm)
1
0.9
0.8
0.7
0.6
0.5
0.4
0.3
0.2
0.1
0
-0.1
300
400
500
600

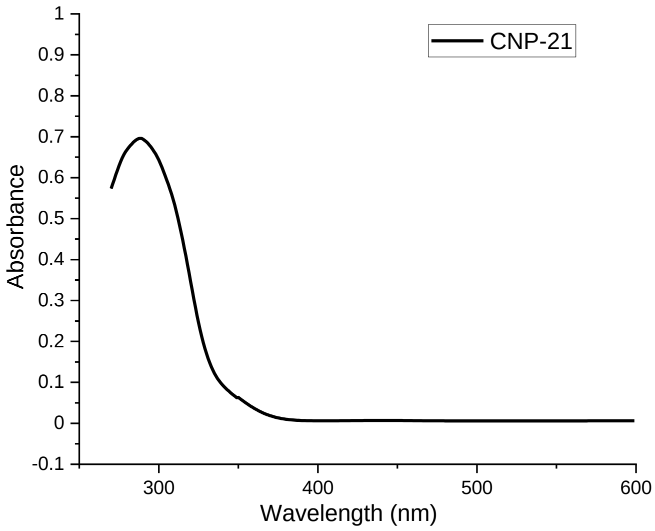
CNP-21
Absorbance
Wavelength (nm)
1
0.9
0.8
0.7
0.6
0.5
0.4
0.3
0.2
0.1
0
-0.1
300
400
500
600

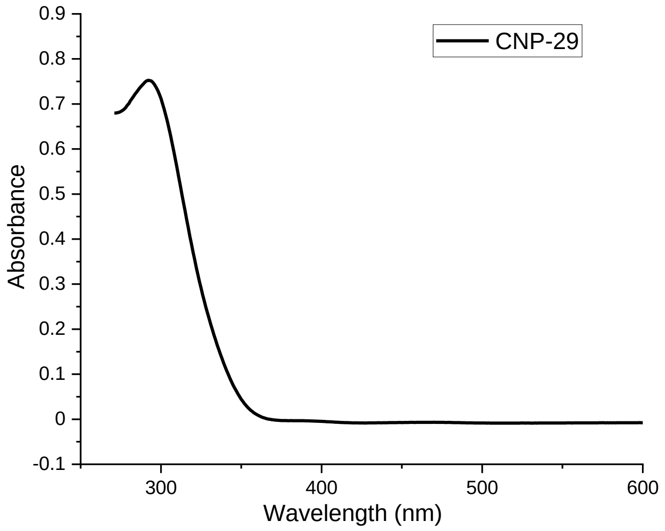
CNP-29
Absorbance
Wavelength (nm)
0.9
0.8
0.7
0.6
0.5
0.4
0.3
0.2
0.1
0
-0.1
300
400
500
600

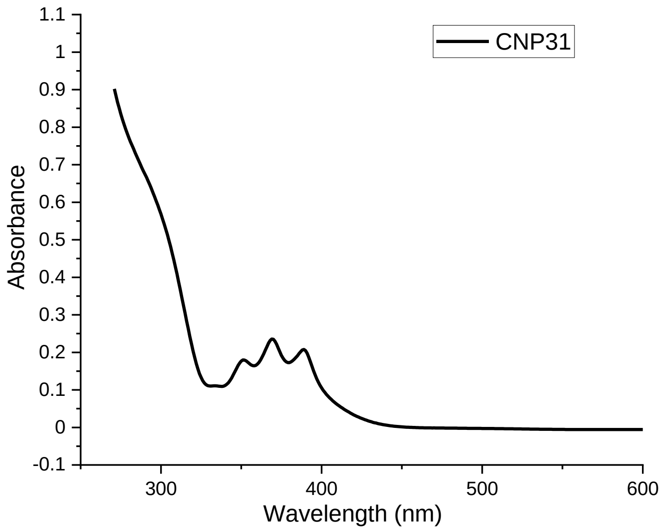
CNP31
Absorbance
Wavelength (nm)
1.1
1
0.9
0.8
0.7
0.6
0.5
0.4
0.3
0.2
0.1
0
-0.1
300
400
500
600

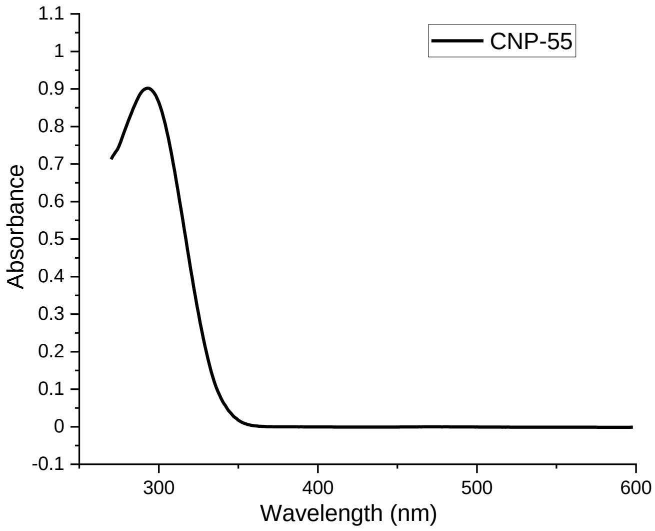
CNP-55
Absorbance
Wavelength (nm)
1.1
1
0.9
0.8
0.7
0.6
0.5
0.4
0.3
0.2
0.1
0
-0.1
300
400
500
600

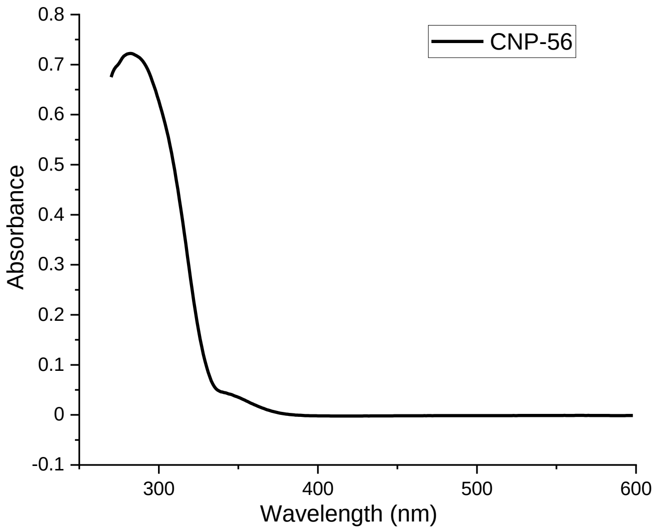
CNP-56
Absorbance
Wavelength (nm)
0.8
0.7
0.6
0.5
0.4
0.3
0.2
0.1
0
-0.1
300
400
500
600

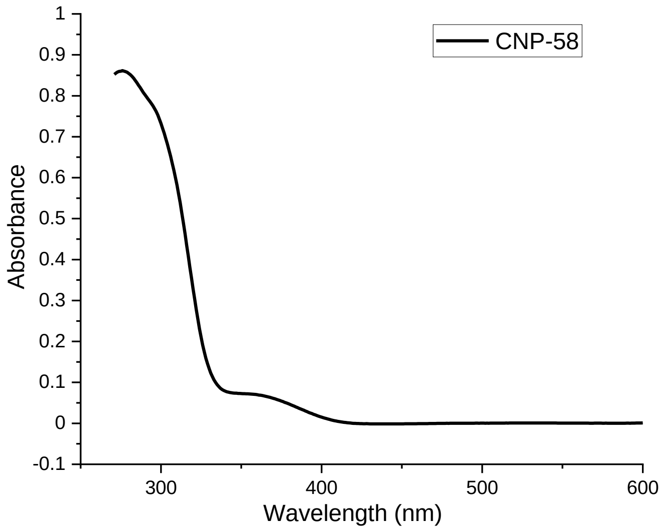
CNP-58
Absorbance
Wavelength (nm)
1
0.9
0.8
0.7
0.6
0.5
0.4
0.3
0.2
0.1
0
-0.1
300
400
500
600

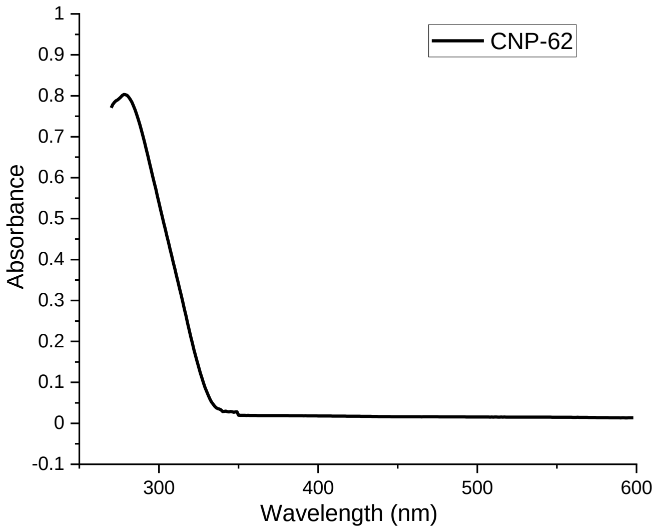
CNP-62
Absorbance
Wavelength (nm)
1
0.9
0.8
0.7
0.6
0.5
0.4
0.3
0.2
0.1
0
-0.1
300
400
500
600

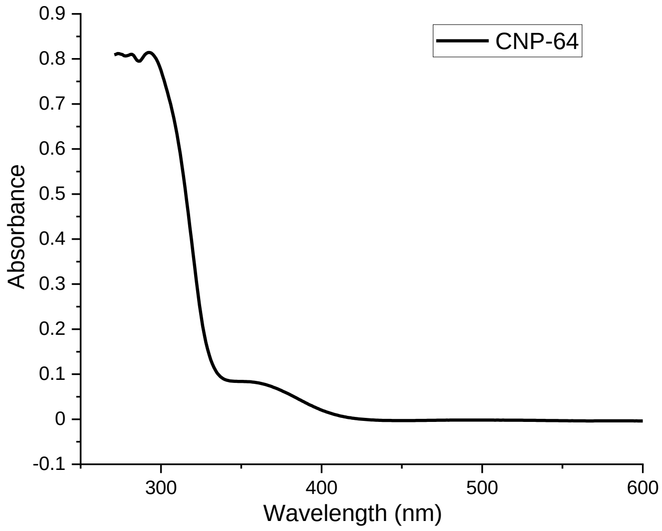

CNP-64
Absorbance
Wavelength (nm)
0.9
0.8
0.7
0.6
0.5
0.4
0.3
0.2
0.1
0
-0.1
300
400
500
600

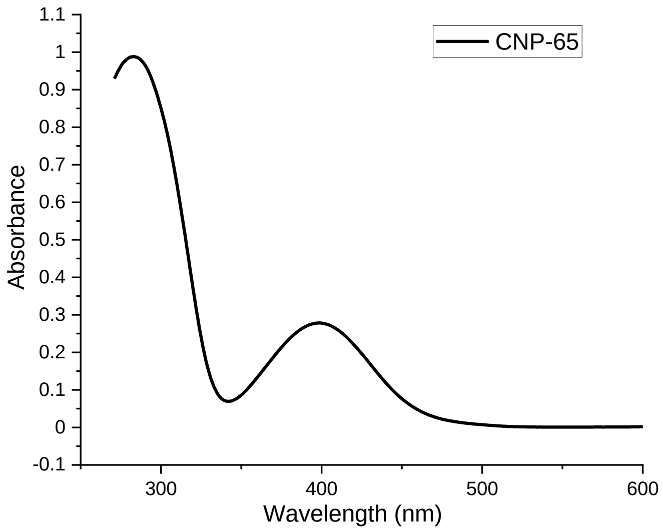

CNP-65
Absorbance
Wavelength (nm)
1.1
1
0.9
0.8
0.7
0.6
0.5
0.4
0.3
0.2
0.1
0
-0.1
300
400
500
600

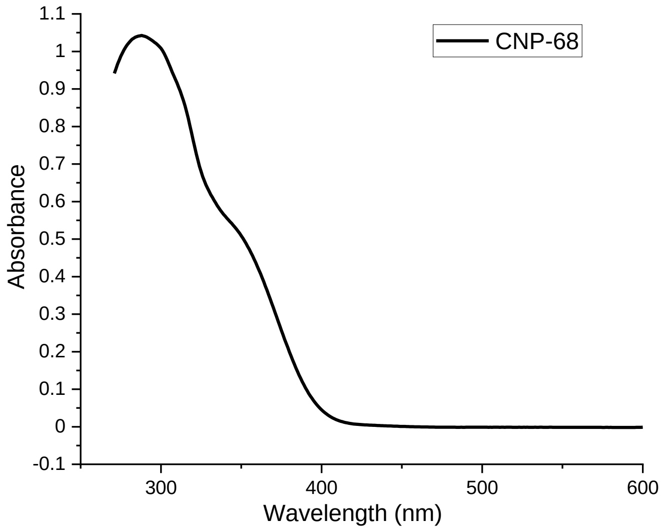
CNP-68
Absorbance
Wavelength (nm)
1.1
1
0.9
0.8
0.7
0.6
0.5
0.4
0.3
0.2
0.1
0
-0.1
300
400
500
600

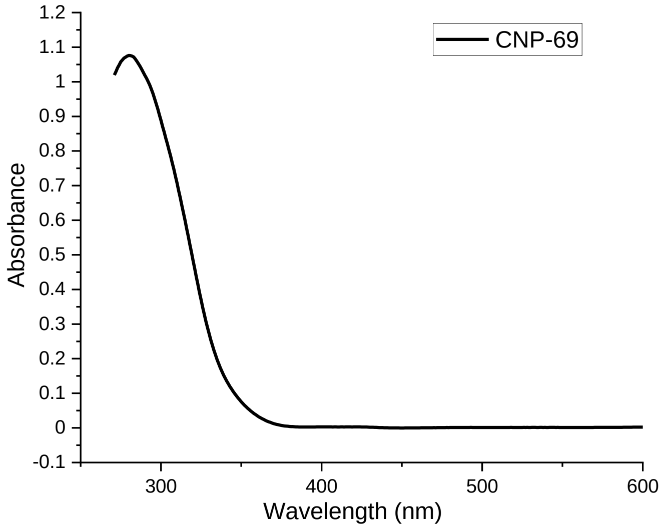
CNP-69
Absorbance
Wavelength (nm)
1.2
1.1
1
0.9
0.8
0.7
0.6
0.5
0.4
0.3
0.2
0.1
0
-0.1
300
400
500
600

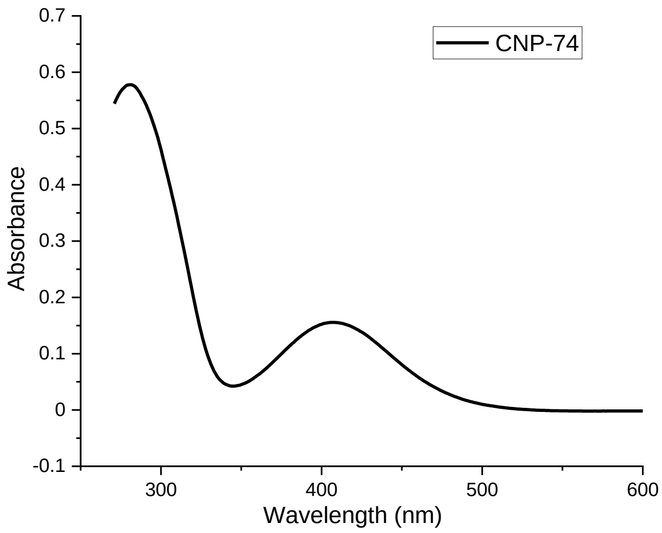
CNP-74
Absorbance
Wavelength (nm)
0.7
0.6
0.5
0.4
0.3
0.2
0.1
0
-0.1
300
400
500
600

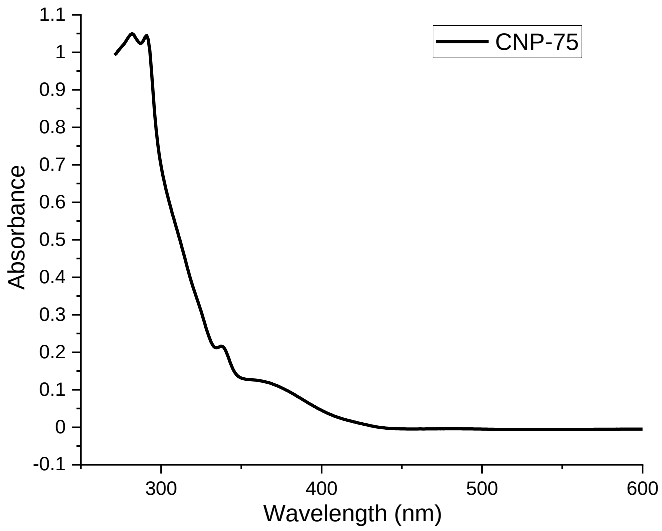
CNP-75
Absorbance
Wavelength (nm)
1.1
1
0.9
0.8
0.7
0.6
0.5
0.4
0.3
0.2
0.1
0
-0.1
300
400
500
600

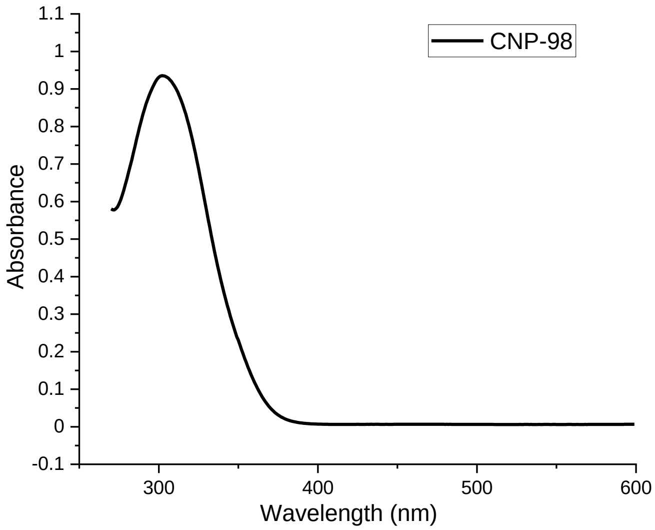
CNP-98
Absorbance
Wavelength (nm)
1.1
1
0.9
0.8
0.7
0.6
0.5
0.4
0.3
0.2
0.1
0
-0.1
300
400
500
600

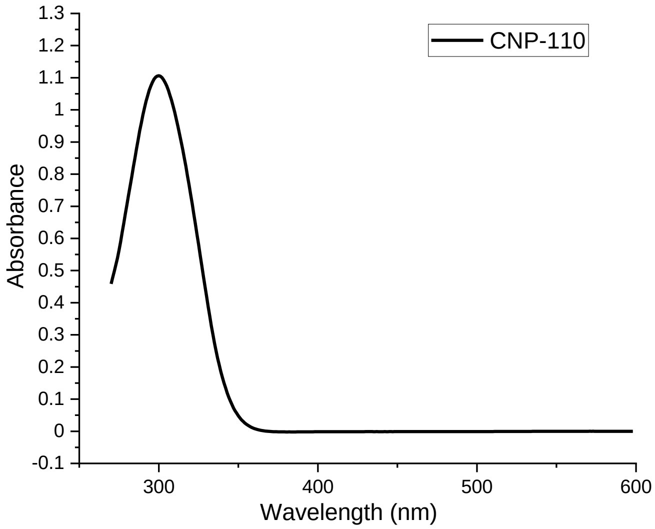
CNP-110
Absorbance
Wavelength (nm)
1.3
1.2
1.1
1
0.9
0.8
0.7
0.6
0.5
0.4
0.3
0.2
0.1
0
-0.1
300
400
500
600

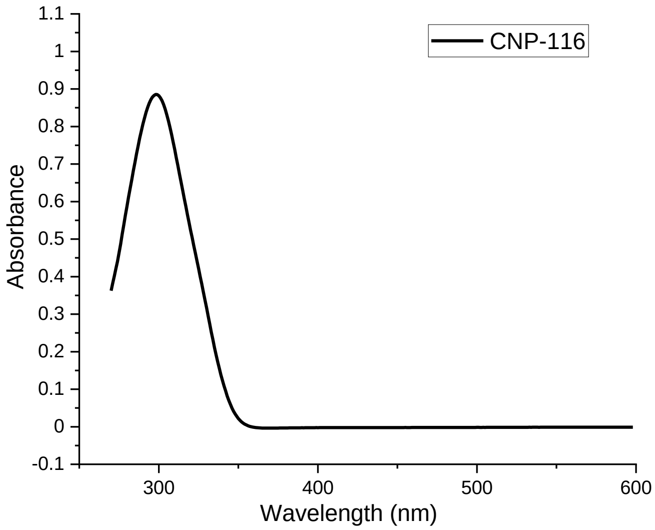
CNP-116
Absorbance
Wavelength (nm)
1.1
1
0.9
0.8
0.7
0.6
0.5
0.4
0.3
0.2
0.1
0
-0.1
300
400
500
600

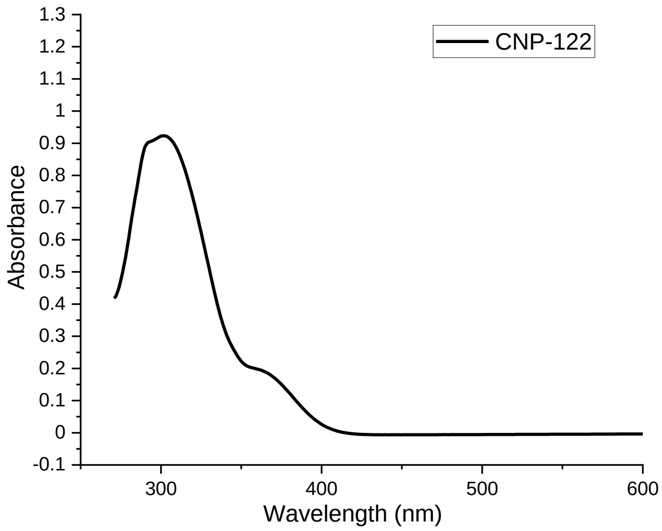
CNP-122
Absorbance
Wavelength (nm)
1.3
1.2
1.1
1
0.9
0.8
0.7
0.6
0.5
0.4
0.3
0.2
0.1
0
-0.1
300
400
500
600

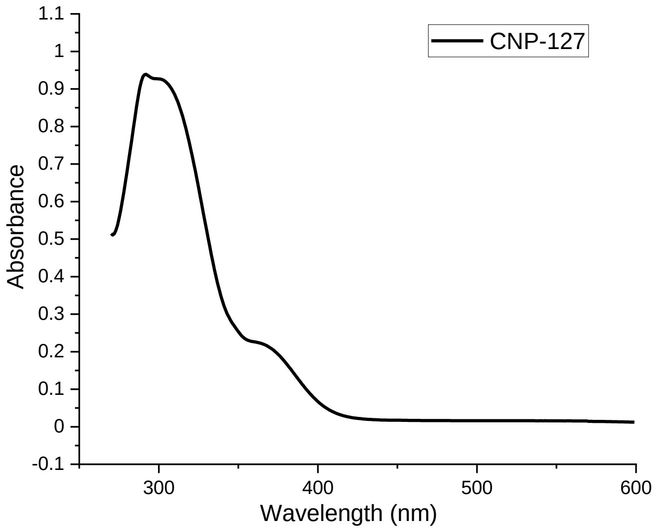

CNP-127
Absorbance
Wavelength (nm)
1.1
1
0.9
0.8
0.7
0.6
0.5
0.4
0.3
0.2
0.1
0
-0.1
300
400
500
600

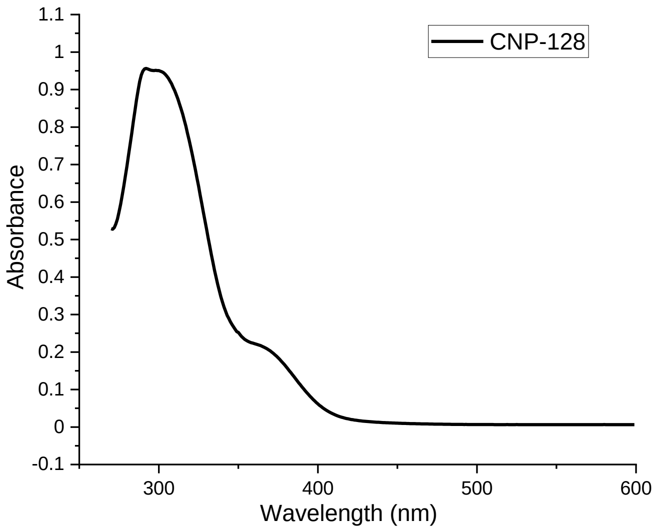

CNP-128
Absorbance
Wavelength (nm)
1.1
1
0.9
0.8
0.7
0.6
0.5
0.4
0.3
0.2
0.1
0
-0.1
300
400
500
600

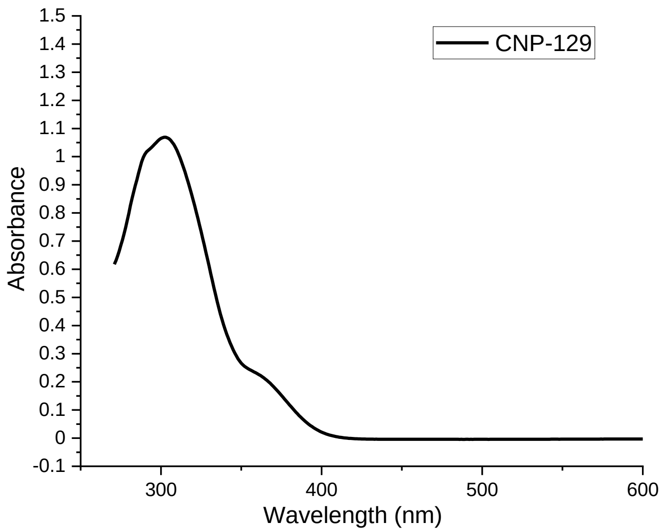
CNP-129
Absorbance
Wavelength (nm)
1.5
1.4
1.3
1.2
1.1
1
0.9
0.8
0.7
0.6
0.5
0.4
0.3
0.2
0.1
0
-0.1
300
400
500
600

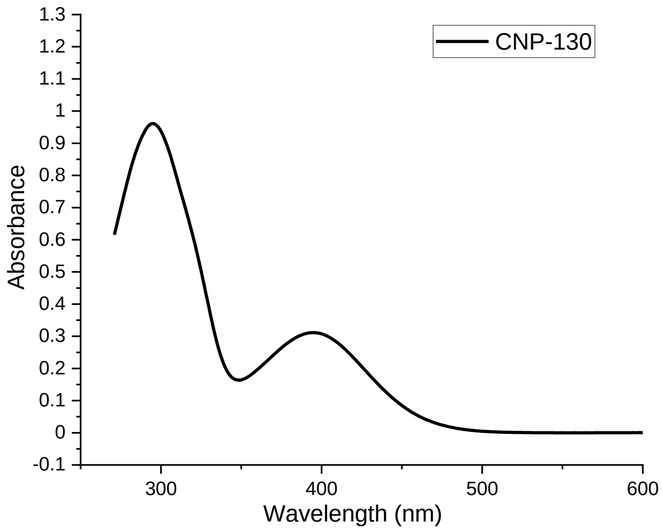
CNP-130
Absorbance
Wavelength (nm)
1.3
1.2
1.1
1
0.9
0.8
0.7
0.6
0.5
0.4
0.3
0.2
0.1
0
-0.1
300
400
500
600

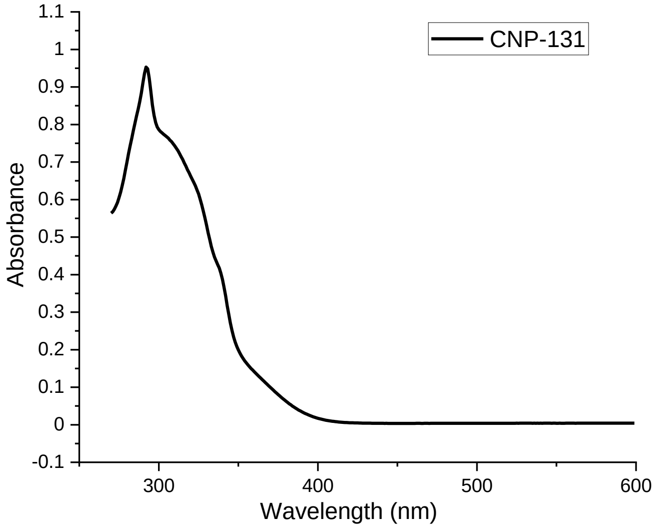

CNP-131
Absorbance
Wavelength (nm)
1.1
1
0.9
0.8
0.7
0.6
0.5
0.4
0.3
0.2
0.1
0
-0.1
300
400
500
600

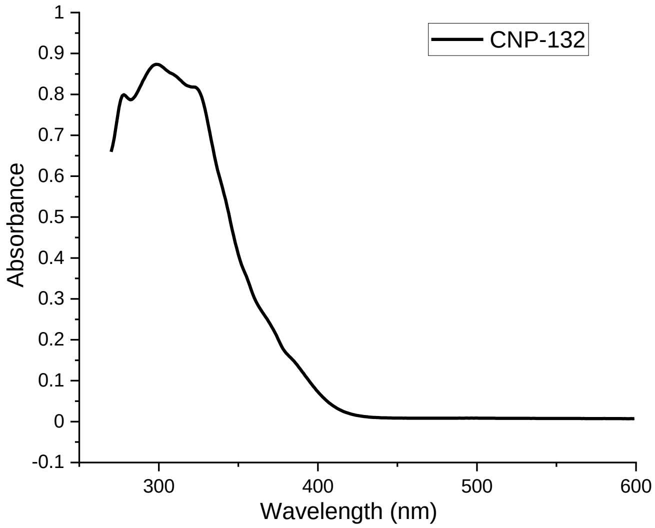

CNP-132
Absorbance
Wavelength (nm)
1
0.9
0.8
0.7
0.6
0.5
0.4
0.3
0.2
0.1
0
-0.1
300
400
500
600

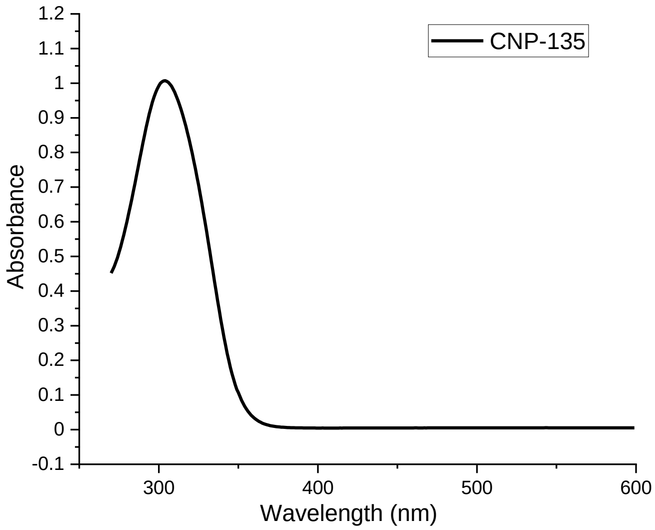
CNP-135
Absorbance
Wavelength (nm)
1.2
1.1
1
0.9
0.8
0.7
0.6
0.5
0.4
0.3
0.2
0.1
0
-0.1
300
400
500
600

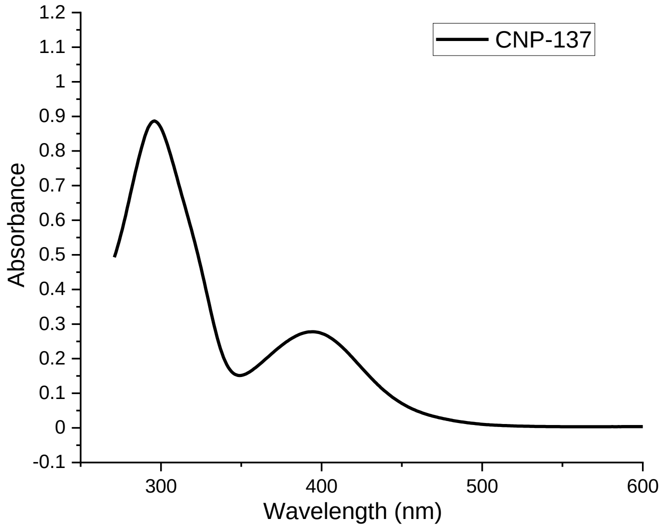
CNP-137
Absorbance
Wavelength (nm)
1.2
1.1
1
0.9
0.8
0.7
0.6
0.5
0.4
0.3
0.2
0.1
0
-0.1
300
400
500
600

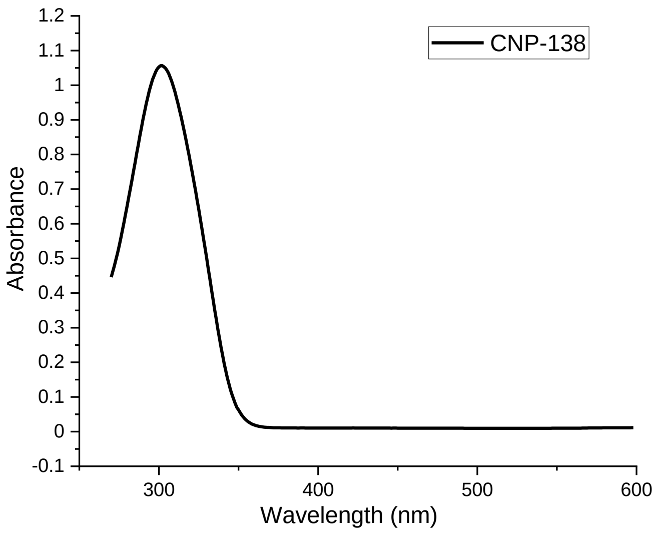

CNP-138
Absorbance
Wavelength (nm)
1.2
1.1
1
0.9
0.8
0.7
0.6
0.5
0.4
0.3
0.2
0.1
0
-0.1
300
400
500
600

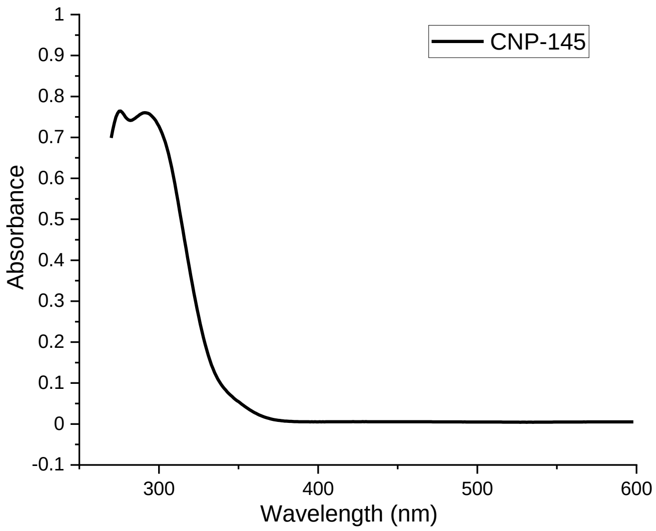

CNP-145
Absorbance
Wavelength (nm)
1
0.9
0.8
0.7
0.6
0.5
0.4
0.3
0.2
0.1
0
-0.1
300
400
500
600

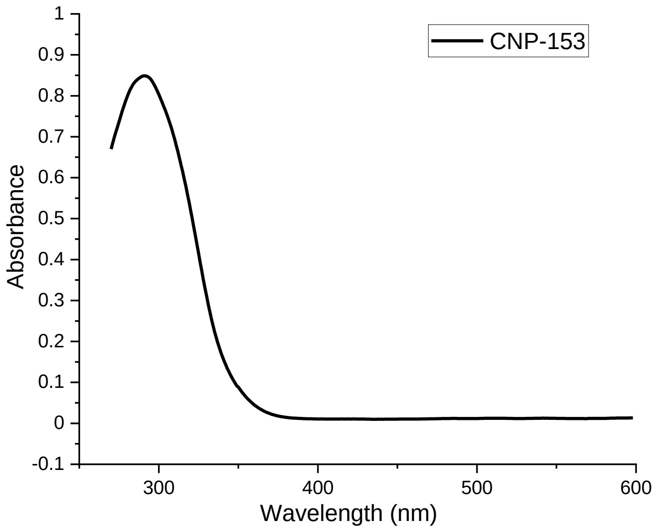
CNP-153
Absorbance
Wavelength (nm)
1
0.9
0.8
0.7
0.6
0.5
0.4
0.3
0.2
0.1
0
-0.1
300
400
500
600

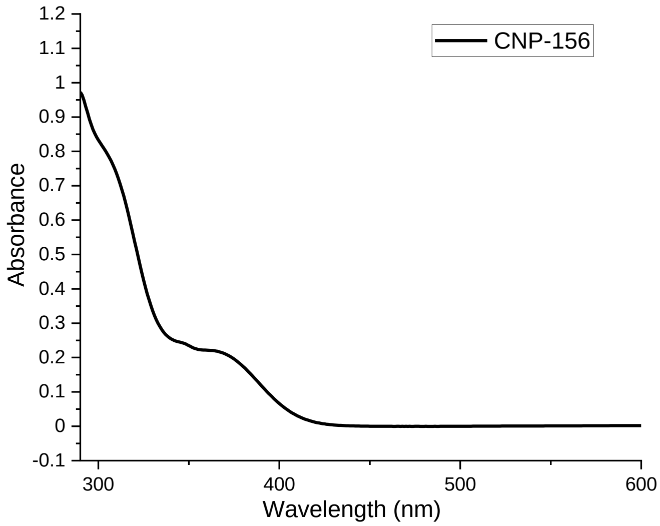
CNP-156
Absorbance
Wavelength (nm)
1.2
1.1
1
0.9
0.8
0.7
0.6
0.5
0.4
0.3
0.2
0.1
0
-0.1
300
400
500
600

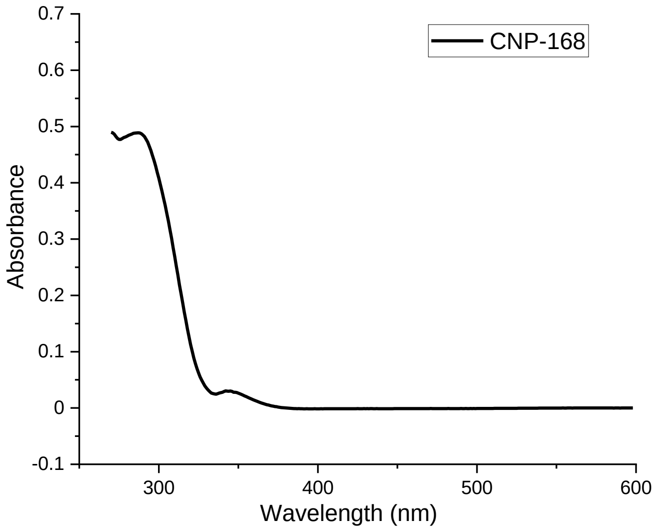
CNP-168
Absorbance
Wavelength (nm)
0.7
0.6
0.5
0.4
0.3
0.2
0.1
0
-0.1
300
400
500
600

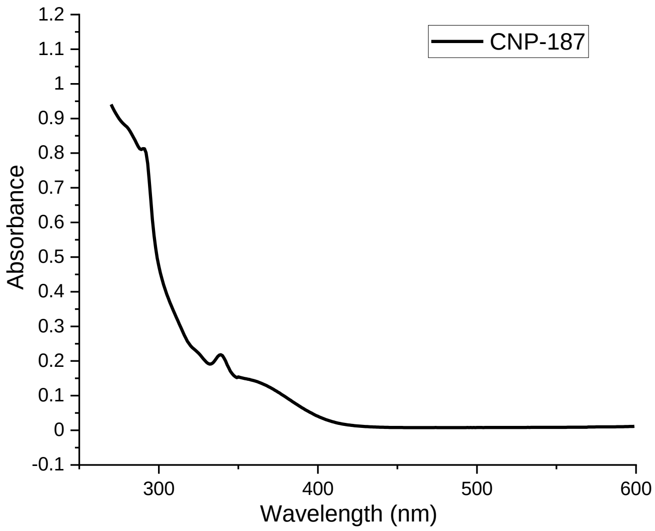
CNP-187
Absorbance
Wavelength (nm)
1.2
1.1
1
0.9
0.8
0.7
0.6
0.5
0.4
0.3
0.2
0.1
0
-0.1
300
400
500
600

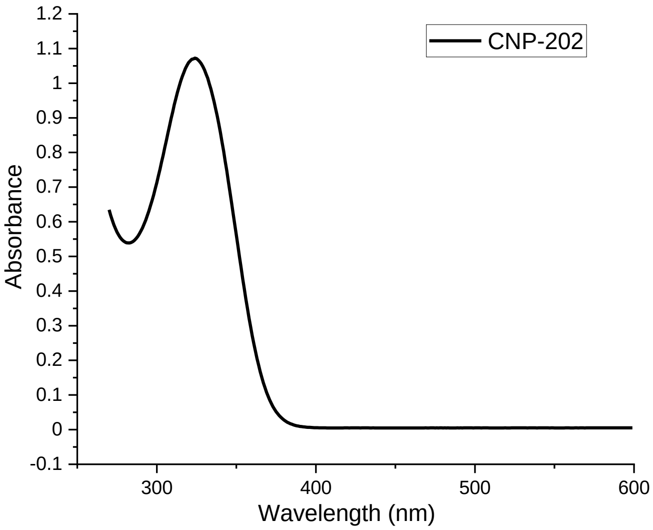

CNP-202
Absorbance
Wavelength (nm)
1.2
1.1
1
0.9
0.8
0.7
0.6
0.5
0.4
0.3
0.2
0.1
0
-0.1
300
400
500
600

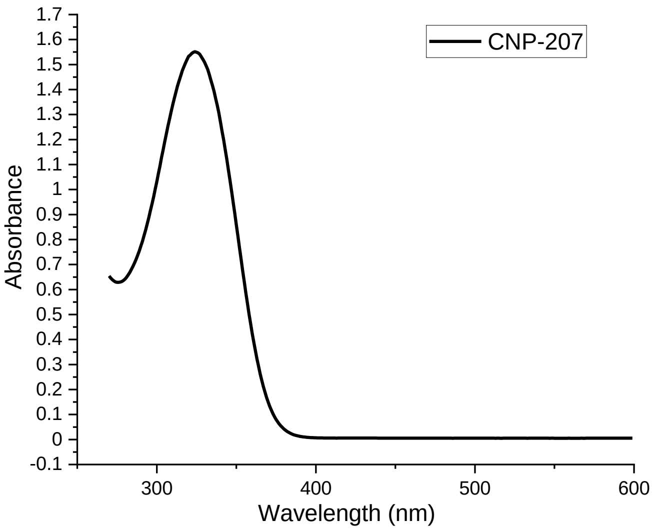

CNP-207
Absorbance
Wavelength (nm)
1.7
1.6
1.5
1.4
1.3
1.2
1.1
1
0.9
0.8
0.7
0.6
0.5
0.4
0.3
0.2
0.1
0
-0.1
300
400
500
600

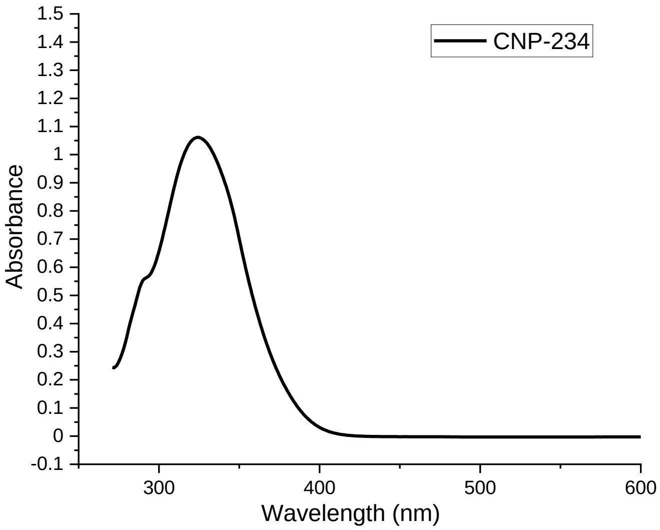

CNP-234
Absorbance
Wavelength (nm)

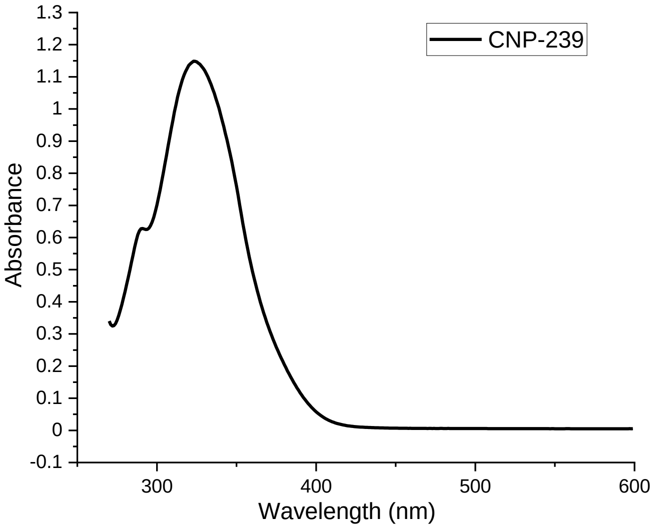

CNP-239
Absorbance
Wavelength (nm)

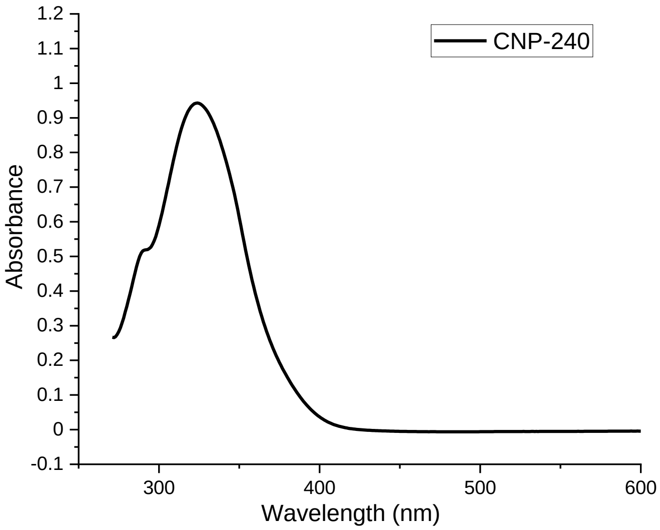
CNP-240
Absorbance
Wavelength (nm)
1.2
1.1
1
0.9
0.8
0.7
0.6
0.5
0.4
0.3
0.2
0.1
0
-0.1
300
400
500
600

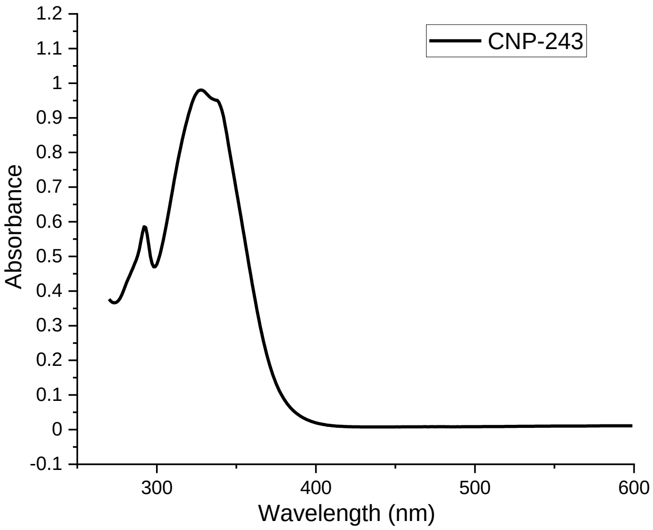
CNP-243
Absorbance
Wavelength (nm)
1.2
1.1
1
0.9
0.8
0.7
0.6
0.5
0.4
0.3
0.2
0.1
0
-0.1
300
400
500
600

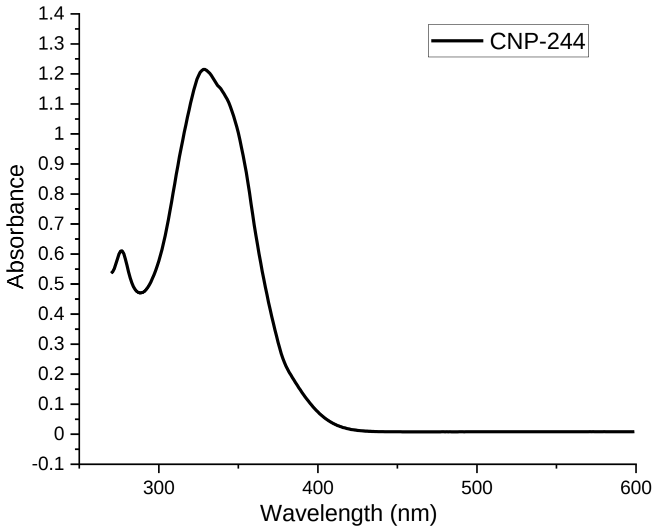
CNP-244
Absorbance
Wavelength (nm)
1.4
1.3
1.2
1.1
1
0.9
0.8
0.7
0.6
0.5
0.4
0.3
0.2
0.1
0
-0.1
300
400
500
600

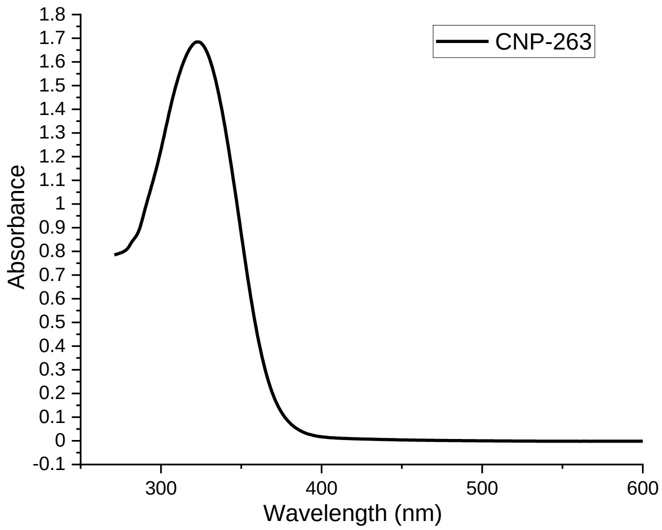
CNP-263
Absorbance
Wavelength (nm)
1.8
1.7
1.6
1.5
1.4
1.3
1.2
1.1
1
0.9
0.8
0.7
0.6
0.5
0.4
0.3
0.2
0.1
0
-0.1
300
400
500
600

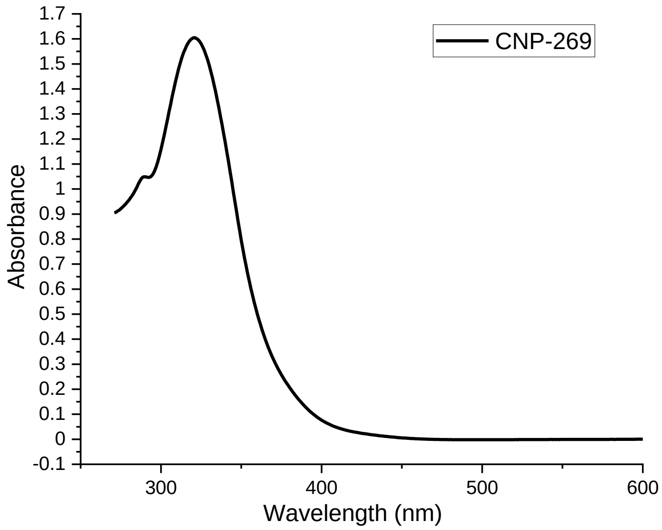

CNP-269
Absorbance
Wavelength (nm)
1.7
1.6
1.5
1.4
1.3
1.2
1.1
1
0.9
0.8
0.7
0.6
0.5
0.4
0.3
0.2
0.1
0
-0.1
300
400
500
600

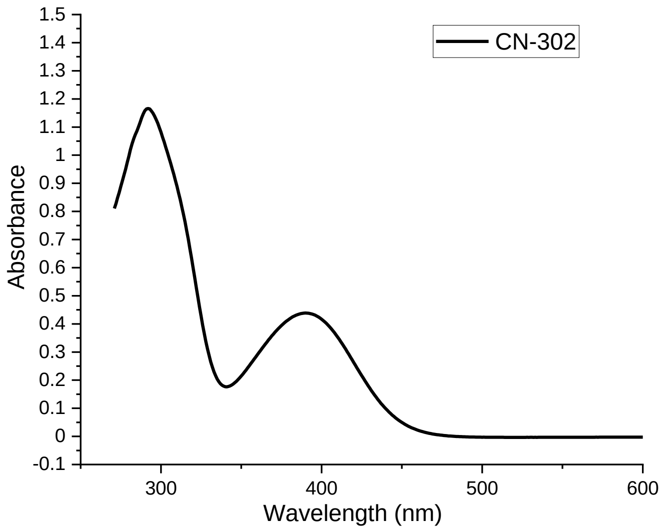

CN-302
Absorbance
Wavelength (nm)
1.5
1.4
1.3
1.2
1.1
1
0.9
0.8
0.7
0.6
0.5
0.4
0.3
0.2
0.1
0
-0.1
300
400
500
600

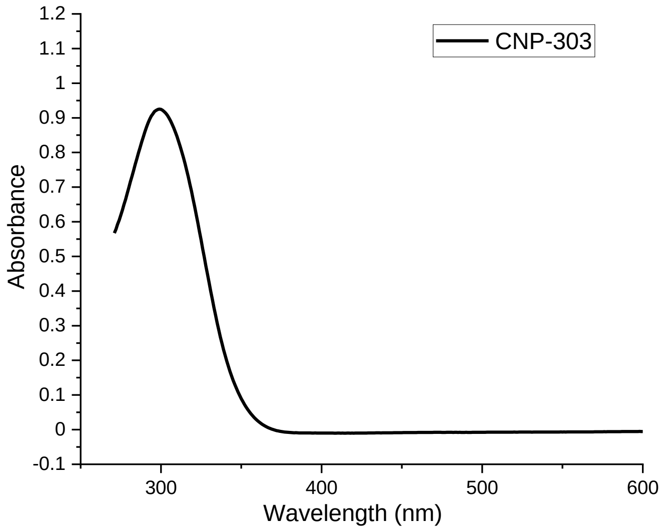

CNP-303
Absorbance
Wavelength (nm)
1.2
1.1
1
0.9
0.8
0.7
0.6
0.5
0.4
0.3
0.2
0.1
0
-0.1
300
400
500
600

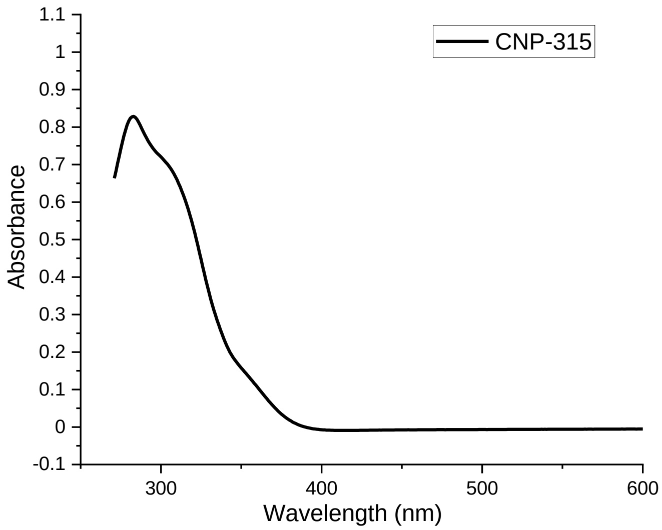

CNP-315
Absorbance
Wavelength (nm)
1.1
1
0.9
0.8
0.7
0.6
0.5
0.4
0.3
0.2
0.1
0
-0.1
300
400
500
600

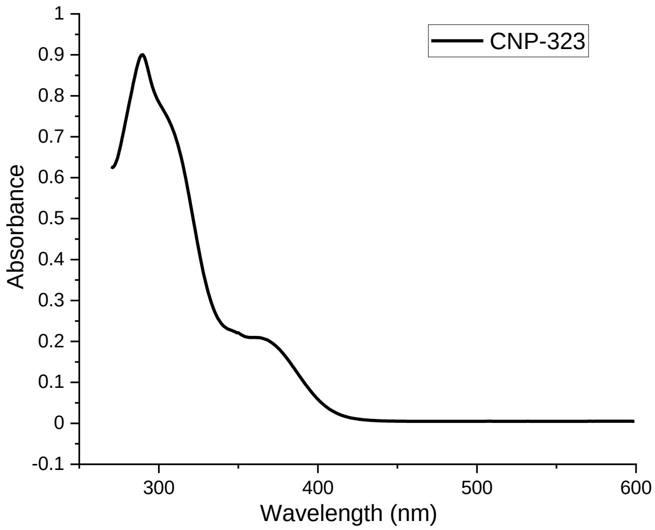

CNP-323
Absorbance
Wavelength (nm)
1
0.9
0.8
0.7
0.6
0.5
0.4
0.3
0.2
0.1
0
-0.1
300
400
500
600

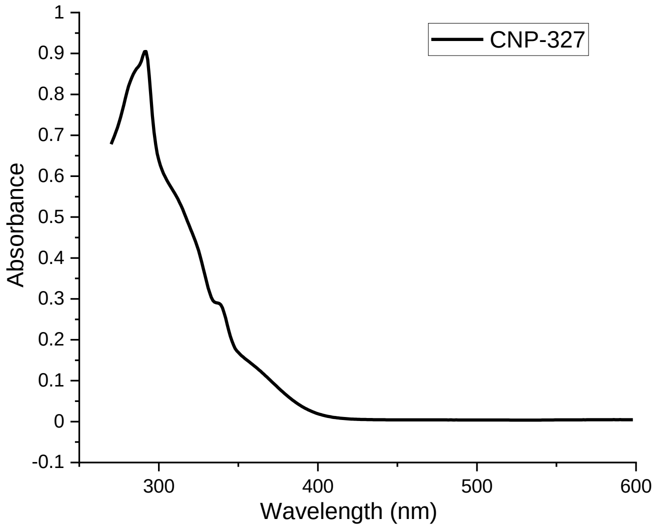

CNP-327
Absorbance
Wavelength (nm)
1
0.9
0.8
0.7
0.6
0.5
0.4
0.3
0.2
0.1
0
-0.1
300
400
500
600

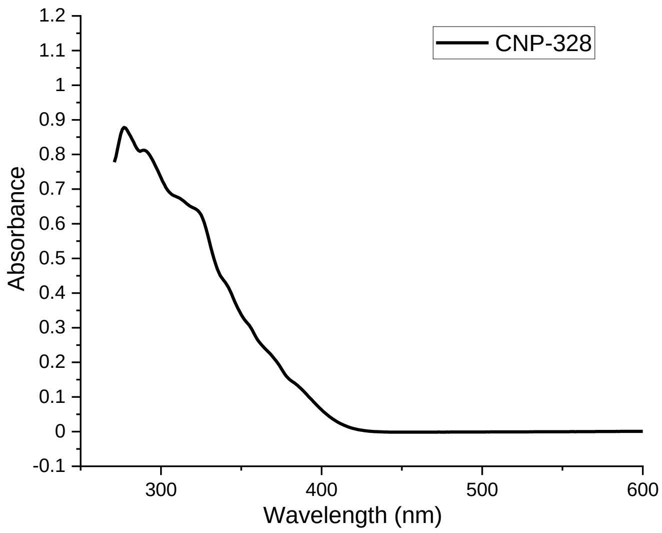

CNP-328
Absorbance
Wavelength (nm)
1.2
1.1
1
0.9
0.8
0.7
0.6
0.5
0.4
0.3
0.2
0.1
0
-0.1
300
400
500
600

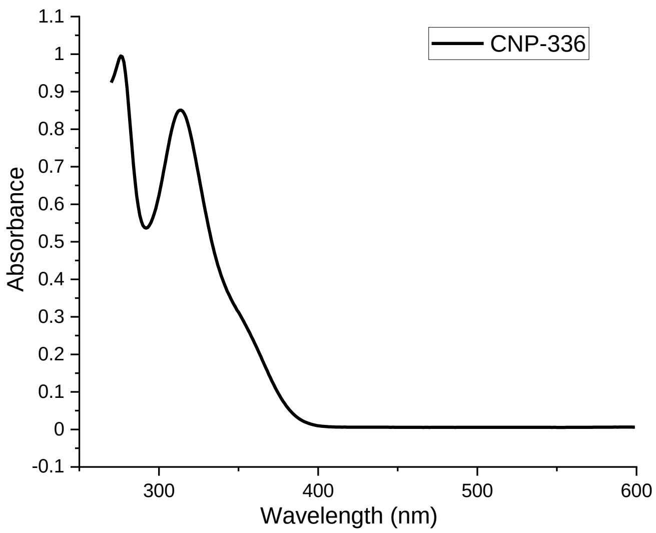

CNP-336
Absorbance
Wavelength (nm)
1.1
1
0.9
0.8
0.7
0.6
0.5
0.4
0.3
0.2
0.1
0
-0.1
300
400
500
600

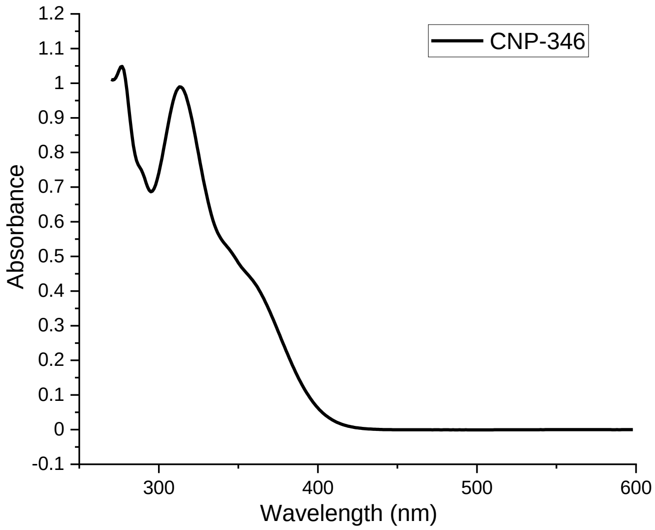
CNP-346
Absorbance
Wavelength (nm)
1.2
1.1
1
0.9
0.8
0.7
0.6
0.5
0.4
0.3
0.2
0.1
0
-0.1
300
400
500
600

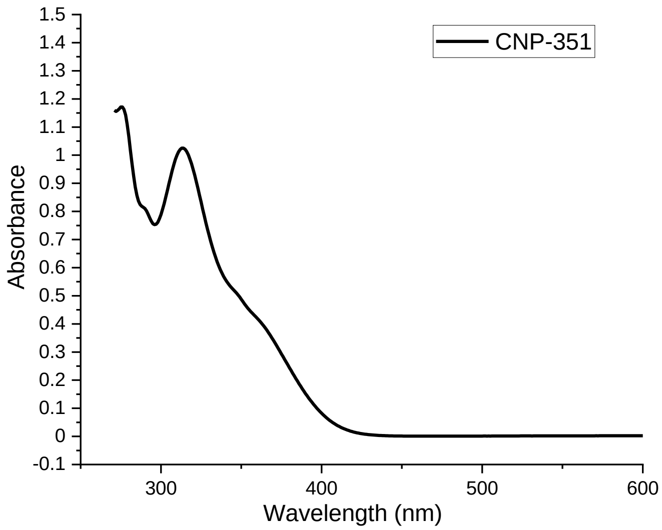
CNP-351
Absorbance
Wavelength (nm)
1.5
1.4
1.3
1.2
1.1
1
0.9
0.8
0.7
0.6
0.5
0.4
0.3
0.2
0.1
0
-0.1
300
400
500
600

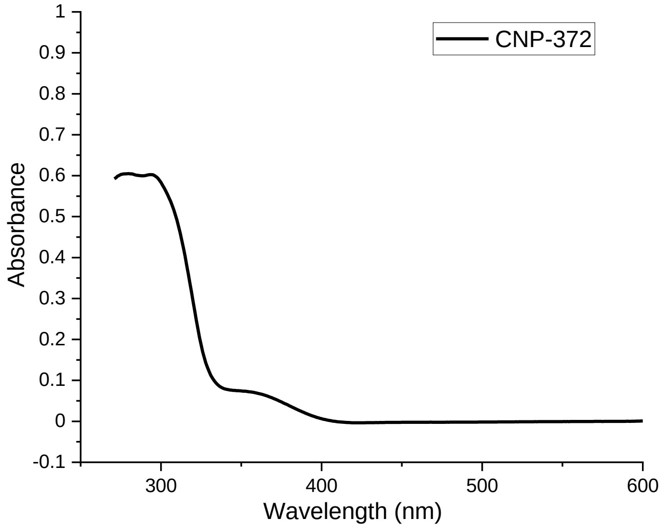
CNP-372
Absorbance
Wavelength (nm)
1
0.9
0.8
0.7
0.6
0.5
0.4
0.3
0.2
0.1
0
-0.1
300
400
500
600

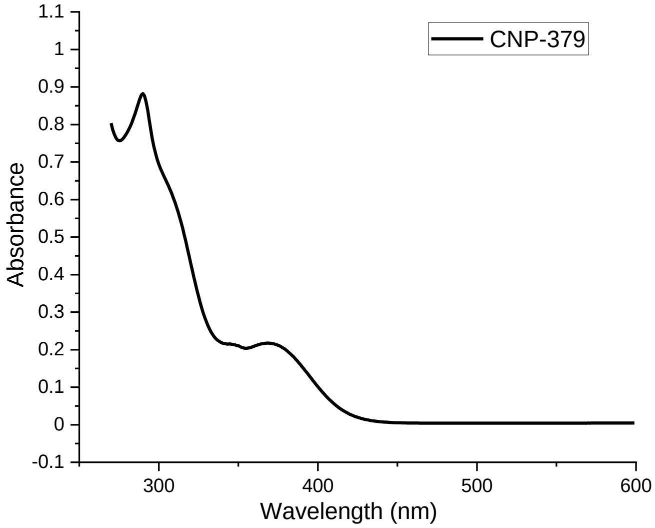
CNP-379
Absorbance
Wavelength (nm)
1.1
1
0.9
0.8
0.7
0.6
0.5
0.4
0.3
0.2
0.1
0
-0.1
300
400
500
600

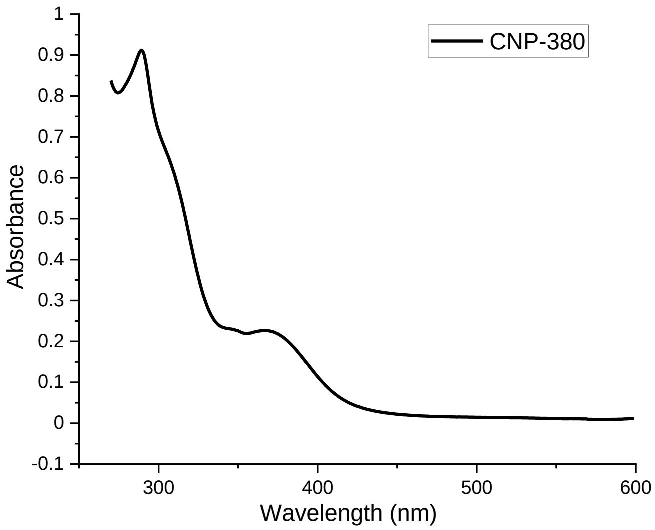

CNP-380
Absorbance
Wavelength (nm)
1
0.9
0.8
0.7
0.6
0.5
0.4
0.3
0.2
0.1
0
-0.1
300
400
500
600

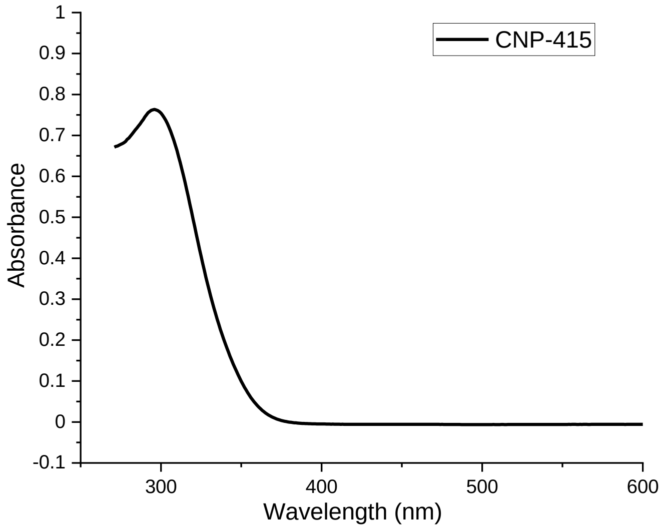

CNP-415
Absorbance
Wavelength (nm)
1
0.9
0.8
0.7
0.6
0.5
0.4
0.3
0.2
0.1
0
-0.1
300
400
500
600

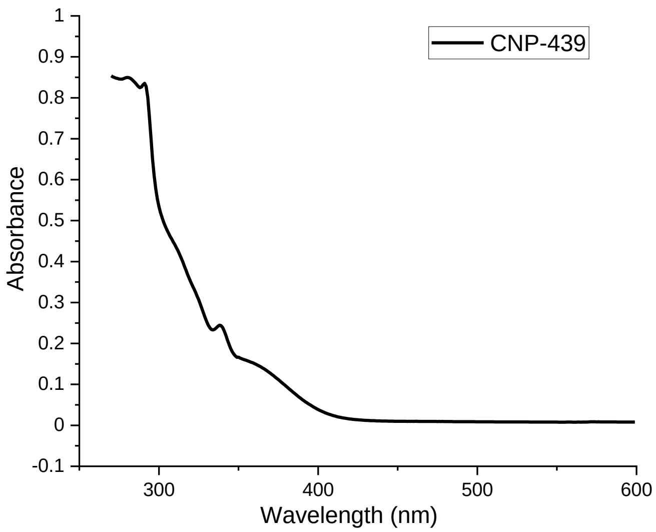
CNP-439
Absorbance
Wavelength (nm)
1
0.9
0.8
0.7
0.6
0.5
0.4
0.3
0.2
0.1
0
-0.1
300
400
500
600

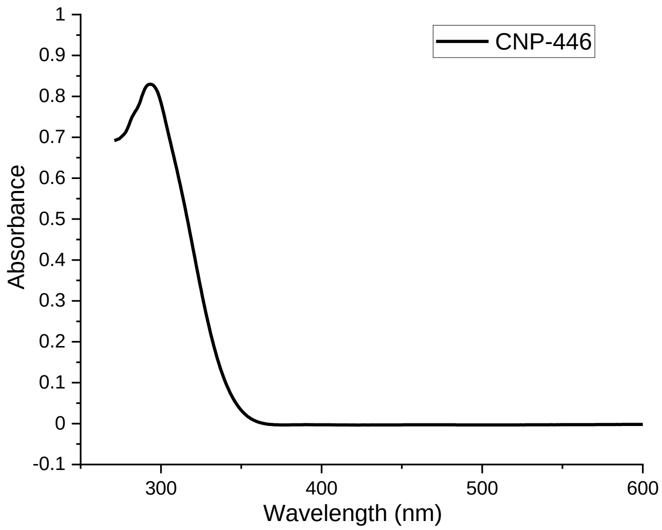
CNP-446
Absorbance
Wavelength (nm)
1
0.9
0.8
0.7
0.6
0.5
0.4
0.3
0.2
0.1
0
-0.1
300
400
500
600

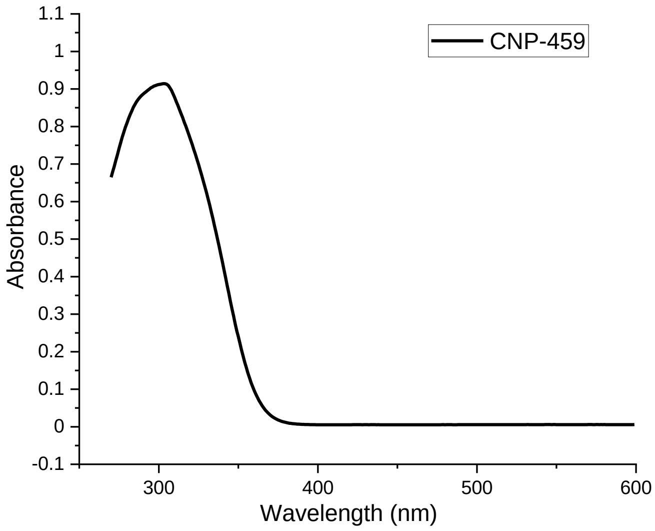

CNP-459
Absorbance
Wavelength (nm)
1.1
1
0.9
0.8
0.7
0.6
0.5
0.4
0.3
0.2
0.1
0
-0.1
300
400
500
600

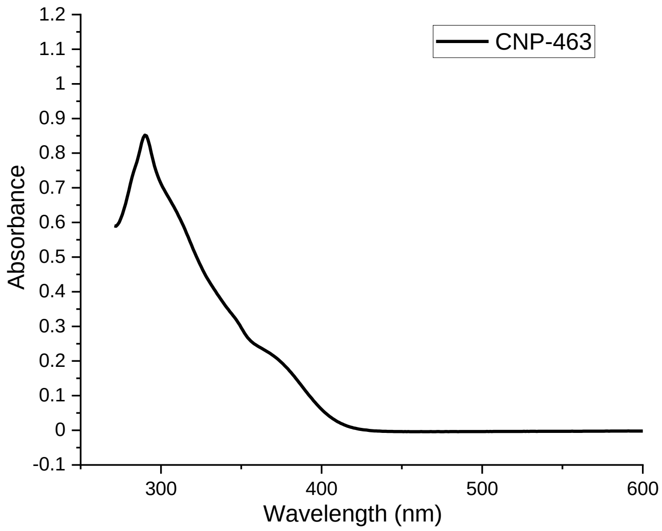

CNP-463
Absorbance
Wavelength (nm)
1.2
1.1
1
0.9
0.8
0.7
0.6
0.5
0.4
0.3
0.2
0.1
0
-0.1
300
400
500
600

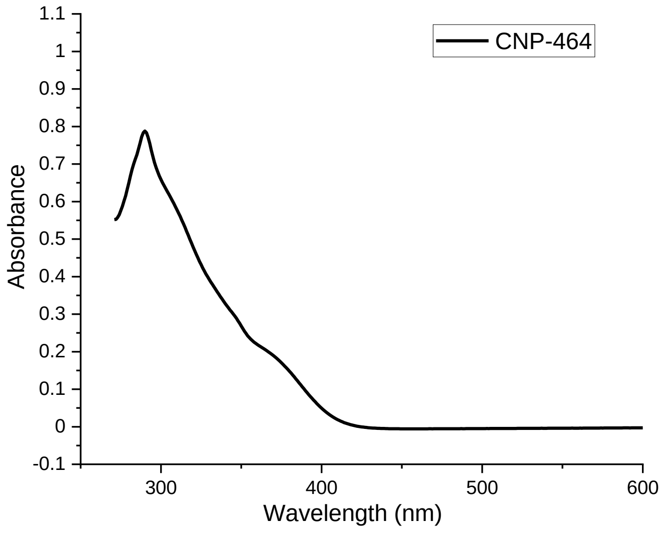
CNP-464
Absorbance
Wavelength (nm)
1.1
1
0.9
0.8
0.7
0.6
0.5
0.4
0.3
0.2
0.1
0
-0.1
300
400
500
600

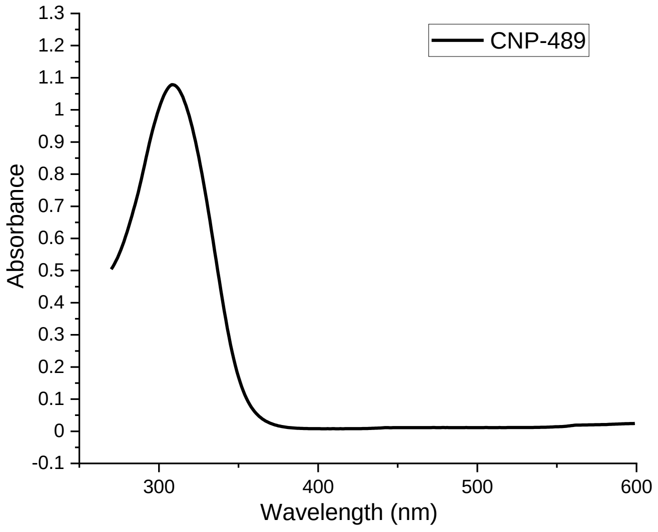
CNP-489
Absorbance
Wavelength (nm)
1.3
1.2
1.1
1
0.9
0.8
0.7
0.6
0.5
0.4
0.3
0.2
0.1
0
-0.1
300
400
500
600

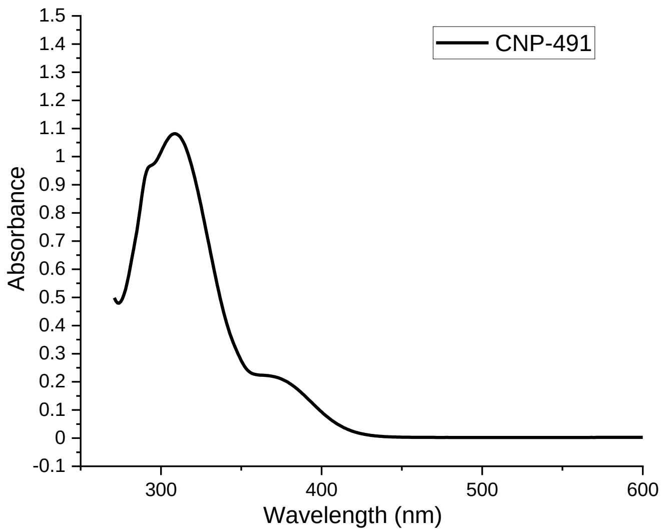
CNP-491
Absorbance
Wavelength (nm)
1.5
1.4
1.3
1.2
1.1
1
0.9
0.8
0.7
0.6
0.5
0.4
0.3
0.2
0.1
0
-0.1
300
400
500
600

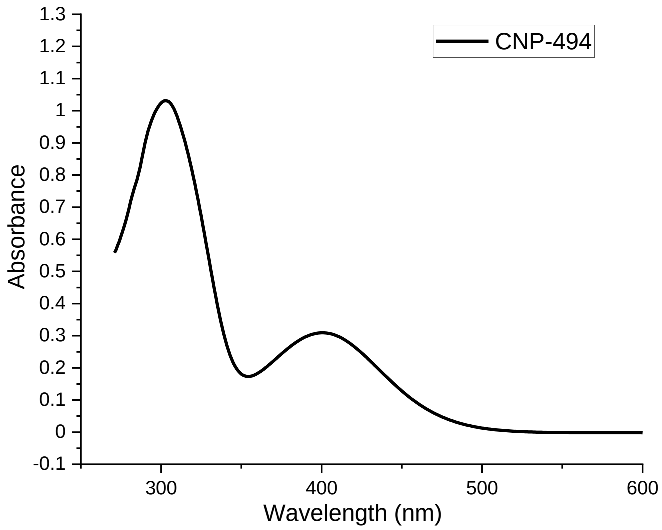
CNP-494
Absorbance
Wavelength (nm)
1.3
1.2
1.1
1
0.9
0.8
0.7
0.6
0.5
0.4
0.3
0.2
0.1
0
-0.1
300
400
500
600

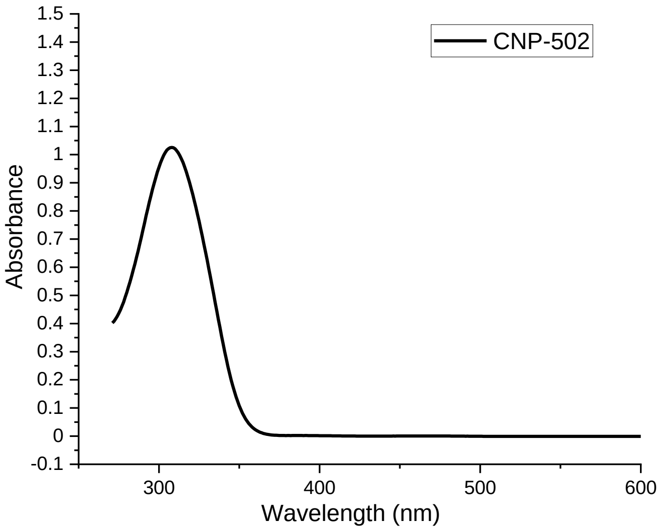

CNP-502
Absorbance
Wavelength (nm)
1.5
1.4
1.3
1.2
1.1
1
0.9
0.8
0.7
0.6
0.5
0.4
0.3
0.2
0.1
0
-0.1
300
400
500
600

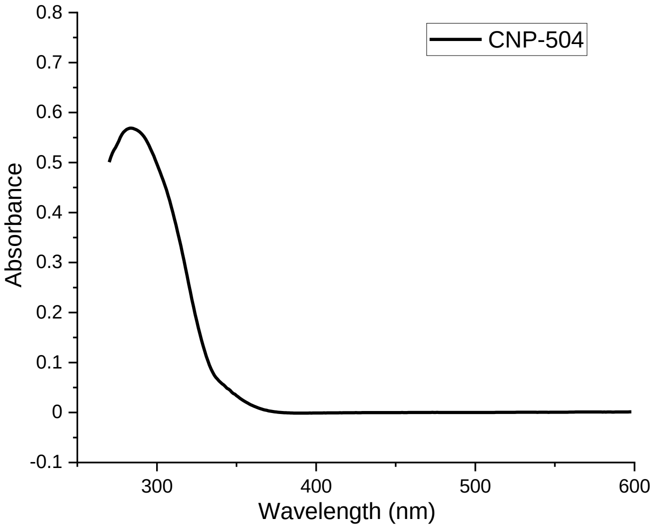

CNP-504
Absorbance
Wavelength (nm)
0.8
0.7
0.6
0.5
0.4
0.3
0.2
0.1
0
-0.1
300
400
500
600

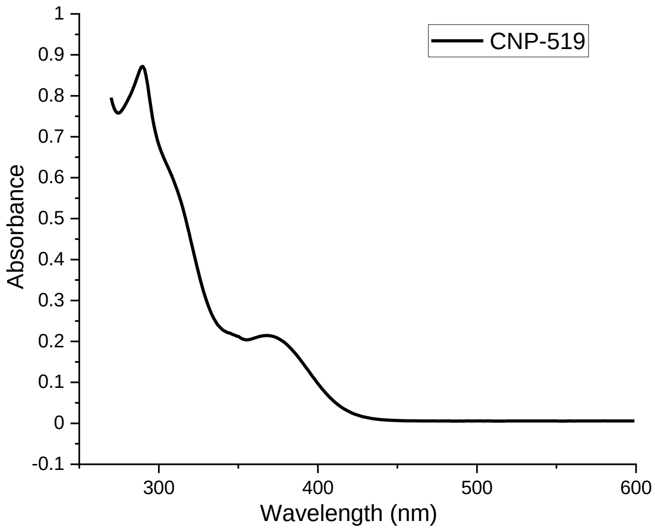
CNP-519
Absorbance
Wavelength (nm)
1
0.9
0.8
0.7
0.6
0.5
0.4
0.3
0.2
0.1
0
-0.1
300
400
500
600

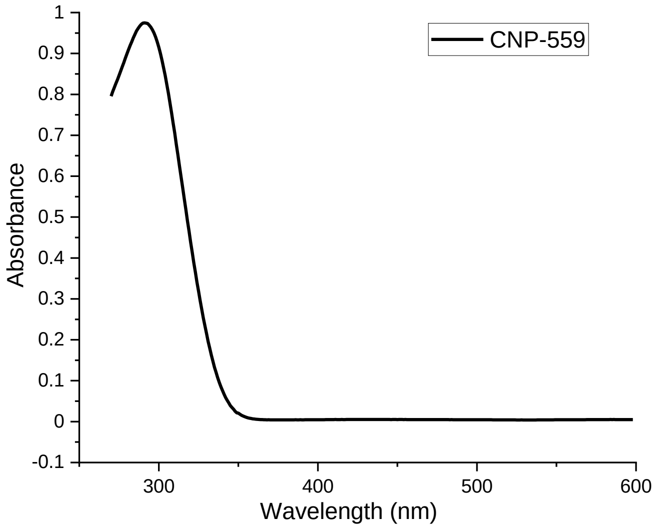
CNP-559
Absorbance
Wavelength (nm)
1
0.9
0.8
0.7
0.6
0.5
0.4
0.3
0.2
0.1
0
-0.1
300
400
500
600

NMR, elemental analysis, and mass spectrometry of CNPs

CNP-3

^{1}H NMR (400 MHz, DMSO) δ 8.97 (1H), 8.30 (2H), 8.22 – 8.05 (4H), 7.78 (2H), 7.72 – 7.54 (10H). ^{13}C NMR (101 MHz, DMSO) δ 163.41, 158.60, 136.84, 131.75, 131.12, 130.63, 130.08, 129.41, 129.38, 129.16, 28.39, 127.37, 126.42, 125.04, 116.03, 108.63. Chemical Formula: $C_{33}H_{19}N_3$, Elemental Analysis: calcd., C, 86.63; H, 4.19; N, 9.18; found C, 86.34; H, 4.09; N,9.12. HRMS (m/z): $[M+H]^+$ calcd., 458.1652; found, 458.1645.

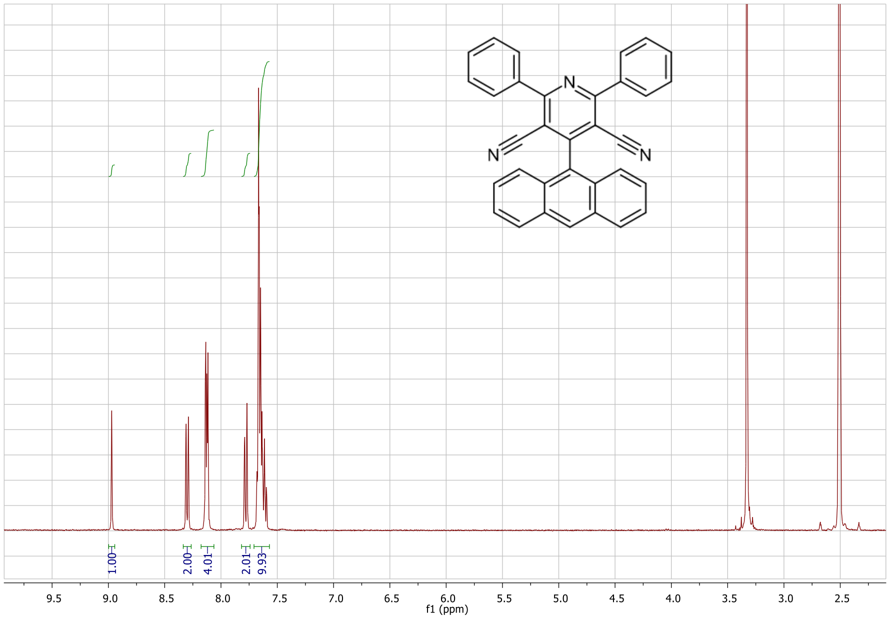

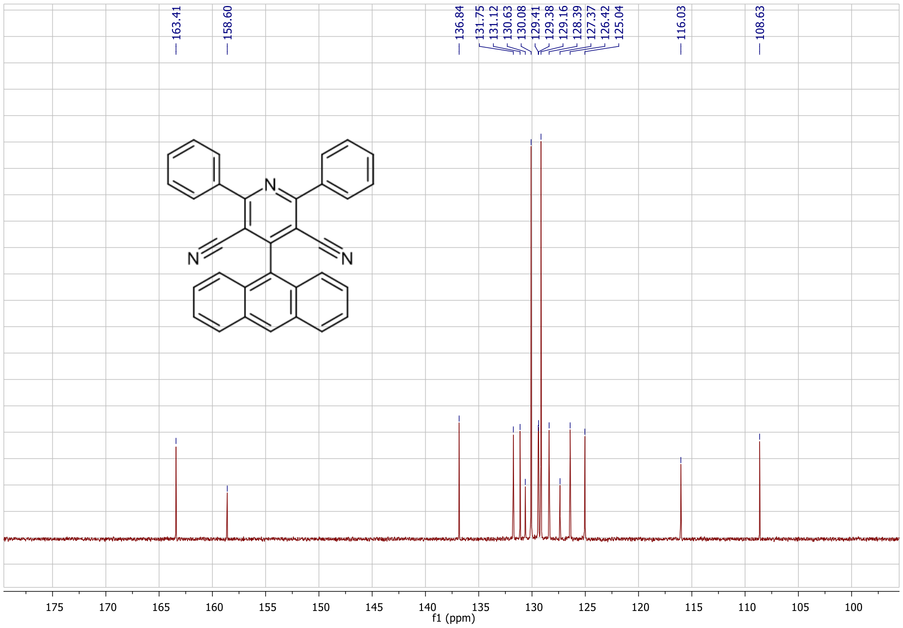

CNP-11

^{1}H NMR (400 MHz, DMSO) δ 8.09 – 8.04 (4H), 7.93 – 7.83 (4H), 7.68 – 7.59 (8H), 7.51 – 7.45 (3H). ^{13}C NMR (101 MHz, DMSO) δ 163.05, 159.09, 136.89, 132.15, 132.03, 130.22, 129.96, 129.35, 129.17, 125.17, 116.49, 106.67, 91.90, 88.79. Chemical Formula: $C_{33}H_{19}N_3$, Elemental Analysis: calcd., C, 86.63; H, 4.19; N, 9.18; found C, 86.12; H, 4.15; N, 9.10. HRMS (m/z): $[M+H]^+$ calcd., 458.1652; found, 458.1642.

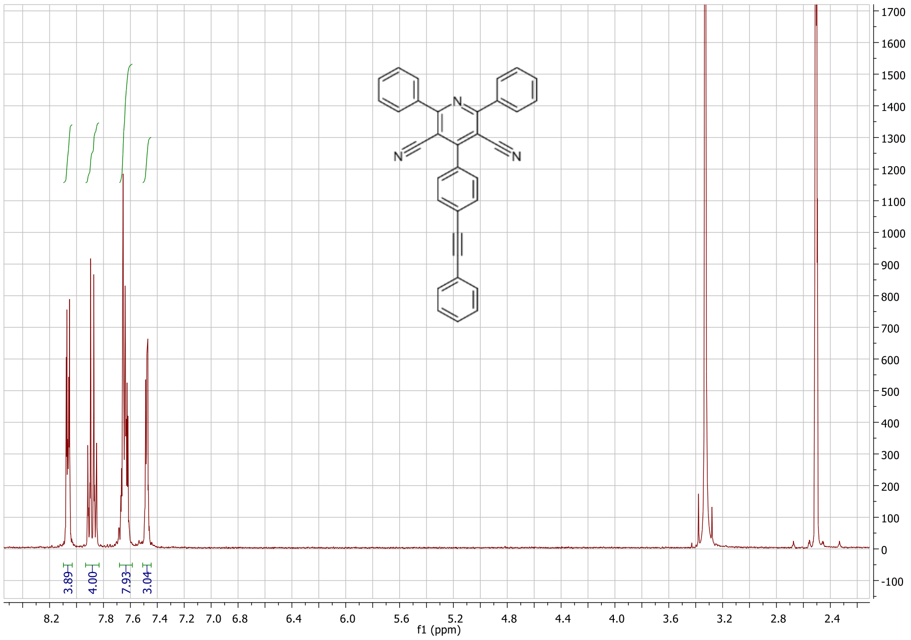

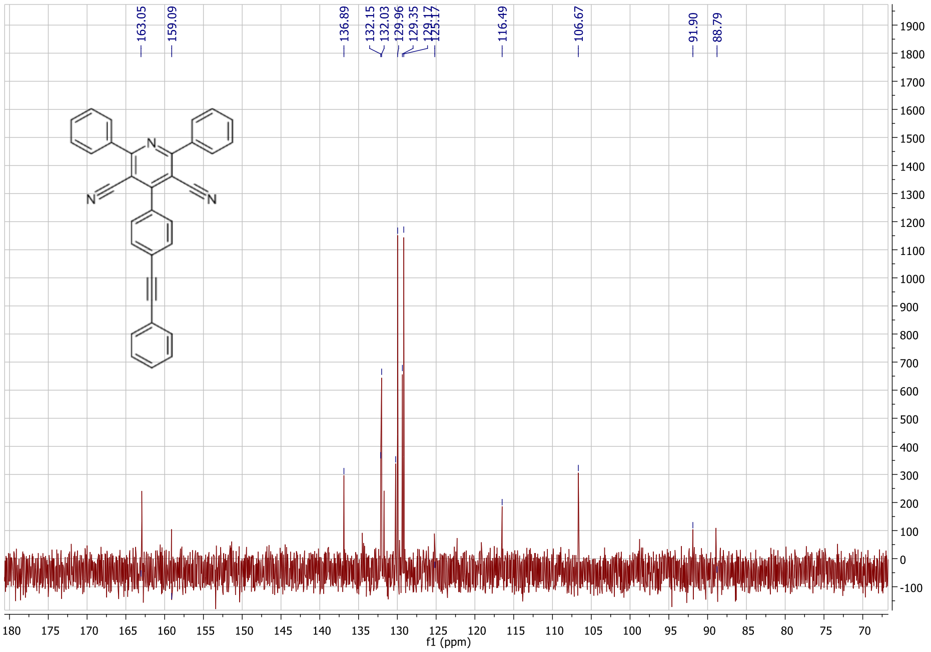

CNP-13

^{1}H NMR (400 MHz, DMSO) δ 8.12 – 8.06 (4H), 8.07 – 7.98 (4H), 7.85 (1H), 7.83 – 7.77 (2H), 7.70 – 7.63 (7H), 7.63 – 7.54 (3H). ^{13}C NMR (101 MHz, DMSO) δ 163.06, 159.58, 142.83, 138.84, 137.00, 133.95, 133.50, 131.68, 131.07, 130.59, 130.02, 129.18, 129.03, 128.74, 127.72, 127.21, 126.61, 126.14, 125.31, 116.68, 106.87. Chemical Formula: $C_{35}H_{21}N_3$, Elemental Analysis: calcd., C, 86.93; H, 4.38; N, 8.69; found C, 86.46; H, 4.38; N, 8.57. HRMS (m/z): $[M+H]^+$ calcd., 484.1808; found, 484.1803.

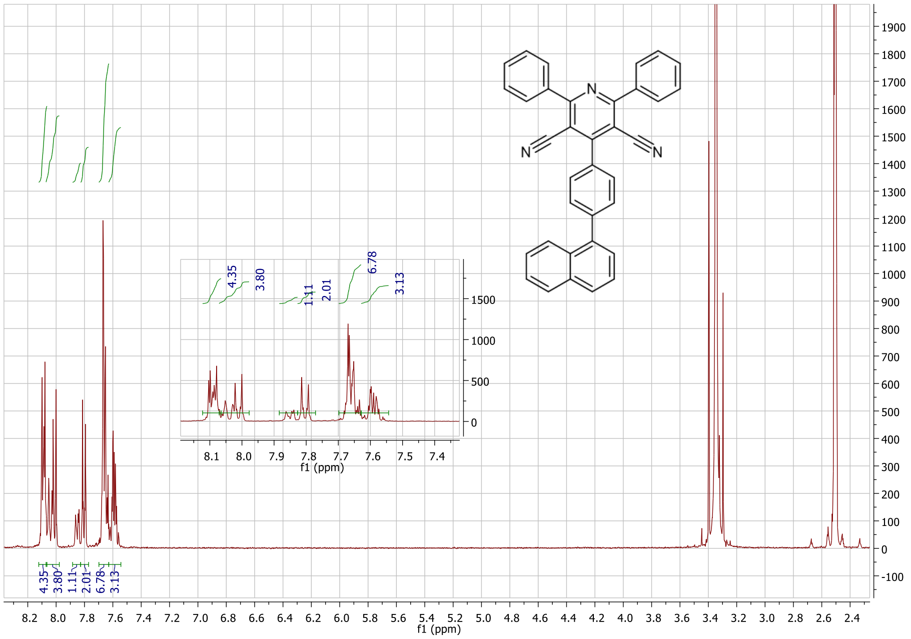

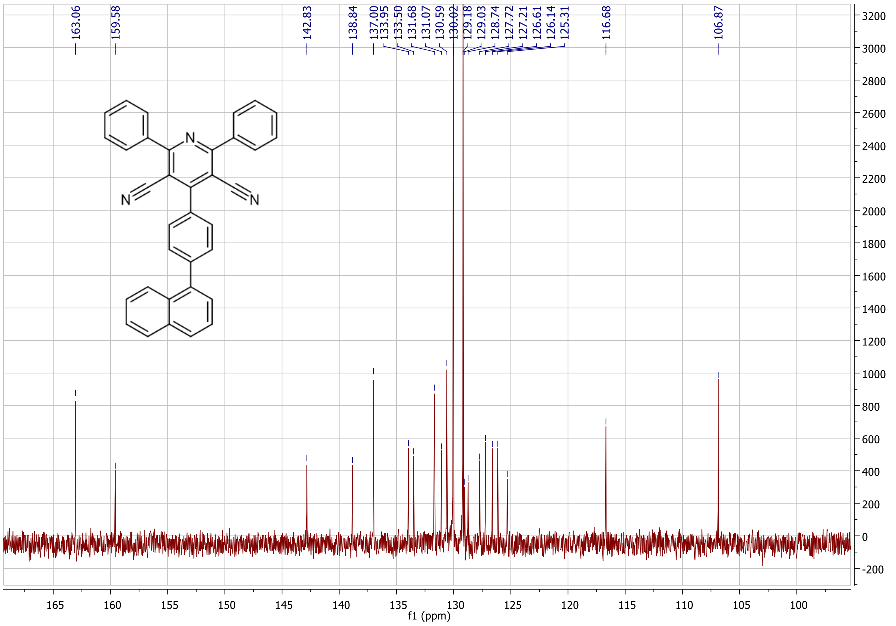

CNP-21

^{1}H NMR (400 MHz, DMSO) δ 8.22 (1H), 8.15 – 8.01 (4H), 7.81 – 7.67 (4H), 7.67 – 7.59 (7H), 2.80 (3H). ^{13}C NMR (101 MHz, DMSO) δ 162.86, 159.58, 137.94, 136.90, 132.53, 131.65, 130.45, 129.99, 129.15, 127.87, 127.79, 127.21, 126.57, 125.81, 125.39, 116.26, 108.08, 19.76. Chemical Formula: $C_{30}H_{19}N_3$, Elemental Analysis: calcd., C, 85.49; H, 4.54; N, 9.97; found, C, 85.22; H, 4.52; N, 9.96. HRMS (m/z): $[M+H]^+$ calcd., 422.1652; found, 422.1655.

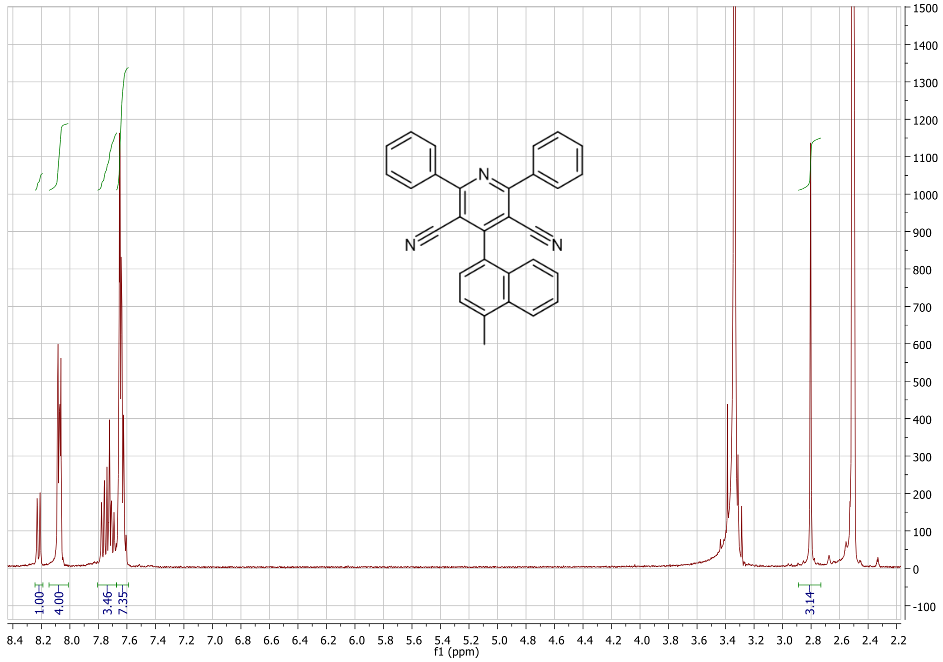

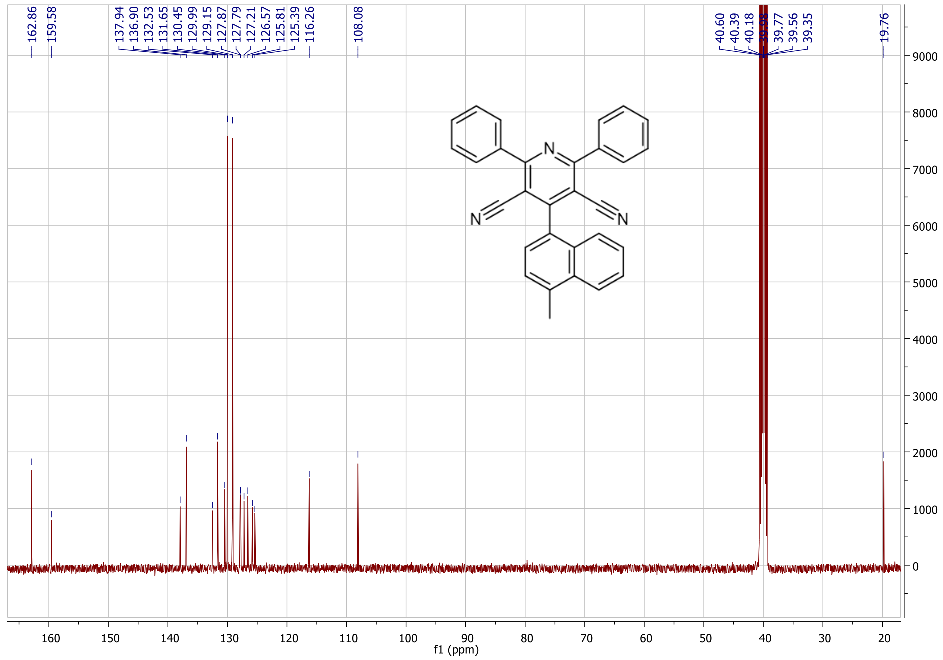

CNP-29

^{1}H NMR (400 MHz, DMSO) δ 8.62 (2H), 8.44 (2H), 8.18 – 8.10 (2H), 8.07 – 7.94 (4H), 7.92 – 7.81 (4H), 7.59 – 7.51 (2H), 7.50 – 7.41 (1H). ^{13}C NMR (101 MHz, DMSO) δ 161.19, 159.14, 143.11, 139.22, 137.82, 135.18, 134.70, 133.74, 132.91, 130.56, 129.61, 128.83, 127.49, 118.65, 116.24, 112.41, 108.06. Chemical Formula: $C_{33}H_{17}N_5$, Elemental Analysis: calcd., C, 81.97; H, 3.54; N, 14.48; found, C, 82.09; H, 3.51; N, 14.53. HRMS (m/z): $[M+H]^+$ calcd., 484.1557; found, 484.1553.

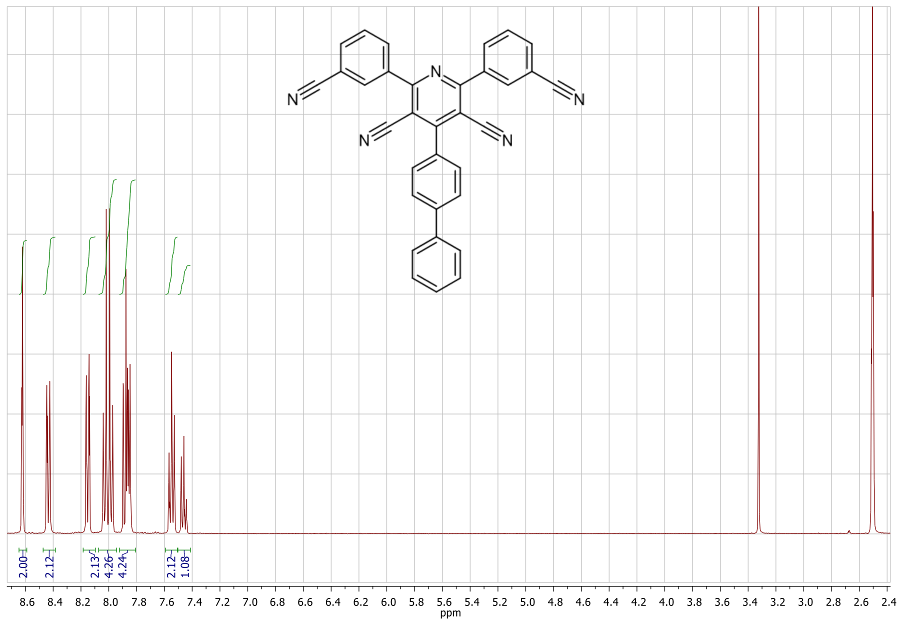

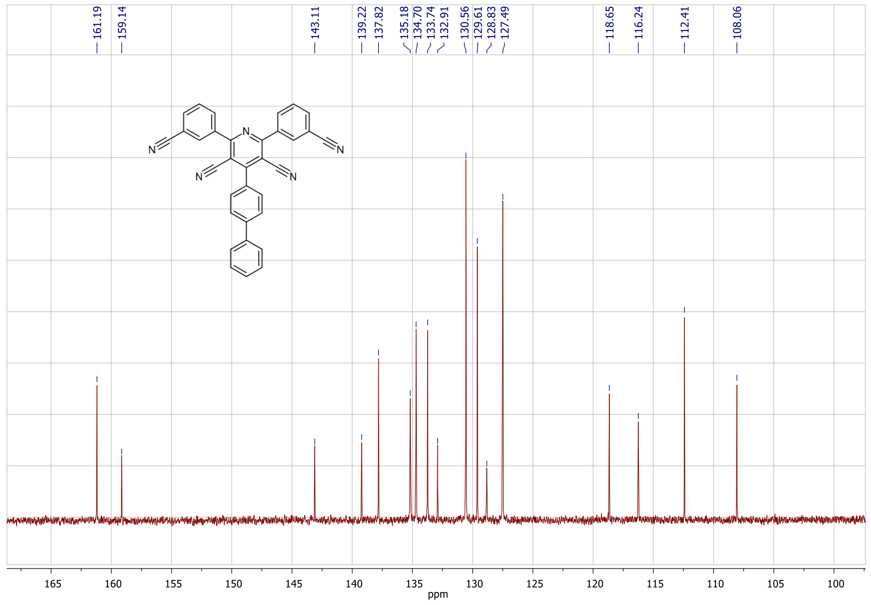

CNP-31

^{1}H NMR (400 MHz, DMSO) δ 8.99 (1H), 8.67 – 8.60 (2H), 8.47 (2H), 8.30 (2H), 8.18 – 8.11 (2H), 7.92 – 7.83 (2H), 7.79 – 7.72 (2H), 7.64 (4H). ^{13}C NMR (101 MHz, DMSO) δ 161.63, 158.53, 137.73, 135.17, 134.81, 133.79, 131.08, 130.89, 130.54, 129.39, 128.36, 126.80, 126.44, 125.17, 118.65, 115.55, 112.35, 110.13. Chemical Formula: $C_{35}H_{17}N_5$, Elemental Analysis: calcd., C, 82.83; H, 3.38; N, 13.80; found, C, 81.19; H, 3.30; N, 13.52. HRMS (m/z): $[M+H]^+$ calcd., 508.1557; found, 508.1549.

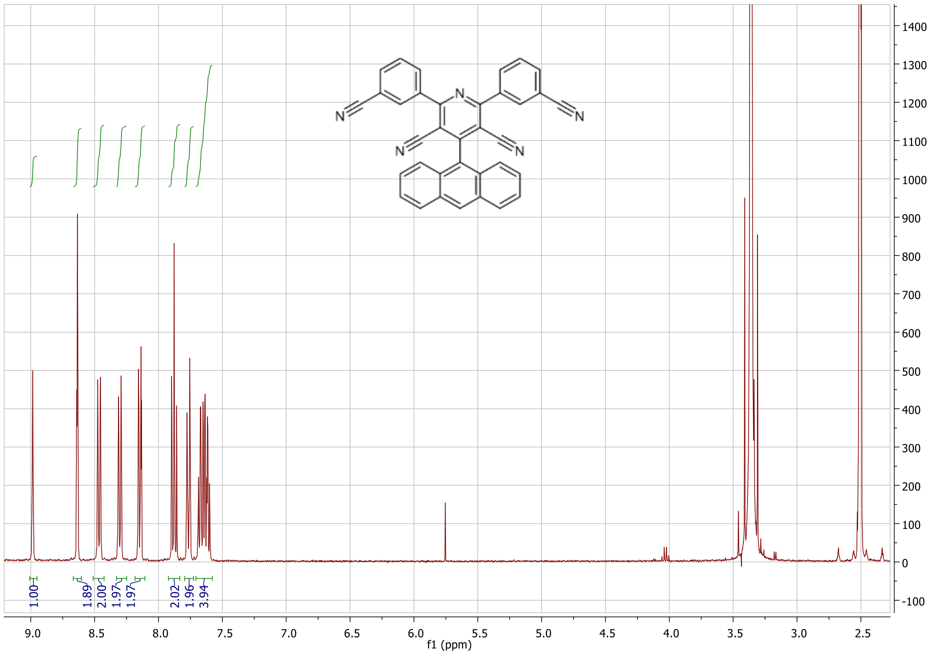

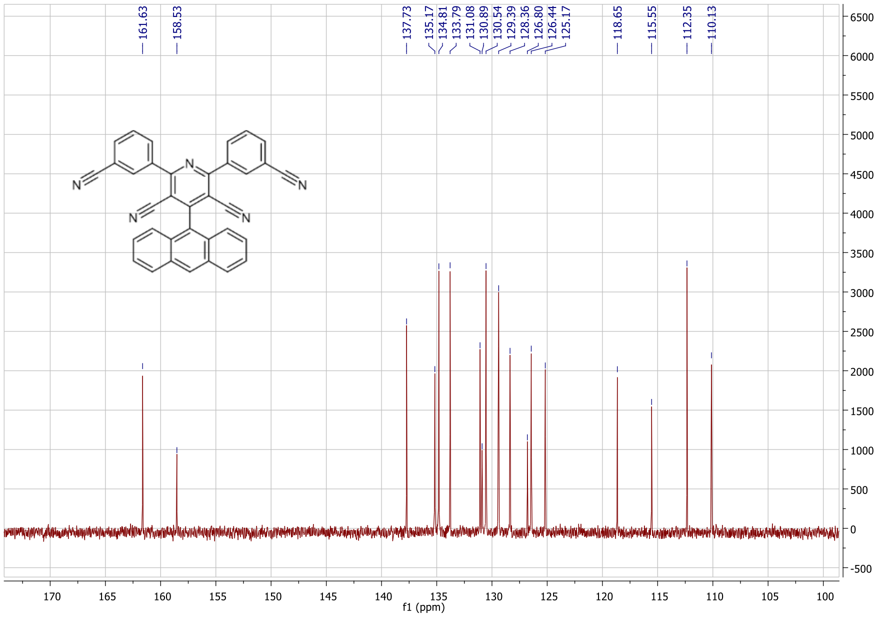

CNP-55

^{1}H NMR (400 MHz, DMSO) δ 8.62 (2H), 8.43 (2H), 8.18 – 8.06 (6H), 8.05 – 7.98 (4H), 7.87 (2H). ^{13}C NMR (101 MHz, DMSO) δ 161.16, 158.88, 143.69, 141.12, 137.77, 135.21, 134.69, 134.11, 133.74, 133.48, 130.58, 128.44, 118.65, 116.16, 112.43, 111.31, 108.06. Chemical Formula: $C_{34}H_{16}N_{6}$, Elemental Analysis: calcd., C, 80.30; H, 3.17; N, 16.53; found, C, 78.89; H, 3.14; N, 16.13. HRMS (m/z): $[M+H]^{+}$ calcd., 509.1509; found, 509.1506.

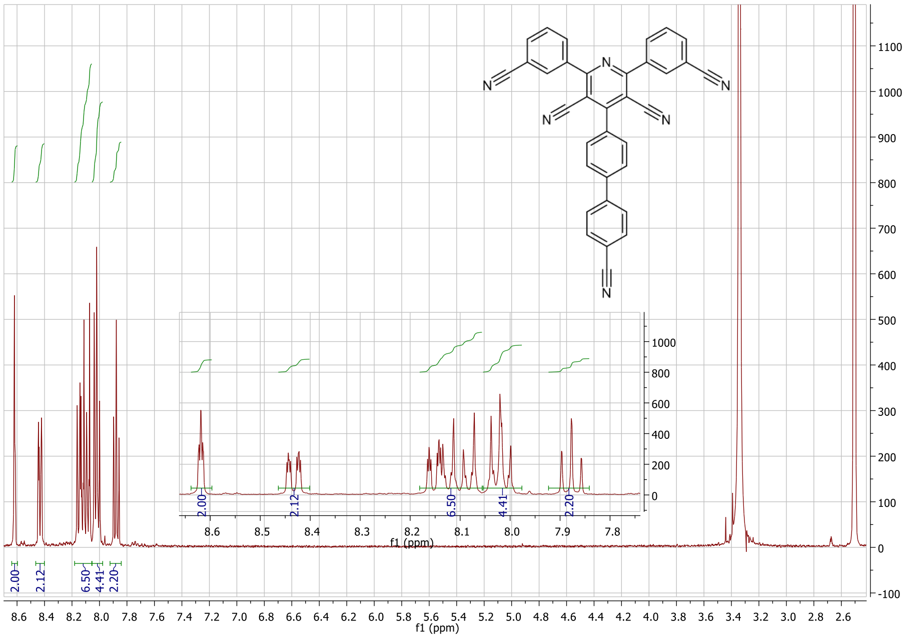

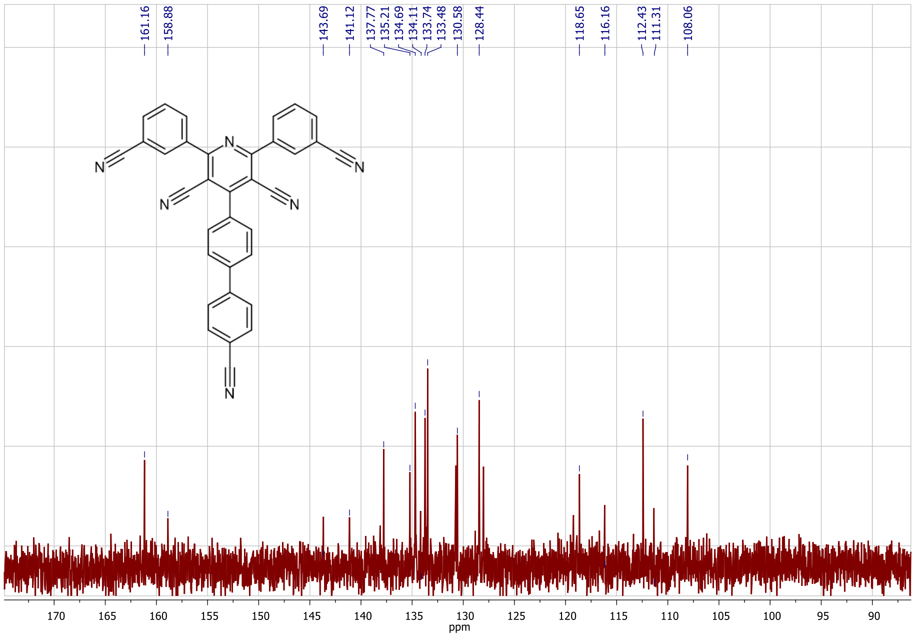

CNP-56

^{1}H NMR (400 MHz, DMSO) δ 8.31 (5H), 8.24 – 8.13 (5H), 7.93 – 7.82 (3H), 7.71 (2H). ^{13}C NMR (101 MHz, DMSO) δ 161.43, 159.22, 140.78, 133.48, 133.13, 131.58, 131.54, 130.91, 130.27, 129.15, 128.28, 128.19, 127.41, 125.94, 125.26, 118.72, 115.65, 114.11, 109.43. Chemical Formula: $C_{31}H_{15}N_5$, Elemental Analysis: calcd., C, 81.39; H, 3.30; N, 15.31; found, C, 81.11; H, 3.33; N, 15.12. HRMS (m/z): $[M+H]^+$ calcd., 458.1400; found, 458.1392.

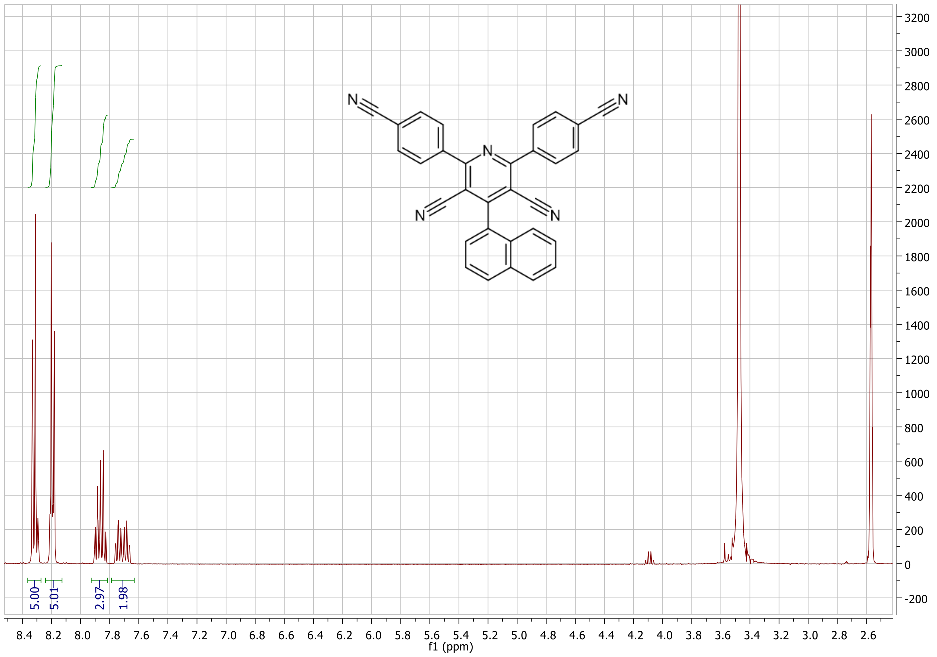

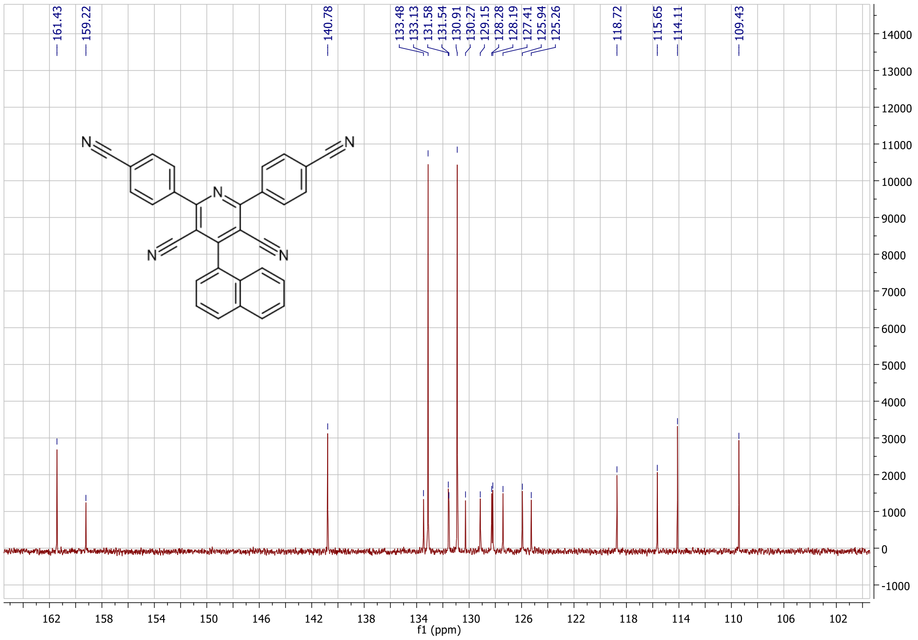

CNP-58

^{1}H NMR (400 MHz, DMSO) δ 8.32 – 8.21 (4H), 8.18 – 8.08 (4H), 7.82 (1H), 7.59 (3H), 7.52 – 7.39 (1H), 3.50 (4H). ^{13}C NMR (101 MHz, DMSO) δ 161.55, 158.99, 150.34, 147.10, 140.85, 139.04, 133.12, 130.92, 130.50, 130.01, 128.72, 126.79, 121.04, 120.37, 119.59, 118.73, 115.85, 114.09, 109.38, 30.48. Chemical Formula: $C_{33}H_{17}N_5$, Elemental Analysis: calcd., C, 81.97; H, 3.54; N, 14.48; found C, 80.83; H, 3.63; N, 14.03. HRMS (m/z): [M+H]$^+$ calcd., 484.1557; found, 484.1554.

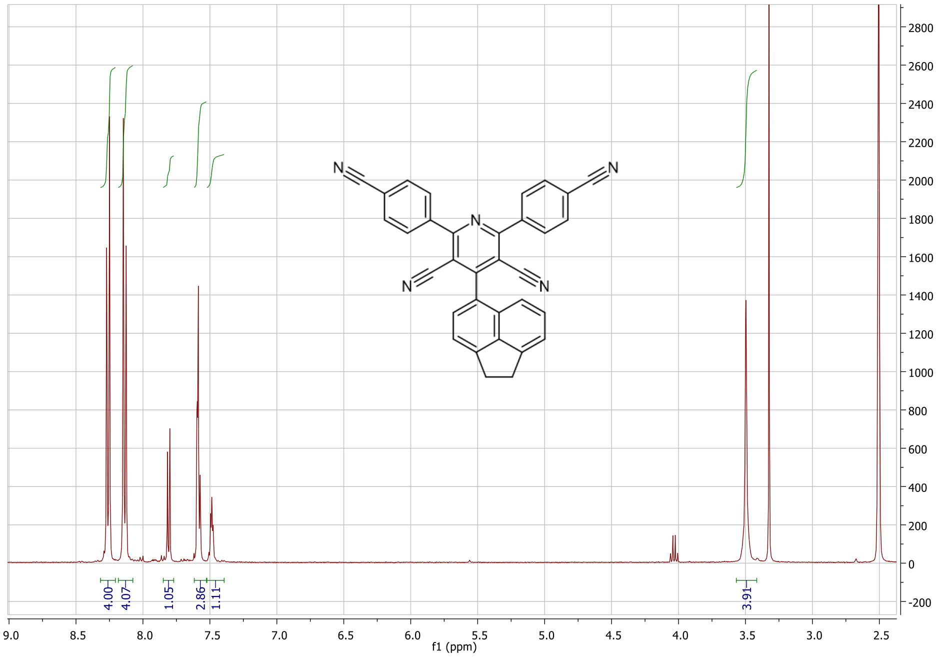

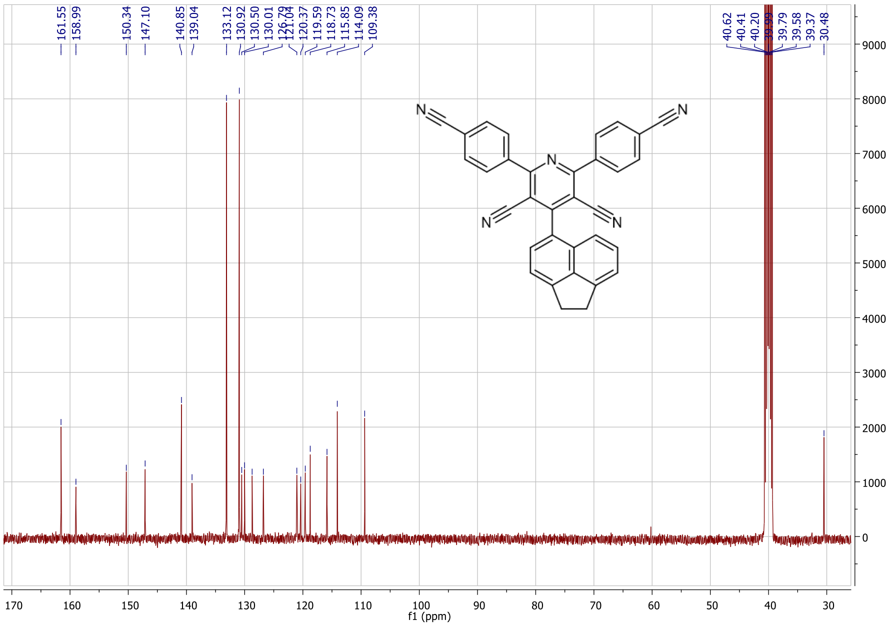

CNP-62

^{1}H NMR (400 MHz, DMSO) δ 8.31 – 8.25 (4H), 8.22 (1H), 8.17 – 8.10 (4H), 8.03 – 7.96 (1H), 7.90 – 7.84 (1H), 7.83 – 7.74 (3H), 7.55 (2H), 7.45 (1H). ^{13}C NMR (101 MHz, DMSO) δ 161.50, 159.34, 141.09, 140.84, 139.52, 134.44, 133.15, 130.93, 129.63, 128.70, 127.36, 118.60, 116.11, 114.17, 108.33. Chemical Formula: $C_{33}H_{17}N_5$, Elemental Analysis: calcd., C, 81.97; H, 3.54; N, 14.48; found, C, 79.84; H, 3.46; N, 14.03. HRMS (m/z): $[M+H]^+$ calcd., 484.1557; found, 484.1553.

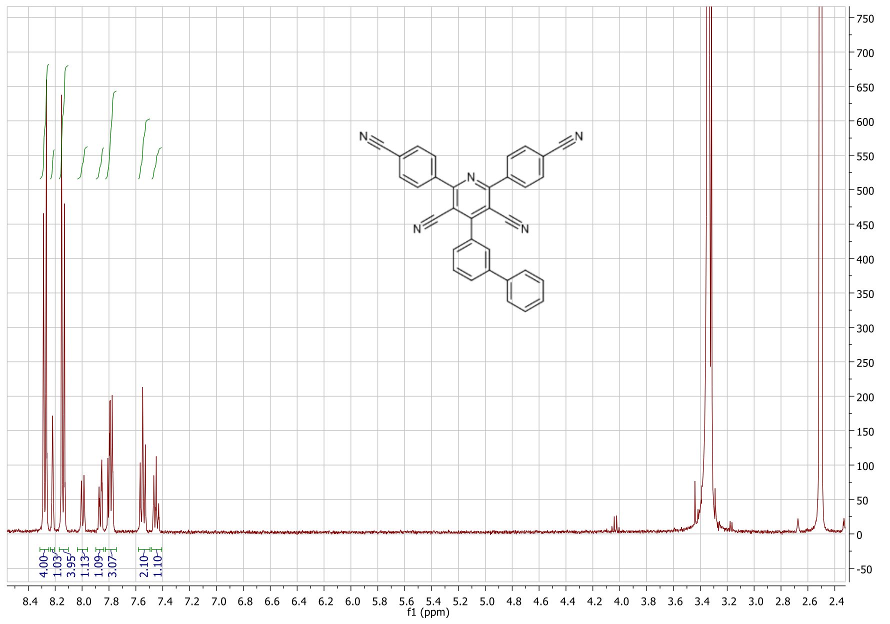

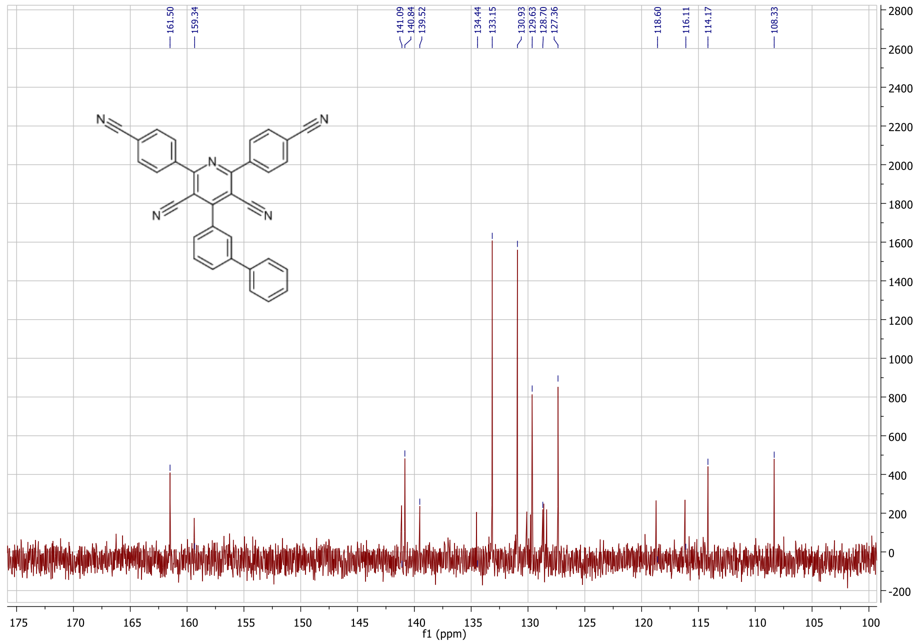

CNP-64

^{1}H NMR (400 MHz, DMSO) δ 8.36 – 8.31 (1H), 8.29 – 8.22 (4H), 8.17 – 8.09 (4H), 7.82 – 7.74 (2H), 7.70 – 7.60 (2H), 7.26 (1H), 4.11 (3H). ^{13}C NMR (101 MHz, DMSO) δ 161.45, 159.35, 157.38, 140.87, 133.10, 131.37, 130.89, 129.47, 128.56, 126.77, 125.26, 125.13, 123.65, 122.62, 118.74, 115.85, 114.05, 109.81, 104.59, 56.50. Chemical Formula: $C_{32}H_{17}N_5O$, Elemental Analysis: calcd., C, 78.84; H, 3.51; N, 14.37; found C, 78.56; H, 3.48; N, 14.27. HRMS (m/z): $[M+H]^+$ calcd., 488.1506; found, 488.1492.

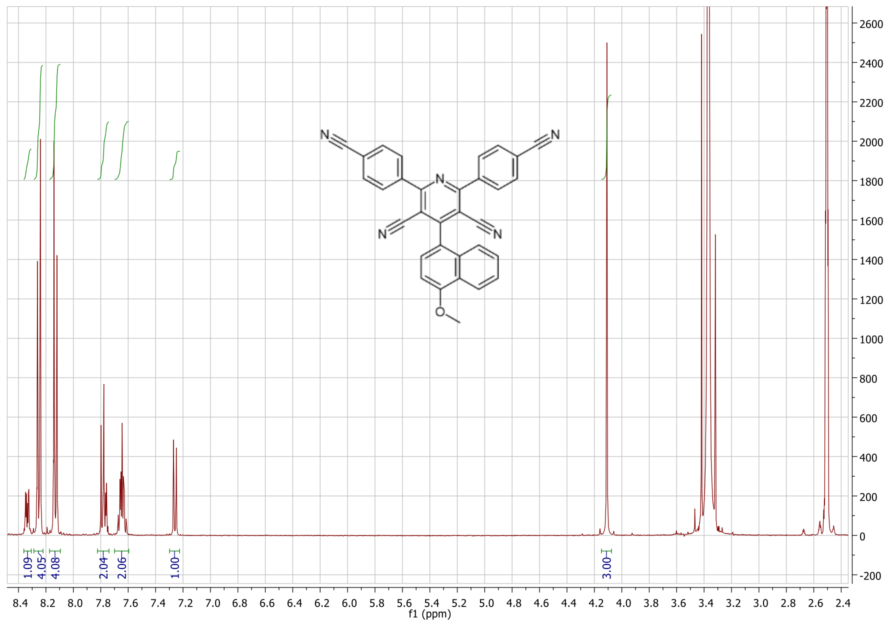

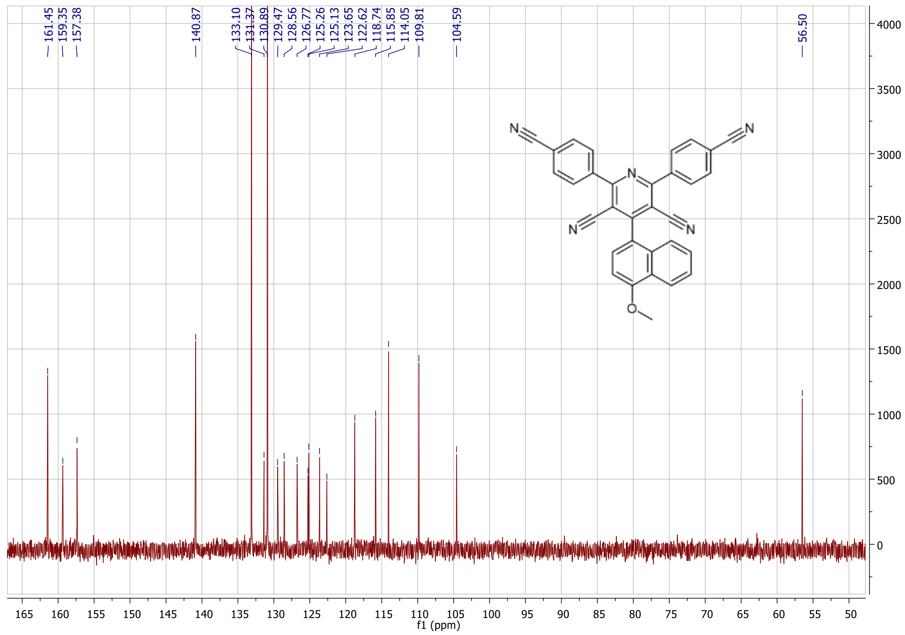

CNP-65

^{1}H NMR (400 MHz, DMSO) δ 8.22 (4H), 8.12 (4H), 7.72 (2H), 7.48 – 7.39 (4H), 7.28 – 7.18 (6H), 7.04 (2H). ^{13}C NMR (101 MHz, DMSO) δ 161.73, 159.00, 150.45, 146.38, 141.00, 133.06, 131.39, 130.89, 130.50, 126.51, 125.53, 125.14, 119.28, 118.73, 116.40, 114.02, 107.85. Chemical Formula: $C_{39}H_{22}N_6$, Elemental Analysis: calcd., C, 81.52; H, 3.86; N, 14.62; found C, 81.05; H, 3.93; N, 14.33. HRMS (m/z): $[M+H]^+$ calcd., 575.1979; found, 575.1972.

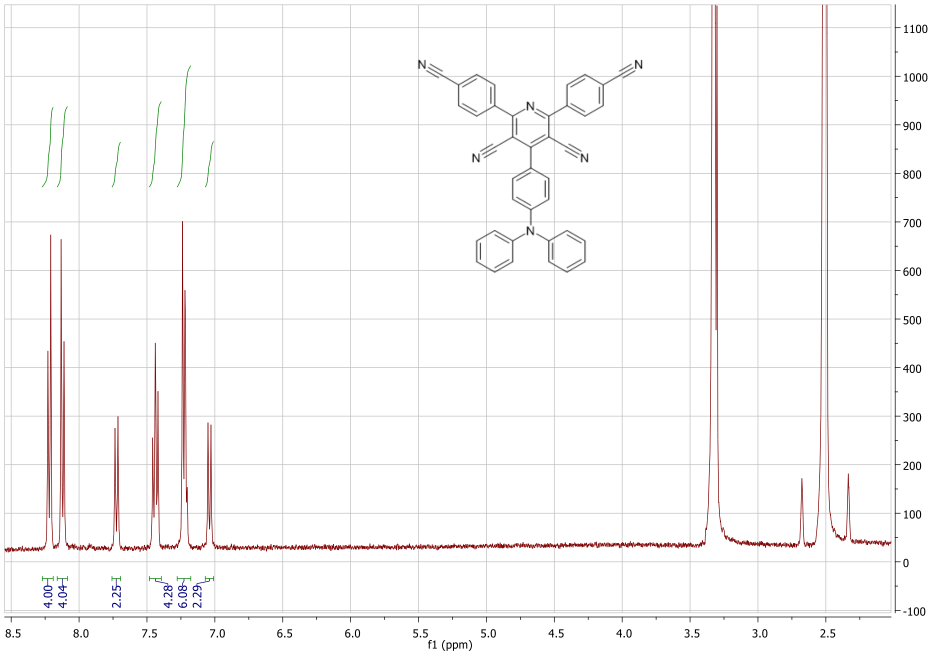

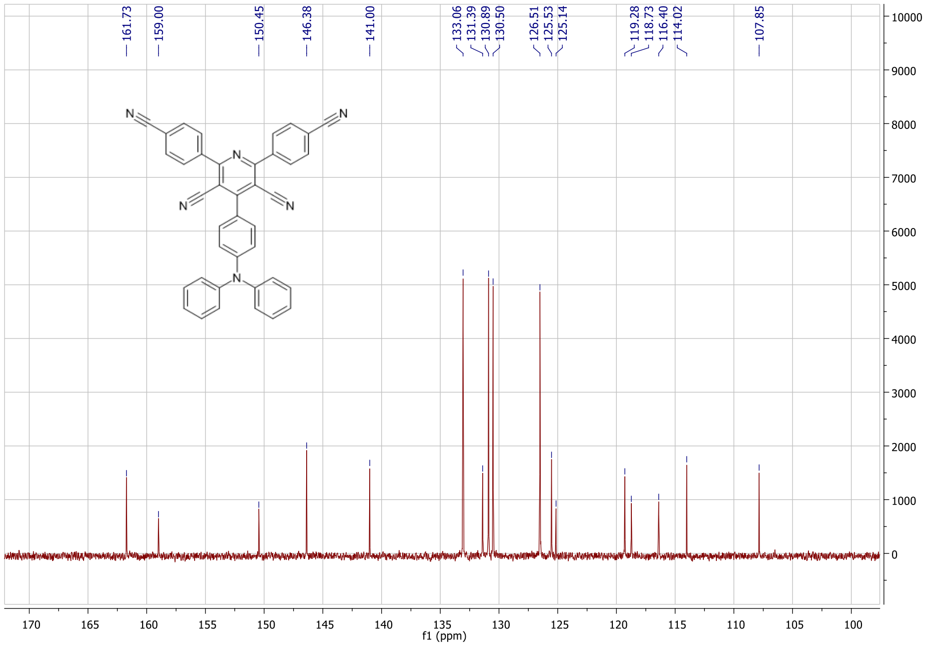

CNP-68

^{1}H NMR (400 MHz, DMSO) δ 8.35 – 8.27 (4H), 8.23 – 8.16 (4H), 7.95 (4H), 7.76 – 7.68 (2H), 7.57 (1H), 7.52 – 7.43 (3H), 7.41 – 7.35 (1H). ^{13}C NMR (101 MHz, DMSO) δ 161.53, 159.24, 140.86, 140.37, 137.20, 133.14, 132.68, 131.50, 130.91, 130.33, 129.29, 128.68, 127.73, 127.29, 127.26, 118.73, 116.14, 114.14, 108.12. Chemical Formula: $C_{35}H_{19}N_5$, Elemental Analysis: calcd., C, 82.50; H, 3.76; N, 13.74; found, C, 83.18; H, 3.74; N, 13.87. HRMS (m/z): $[M+H]^+$ calcd., 510.1713; found, 510.1709.

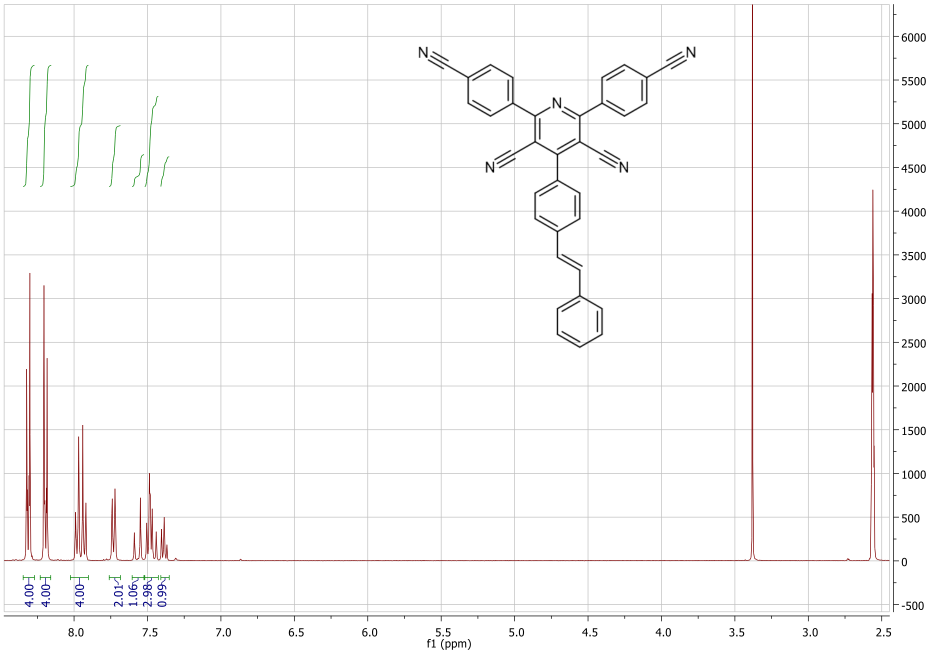

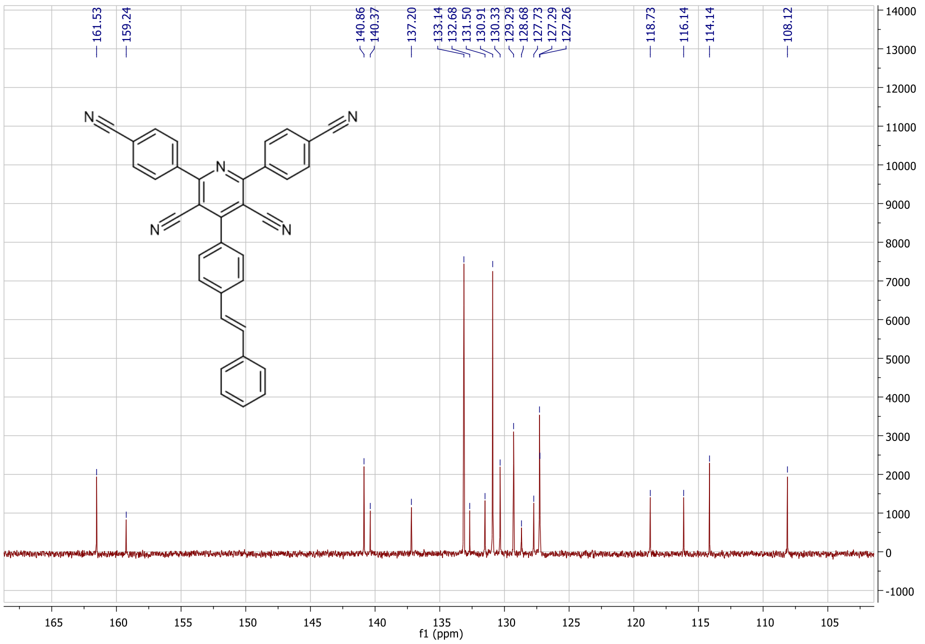

CNP-69

^{1}H NMR (400 MHz, DMSO) δ 8.29 (4H), 8.16 (4H), 8.09 – 7.98 (4H), 7.84 (3H), 7.65 (1H), 7.62 – 7.54 (3H). ^{13}C NMR (101 MHz, DMSO) δ 161.55, 159.34, 143.15, 140.86, 138.73, 133.94, 133.17, 133.00, 131.03, 130.93, 130.71, 130.01, 129.04, 128.79, 127.75, 127.24, 126.62, 126.13, 125.27, 118.73, 116.14, 114.18, 108.32. Chemical Formula: $C_{37}H_{19}N_5$, Elemental Analysis: calcd., C, 83.29; H, 3.59; N, 13.13; found C, 82.88; H, 3.55; N, 13.10. HRMS (m/z): $[M+H]^+$ calcd., 534.1713; found, 534.1699.

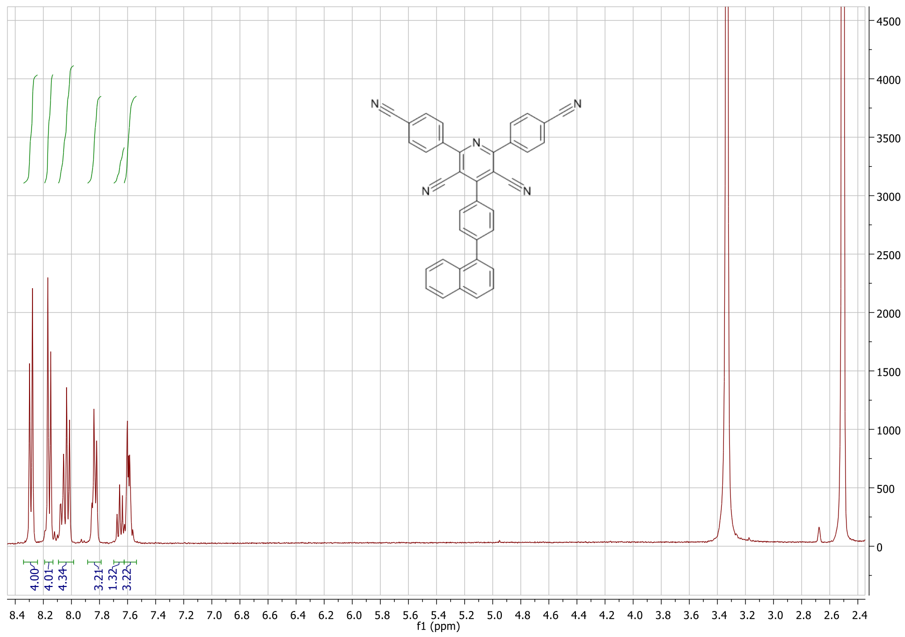

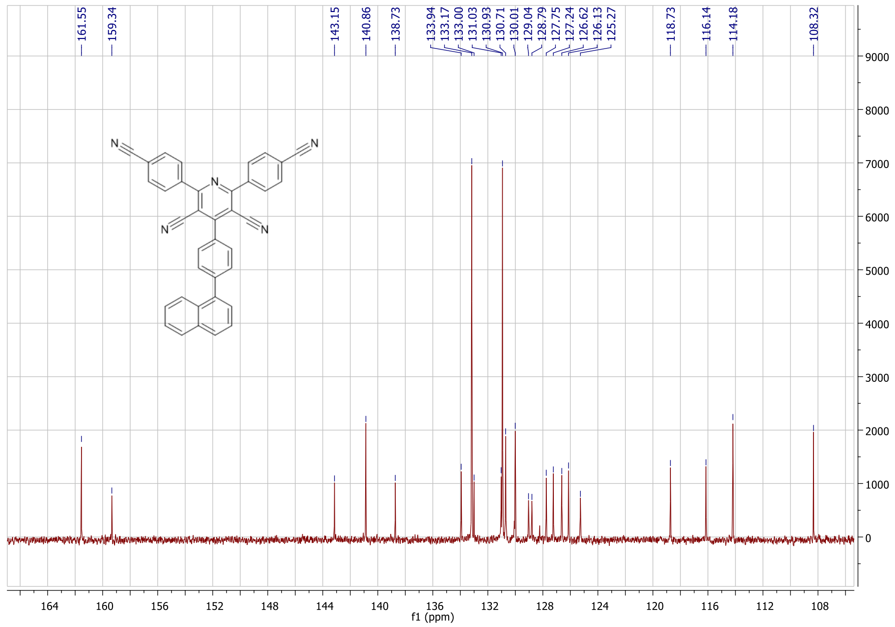

CNP-74

^{1}H NMR (400 MHz, DMSO) δ 8.26 – 8.15 (4H), 8.15 – 8.06 (4H), 7.71 – 7.56 (2H), 7.31 – 7.19 (4H), 7.08 – 6.96 (4H), 6.85 – 6.68 (2H), 3.78 (6H). ^{13}C NMR (101 MHz, DMSO) δ 161.82, 157.56, 151.62, 141.08, 138.84, 133.04, 130.89, 129.00, 118.68, 116.50, 115.81, 113.98, 107.66, 55.79. Chemical Formula: $C_{41}H_{26}N_6O_2$, Elemental Analysis: calcd., C, 77.59; H, 4.13; N, 13.24; found, C, 76.50; H, 4.26; N, 12.66. HRMS (m/z): $[M+H]^+$ calcd., 635.2190; found, 635.2184.

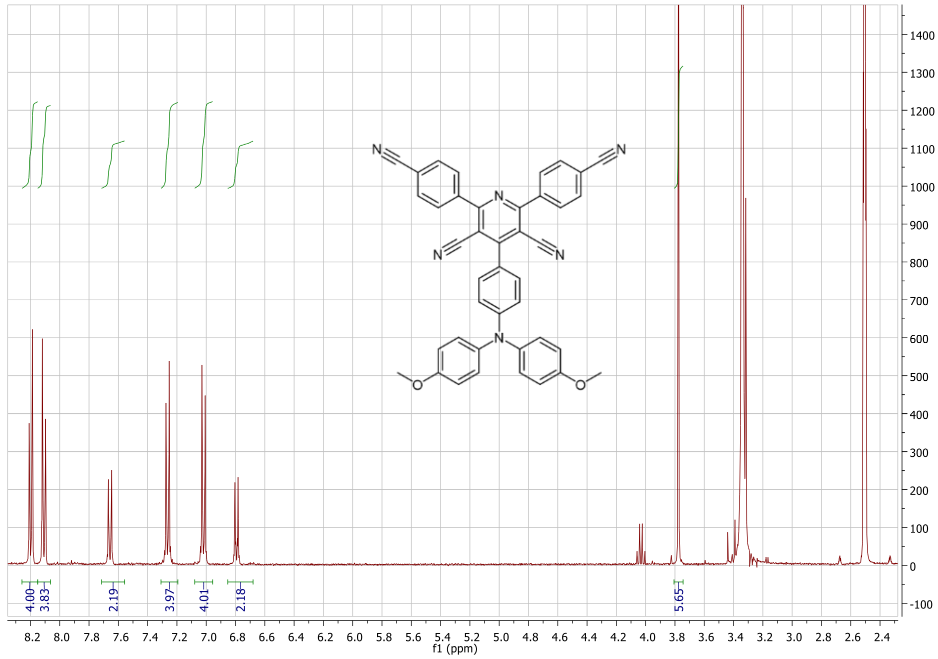

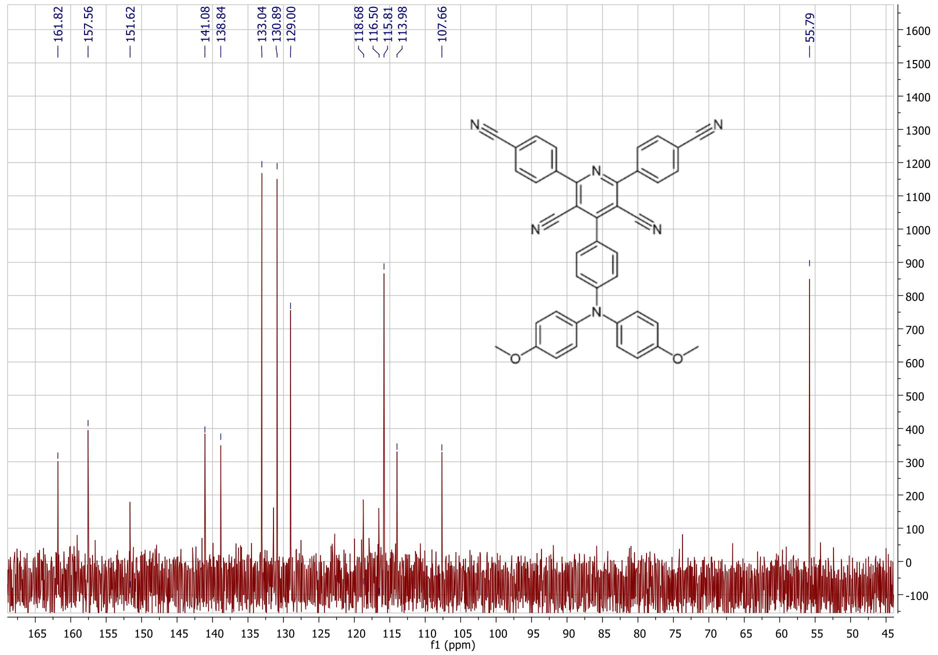

CNP-75

^{1}H NMR (400 MHz, DMSO) δ 8.35 – 8.26 (6H), 8.17 (6H), 8.03 (2H), 7.51 (4H), 7.36 (2H). ^{13}C NMR (101 MHz, DMSO) δ 161.45, 158.84, 140.84, 140.17, 133.20, 131.84, 130.93, 127.25, 127.01, 123.59, 121.19, 121.08, 118.64, 116.05, 114.22, 110.10, 108.39. Chemical Formula: $C_{39}H_{20}N_6$, Elemental Analysis: calcd., C, 81.80; H, 3.52; N, 14.68; found C, 80.85; H, 3.77; N, 14.05. HRMS (m/z): $[M+H]^+$ calcd., 573.1822; found, 573.1764.

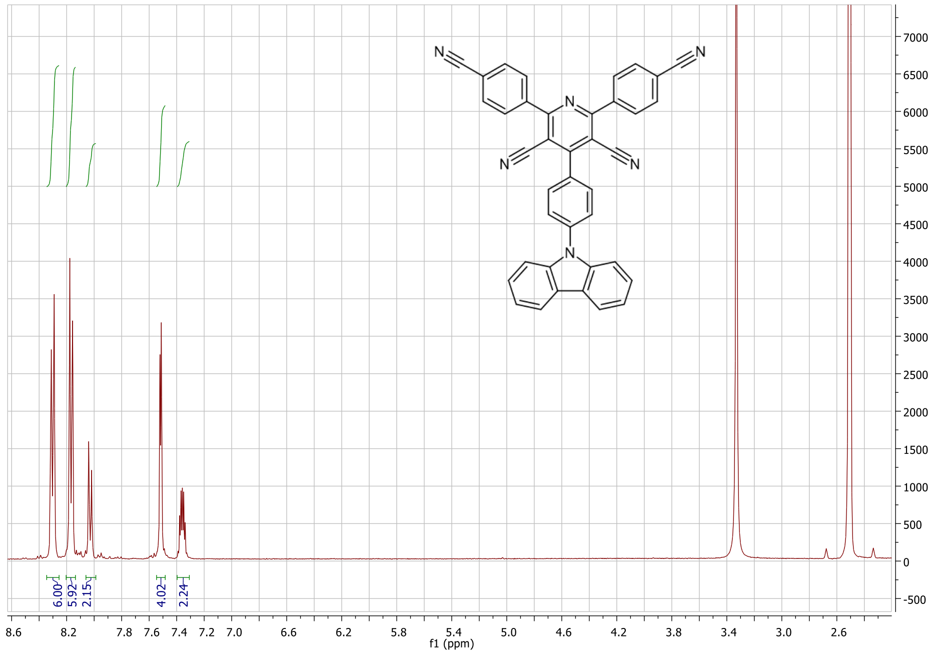

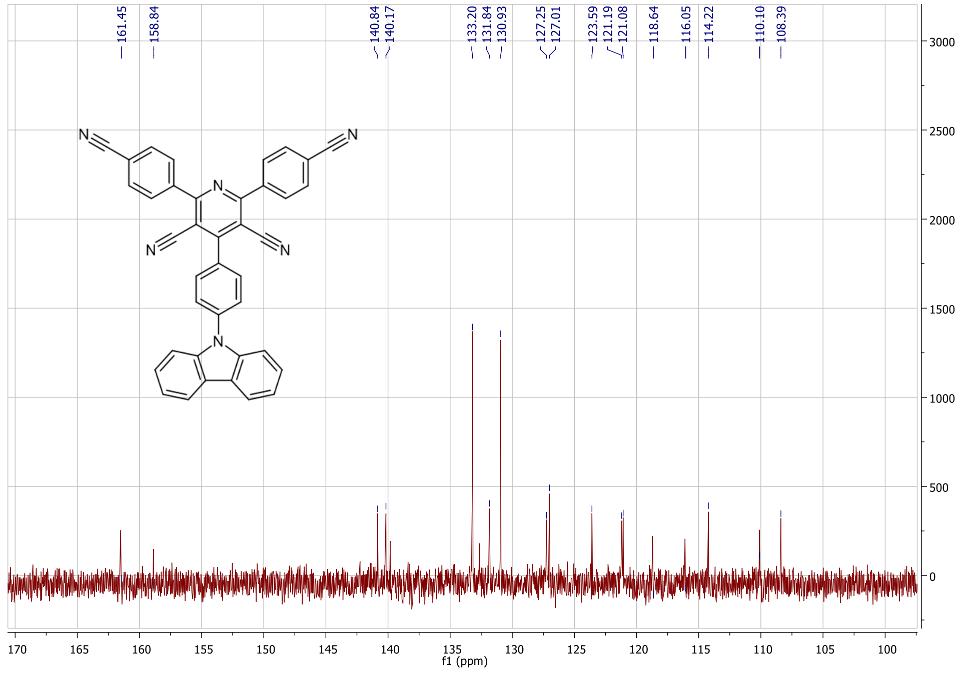

CNP-98

^{1}H NMR (400 MHz, DMSO) δ 8.21 (1H), 8.12 – 7.98 (6H), 7.92 – 7.80 (5H), 7.68 (1H), 7.45 (2H), 4.10 (s, 2H). ^{13}C NMR (101 MHz, DMSO) δ 161.95, 160.06, 144.16, 143.74, 140.45, 136.02, 132.25, 132.04, 128.77, 127.64, 126.53, 125.86, 125.72, 121.38, 120.88, 116.57, 107.16, 37.00. Chemical Formula: $C_{32}H_{17}Br_2N_3$, Elemental Analysis: calcd., C, 63.71; H, 2.84; N, 6.96; found, C, 63.17; H, 2.82; N, 6.88. HRMS (m/z): $[M+H]^+$ calcd., 601.9862; found, 601.9862.

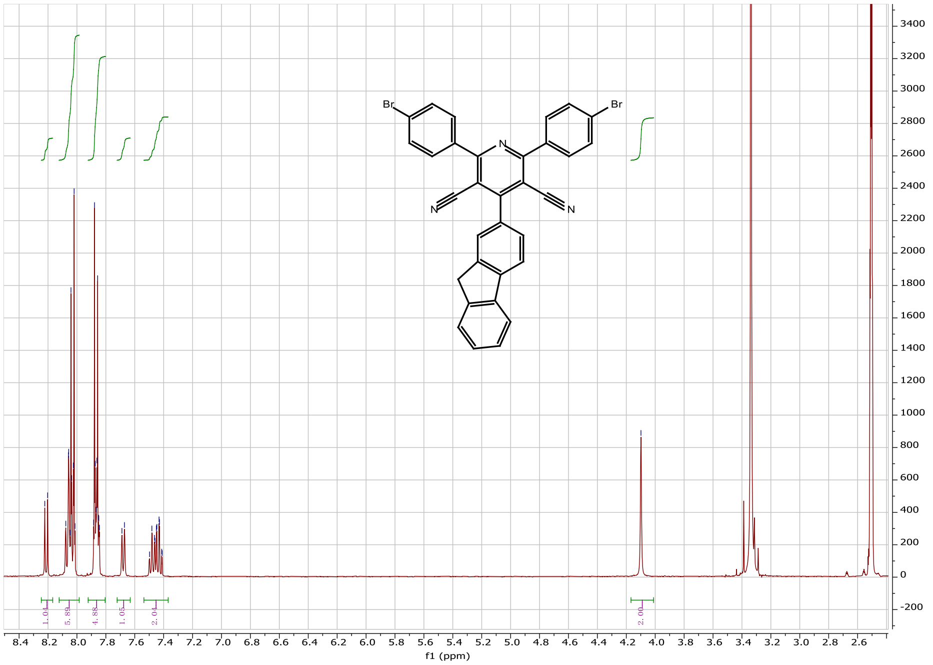

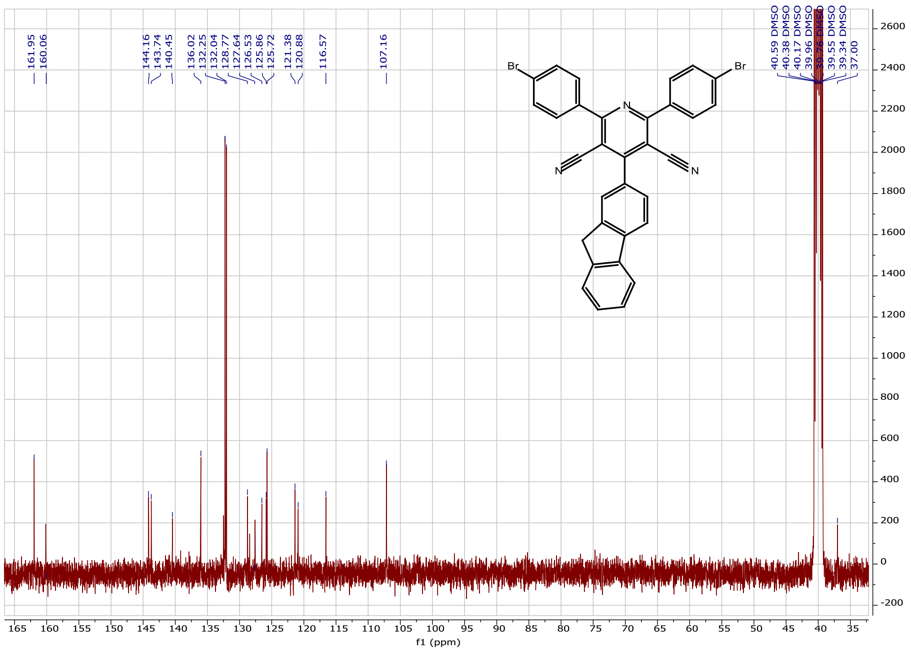

CNP-110

^{1}H NMR (400 MHz, DMSO) δ 8.05 – 8.01 (4H), 8.01 – 7.96 (2H), 7.95 – 7.89 (4H), 7.89 – 7.84 (4H), 7.41 – 7.33 (2H). ^{13}C NMR (101 MHz, DMSO) δ 164.06, 161.95, 159.41, 141.83, 135.97, 135.76, 133.17, 132.27, 132.02, 130.49, 129.62, 129.53, 127.39, 125.75, 116.53, 116.47, 116.32, 107.05. Chemical Formula: $C_{31}H_{16}Br_2FN_3$, Elemental Analysis: calcd., C, 61.11; H, 2.65; N, 6.90; found C, 61.19; H, 2.52; N, 6.89. HRMS (m/z): $[M+H]^+$ calcd., 607.9768; found, 607.9753.

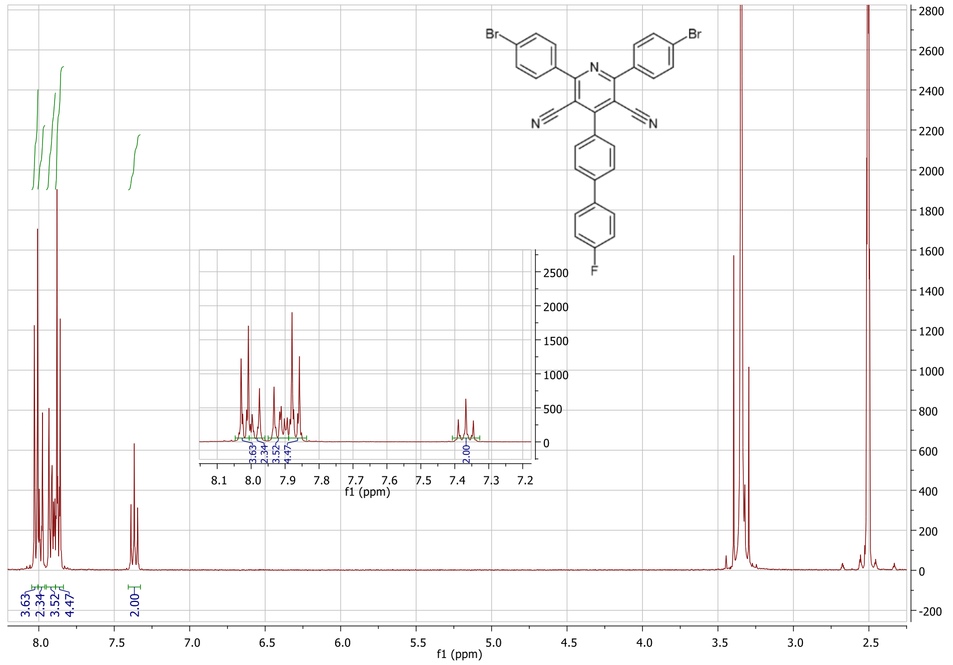

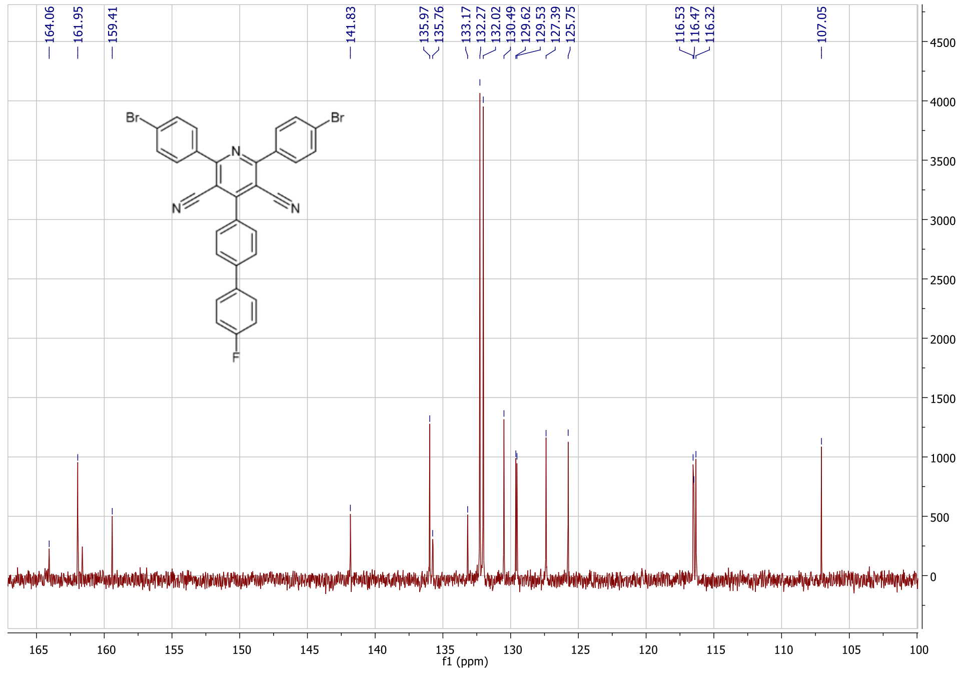

CNP-116

^{1}H NMR (400 MHz, DMSO) δ 7.98 – 7.91 (4H), 7.86 – 7.74 (4H), 7.44 (4H), 4.44 (1H), 2.43 (6H). ^{13}C NMR (101 MHz, DMSO) δ 162.72, 159.18, 141.90, 134.93, 134.12, 132.50, 130.09, 129.92, 129.74, 124.57, 116.60, 105.98, 83.47, 83.13, 21.51. Chemical Formula: $C_{29}H_{19}N_3$, Elemental Analysis: calcd., C, 85.06; H, 4.68; N, 10.26; found, C, 84.68; H, 4.61; N, 10.29. HRMS (m/z): $[M+H]^+$ calcd., 410.1652; found, 410.1653.

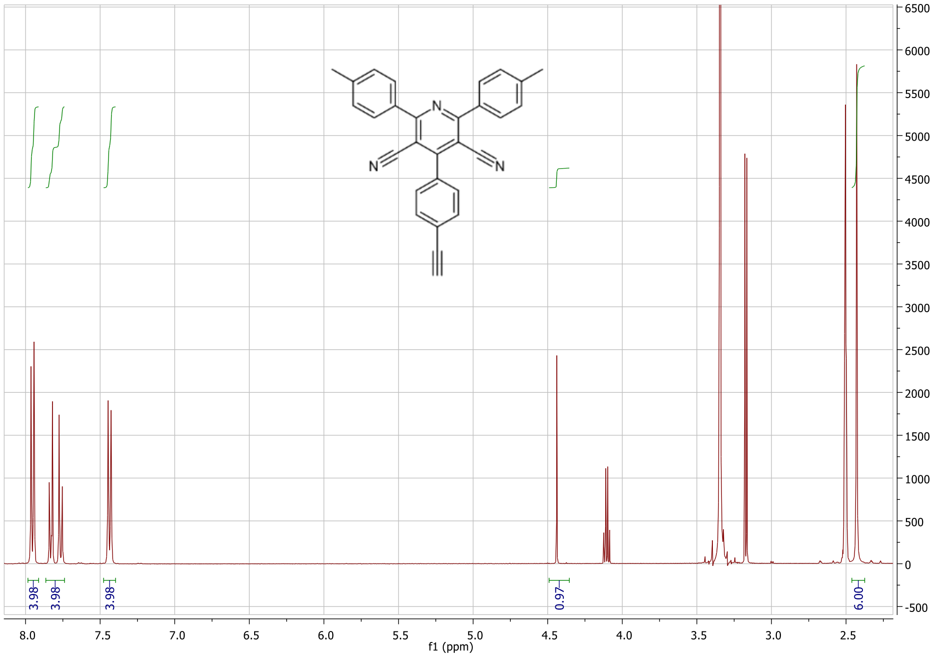

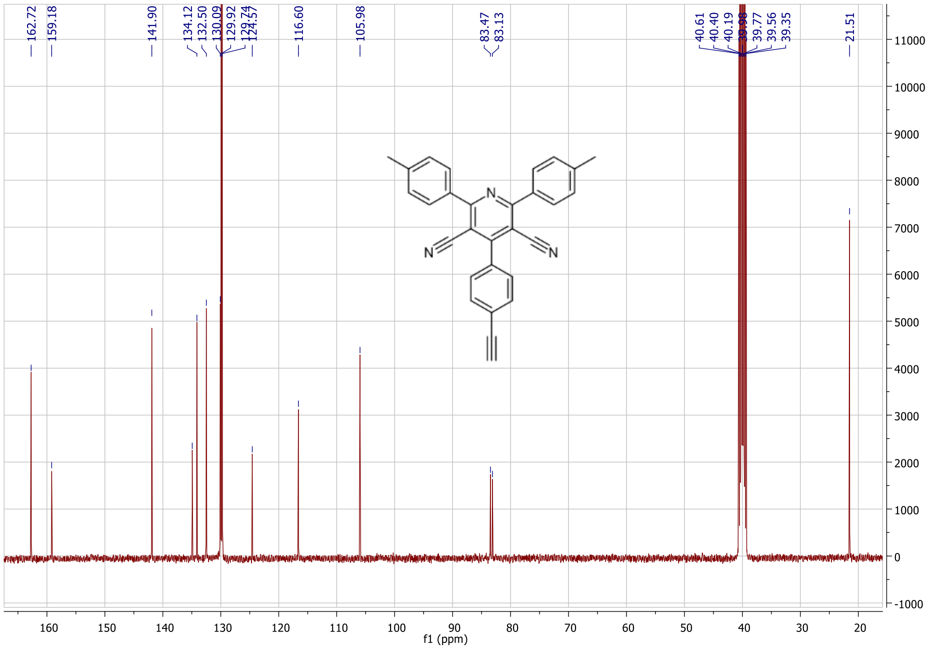

CNP-122

^{1}H NMR (400 MHz, DMSO) δ 8.70 (1H), 8.27 (1H), 8.08 – 7.96 (4H), 7.96 – 7.85 (2H), 7.74 (1H), 7.53 (1H), 7.44 (4H), 7.38 – 7.18 (6H), 5.78 (2H), 2.43 (6H). ^{13}C NMR (101 MHz, DMSO) δ 162.92, 160.72, 141.75, 141.64, 141.22, 137.99, 134.37, 130.00, 129.69, 129.19, 127.93, 127.60, 127.39, 127.23, 125.16, 122.66, 122.54, 121.06, 120.43, 117.24, 110.66, 110.29, 106.44, 46.37, 21.51. Chemical Formula: $C_{40}H_{28}N_4$, Elemental Analysis: calcd., 85.08; H, 5.00; N, 9.92; found, C, 84.98; H, 4.93; N, 9.93. HRMS (m/z): $[M+H]^+$ calcd., 565.2387; found, 565.2390.

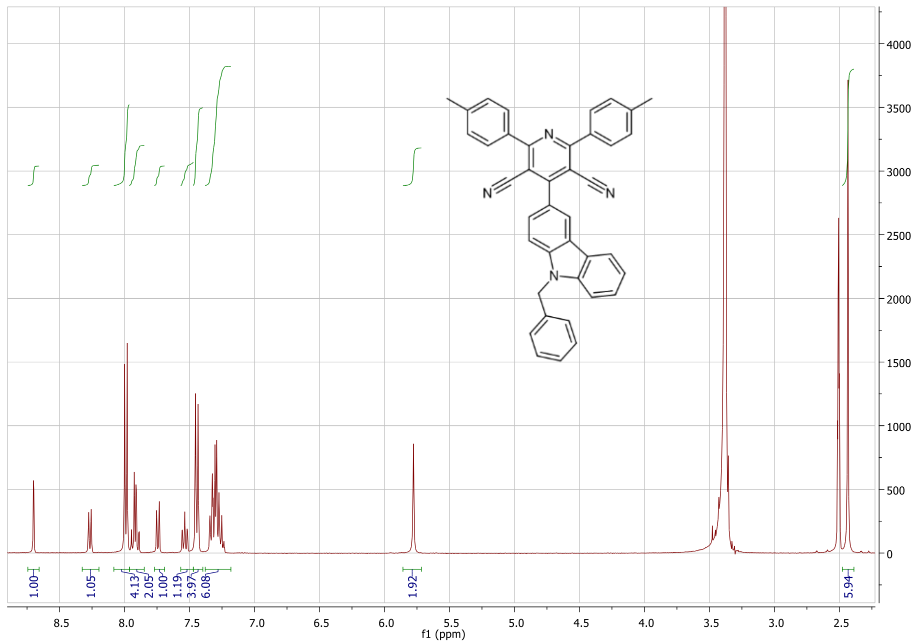

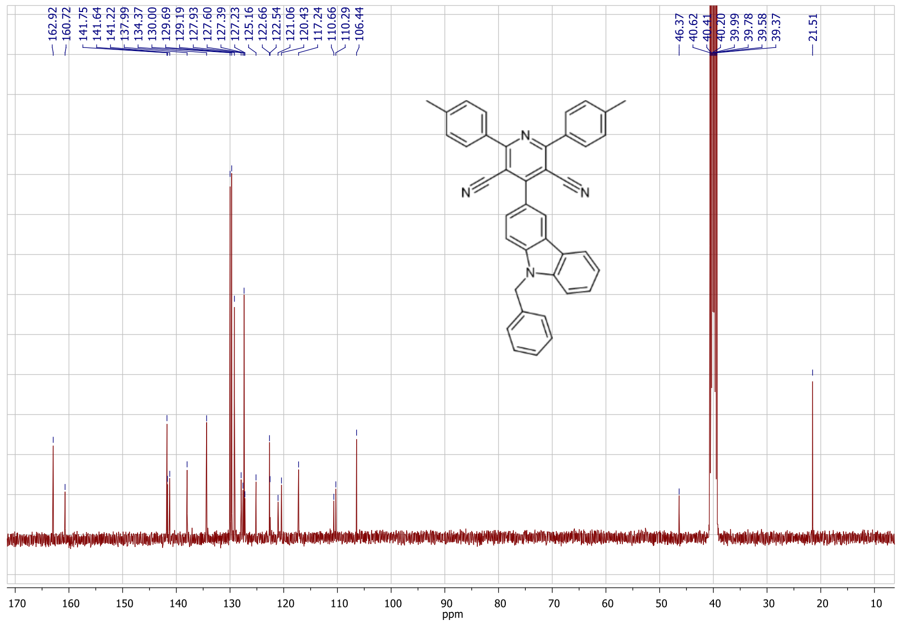

CNP-127

^{1}H NMR (400 MHz, DMSO) δ 8.66 (1H), 8.24 (1H), 8.05 – 7.95 (4H), 7.95 – 7.85 (2H), 7.73 (1H), 7.56 (1H), 7.44 (4H), 7.35 – 7.25 (1H), 4.55 (2H), 2.43 (6H), 1.40 (3H). ^{13}C NMR (101 MHz, DMSO) δ 162.93, 160.85, 141.75, 140.99, 140.57, 134.36, 129.99, 129.68, 127.39, 127.10, 124.80, 122.61, 122.57, 122.44, 121.07, 120.10, 117.27, 110.16, 109.86, 106.44, 21.50, 14.32. Chemical Formula: $C_{35}H_{26}N_4$, Elemental Analysis: calcd., C, 83.64; H, 5.21; N, 11.15; found, C, 83.95; H, 5.19; N, 11.26. HRMS (m/z): $[M+H]^+$ calcd., 503.2230; found, 503.2231.

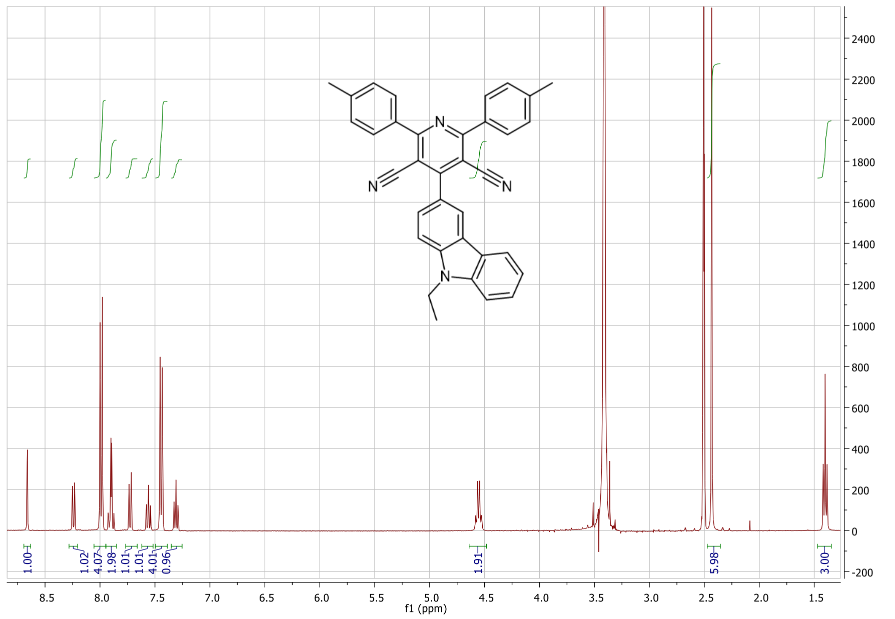

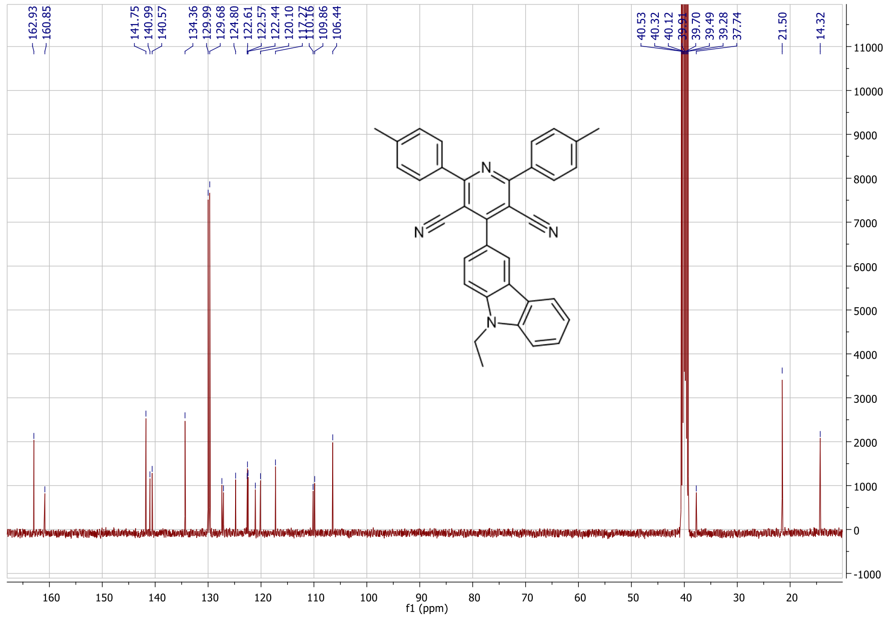

CNP-128

^{1}H NMR (400 MHz, DMSO) δ 8.66 (1H), 8.24 (1H), 7.99 (4H), 7.91 (2H), 7.71 (1H), 7.57 (1H), 7.45 (4H), 7.32 (1H), 3.99 (3H), 2.44 (6H). ^{13}C NMR (101 MHz, DMSO) δ 162.87, 160.92, 142.04, 141.74, 141.69, 134.37, 129.99, 129.69, 127.36, 127.07, 124.85, 122.45, 122.42, 122.28, 120.93, 120.10, 117.23, 110.22, 110.02, 106.49, 29.74, 21.51. Chemical Formula: $C_{34}H_{24}N_4$, Elemental Analysis: calcd., C, 83.58; H, 4.95; N, 11.47; found, C, 83.39; H, 4.89; N, 11.41. HRMS (m/z): $[M+H]^+$ calcd., 489.2074; found, 489.2076.

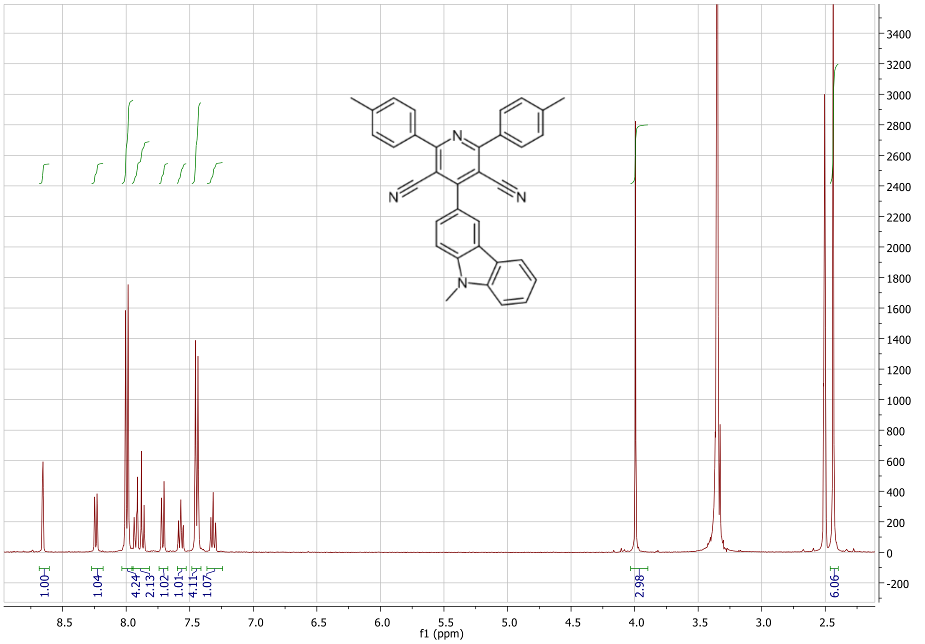

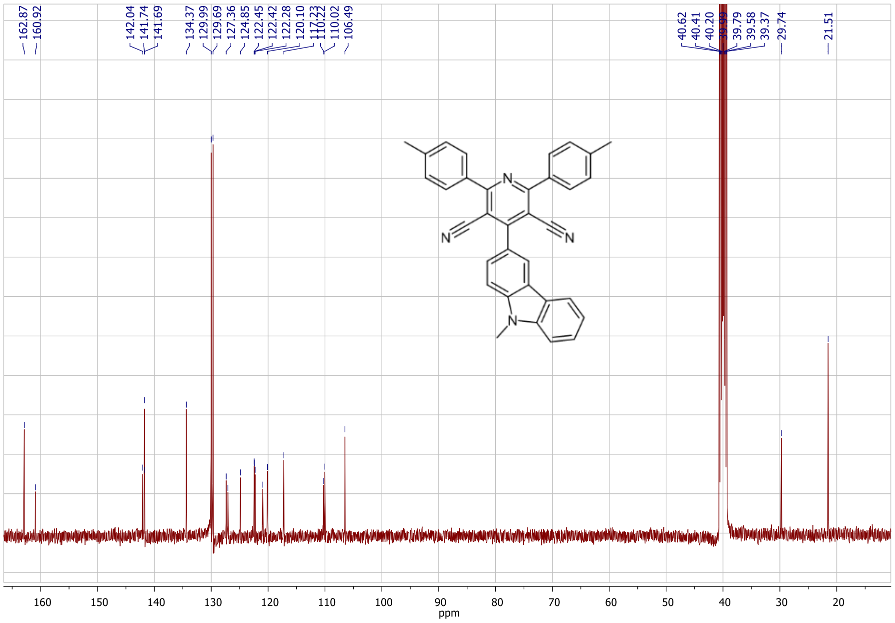

CNP-129

^{1}H NMR (400 MHz, DMSO) δ 8.80 – 8.75 (1H), 8.34 (1H), 8.04 – 7.97 (4H), 7.88 (1H), 7.78 – 7.69 (4H), 7.65 – 7.57 (2H), 7.54 (1H), 7.49 – 7.36 (6H), 2.44 (6H). ^{13}C NMR (101 MHz, DMSO) δ 162.93, 160.47, 141.79, 141.59, 141.35, 136.72, 134.32, 130.84, 129.98, 129.70, 128.74, 127.90, 127.68, 127.43, 126.21, 123.19, 122.85, 121.34, 117.18, 110.61, 110.32, 106.46, 21.50. Chemical Formula: $C_{39}H_{26}N_4$, Elemental Analysis: calcd., C, 85.07; H, 4.76; N, 10.17; found C, 84.91; H, 4.93; N, 10.16. HRMS (m/z): $[M+H]^+$ calcd., 551.2230; found, 551.2224.

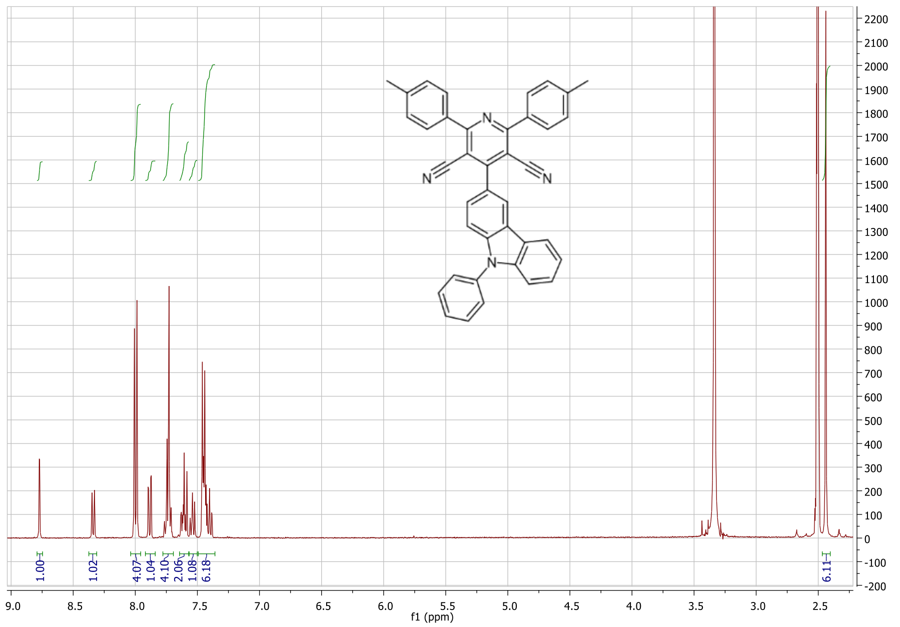

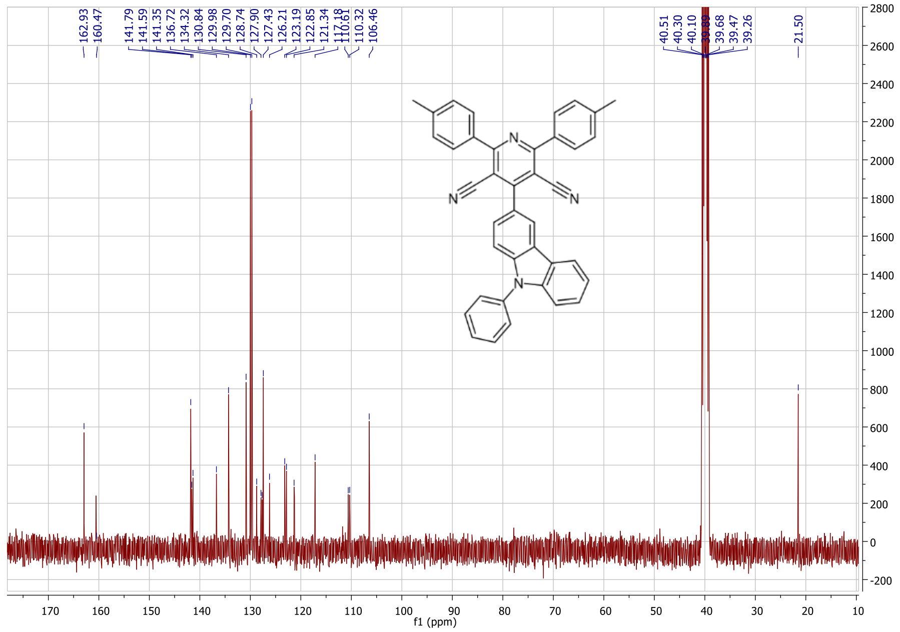

CNP-130

^{1}H NMR (400 MHz, $CDCl_3$) δ 7.92 – 7.83 (4H), 7.43 – 7.33 (2H), 7.28 (4H), 7.15 – 7.05 (4H), 6.98 – 6.88 (2H), 6.87 – 6.74 (4H), 3.75 (6H), 2.37 (6H). ^{13}C NMR (101 MHz, $CDCl_3$) δ 163.49, 160.12, 156.96, 151.21, 141.70, 139.38, 134.04, 130.40, 129.57, 129.41, 128.02, 123.40, 117.41, 116.92, 115.00, 104.82, 55.52, 21.55. Chemical Formula: $C_{41}H_{32}N_4O_2$, Elemental Analysis: calcd., C, 80.37; H, 5.26; N, 9.14; found, C, 79.68; H, 5.14; N, 9.09. HRMS (m/z): $[M+H]^+$ calcd., 613.2598; found, 613.2601.

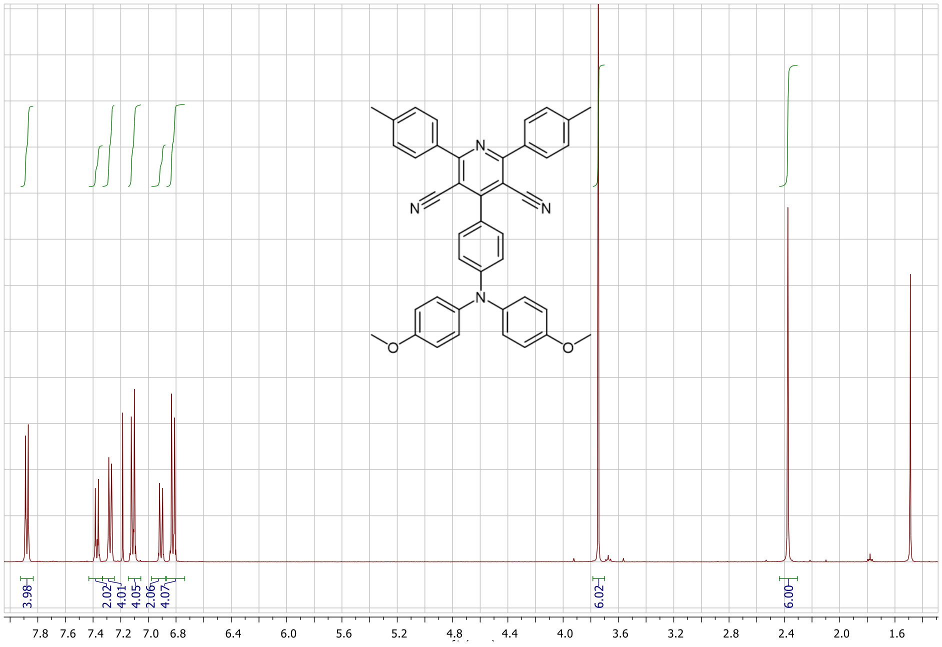

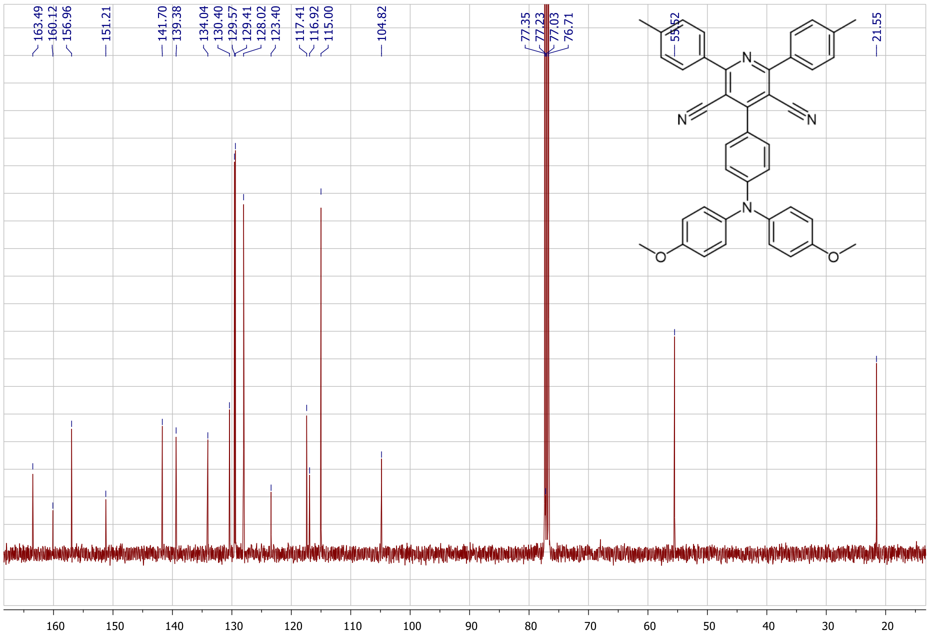

CNP-131

^{1}H NMR (400 MHz, DMSO) δ 8.30 (1H), 8.17 – 8.10 (1H), 8.03 – 7.99 (2H), 7.99 – 7.95 (1H), 7.54 – 7.48 (2H), 7.46 (2H), 7.39 – 7.31 (1H), 2.45 (3H). ^{13}C NMR (101 MHz, $CDCl_3$) δ 163.39, 159.47, 142.19, 140.39, 140.33, 133.68, 132.24, 130.85, 129.61, 129.59, 127.22, 126.25, 123.80, 120.57, 120.41, 116.24, 109.95, 105.10, 21.60. Chemical Formula: $C_{39}H_{26}N_4$, Elemental Analysis: calcd., C, 85.07; H, 4.76; N, 10.17; found C, 84.86; H, 4.72; N, 10.26. HRMS (m/z): $[M+H]^+$ calcd., 551.2230; found, 551.2224

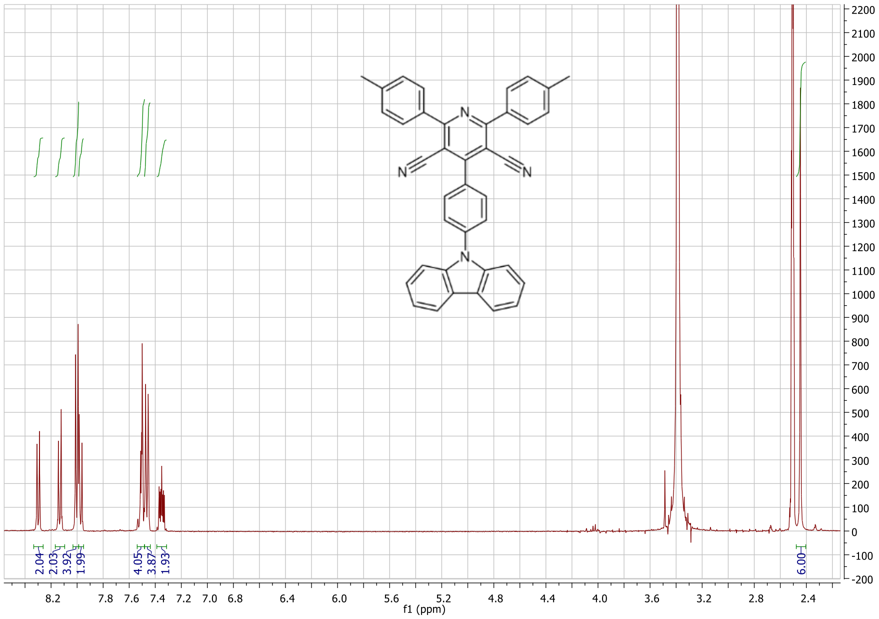

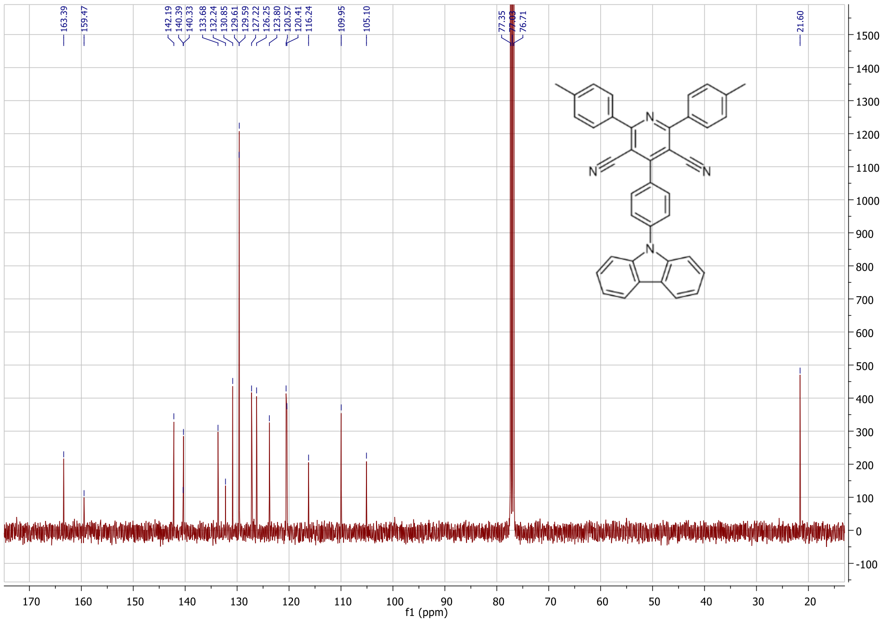

CNP-132

^{1}H NMR (400 MHz, DMSO) δ 8.55 (1H), 8.45 (2H), 8.40 (1H), 8.37 – 8.29 (3H), 8.20 (1H), 8.08 (1H), 8.04 (4H), 7.46 (4H), 2.44 (6H). ^{13}C NMR (101 MHz, DMSO) δ 162.73, 159.77, 141.89, 134.21, 132.71, 131.20, 130.73, 130.00, 129.77, 129.46, 129.16, 128.59, 127.73, 127.56, 127.43, 126.87, 126.63, 125.43, 124.33, 124.12, 124.00, 116.56, 107.56, 21.53. Chemical Formula: $C_{37}H_{23}N_3$, Elemental Analysis: calcd., C, 87.20; H, 4.55; N, 8.25; found, C, 86.88; H, 4.48; N, 8.33. HRMS (m/z): $[M+H]^+$ calcd., 510.1965; found, 510.1948.

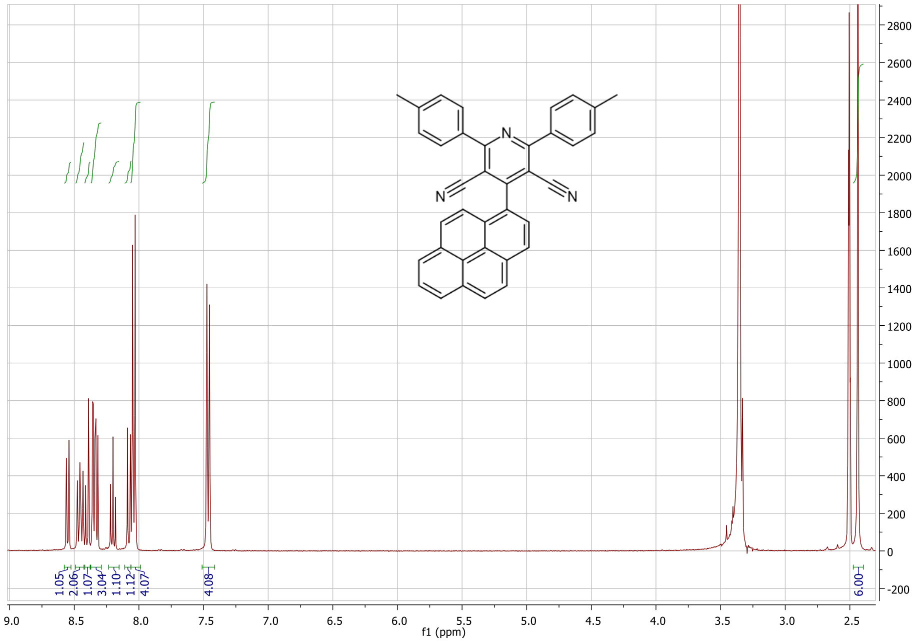

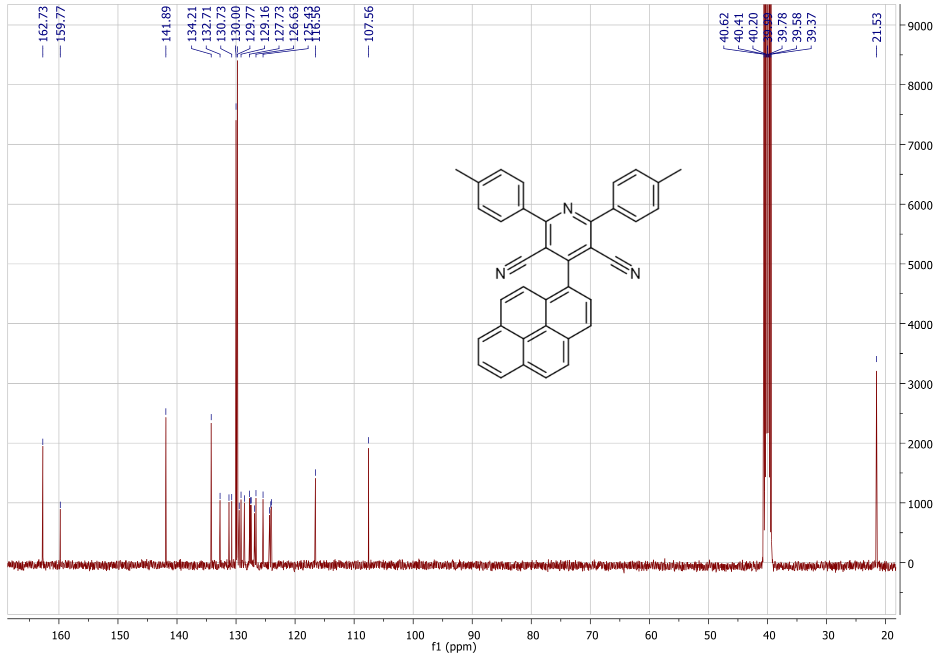

CNP-135

^{1}H NMR (400 MHz, DMSO) δ 8.01 – 7.93 (6H), 7.92 – 7.86 (2H), 7.74 (2H), 7.44 (4H), 7.35 (2H), 2.44 (6H), 2.39 (3H). ^{13}C NMR (101 MHz, DMSO) δ 162.87, 159.64, 142.61, 141.82, 138.19, 136.43, 134.24, 133.20, 130.43, 130.20, 129.96, 129.72, 127.24, 127.03, 116.84, 106.09, 40.62, 21.51, 21.20. Chemical Formula: $C_{34}H_{25}N_{3}$, Elemental Analysis: calcd., C, 85.87; H, 5.30; N, 8.84; found, C, 85.73; H, 5.24; N, 8.87. HRMS (m/z): $[M+H]^{+}$ calcd., 476.2121; found, 476.2123.

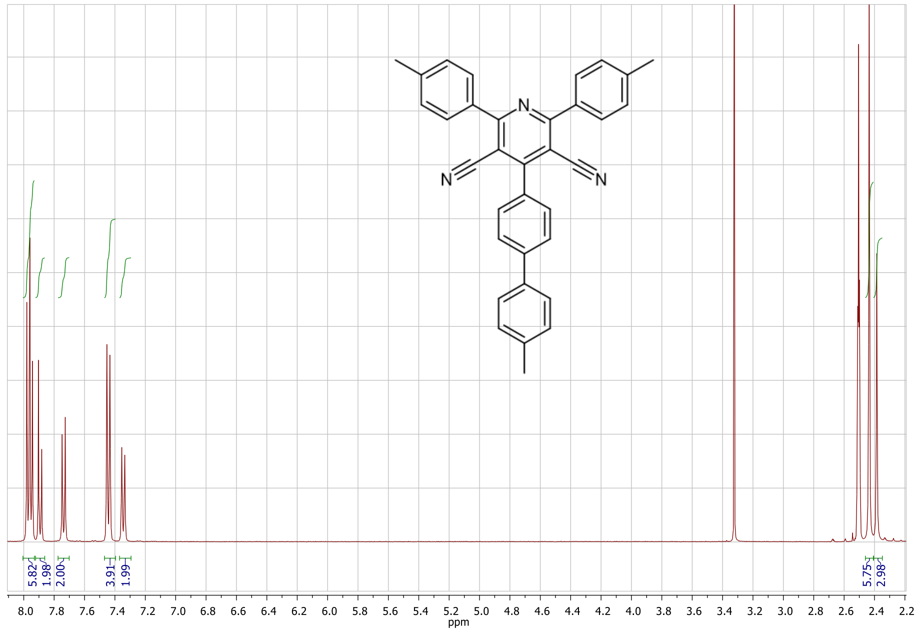

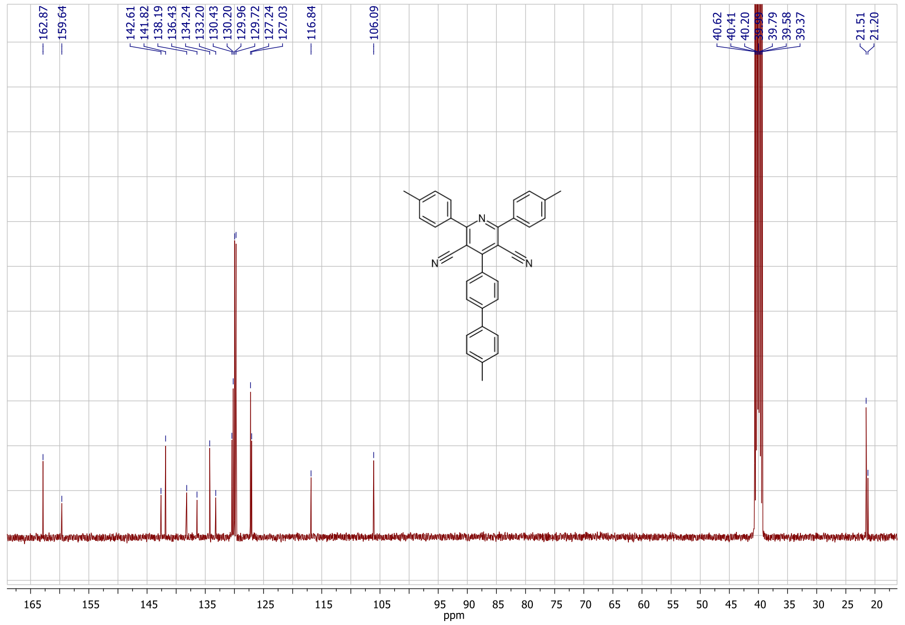

CNP-137

^1H NMR (400 MHz, $CDCl_3$) δ 7.88 (d, *J* = 8.2 Hz, 2H), 7.38 (d, *J* = 8.9 Hz, 1H), 7.28 (d, *J* = 8.0 Hz, 2H), 7.10 – 6.99 (m, 5H), 2.37 (d, *J* = 7.7 Hz, 3H), 2.28 (d, *J* = 4.1 Hz, 3H). Chemical Formula: $C_{41}H_{32}N_4$, Elemental Analysis: calcd., C, 84.80; H, 5.55; N, 9.65; found C, 82.75; H, 5.69; N, 8.95. HRMS (m/z): $[M+H]^+$ calcd., 580.2627; found, 580.2617.

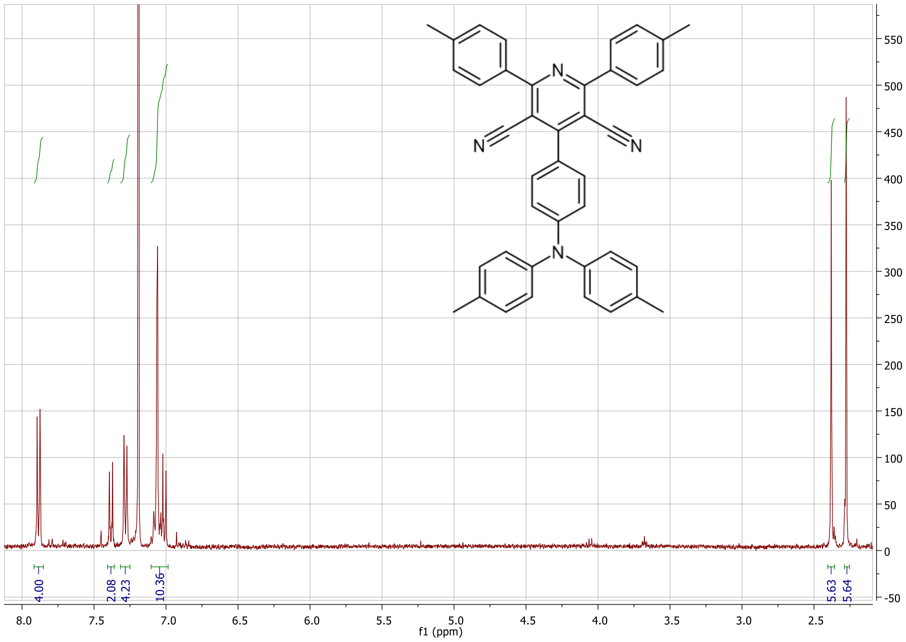

CNP-138

^{1}H NMR (400 MHz, DMSO) δ 7.97 (6H), 7.93 – 7.87 (4H), 7.47 – 7.41 (4H), 7.40 – 7.33 (2H), 2.44 (6H). ^{13}C NMR (101 MHz, DMSO) δ 162.85, 161.59, 159.56, 141.83, 141.63, 135.84, 135.80, 134.23, 133.52, 130.48, 129.95, 129.72, 129.59, 129.51, 127.31, 116.81, 116.52, 116.31, 106.10, 21.51. Chemical Formula: $C_{33}H_{22}FN_3$, Elemental Analysis: calcd., C, 82.65; H, 4.62; N, 8.76; found, C, 82.97; H, 4.58; N, 8.88. HRMS (m/z): $[M+H]^+$ calcd., 480.1871; found, 480.1872.

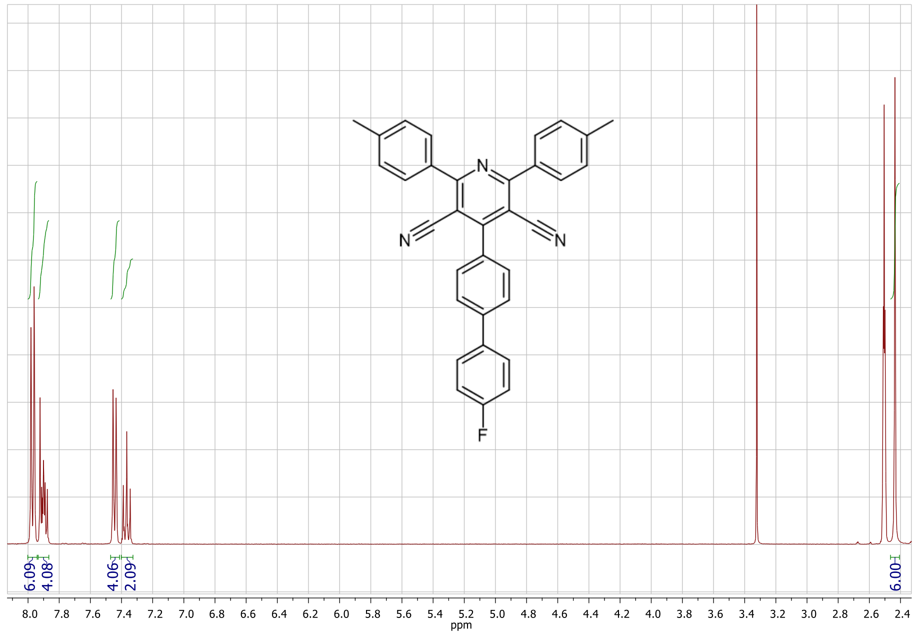

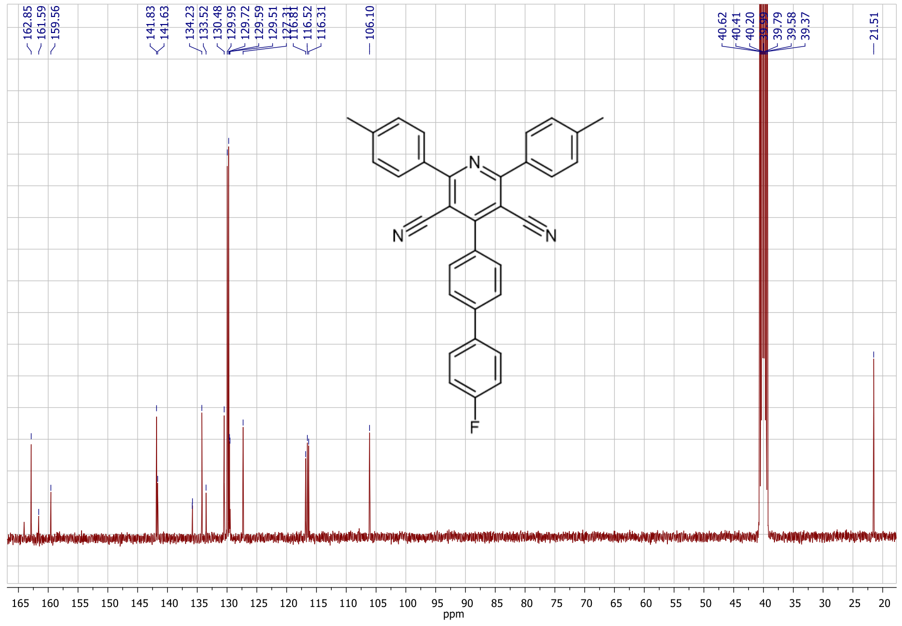

CNP-145

^{1}H NMR (400 MHz, DMSO) δ 8.41 (1H), 8.22 (1H), 8.20 – 8.13 (4H), 8.10 (2H), 7.91 (1H), 7.76 – 7.63 (2H), 7.56 – 7.42 (4H). ^{13}C NMR (101 MHz, DMSO) δ 165.61, 163.13, 161.82, 159.81, 133.99, 133.37, 133.34, 132.70, 132.61, 131.87, 129.91, 129.04, 128.52, 128.39, 127.77, 126.34, 116.61, 116.43, 116.21, 106.93. Chemical Formula: $C_{29}H_{15}F_2N_3$, Elemental Analysis: calcd., C, 78.55; H, 3.41; N, 9.48; found, C, 78.51; H, 3.39; N, 9.56. HRMS (m/z): [M+H]$^+$ calcd., 444.1307; found, 444.1312.

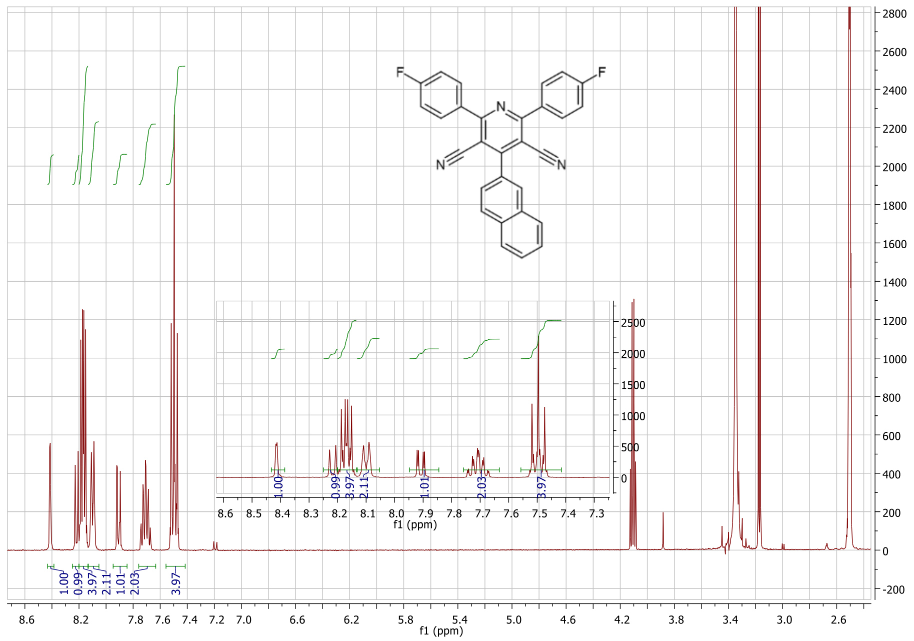

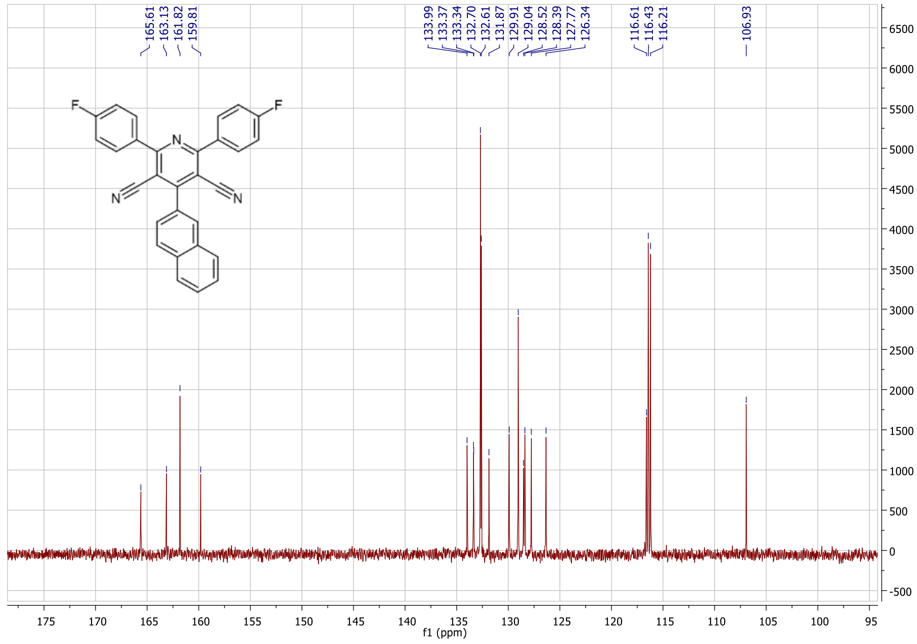

CNP-153

^{1}H NMR (400 MHz, DMSO) δ 8.22 – 8.14 (4H), 8.08 – 7.97 (4H), 7.85 (1H), 7.83 – 7.77 (2H), 7.68 – 7.62 (1H), 7.63 – 7.55 (3H), 7.54 – 7.45 (4H). ^{13}C NMR (101 MHz, DMSO) δ 165.62, 163.14, 161.91, 159.50, 142.92, 138.81, 133.94, 133.42, 133.39, 132.73, 132.64, 131.06, 130.62, 130.00, 129.03, 128.75, 127.73, 127.21, 126.61, 126.13, 125.30, 116.64, 116.44, 116.22, 106.81. Chemical Formula: $C_{35}H_{19}F_{2}N_{3}$, Elemental Analysis: calcd., C, 80.91; H, 3.69; N, 8.09; found, C, 80.48; H, 3.62; N, 8.11. HRMS (m/z): $[M+H]^{+}$ calcd., 520.1620; found, 520.1600.

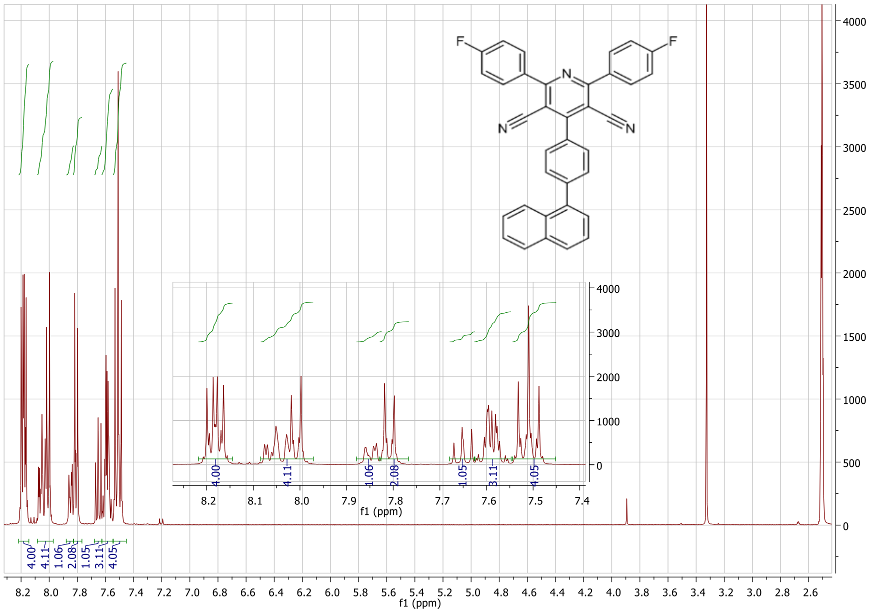

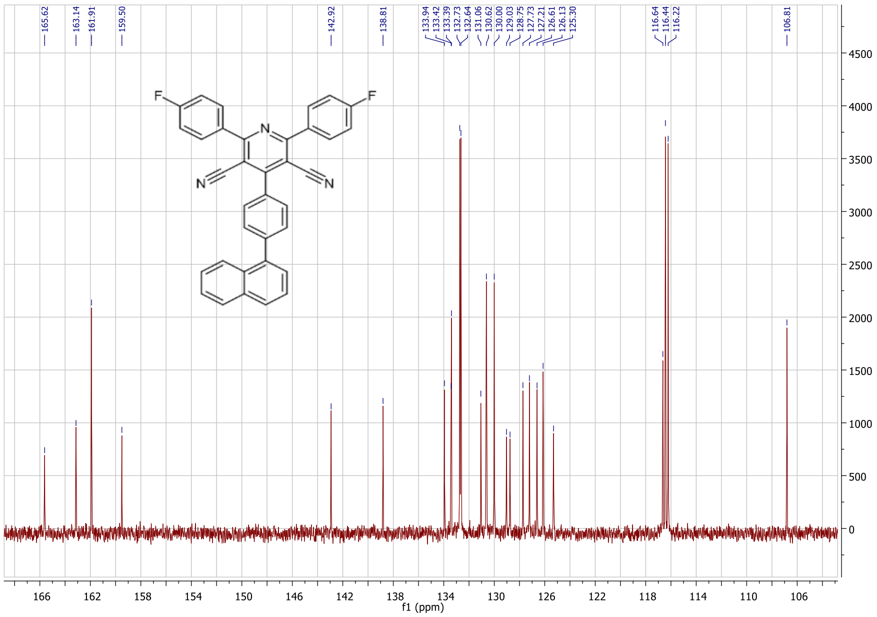

CNP-156

^{1}H NMR (400 MHz, DMSO) δ 8.70 – 8.65 (1H), 8.23 (1H), 8.21 – 8.14 (4H), 7.95 (1H), 7.89 (d, J = 8.3 Hz, 1H), 7.72 (1H), 7.61 – 7.54 (1H), 7.54 – 7.43 (4H), 7.32 (1H), 4.00 (3H). ^{13}C NMR (101 MHz, DMSO) δ 163.05, 161.89, 142.02, 141.56, 133.43, 132.72, 132.63, 127.22, 127.10, 124.74, 122.44, 120.85, 120.22, 120.16, 117.03, 116.36, 116.14, 110.09, 107.09, 29.75. Chemical Formula: $C_{32}H_{18}F_{2}N_{4}$, Elemental Analysis: calcd., C, 77.41; H, 3.65; N, 11.28; found, C, 77.01; H, 3.61; N, 11.13. HRMS (m/z): $[M+H]^{+}$ calcd., 497.1572; found, 497.1572.

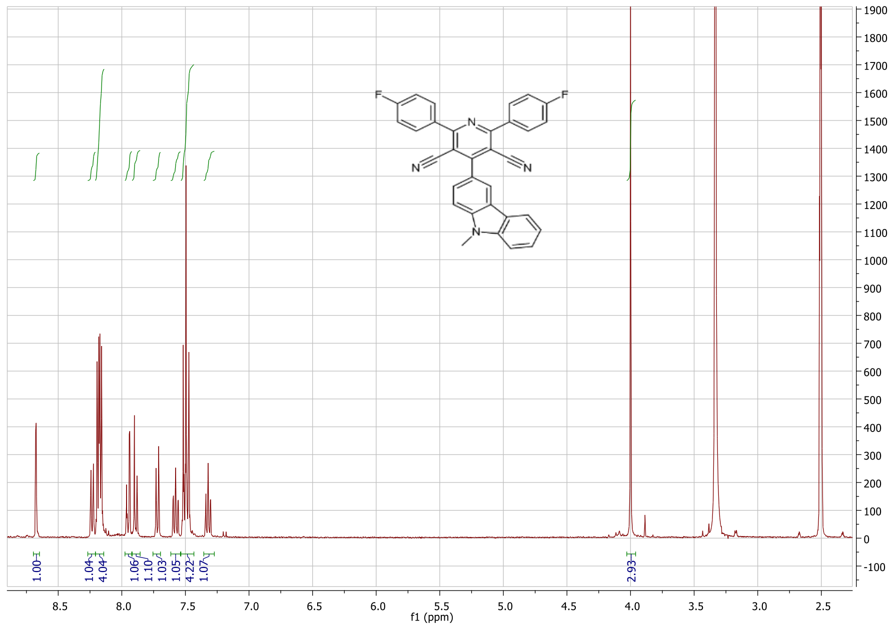

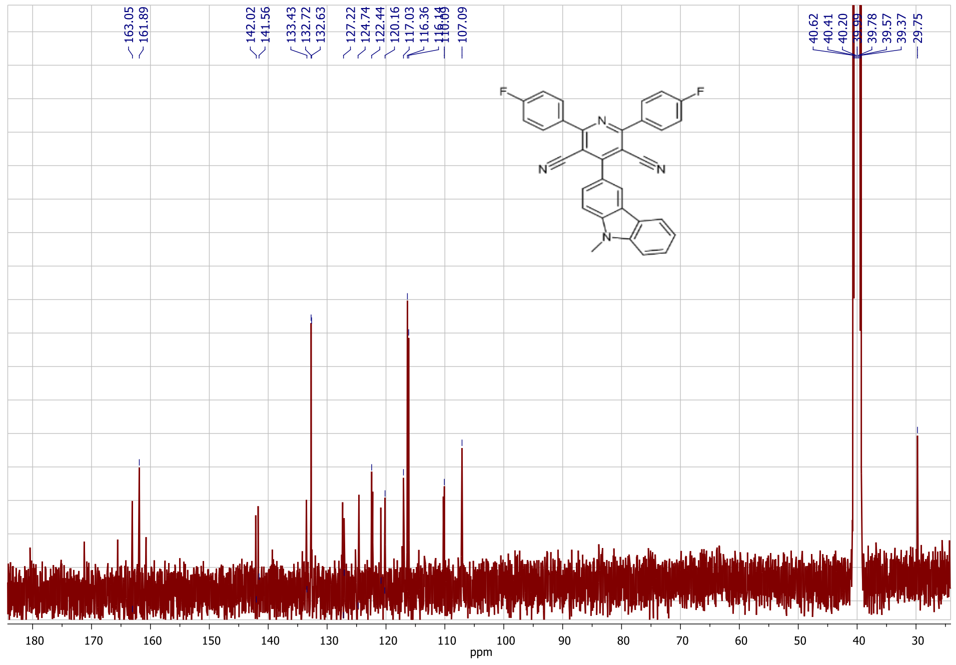

CNP-168

^{1}H NMR (400 MHz, DMSO) δ 8.29 (4H), 8.25 (1H), 8.15 (1H), 8.05 (4H), 7.88 – 7.75 (3H), 7.66 (2H). ^{13}C NMR (101 MHz, DMSO) δ 161.71, 159.24, 140.54, 133.50, 131.70, 131.50, 131.36, 130.96, 130.32, 129.15, 128.27, 128.17, 127.40, 126.20, 126.16, 125.95, 125.74, 125.30, 123.03, 115.75, 109.31. Chemical Formula: $C_{31}H_{15}F_6N_3$, Elemental Analysis: calcd., C, 68.51; H, 2.78; N, 7.73; found, C, 67.90; H, 2.79; N, 7.61. HRMS (m/z): $[M+H]^+$ calcd., 544.1243; found, 544.1240.

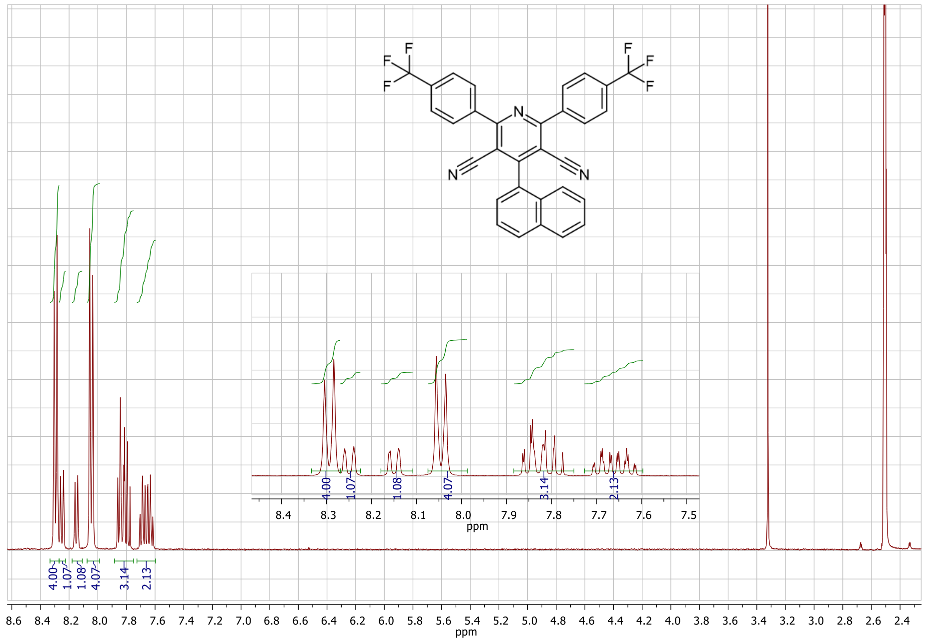

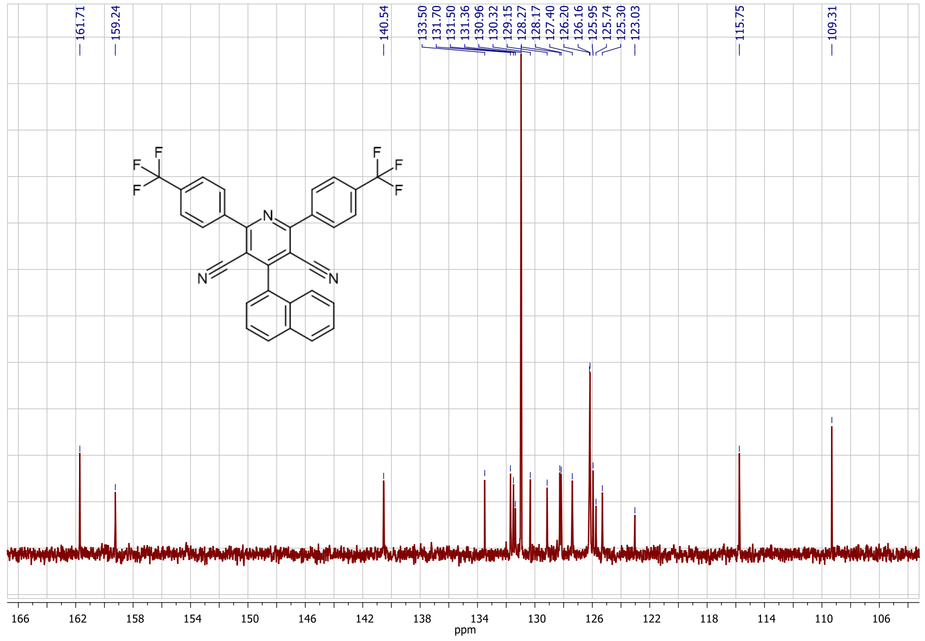

CNP-187

^{1}H NMR (400 MHz, DMSO) δ 8.31 (6H), 8.21 – 8.14 (2H), 8.10 – 8.00 (6H), 7.56 – 7.47 (4H), 7.40 – 7.30 (2H). ^{13}C NMR (101 MHz, DMSO) δ 161.80, 158.92, 140.59, 140.17, 139.78, 132.75, 131.84, 131.76, 131.44, 130.98, 127.24, 127.01, 126.24, 126.20, 125.75, 123.59, 123.04, 121.19, 121.07, 116.21, 110.12, 108.25. Chemical Formula: $C_{39}H_{20}F_6N_4$, Elemental Analysis: calcd., C, 71.12; H, 3.06; N, 8.51; found, C, 71.02; H, 3.03; N, 8.49. HRMS (m/z): $[M+H]^+$ calcd., 659.1665; found, 659.1647.

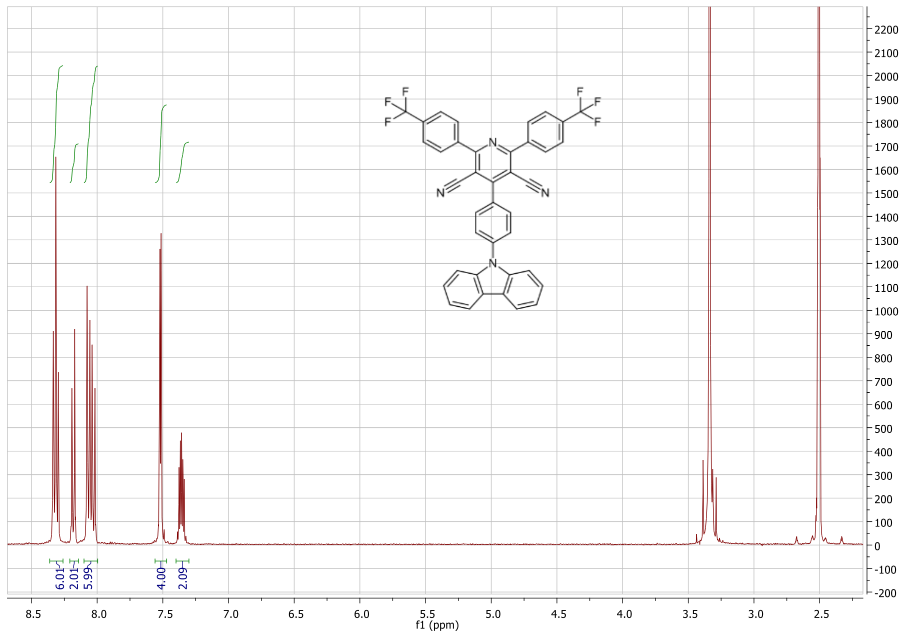

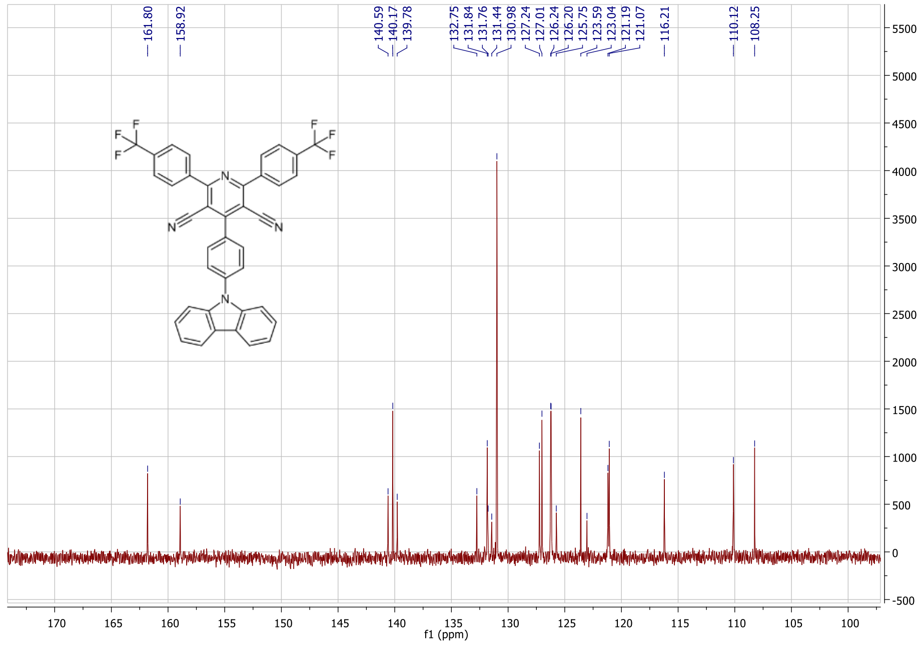

CNP-202

^{1}H NMR (400 MHz, DMSO) δ 8.27 – 8.19 (5H), 8.02 – 7.93 (5H), 7.92 – 7.86 (1H), 7.85 – 7.75 (7H), 7.60 – 7.50 (6H), 7.48 – 7.40 (3H). ^{13}C NMR (101 MHz, DMSO) δ 162.49, 159.79, 143.32, 141.03, 139.64, 139.45, 135.87, 135.07, 130.73, 130.00, 129.61, 129.60, 129.51, 128.77, 128.55, 128.36, 127.47, 127.39, 116.87, 106.55. Chemical Formula: $C_{43}H_{27}N_3$, Elemental Analysis: calcd., C, 88.18; H, 4.65; N, 7.17; found, C, 88.08; H, 4.63; N, 7.22. HRMS (m/z): $[M+H]^+$ calcd., 586.2278; found, 586.2278.

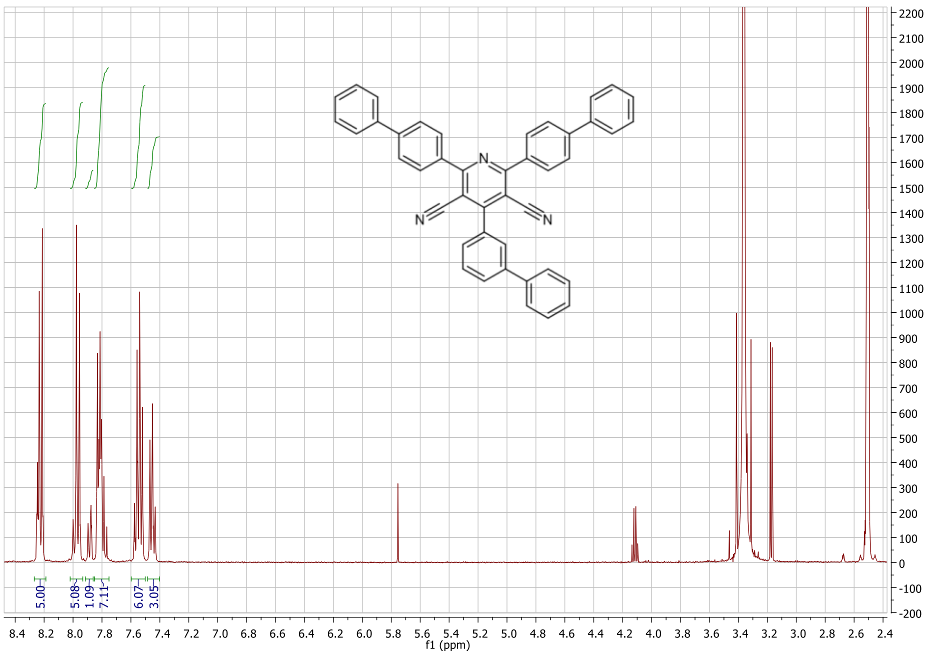

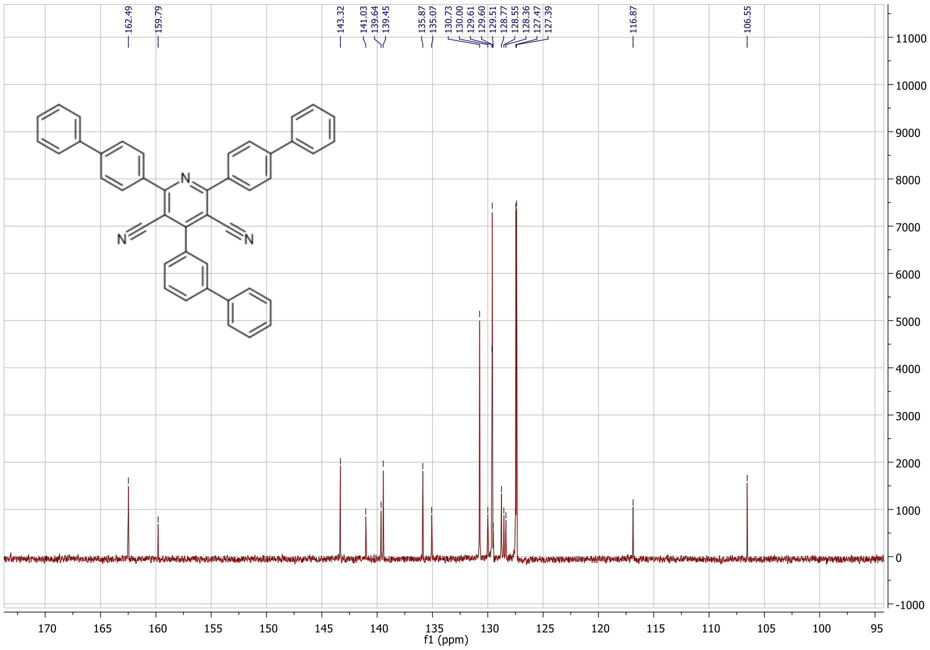

CNP-207

^{1}H NMR (400 MHz, DMSO) δ 8.24 – 8.17 (4H), 7.99 – 7.95 (4H), 7.95 – 7.92 (2H), 7.91 – 7.85 (m, 2H), 7.83 (4H), 7.63 (2H), 7.54 (4H), 7.51 – 7.41 (m, 5H). ^{13}C NMR (101 MHz, DMSO) δ 162.37, 159.29, 143.35, 139.42, 135.78, 134.59, 132.18, 132.04, 130.69, 130.28, 129.60, 129.35, 128.78, 127.47, 127.42, 125.26, 122.33, 116.63, 106.36, 91.92, 88.96. Chemical Formula: $C_{45}H_{27}N_3$, Elemental Analysis: calcd., C, 88.64; H, 4.46; N, 6.89; found C, 88.11; H, 4.41; N, 6.91. HRMS (m/z): $[M+H]^+$ calcd., 610.2278; found, 610.2230.

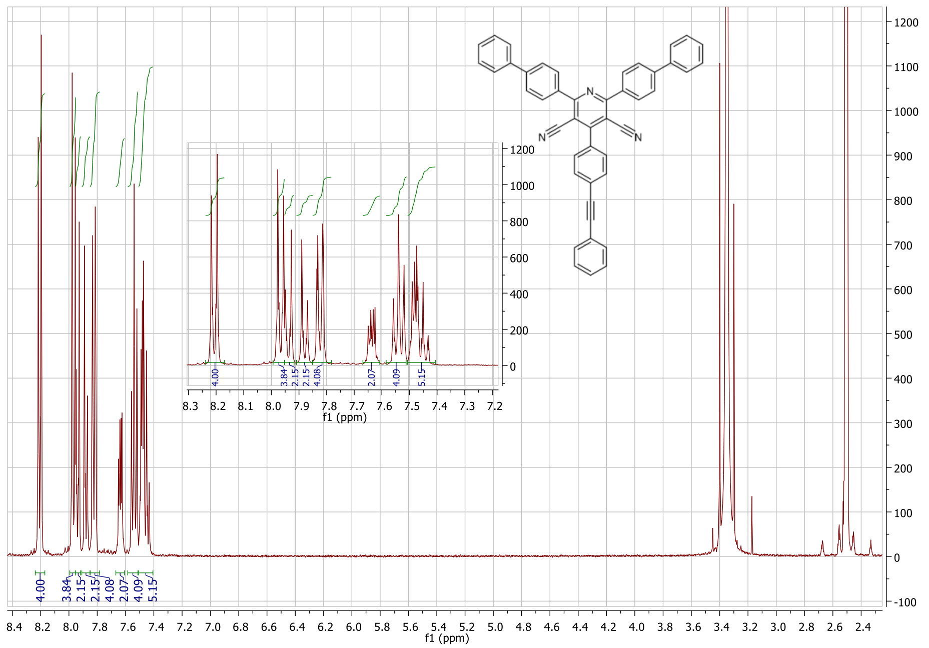

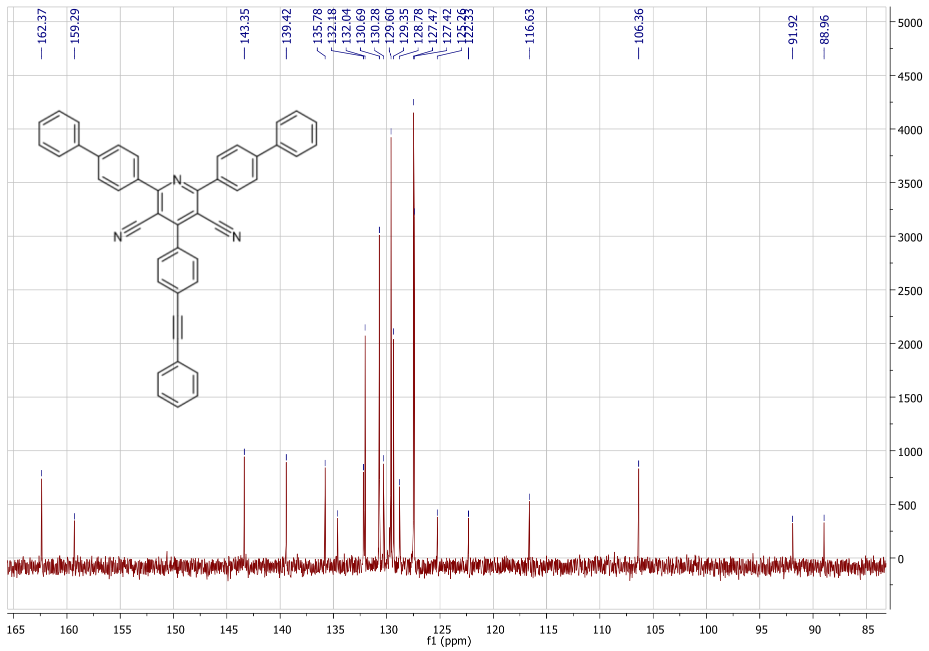

CNP-234

^{1}H NMR (400 MHz, DMSO) δ 8.76 – 8.62 (1H), 8.27 (1H), 8.19 – 8.00 (4H), 8.01 – 7.86 (2H), 7.74 (1H), 7.60 – 7.48 (1H), 7.41 – 7.23 (6H), 7.23 – 7.11 (4H), 3.88 (6H). ^{13}C NMR (101 MHz, DMSO) δ 162.25, 162.20, 160.90, 141.59, 141.19, 137.96, 131.86, 129.35, 129.19, 127.96, 127.60, 127.37, 127.24, 125.33, 122.65, 122.53, 121.09, 120.42, 117.53, 114.57, 110.62, 110.23, 105.30, 55.99, 46.36. Chemical Formula: $C_{40}H_{28}N_4O_2$, Elemental Analysis: calcd., C, 80.52; H, 4.73; N, 9.39; found, C, 79.61; H, 4.62; N, 9.33. HRMS (m/z): $[M+H]^+$ calcd., 597.2285; found, 597.2289.

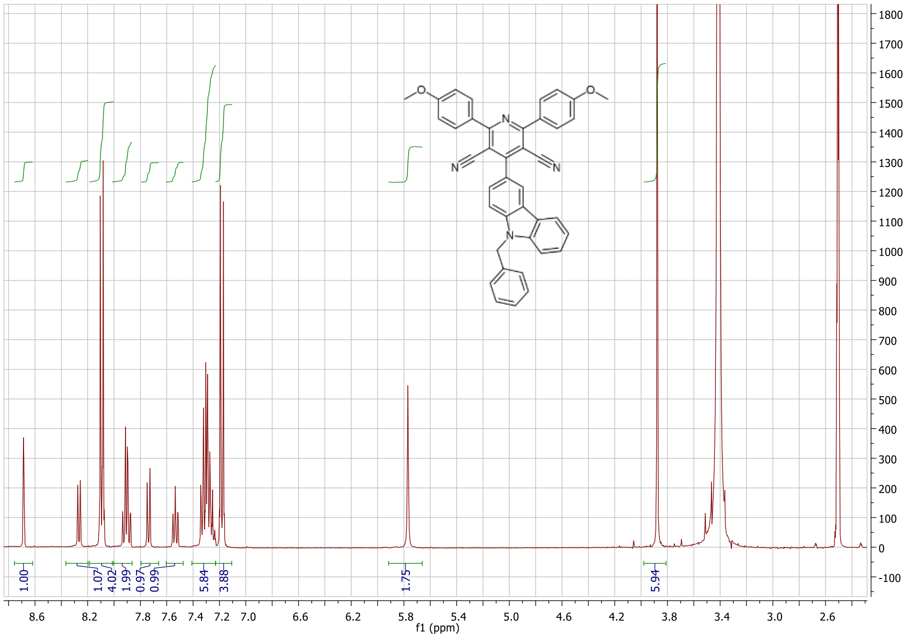

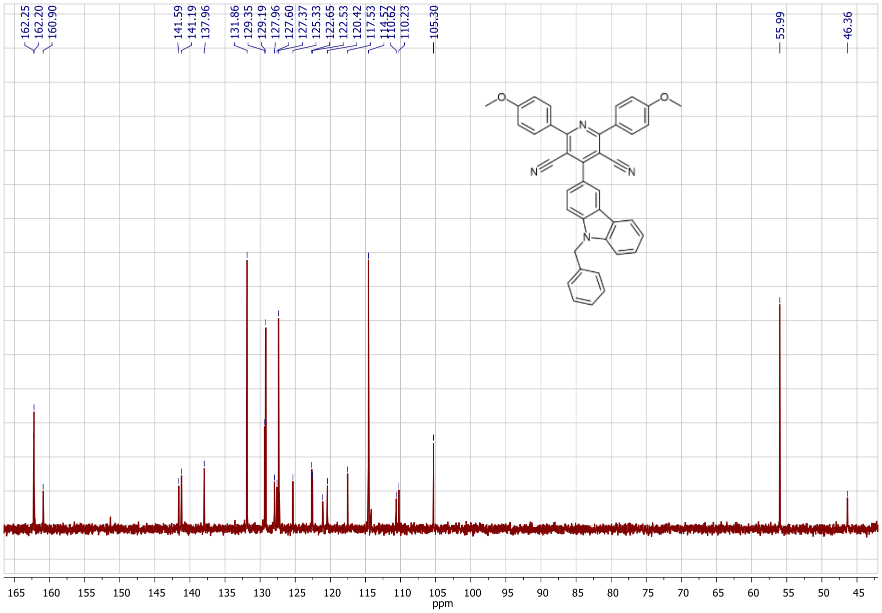

CNP-239

^{1}H NMR (400 MHz, DMSO) δ 8.68 – 8.62 (1H), 8.24 (1H), 8.20 – 8.04 (4H), 8.00 – 7.84 (2H), 7.73 (1H), 7.61 – 7.48 (1H), 7.30 (1H), 7.26 – 7.14 (4H), 4.56 (2H), 3.88 (6H), 1.41 (3H). ^{13}C NMR (101 MHz, DMSO) δ 162.21, 161.06, 140.98, 140.57, 131.86, 129.37, 127.40, 127.06, 124.99, 122.56, 122.47, 121.07, 120.07, 117.54, 114.58, 110.16, 109.85, 105.34, 56.00, 37.74, 14.34. Chemical Formula: $C_{35}H_{26}N_4O_2$, Elemental Analysis: calcd., C,78.63; H, 4.90; N, 10.48; found, C, 78.59; H, 4.88; N, 10.61. HRMS (m/z): $[M+H]^+$ calcd., 535.2129; found, 535.2125.

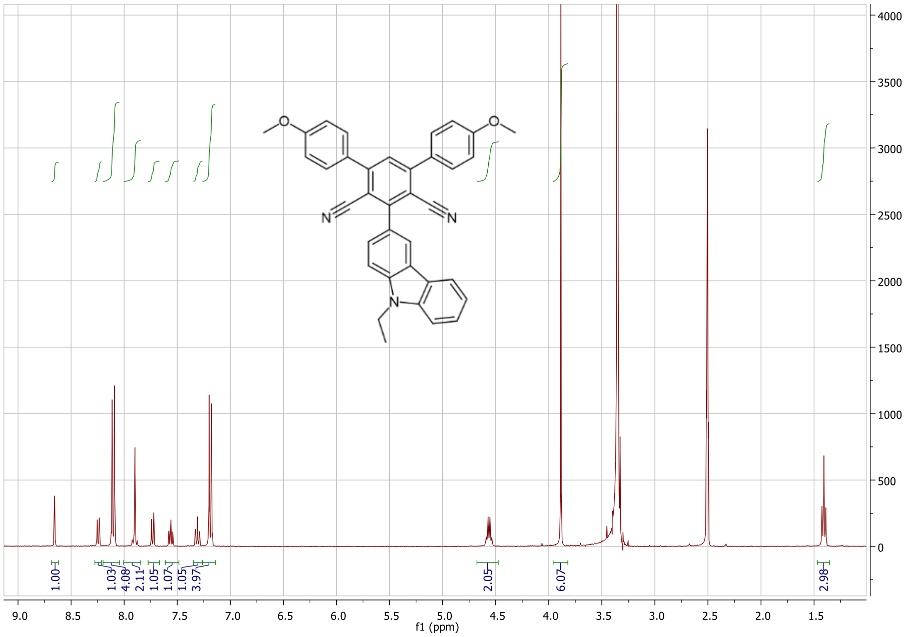

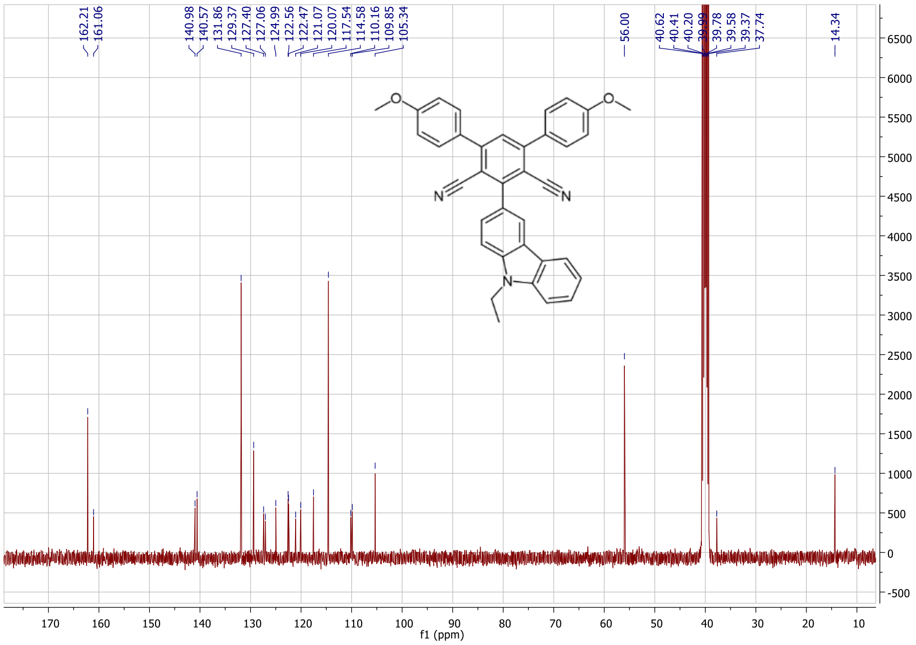

CNP-240

^{1}H NMR (400 MHz, DMSO) δ 8.65 (1H), 8.24 (1H), 8.15 – 8.03 (4H), 7.90 (2H), 7.71 (1H), 7.57 (1H), 7.31 (1H), 7.18 (4H), 4.00 (3H), 3.88 (6H). ^{13}C NMR (101 MHz, DMSO) δ 162.21, 162.16, 161.11, 142.01, 141.68, 131.85, 129.36, 127.38, 127.04, 125.02, 122.41, 122.29, 120.93, 120.08, 117.51, 114.58, 110.20, 109.99, 105.36, 56.00, 29.73. Chemical Formula: $C_{34}H_{24}N_4O_2$, Elemental Analysis: calcd., C, 78.44; H, 4.65; N, 10.76; found, C, 77.71; H, 4.56; N, 10.62. HRMS (m/z): $[M+H]^+$ calcd., 521.1972; found, 521.1973.

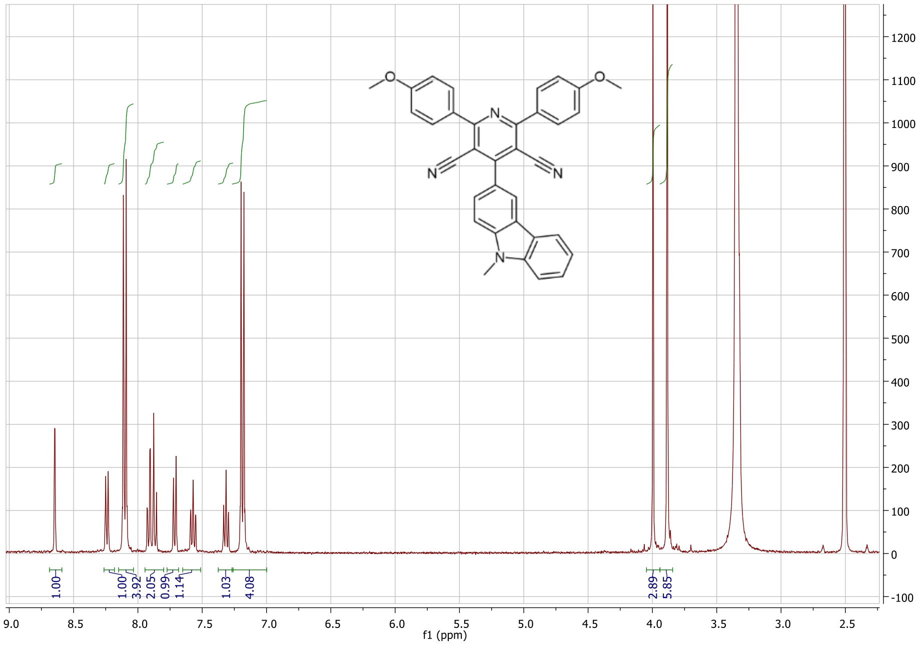

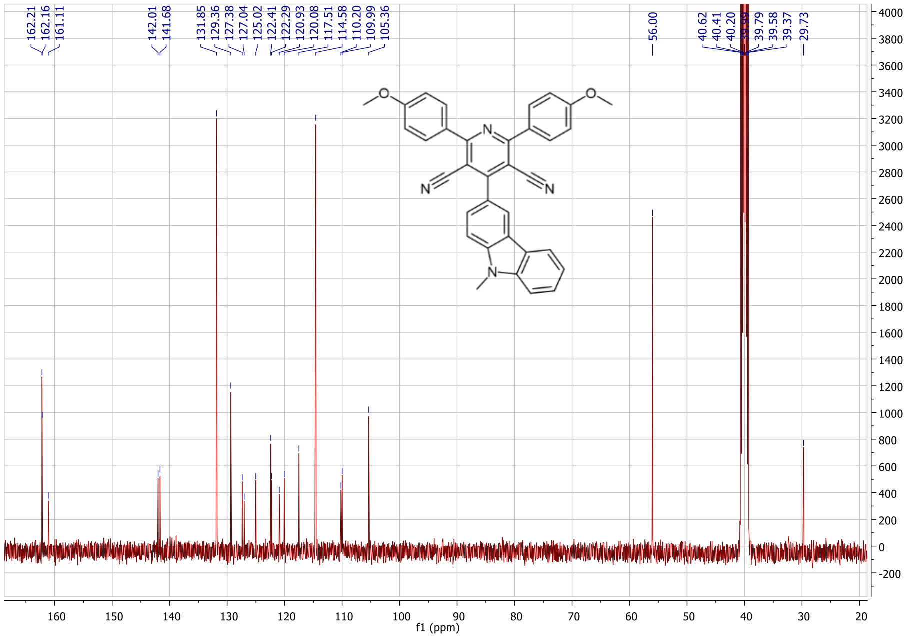

CNP-243

^{1}H NMR (400 MHz, DMSO) δ 8.30 (2H), 8.18 – 8.04 (6H), 8.01 – 7.93 (2H), 7.59 – 7.43 (4H), 7.40 – 7.28 (2H), 7.24 – 7.15 (4H), 3.90 (6H). ^{13}C NMR (101 MHz, DMSO) δ 162.31, 162.15, 159.37, 140.23, 139.34, 133.52, 131.86, 131.80, 129.23, 127.13, 126.98, 123.54, 121.18, 121.00, 117.09, 114.68, 110.10, 105.08, 56.02. Chemical Formula: $C_{39}H_{26}N_4O_2$, Elemental Analysis: calcd., C, 80.39; H, 4.50; N, 9.62; found, C, 79.27; H, 4.41; N, 9.45. HRMS (m/z): $[M+H]^+$ calcd., 583.2129; found, 583.2128.

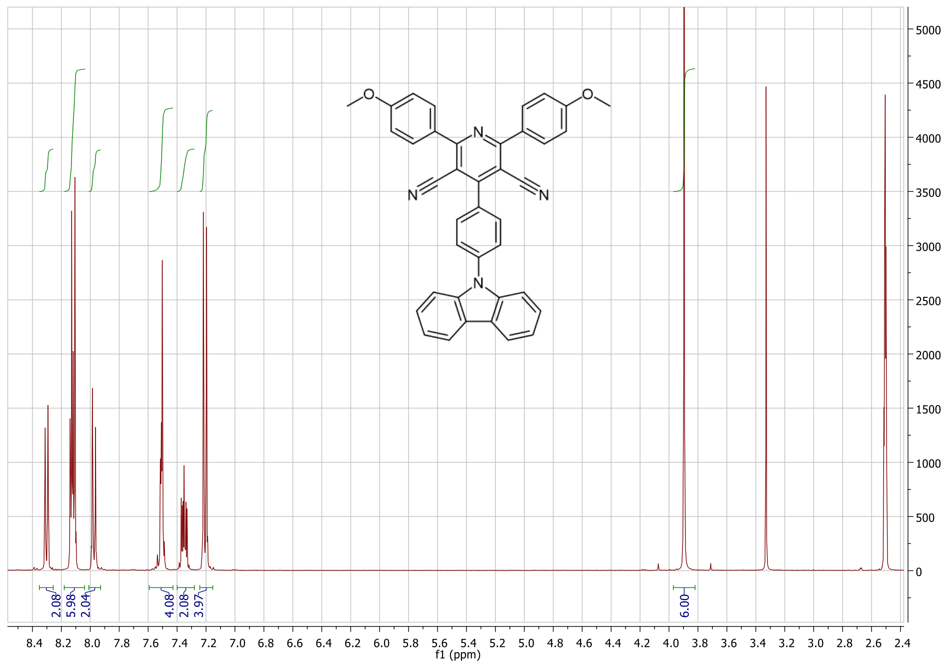

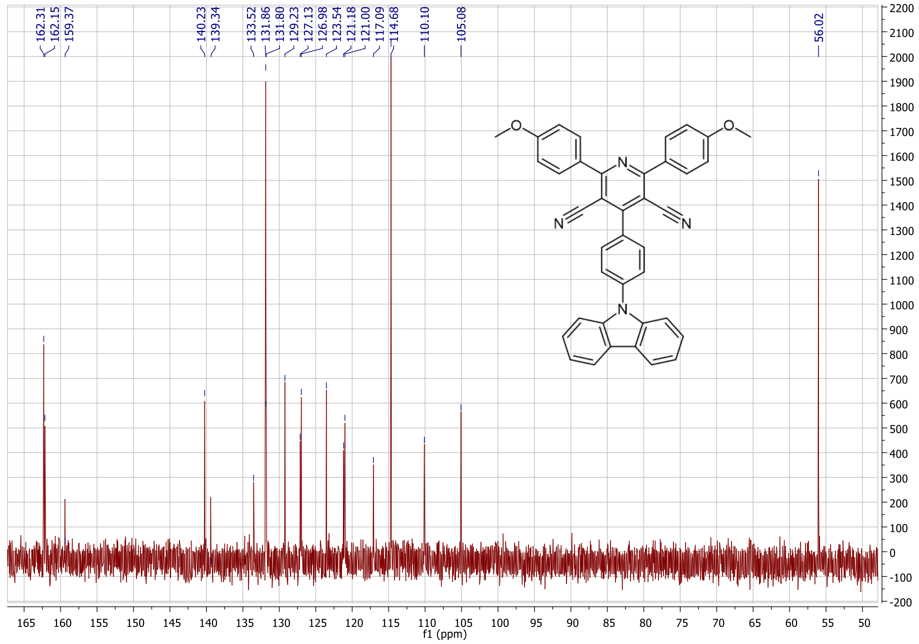

CNP-244

^{1}H NMR (400 MHz, DMSO) δ 8.55 (1H), 8.45 (2H), 8.42 – 8.29 (4H), 8.19 (1H), 8.17 – 8.10 (4H), 8.06 (1H), 7.29 – 7.12 (m, 4H), 3.88 (s, 6H). ^{13}C NMR (101 MHz, DMSO) δ 162.29, 161.99, 132.60, 131.86, 131.07, 130.60, 129.66, 129.19, 128.61, 127.65, 126.85, 126.61, 125.34, 124.29, 124.14, 116.79, 114.68, 106.37, 56.01, 40.60. Chemical Formula: $C_{37}H_{23}N_3O_2$, Elemental Analysis: calcd., C, 82.05; H, 4.28; N, 7.76; found, C, 81.66; H, 4.25; N, 7.66. HRMS (m/z): $[M+H]^+$ calcd., 542.1863; found, 542.1864.

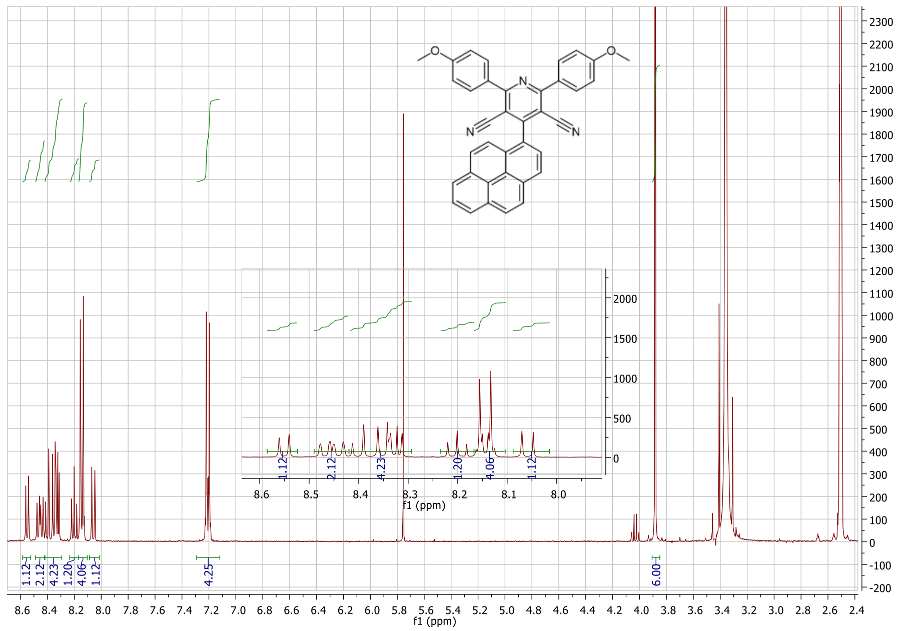

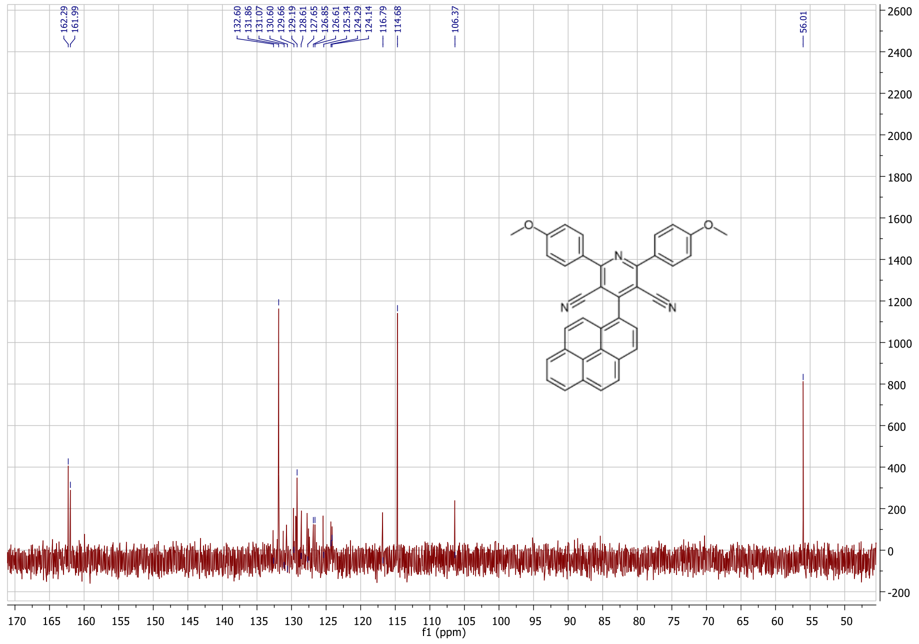

CNP-263

^{1}H NMR (400 MHz, DMSO) δ 8.33 – 8.25 (4H), 8.10 (12H), 8.03 – 7.90 (4H), 7.73 – 7.65 (2H), 7.57 – 7.50 (3H). ^{13}C NMR (101 MHz, DMSO) δ 162.19, 159.21, 143.88, 141.35, 136.91, 134.46, 133.46, 132.51, 132.20, 132.10, 132.03, 130.83, 130.28, 129.73, 129.35, 128.48, 128.40, 127.92, 125.34, 122.32, 119.24, 116.51, 111.29, 106.76, 91.97, 88.94. Chemical Formula: $C_{47}H_{25}N_5$, Elemental Analysis: calcd., C, 85.57; H, 3.82; N, 10.62; found, C, 83.23; H, 3.75; N, 10.02. HRMS (m/z): $[M+H]^+$ calcd., 660.2183; found, 660.2174.

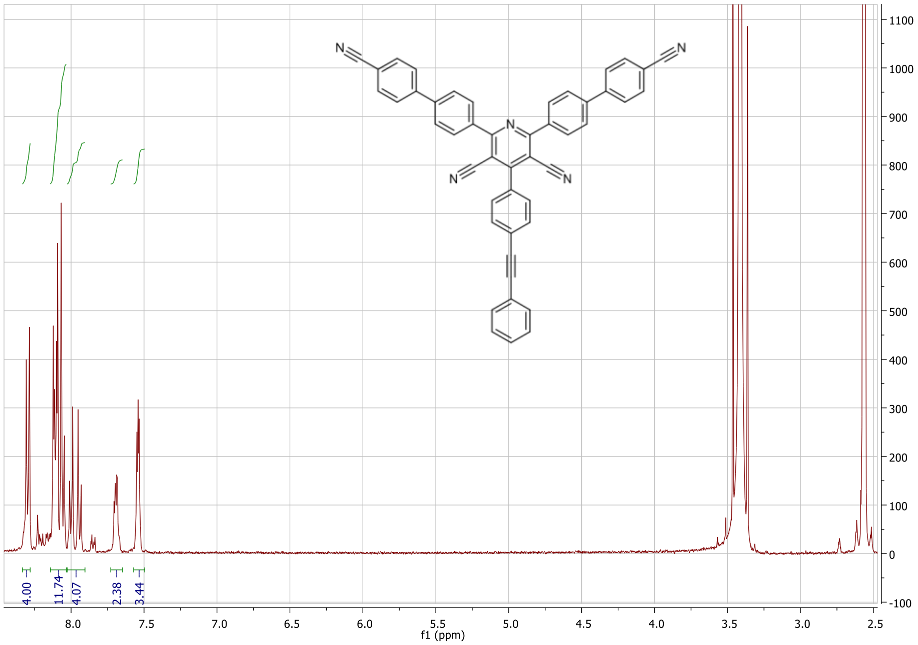

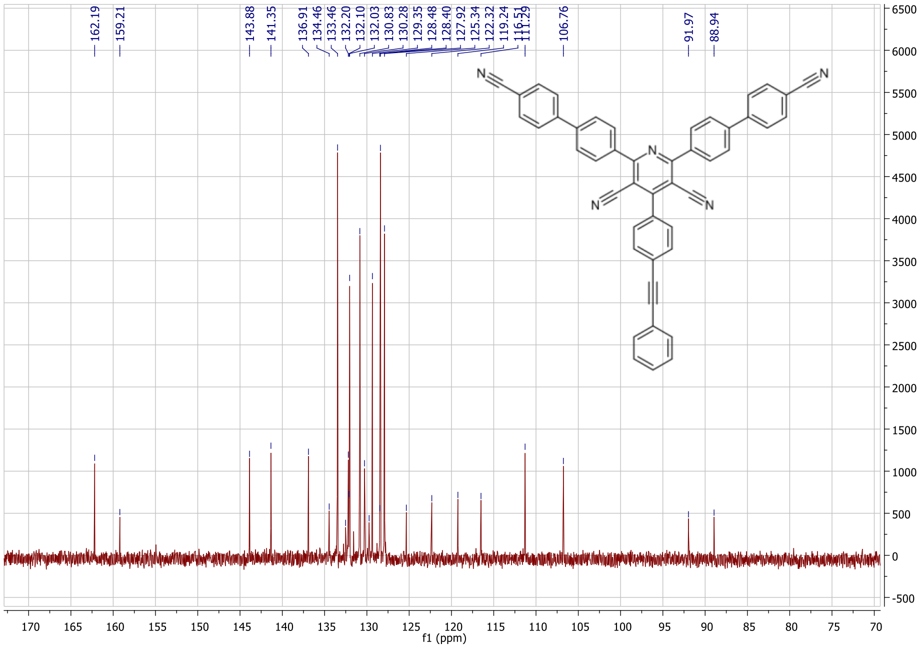

CNP-269

^{1}H NMR (400 MHz, DMSO) δ 8.83 (1H), 8.36 (1H), 8.25 (4H), 8.11 – 7.97 (11H), 7.97 – 7.86 (2H), 7.79 – 7.69 (4H), 7.62 (2H), 7.55 (1H), 7.43 (2H). ^{13}C NMR (101 MHz, DMSO) δ 167.74, 162.38, 143.93, 141.70, 141.38, 141.20, 137.12, 136.72, 133.46, 130.89, 130.84, 128.74, 128.39, 127.87, 127.59, 127.49, 127.27, 123.22, 122.66, 121.43, 119.07, 117.11, 111.26, 110.45, 107.05. Chemical Formula: $C_{51}H_{28}N_6$, Elemental Analysis: calcd., C, 84.51; H, 3.89; N, 11.59; found, C, 80.65; H, 3.84; N, 11.13. HRMS (m/z): $[M+H]^+$ calcd., 725.2448; found, 725.2449.

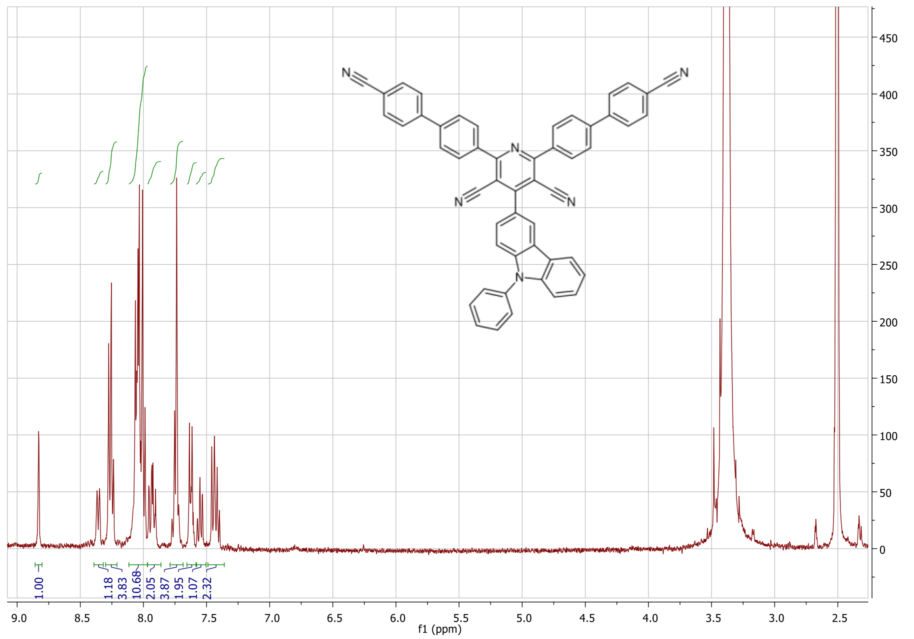

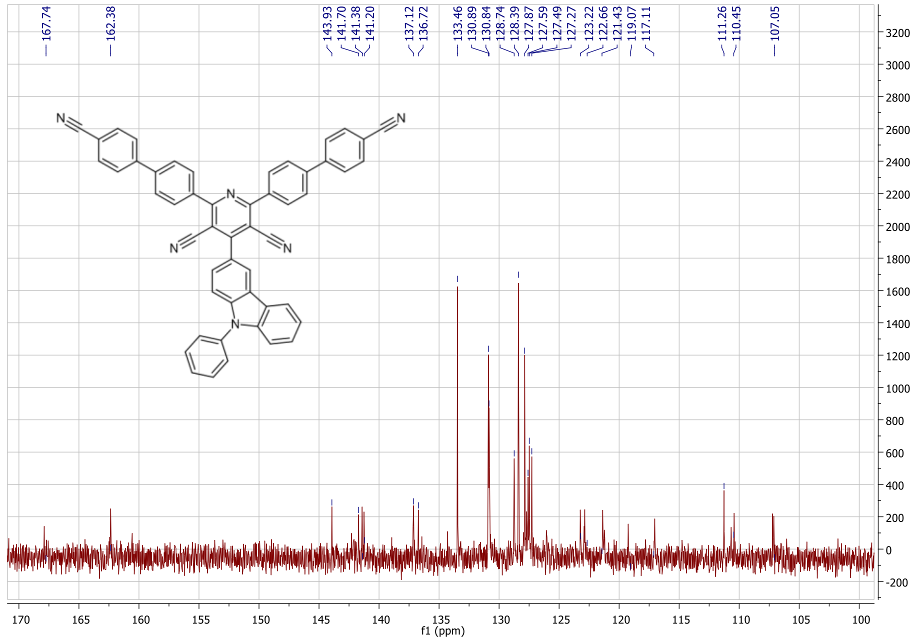

CNP-295

^{1}H NMR (400 MHz, DMSO) δ 8.68 (1H), 8.23 (1H), 8.18 – 8.08 (4H), 7.99 – 7.83 (2H), 7.79 – 7.67 (5H), 7.61 – 7.50 (1H), 7.39 – 7.24 (1H), 4.57 (2H), 1.41 (3H). ^{13}C NMR (101 MHz, DMSO) δ 161.88, 160.68, 141.11, 140.60, 136.73, 135.78, 131.90, 129.28, 127.38, 127.15, 124.46, 122.68, 122.60, 122.43, 121.01, 120.18, 116.94, 110.23, 109.97, 107.40, 37.77, 14.33. Chemical Formula: $C_{33}H_{20}Cl_2N_4$, Elemental Analysis: calcd., C, 72.93; H, 3.71; N, 10.31; found, C, 72.64; H, 3.36; N, 10.29. HRMS (m/z): $[M+H]^+$ calcd., 543.1138; found, 543.1135.

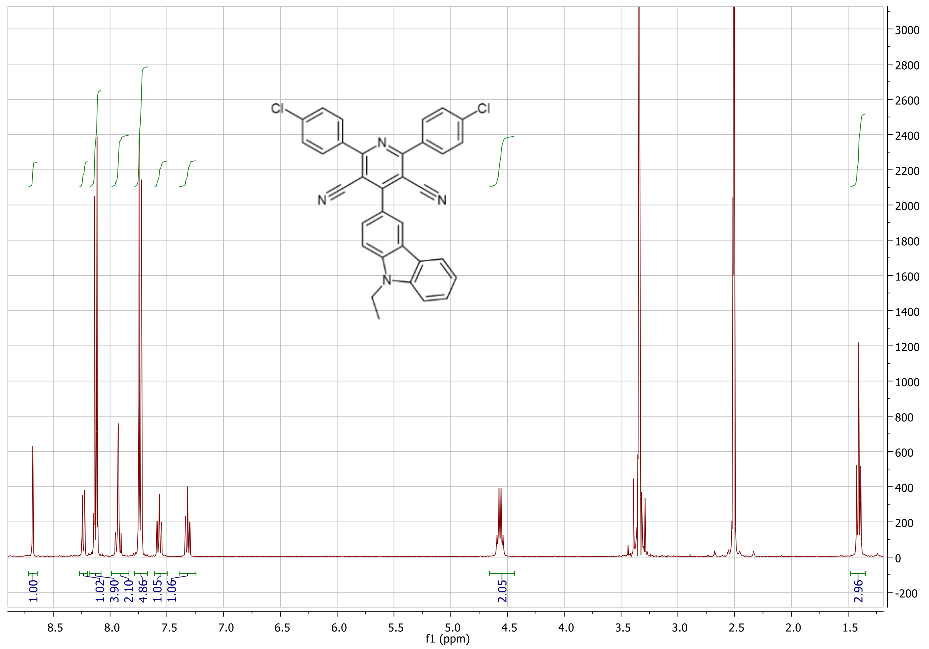

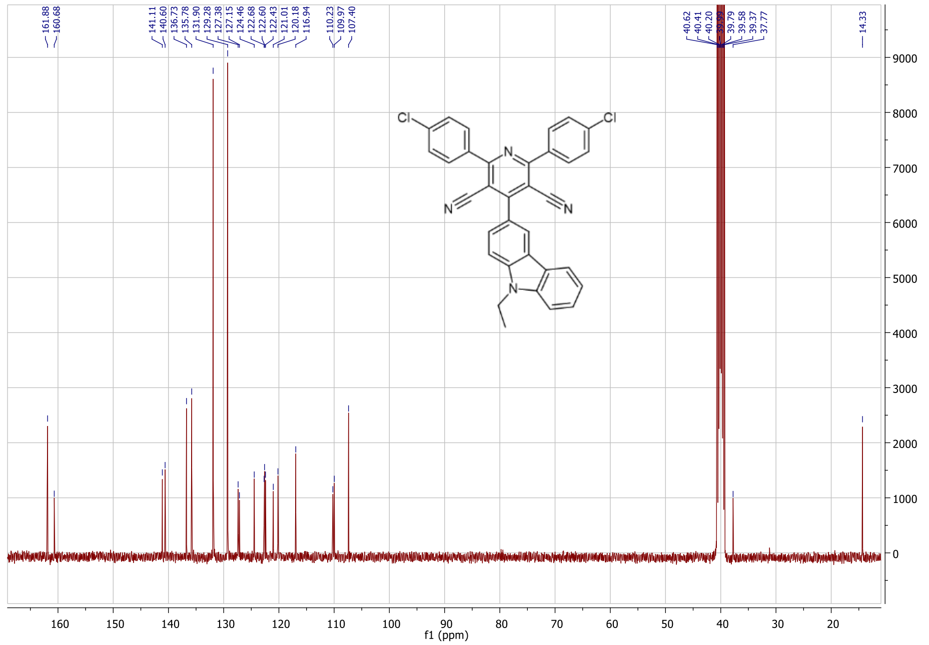

CNP-302

^{1}H NMR (400 MHz, $CDCl_3$) δ 8.03 – 7.94 (4H), 7.58 – 7.49 (6H), 7.46 – 7.39 (2H), 7.33 – 7.19 (m, 3H), 6.94 (2H), 3.42 (3H). Chemical Formula: $C_{32}H_{20}Cl_2N_4$. HRMS (m/z): $[M+H]^+$ calcd., 531.1138; found, 531.1126.

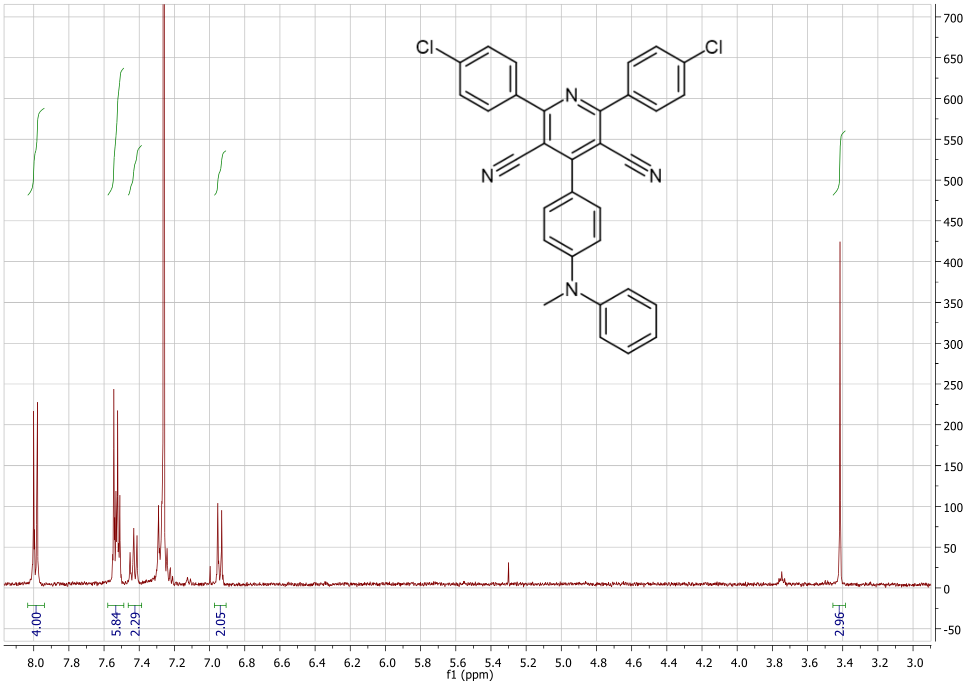

CNP-303

^{1}H NMR (400 MHz, DMSO) δ 8.19 – 8.06 (4H), 8.04 – 7.87 (4H), 7.73 (6H), 7.35 (2H), 2.38 (3H). ^{13}C NMR (101 MHz, DMSO) δ 161.84, 159.47, 142.81, 138.26, 136.81, 136.35, 135.63, 132.86, 131.86, 130.45, 130.21, 129.32, 127.26, 127.11, 116.50, 107.06, 21.21. Chemical Formula: $C_{32}H_{19}Cl_2N_3$, Elemental Analysis: calcd., C, 74.43; H, 3.71; N, 8.14; found, C, 74.31; H, 3.66; N, 8.16. HRMS (m/z): $[M+H]^+$ calcd., 516.1029; found, 516.1026.

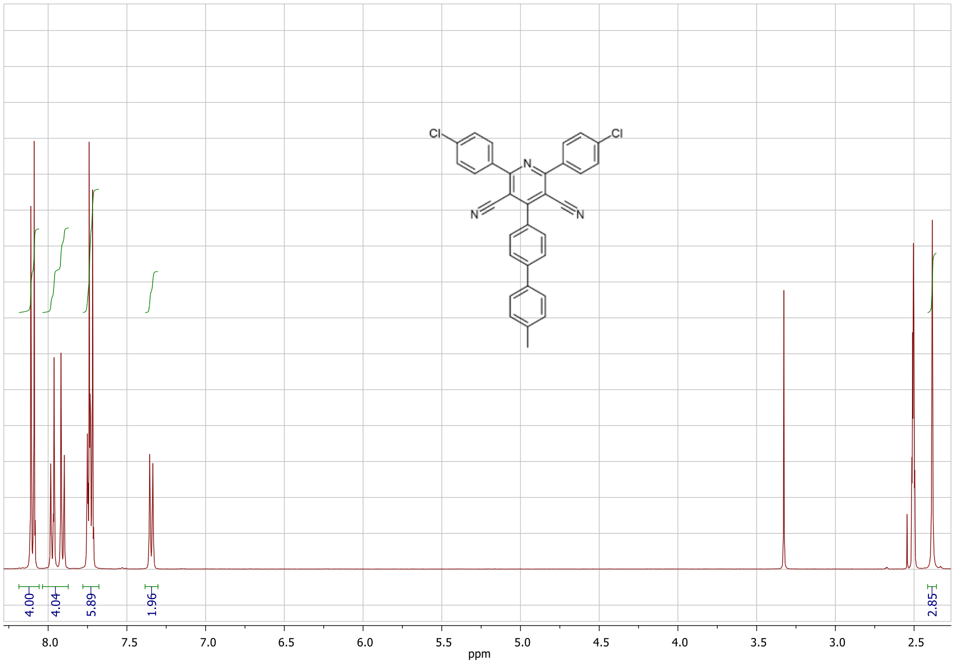

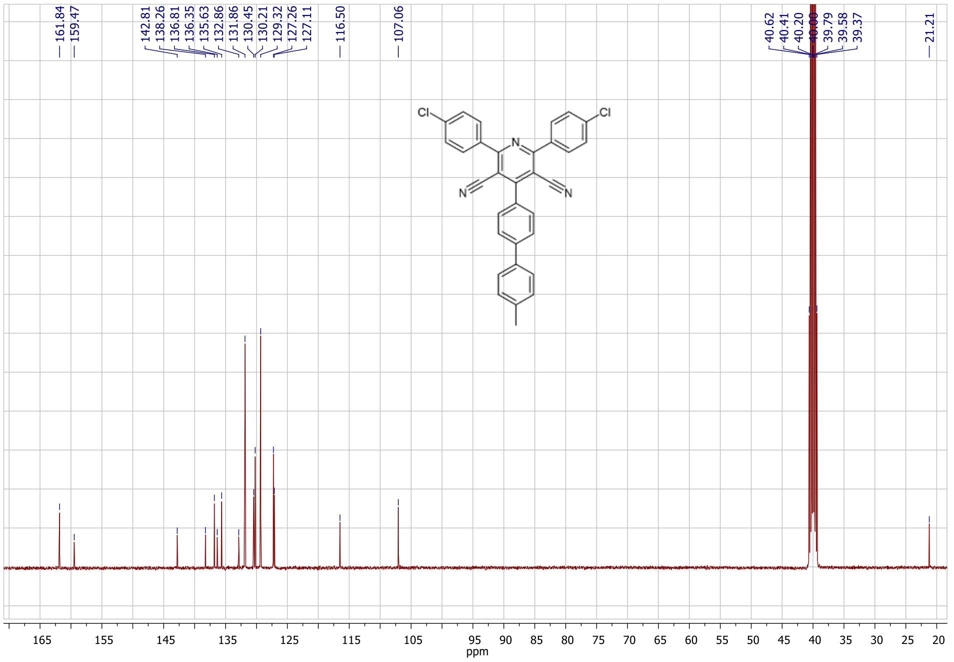

CNP-315

^{1}H NMR (400 MHz, DMSO) δ 8.34 (1H), 8.08 (1H), 8.01 (1H), 7.90 – 7.79 (5H), 7.56 – 7.40 (5H), 7.32 (1H), 3.95 (3H), 2.44 (6H). ^{13}C NMR (101 MHz, DMSO) δ 163.22, 159.90, 159.28, 138.54, 137.04, 135.66, 132.22, 130.65, 130.36, 129.76, 129.49, 129.03, 128.13, 127.72, 127.17, 127.00, 120.31, 116.77, 106.90, 106.59, 55.94, 21.45. Chemical Formula: $C_{32}H_{23}N_3O$, Elemental Analysis: calcd., C, 82.56; H, 4.98; N, 9.03; found, C, 82.34; H, 4.91; N, 9.06. HRMS (m/z): $[M+H]^+$ calcd., 466.1914; found, 466.1911.

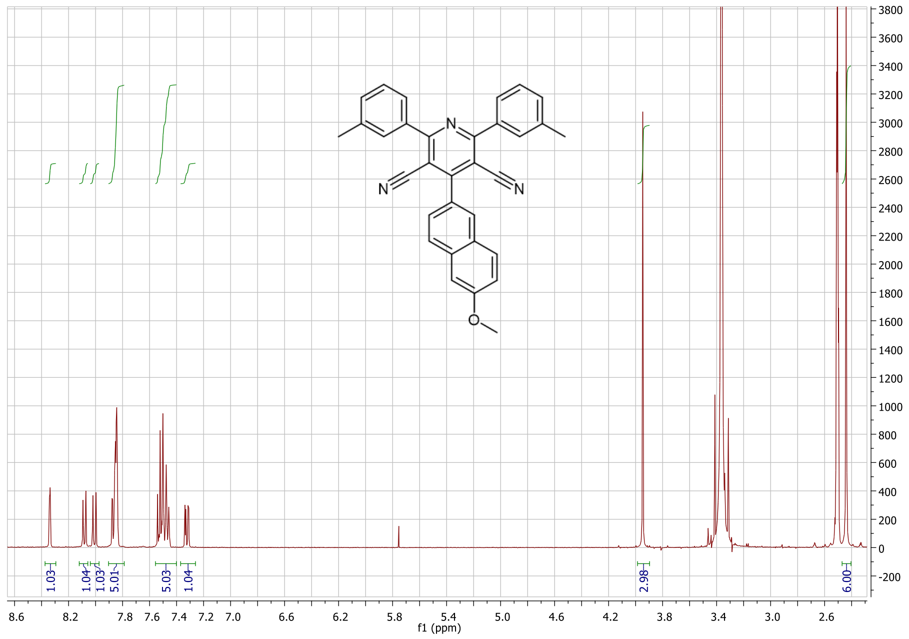

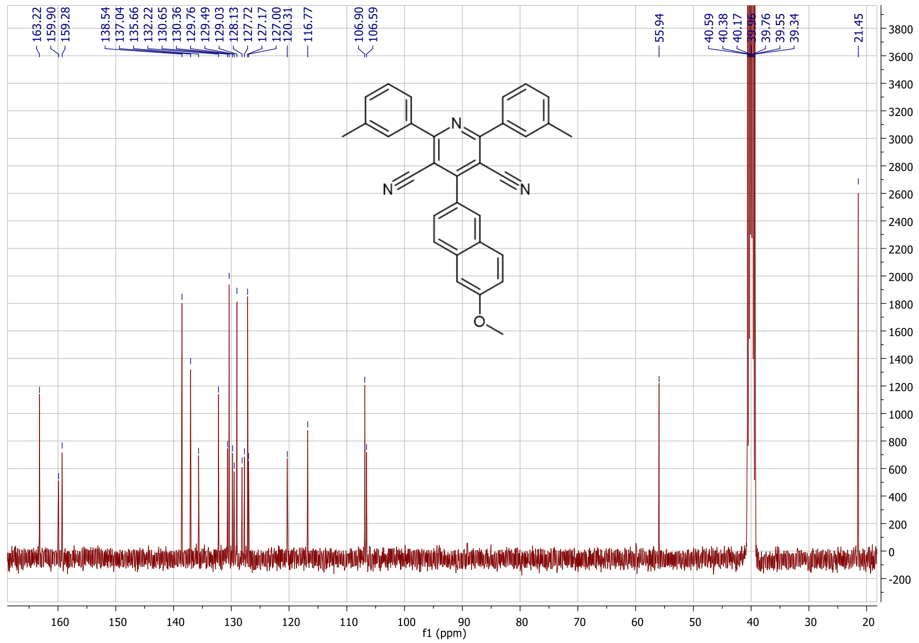

CNP-323

^{1}H NMR (400 MHz, DMSO) δ 8.68 (1H), 8.25 (1H), 7.93 (1H), 7.91 – 7.82 (5H), 7.72 (1H), 7.54 (3H), 7.49 – 7.43 (2H), 4.55 (2H), 2.44 (6H), 1.40 (3H). ^{13}C NMR (101 MHz, DMSO) δ 163.29, 160.68, 141.02, 140.57, 138.51, 137.16, 132.15, 130.38, 128.98, 127.43, 127.21, 127.11, 124.71, 122.62, 122.58, 122.44, 121.09, 120.11, 117.11, 110.16, 109.87, 107.06, 37.75, 21.45, 14.32. Chemical Formula: $C_{35}H_{26}N_4$, Elemental Analysis: calcd., C, 83.64; H, 5.21; N, 11.15; found, C, 83.71; H, 5.17; N, 11.25. HRMS (m/z): $[M+H]^+$ calcd., 503.2230; found, 503.2233.

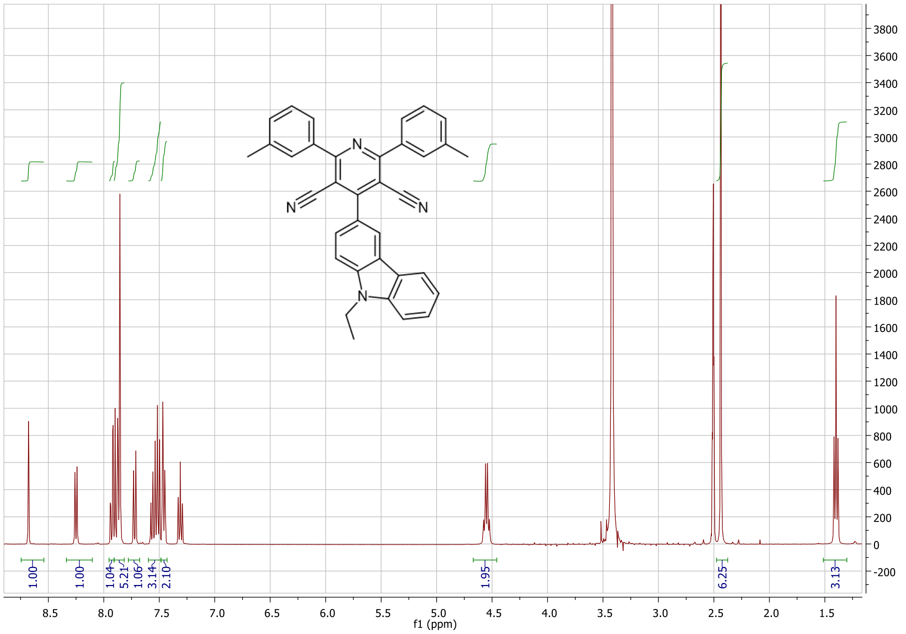

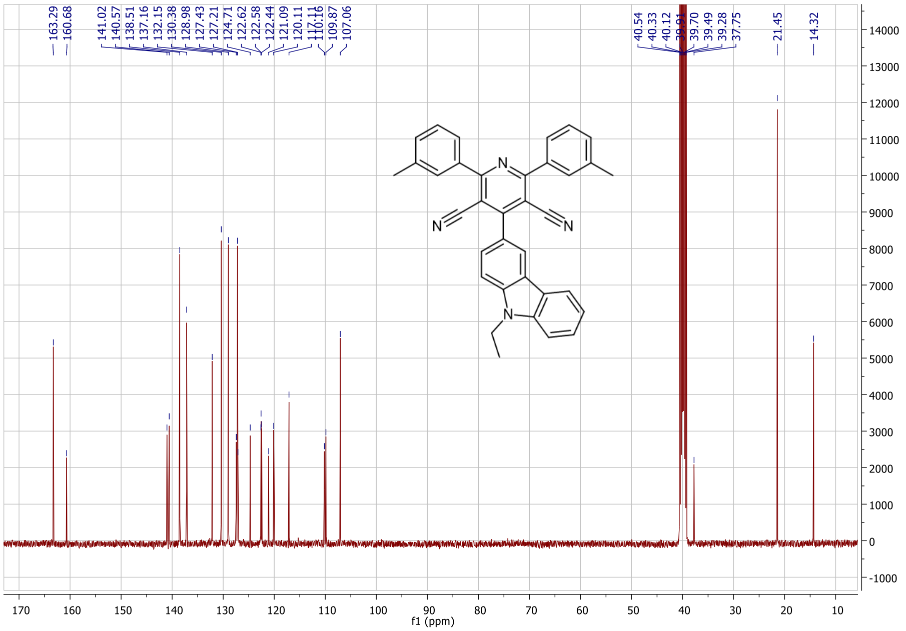

CNP-327

^{1}H NMR (400 MHz, DMSO) δ 8.33 – 8.26 (2H), 8.21 – 8.11 (2H), 8.04 – 7.94 (2H), 7.88 (4H), 7.59 – 7.45 (8H), 7.41 – 7.29 (2H), 2.46 (6H). ^{13}C NMR (101 MHz, DMSO) δ 163.21, 159.06, 140.21, 139.52, 138.59, 137.01, 133.24, 132.30, 131.84, 130.43, 129.07, 127.18, 127.13, 126.98, 123.56, 121.18, 121.02, 116.67, 110.11, 106.84, 21.46. Chemical Formula: $C_{39}H_{26}N_4$, Elemental Analysis: calcd., C, 85.07; H, 4.76; N, 10.17; found, C, 85.25; H, 4.69; N, 10.21. HRMS (m/z): $[M+H]^+$ calcd., 551.2230; found, 551.2229.

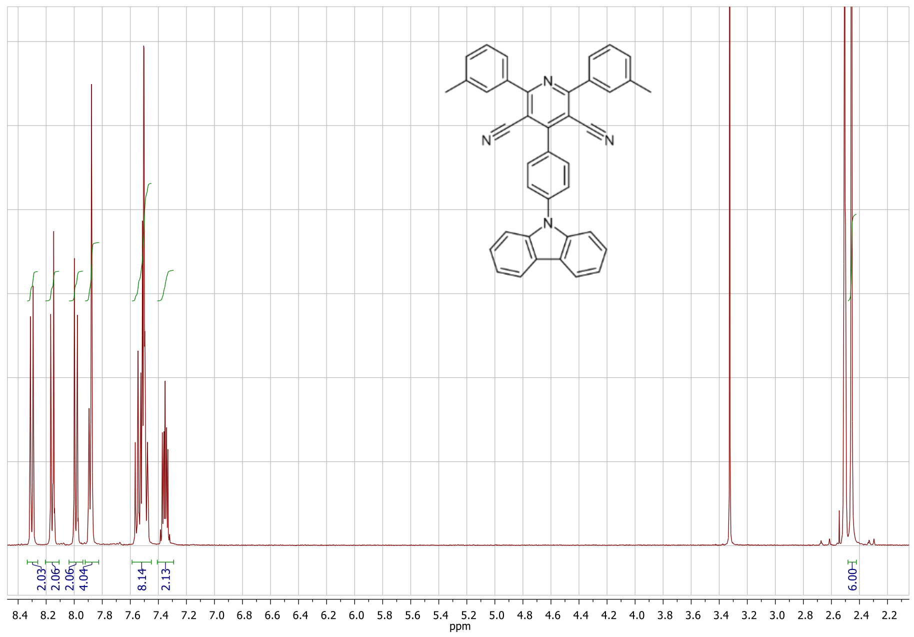

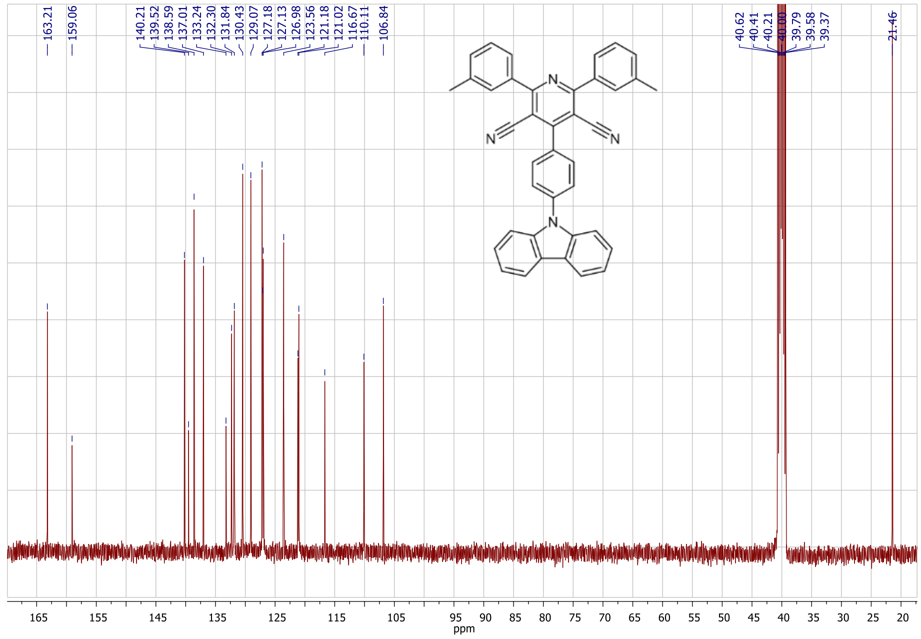

CNP-328

^{1}H NMR (400 MHz, DMSO) δ 8.56 (1H), 8.46 (2H), 8.40 (1H), 8.35 (3H), 8.20 (1H), 8.07 (1H), 7.96 – 7.86 (4H), 7.58 – 7.51 (2H), 7.48 (2H), 2.45 (6H). ^{13}C NMR (101 MHz, DMSO) δ 163.10, 159.60, 138.57, 136.99, 132.74, 132.28, 131.20, 130.73, 130.41, 129.80, 129.49, 129.07, 129.05, 128.59, 127.73, 127.57, 127.44, 127.21, 126.89, 126.65, 125.43, 124.31, 124.12, 124.00, 116.39, 108.23, 21.48. Chemical Formula: $C_{37}H_{23}N_3$, Elemental Analysis: calcd., C, 87.20; H, 4.55; N, 8.25; found, C, 87.2; H, 4.5; N, 8.38. HRMS (m/z): $[M+H]^+$ calcd., 510.1965; found, 510.1966.

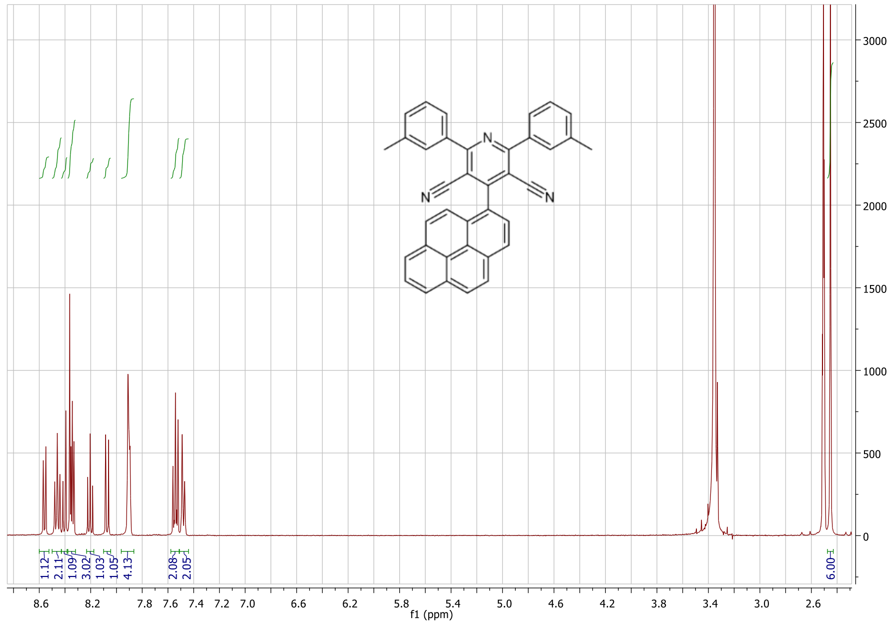

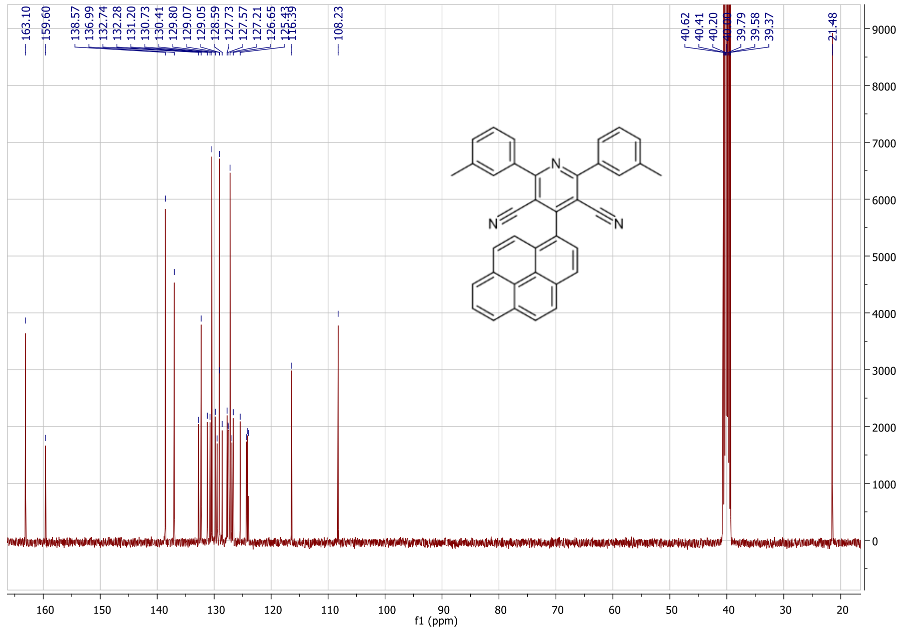

CNP-336

^{1}H NMR (400 MHz, DMSO) δ 8.72 (2H), 8.25 (1H), 8.23 – 8.12 (7H), 8.10 – 8.04 (2H), 7.90 (1H), 7.89 – 7.84 (1H), 7.81 (1H), 7.74 – 7.62 (6H). ^{13}C NMR (101 MHz, DMSO) δ 162.95, 159.44, 134.33, 134.28, 133.52, 132.67, 132.14, 131.31, 130.49, 130.42, 129.43, 129.15, 128.87, 128.66, 128.23, 127.60, 127.34, 126.63, 125.97, 125.27, 116.36, 108.20. Chemical Formula: $C_{37}H_{21}N_3$, Elemental Analysis: calcd., C, 87.55; H, 4.17; N, 8.28; found C, 87.33; H, 4.12; N, 8.23. HRMS (m/z): $[M+H]^+$ calcd., 508.1808; found, 508.1804.

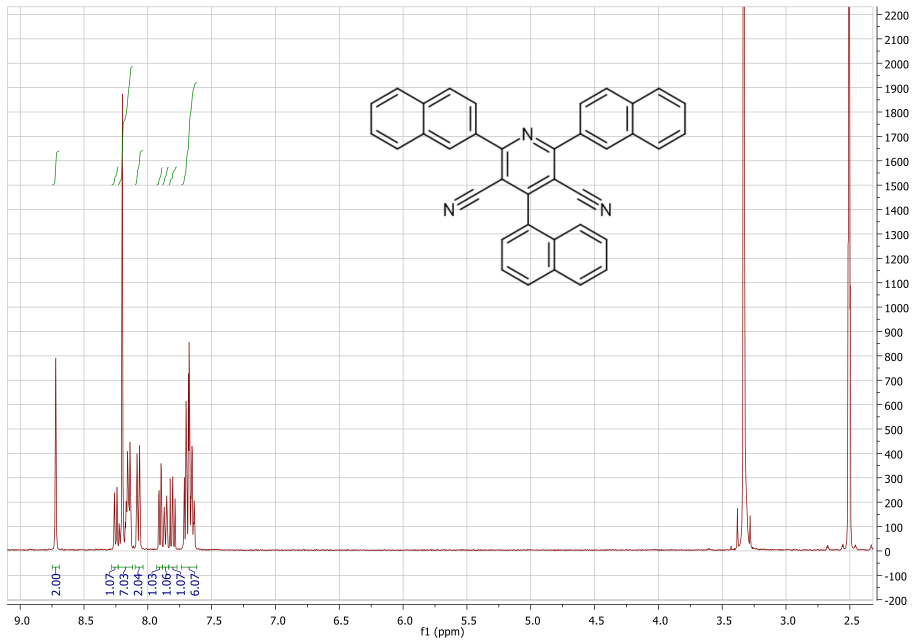

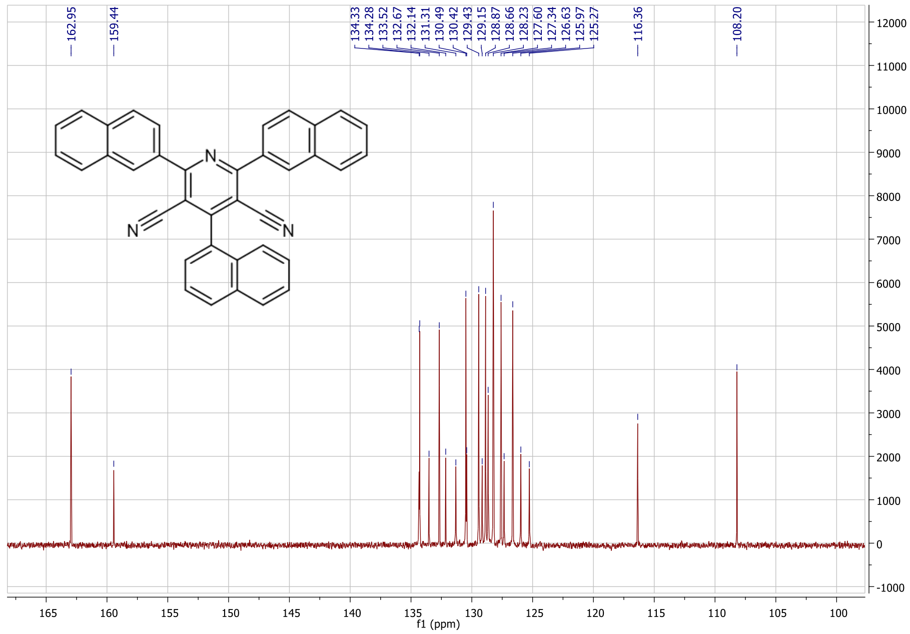

CNP-338

^{1}H NMR (400 MHz, DMSO) δ 8.71 (2H), 8.25 – 8.17 (4H), 8.17 – 8.12 (2H), 8.07 (2H), 7.87 (1H), 7.73 – 7.57 (7H), 7.54 – 7.45 (1H), 3.51 (4H). ^{13}C NMR (101 MHz, DMSO) δ 163.06, 159.19, 149.96, 147.06, 139.09, 134.41, 134.27, 132.69, 130.47, 130.39, 130.04, 129.44, 128.87, 128.82, 128.61, 128.22, 127.56, 127.36, 126.67, 120.94, 120.36, 119.59, 116.54, 108.21, 30.49. Chemical Formula: $C_{39}H_{23}N_3$, Elemental Analysis: calcd., C, 87.78; H, 4.34; N, 7.87; found, C, 86.76; H, 4.33; N, 7.79. HRMS (m/z): $[M+H]^+$ calcd., 534.1965; found, 534.1964.

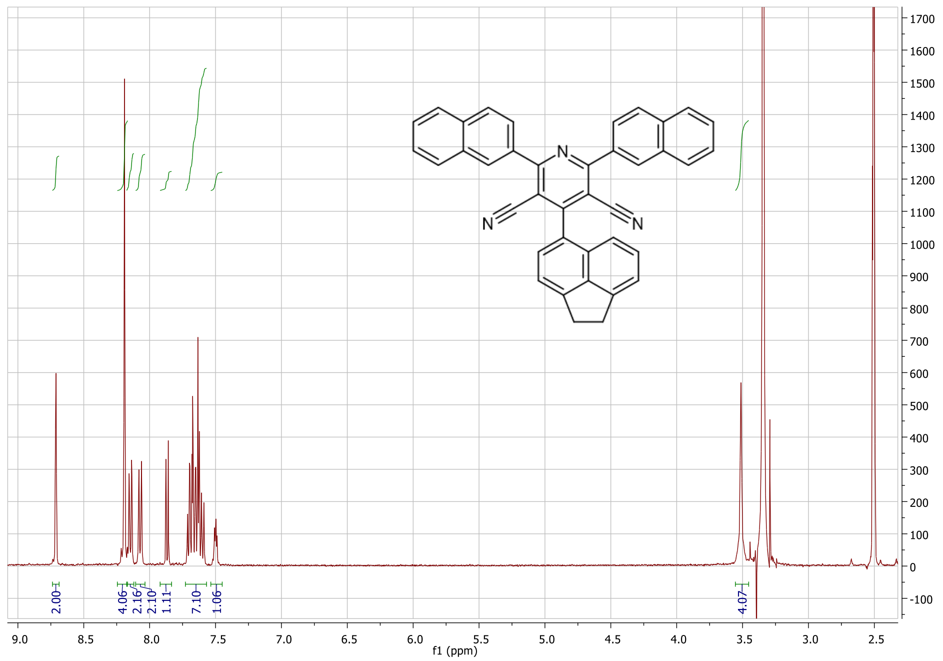

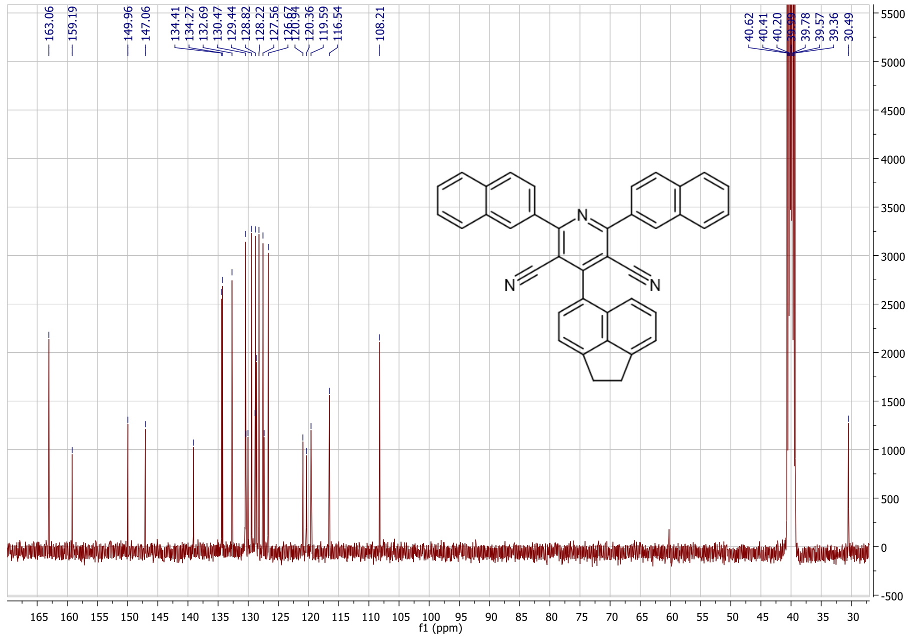

CNP-346

^{1}H NMR (400 MHz, DMSO) δ 8.79 (1H), 8.73 (2H), 8.30 (1H), 8.23 – 8.16 (4H), 8.11 (4H), 8.04 – 7.94 (2H), 7.76 (1H), 7.68 (4H), 7.55 (1H), 7.42 – 7.17 (6H), 5.80 (2H). ^{13}C NMR (101 MHz, DMSO) δ 163.13, 160.73, 141.74, 141.24, 137.98, 134.56, 134.29, 132.73, 130.51, 129.39, 129.20, 128.79, 128.59, 128.24, 127.95, 127.71, 127.56, 127.40, 127.28, 126.71, 125.11, 122.73, 122.70, 122.56, 121.11, 120.49, 117.25, 110.70, 110.38, 107.34, 46.40. Chemical Formula: $C_{46}H_{28}N_4$, Elemental Analysis: calcd., C, 86.77; H, 4.43; N, 8.80; found, C, 86.41; H, 4.38; N, 8.80. HRMS (m/z): $[M+H]^+$ calcd., 637.2387; found, 637.2385.

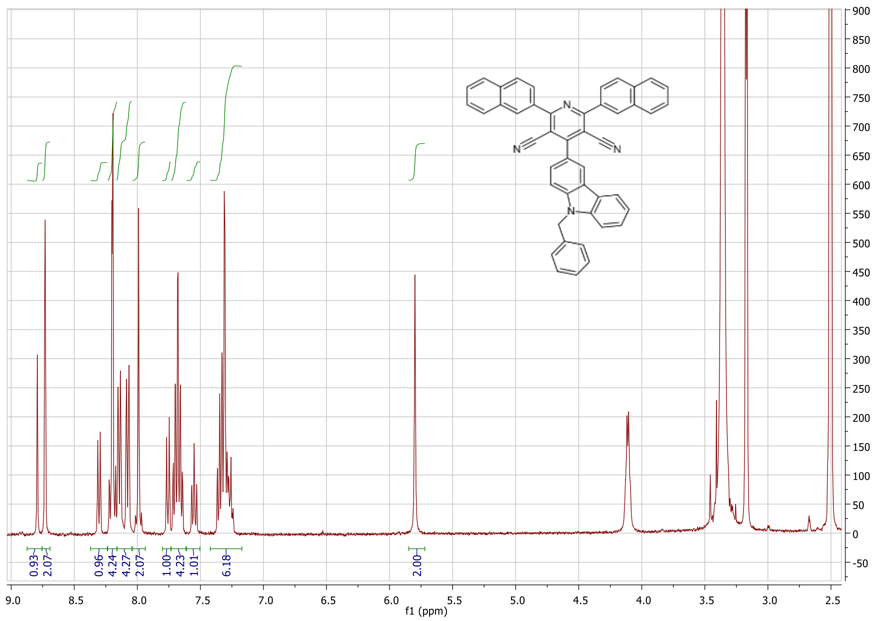

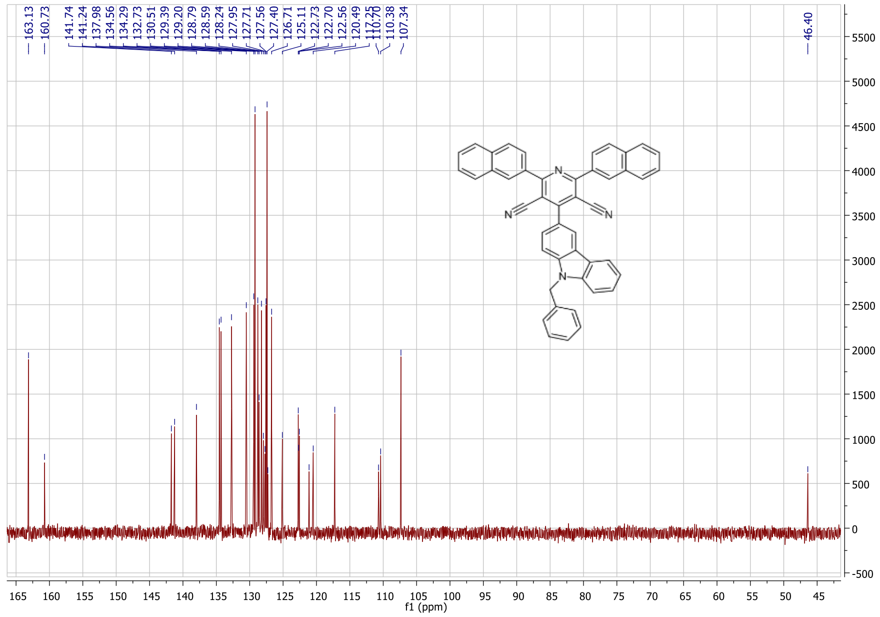

CNP-351

^{1}H NMR (400 MHz, DMSO) δ 8.77 – 8.75 (1H), 8.74 (2H), 8.28 (1H), 8.24 – 8.17 (4H), 8.15 (2H), 8.08 (2H), 8.01 (1H), 7.94 (1H), 7.75 (1H), 7.73 – 7.63 (4H), 7.61 – 7.50 (1H), 7.33 (1H), 4.58 (2H), 1.42 (3H). ^{13}C NMR (101 MHz, DMSO) δ 163.12, 160.87, 141.09, 140.61, 134.57, 134.29, 132.73, 130.50, 129.40, 128.78, 128.58, 128.24, 127.55, 127.50, 127.13, 126.72, 124.76, 122.66, 122.63, 122.48, 121.10, 120.15, 117.27, 110.22, 109.96, 107.37, 37.78, 14.35. Chemical Formula: $C_{41}H_{26}N_4$, Elemental Analysis: calcd., C, 85.69; H, 4.56; N, 9.75; found, C, 85.34; H, 4.48; N, 9.78. HRMS (m/z): $[M+H]^+$ calcd., 575.2230; found, 575.2231.

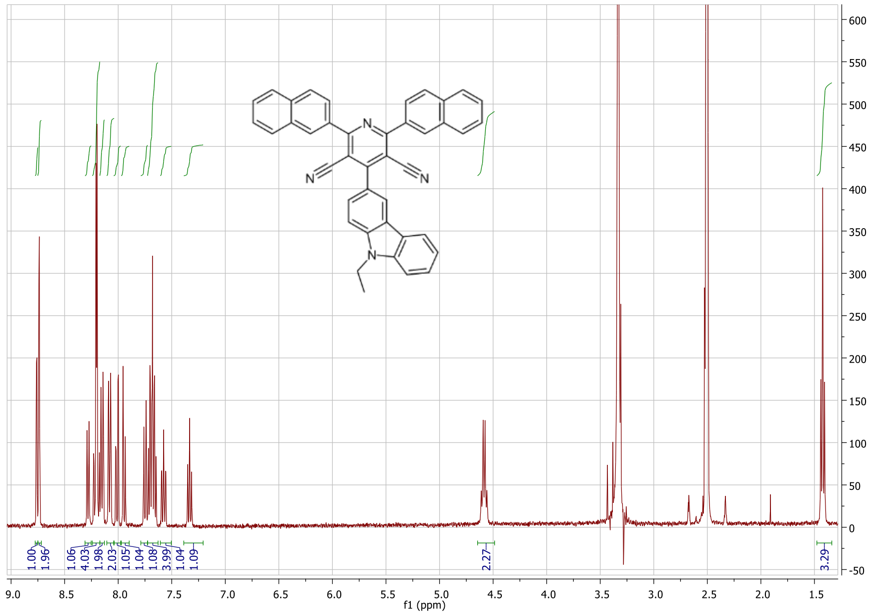

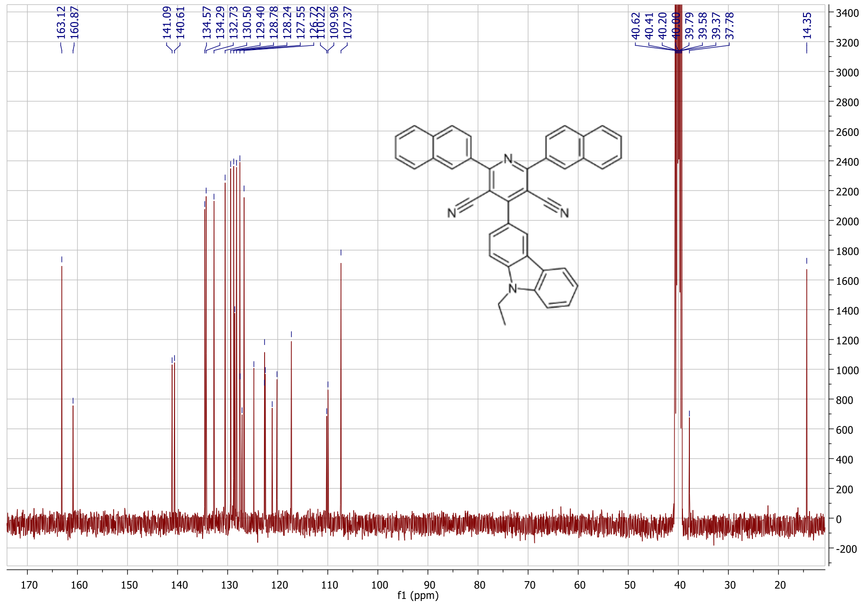

CNP-372

^{1}H NMR (400 MHz, DMSO) δ 8.37 – 8.31 (1H), 8.24 (2H), 8.06 (2H), 7.87 (2H), 7.78 (1H), 7.76 – 7.70 (1H), 7.69 – 7.57 (4H), 7.25 (1H), 4.11 (3H). ^{13}C NMR (101 MHz, DMSO) δ 161.42, 159.26, 157.29, 138.97, 134.31, 132.43, 131.36, 129.38, 129.05, 128.56, 126.72, 125.25, 125.13, 123.83, 122.60, 122.20, 116.04, 109.29, 104.56, 56.48. Chemical Formula: $C_{30}H_{17}Br_2N_3O$, Elemental Analysis: calcd., C, 60.53; H, 2.88; N, 7.06; found C, 60.53; H, 2.82; N, 6.95. HRMS (m/z): $[M+H]^+$ calcd., 593.9811; found, 593.9804.

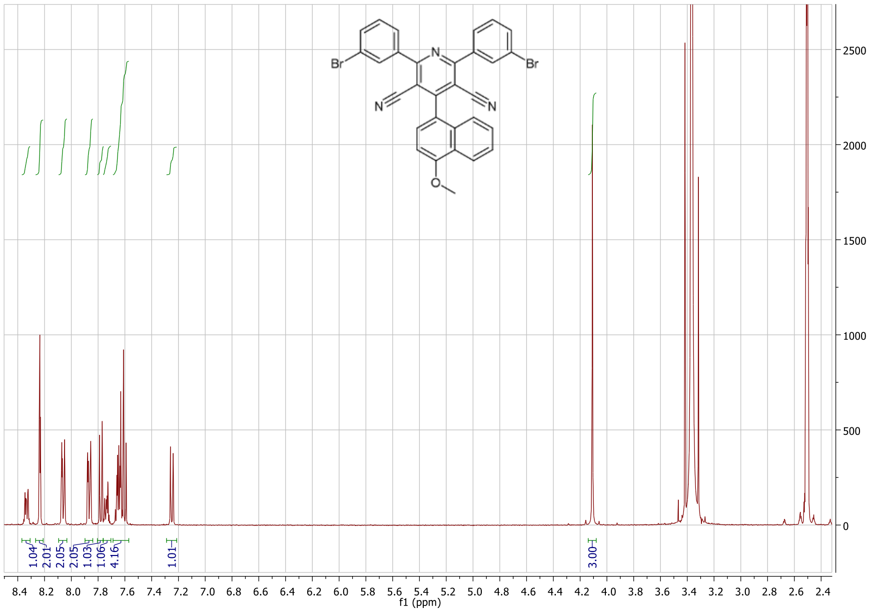

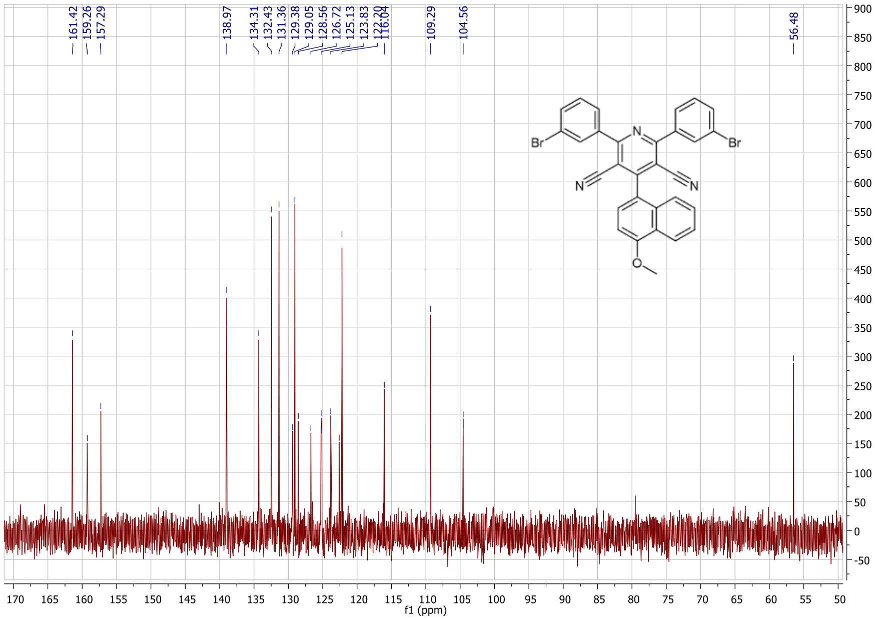

CNP-379

^{1}H NMR (400 MHz, DMSO) δ 8.74 – 8.67 (1H), 8.33 – 8.21 (3H), 8.09 (2H), 7.95 (2H), 7.87 (2H), 7.74 (1H), 7.62 (2H), 7.59 – 7.54 (1H), 7.32 (1H), 4.57 (2H), 1.48 – 1.32 (3H). ^{13}C NMR (101 MHz, DMSO) δ 161.55, 160.45, 141.15, 140.61, 139.14, 134.32, 132.48, 131.34, 129.17, 127.47, 127.17, 124.36, 122.73, 122.62, 122.44, 122.23, 121.06, 120.19, 116.79, 110.25, 109.99, 107.96, 37.78, 14.34. Chemical Formula: $C_{33}H_{20}Br_2N_4$, Elemental Analysis: calcd., C, 62.68; H, 3.19; N, 8.86; found C, 62.61; H, 3.12; N, 8.75. HRMS (m/z): $[M+H]^+$ calcd., 631.0127; found, 631.0120.

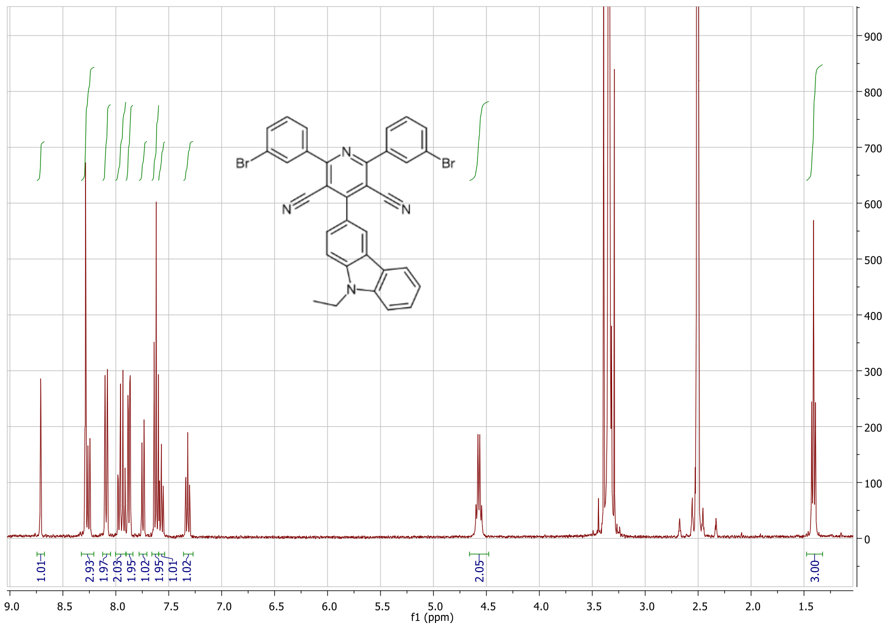

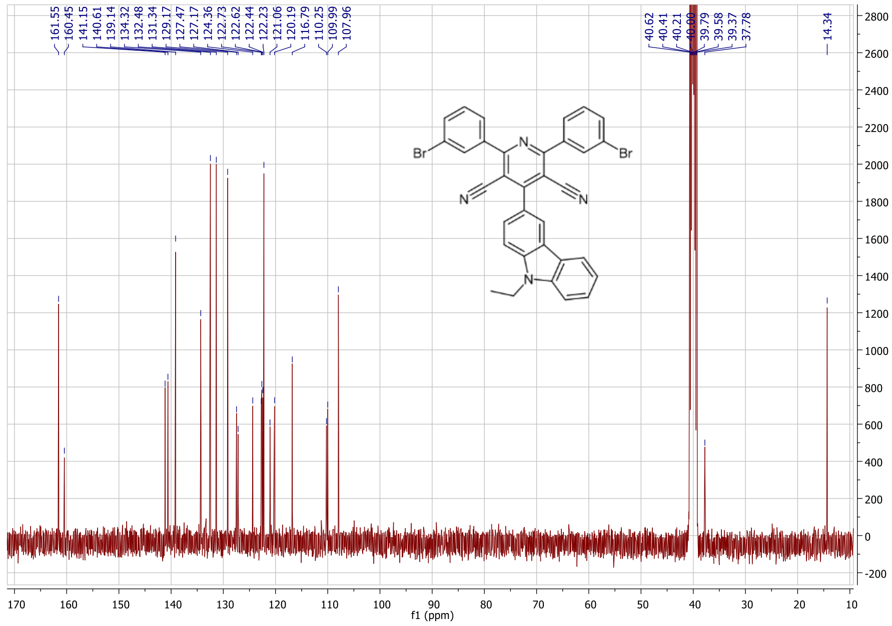

CNP-380

^{1}H NMR (400 MHz, DMSO) δ 8.72 – 8.68 (1H), 8.28 (2H), 8.25 (1H), 8.09 (2H), 7.97 (1H), 7.93 – 7.84 (3H), 7.72 (1H), 7.59 (3H), 7.36 – 7.27 (1H), 4.00 (3H). ^{13}C NMR (101 MHz, DMSO) δ 161.52, 160.51, 142.18, 141.72, 139.13, 134.32, 132.46, 131.34, 129.16, 127.44, 127.16, 124.38, 122.56, 122.47, 122.25, 122.22, 120.92, 120.20, 116.76, 110.28, 110.12, 107.98, 29.76. Chemical Formula: $C_{32}H_{18}Br_2N_4$, Elemental Analysis: calcd., C, 62.16; H, 2.93; N, 9.06; found, C, 62.51; H, 3.06; N, 8.85. HRMS (m/z): $[M+H]^+$ calcd., 616.9971; found, 616.9958.

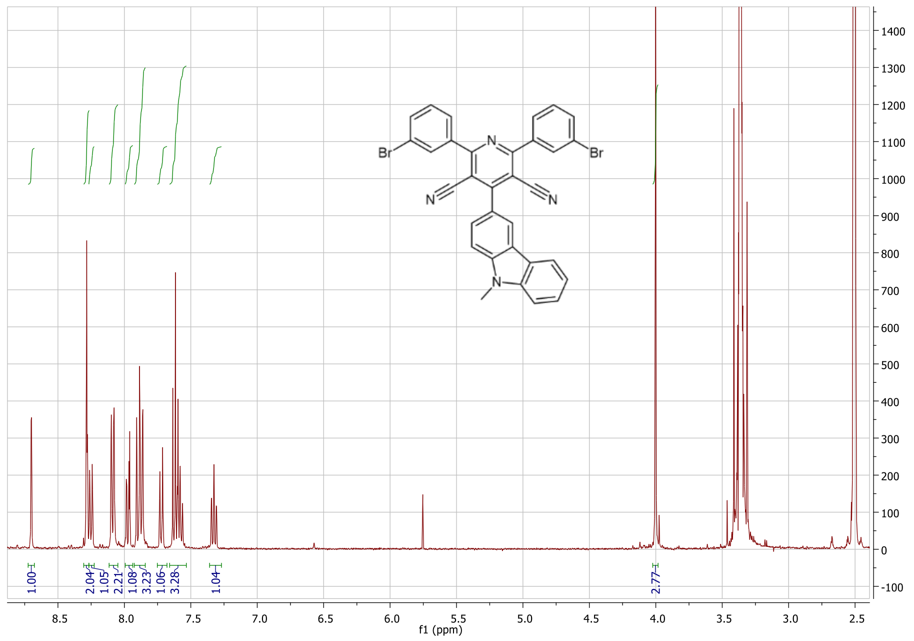

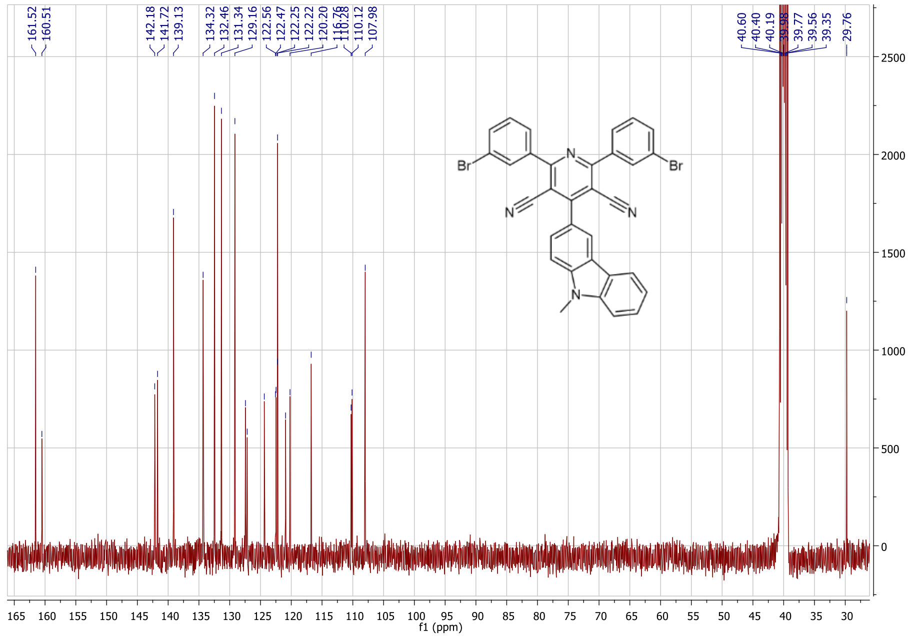

CNP-415

^{1}H NMR (400 MHz, DMSO) δ 8.02 – 7.96 (2H), 7.96 – 7.89 (6H), 7.78 – 7.66 (4H), 7.57 – 7.48 (2H), 7.35 (2H), 2.39 (3H). ^{13}C NMR (101 MHz, DMSO) δ 163.59, 161.61, 161.59, 161.16, 159.38, 142.87, 138.99, 138.91, 138.28, 136.34, 132.77, 131.45, 131.37, 130.50, 130.22, 127.28, 127.12, 126.29, 126.27, 118.73, 118.52, 117.01, 116.78, 116.37, 107.56, 21.21. Chemical Formula: $C_{32}H_{19}F_2N_3$, Elemental Analysis: calcd., C, 79.49; H, 3.96; N, 8.69; found C, 78.20; H, 3.84; N, 8.64. HRMS (m/z): $[M+H]^+$ calcd., 484.1620; found, 484.1614.

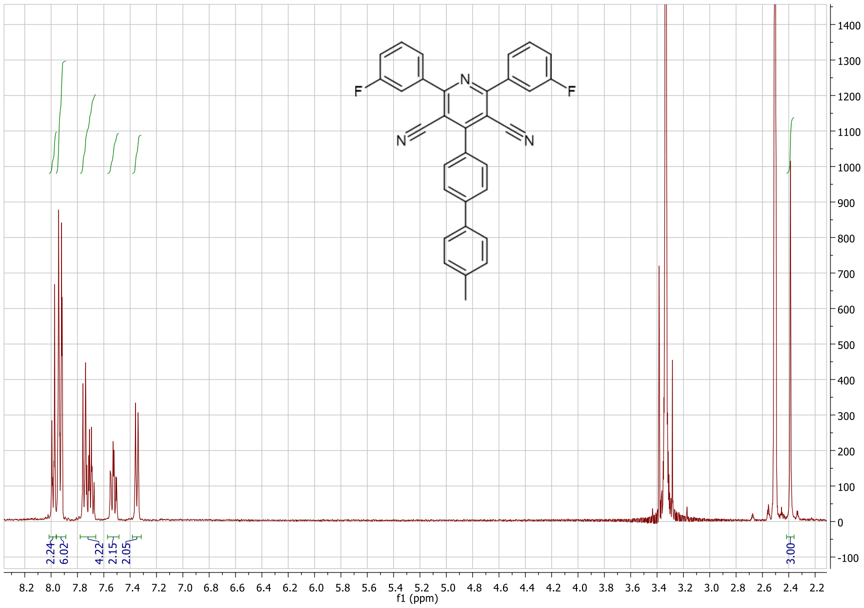

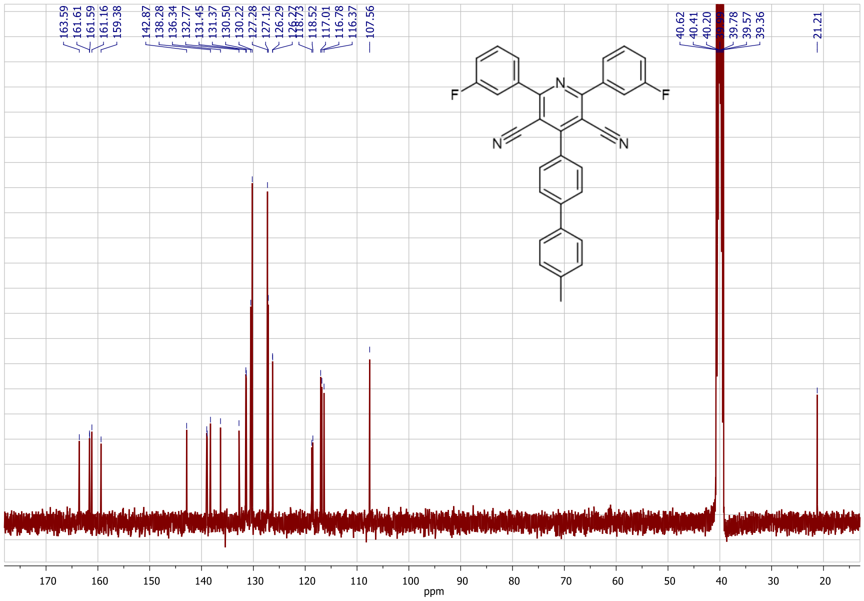

CNP-439

^{1}H NMR (400 MHz, DMSO) δ 8.30 (2H), 8.21 – 8.14 (4H), 8.09 – 8.03 (2H), 8.03 – 7.97 (2H), 7.78 – 7.73 (2H), 7.69 (2H), 7.54 – 7.48 (4H), 7.39 – 7.32 (2H). ^{13}C NMR (101 MHz, DMSO) δ 161.59, 158.83, 140.16, 139.72, 138.74, 133.89, 132.83, 131.89, 131.57, 131.22, 129.70, 128.76, 127.16, 127.01, 123.57, 121.18, 121.08, 116.34, 110.10, 107.78. Chemical Formula: $C_{37}H_{20}Cl_2N_4$, Elemental Analysis: calcd., C, 75.13; H, 3.41; N, 9.47; found C, 74.78; H, 3.36; N, 9.33. HRMS (m/z): $[M+H]^+$ calcd., 591.1138; found, 591.1133.

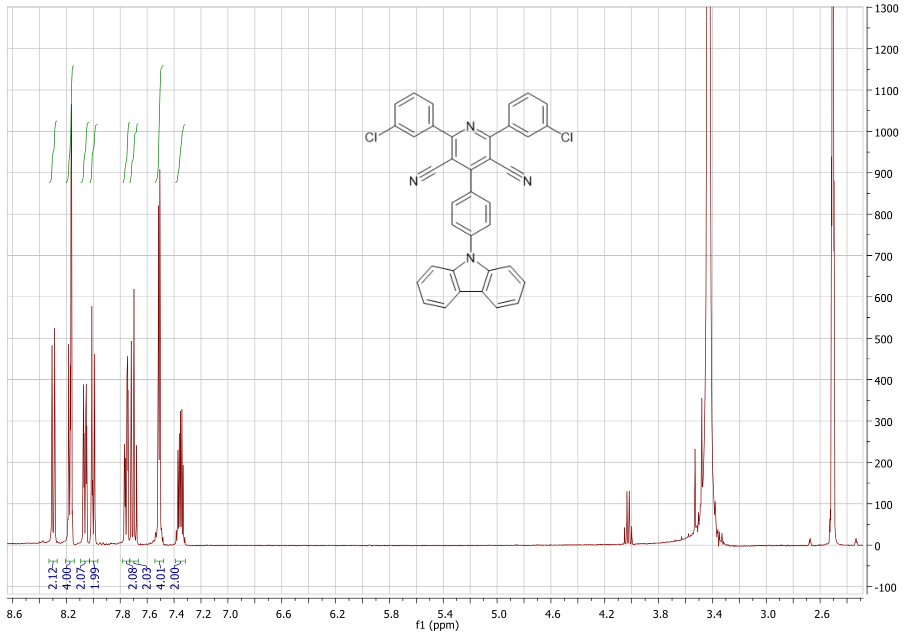

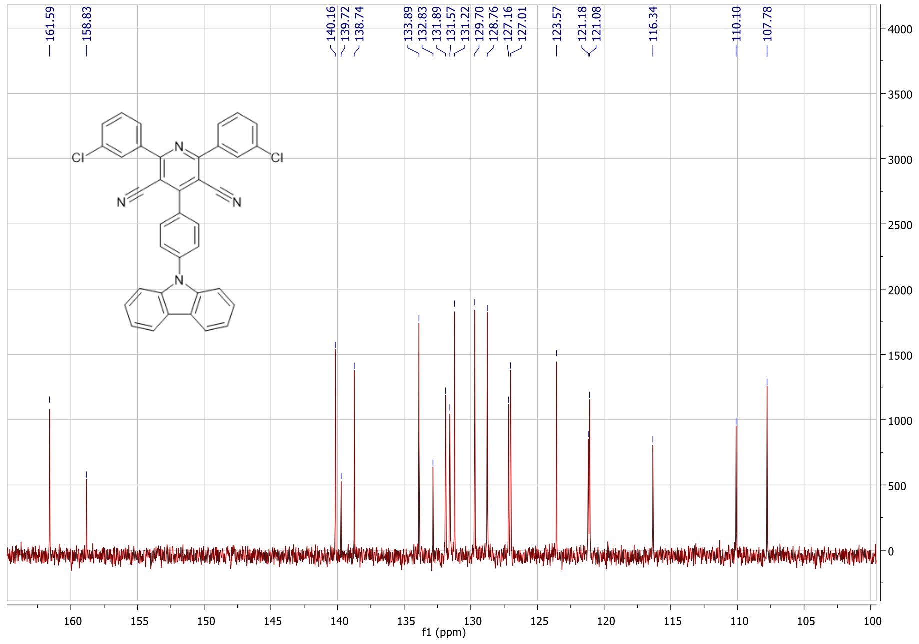

CNP-446

^{1}H NMR (400 MHz, DMSO) δ 8.15 (2H), 8.03 (2H), 8.02 – 7.94 (4H), 7.93 – 7.86 (2H), 7.75 (2H), 7.72 – 7.64 (2H), 7.43 – 7.30 (2H). ^{13}C NMR (101 MHz, DMSO) δ 164.07, 161.63, 161.57, 159.18, 141.92, 138.74, 135.74, 135.71, 133.86, 133.06, 131.53, 131.17, 130.57, 129.67, 129.64, 129.55, 128.76, 127.40, 116.53, 116.32, 107.65. Chemical Formula: $C_{31}H_{16}Cl_2FN_3$, Elemental Analysis: calcd., C, 71.55; H, 3.10; N, 8.07; found C, 71.49; H, 3.06; N, 8.13. HRMS (m/z): $[M+H]^+$ calcd., 520.0778; found, 520.0770.

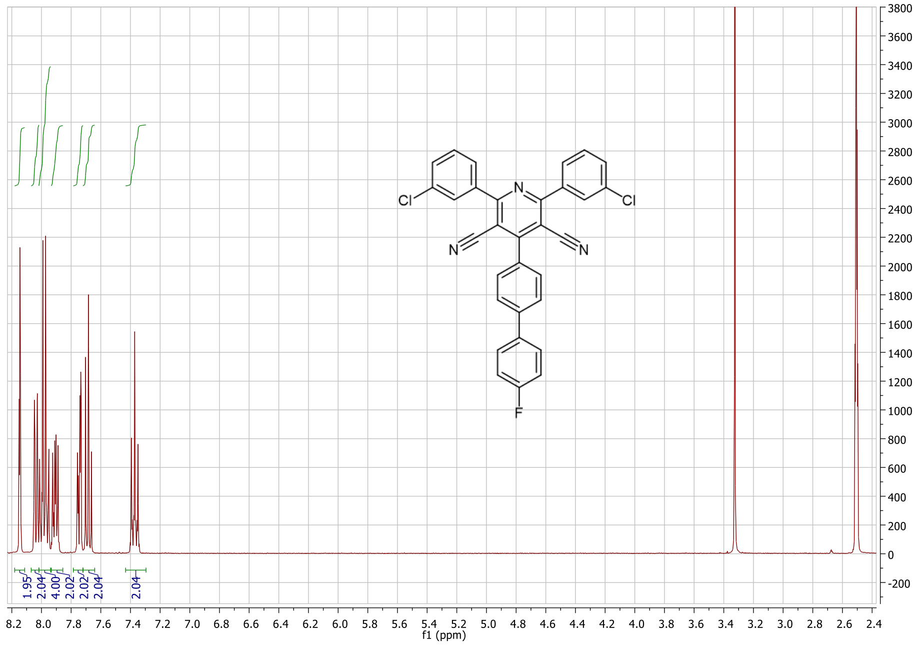

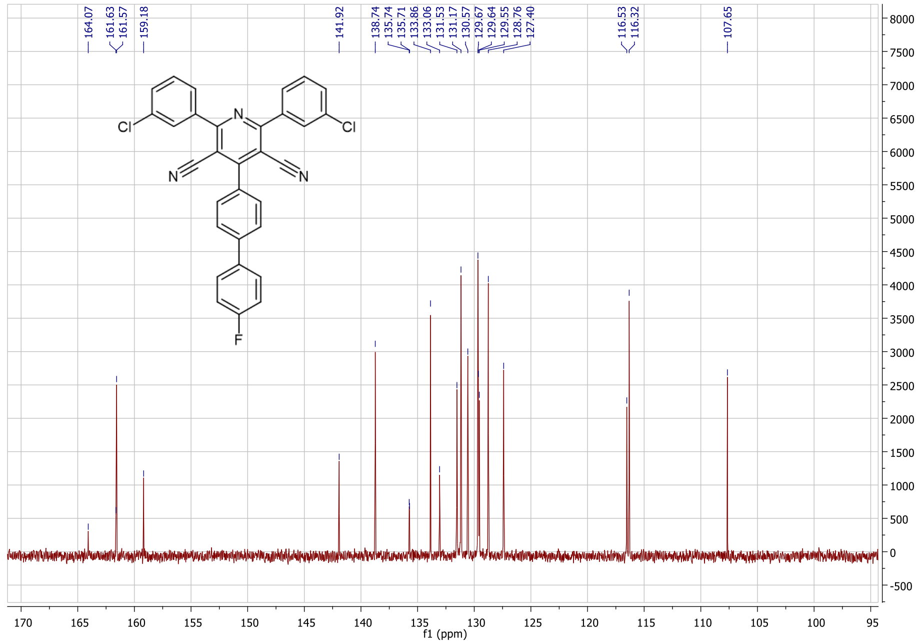

CNP-459

^{1}H NMR (400 MHz, DMSO) δ 7.96 – 7.81 (4H), 7.66 – 7.58 (6H), 7.56 (2H), 7.51 – 7.43 (3H), 7.23 (2H), 3.86 (6H). ^{13}C NMR (101 MHz, DMSO) δ 162.60, 159.66, 159.06, 138.12, 134.49, 132.14, 132.03, 130.45, 130.26, 129.72, 129.34, 125.25, 122.32, 122.24, 117.30, 116.41, 115.37, 106.90, 91.91, 88.95, 55.91. Chemical Formula: $C_{35}H_{23}N_3O_2$, Elemental Analysis: calcd., 81.22; H, 4.48; N, 8.12; found, C, 81.07; H, 4.44; N, 8.12. HRMS (m/z): $[M+H]^+$ calcd., 518.1863; found, 518.1869.

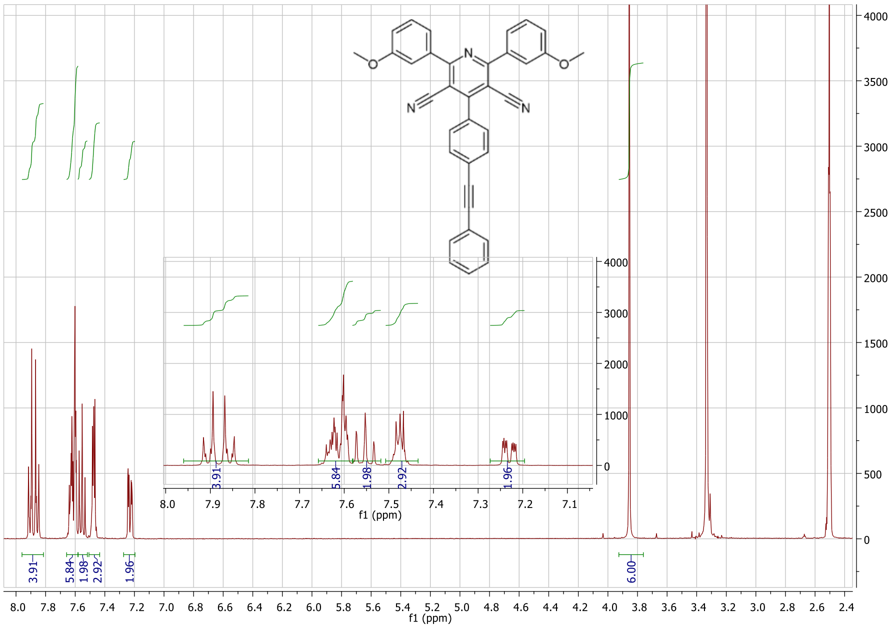

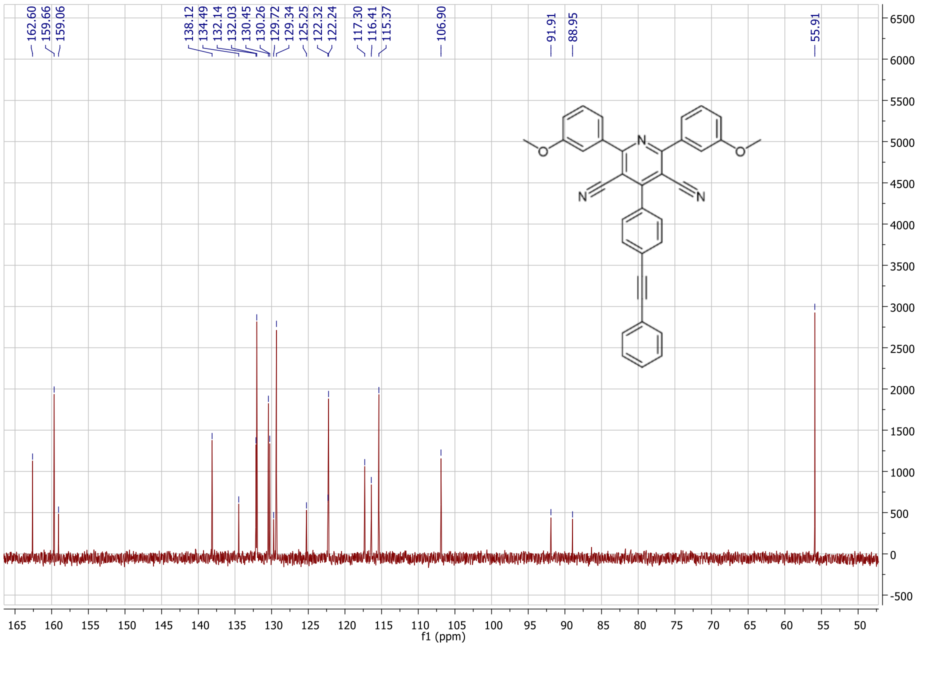

CNP-463

^{1}H NMR (400 MHz, DMSO) δ 8.68 (1H), 8.25 (1H), 7.96 – 7.85 (2H), 7.73 (1H), 7.63 (4H), 7.60 – 7.50 (m, 3H), 7.31 (1H), 7.23 (2H), 4.64 – 4.45 (2H), 3.86 (6H), 1.40 (3H). ^{13}C NMR (101 MHz, DMSO) δ 162.75, 160.71, 159.65, 141.04, 140.58, 138.36, 130.37, 127.42, 127.11, 124.64, 122.63, 122.57, 122.44, 122.29, 121.07, 120.12, 117.11, 117.00, 115.50, 110.19, 109.90, 107.35, 55.92, 37.75, 14.33. Chemical Formula: $C_{35}H_{26}N_4O_2$, Elemental Analysis: calcd., C, 78.63; H, 4.90; N, 10.48; found C, 78.35; H, 4.84; N, 10.45. HRMS (m/z): $[M+H]^+$ calcd., 535.2129; found, 535.2127.

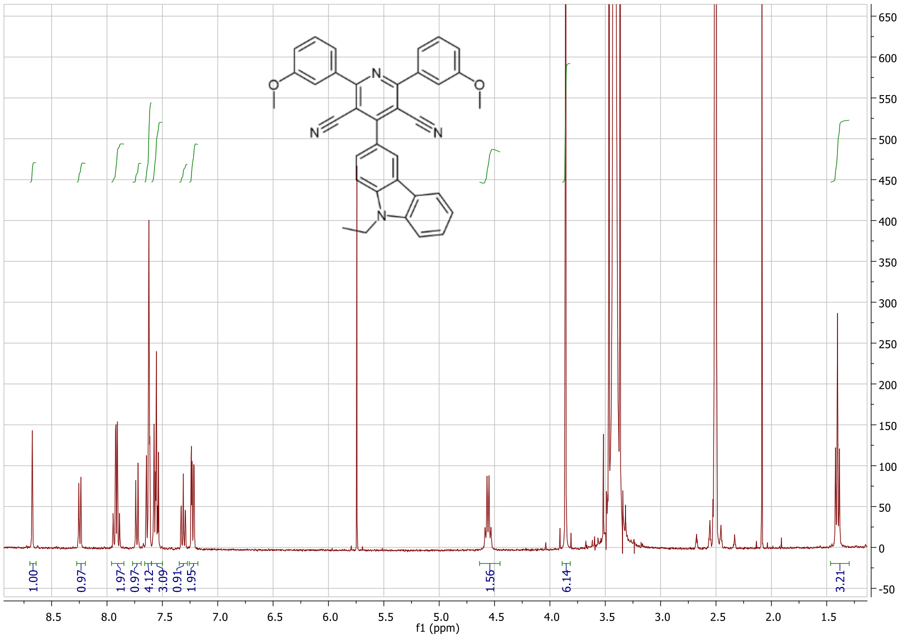

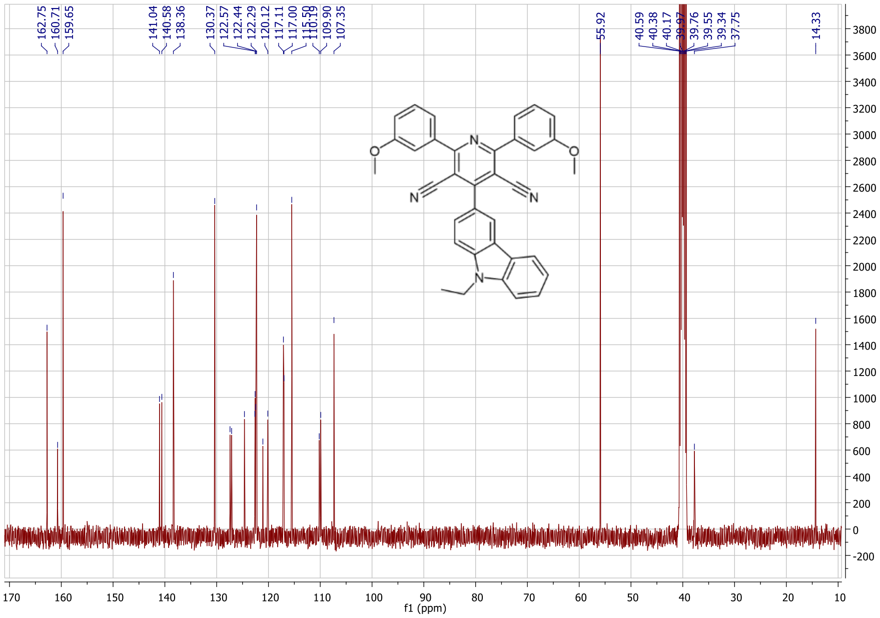

CNP-464

^{1}H NMR (400 MHz, DMSO) δ 8.67 (1H), 8.24 (1H), 7.95 (1H), 7.88 (1H), 7.71 (1H), 7.68 – 7.61 (4H), 7.61 – 7.50 (3H), 7.36 – 7.29 (1H), 7.23 (2H), 3.99 (3H), 3.86 (6H). ^{13}C NMR (101 MHz, DMSO) δ 162.71, 160.77, 159.65, 142.07, 141.69, 138.35, 130.36, 127.39, 127.09, 124.67, 122.46, 122.42, 122.29, 120.93, 120.12, 117.11, 116.96, 115.49, 110.23, 110.04, 107.38, 55.92, 29.74. Chemical Formula: $C_{34}H_{24}N_4O_2$, Elemental Analysis: calcd., C, 78.44; H, 4.65; N, 10.76; found, C, 77.63; H, 4.57; N, 10.75. HRMS (m/z): $[M+H]^+$ calcd., 521.1972; found, 521.1976.

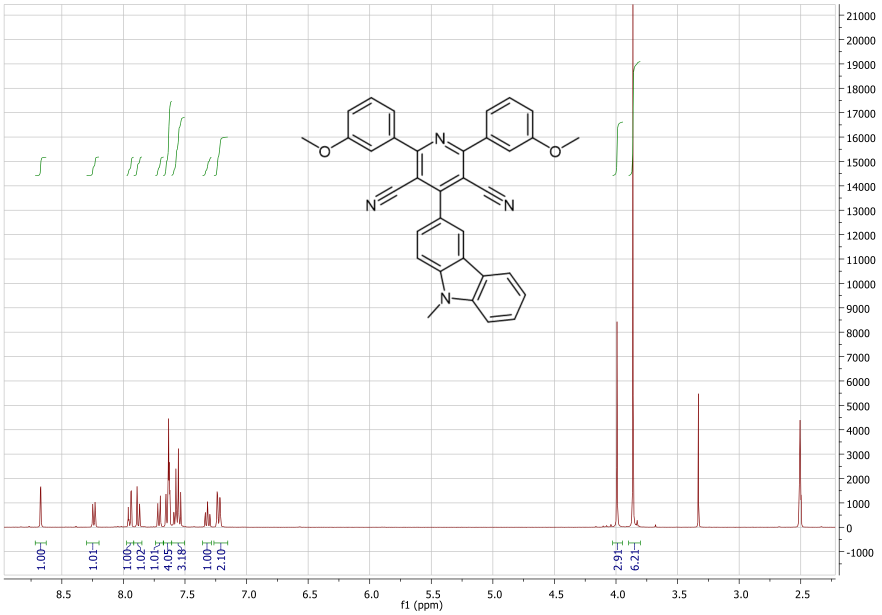

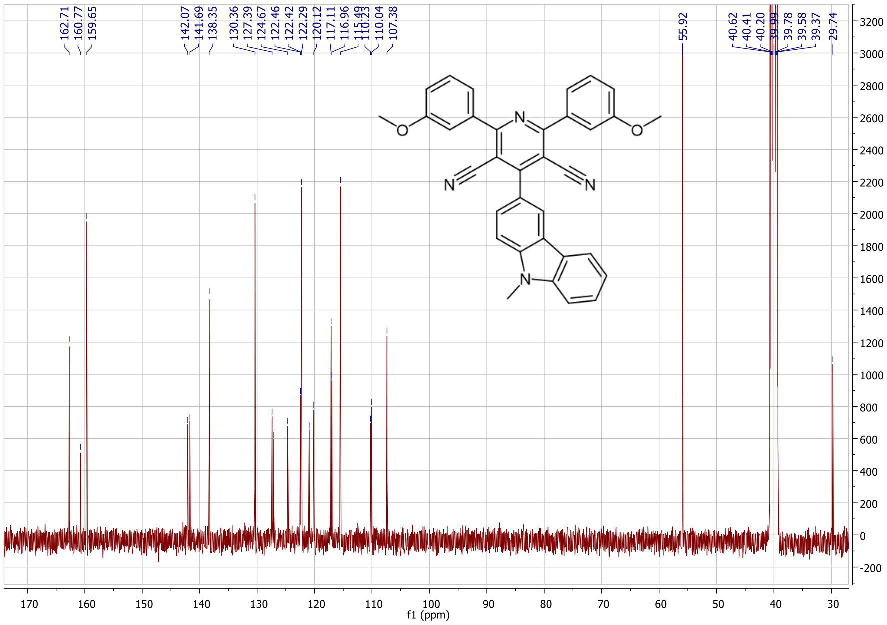

CNP-489

^{1}H NMR (400 MHz, DMSO) δ 8.09 – 8.02 (6H), 8.01 – 7.96 (2H), 7.90 – 7.82 (5H), 7.82 – 7.76 (2H), 7.68 – 7.62 (1H), 7.62 – 7.52 (3H). 13C NMR (101 MHz, DMSO) δ 162.26, 159.48, 138.68, 138.12, 136.29, 133.98, 133.30, 131.84, 130.62, 127.75, 127.17, 126.19, 116.49, 106.98, 99.72. Chemical Formula: $C_{35}H_{19}I_2N_3$, Elemental Analysis: calcd., C, 57.17; H, 2.60; N, 5.71; found, C, 56.94; H, 2.56; N, 5.72. HRMS (m/z): $[M+H]^{+}$ calcd., 735.9741; found, 735.9736.

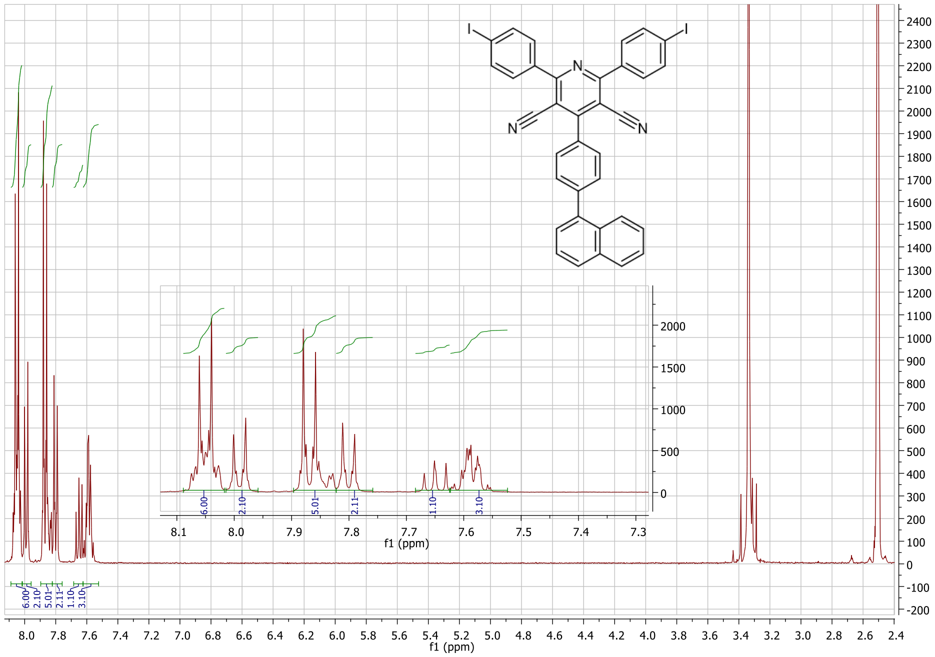

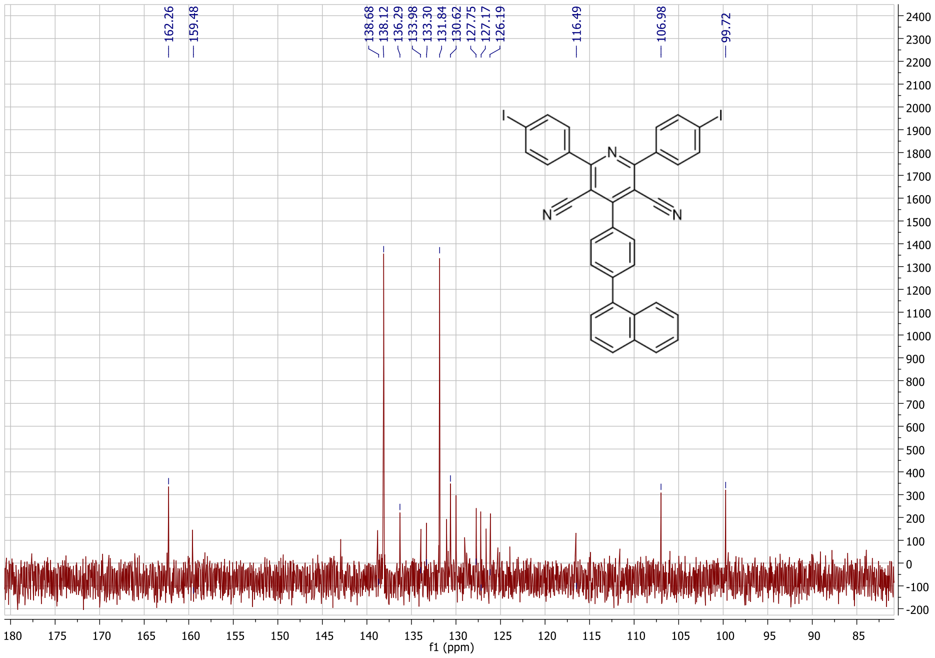

CNP-491

^{1}H NMR (400 MHz, DMSO) δ 8.72 – 8.62 (1H), 8.23 (1H), 8.09 – 7.99 (4H), 7.96 – 7.82 (6H), 7.74 (1H), 7.60 – 7.44 (1H), 7.35 – 7.25 (1H), 4.56 (2H), 1.40 (3H). ^{13}C NMR (101 MHz, DMSO) δ 162.27, 160.76, 141.09, 140.60, 138.05, 136.43, 131.86, 127.39, 127.14, 124.49, 122.66, 122.59, 122.43, 121.03, 120.17, 116.97, 110.23, 109.96, 107.21, 99.58, 37.76, 14.33. Chemical Formula: $C_{33}H_{20}I_2N_4$, Elemental Analysis: calcd., C, 54.57; H, 2.78; N, 7.71; found, C, 54.14; H, 2.69; N, 7.61. HRMS (m/z): $[M+H]^+$ calcd., 726.9850; found, 726.9845.

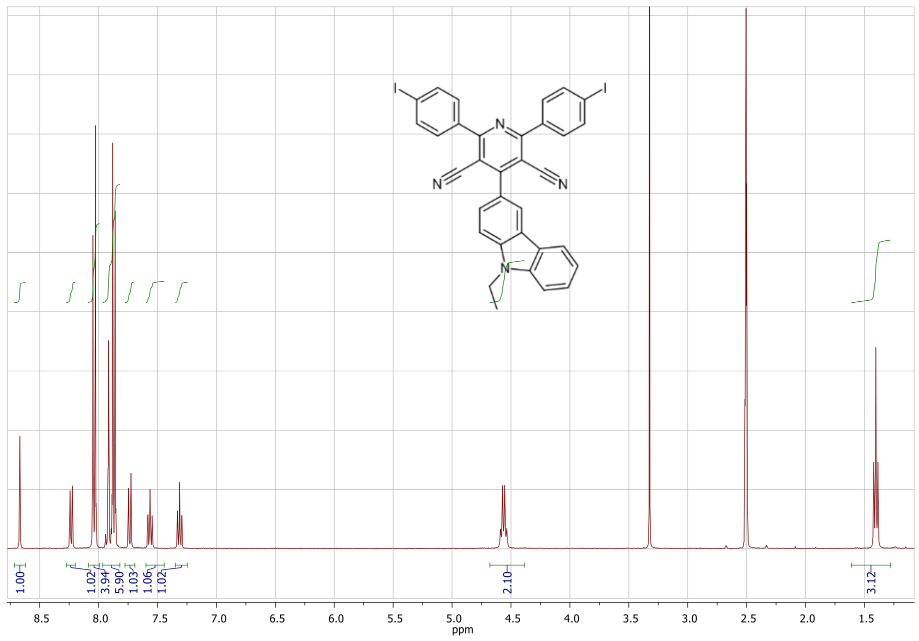

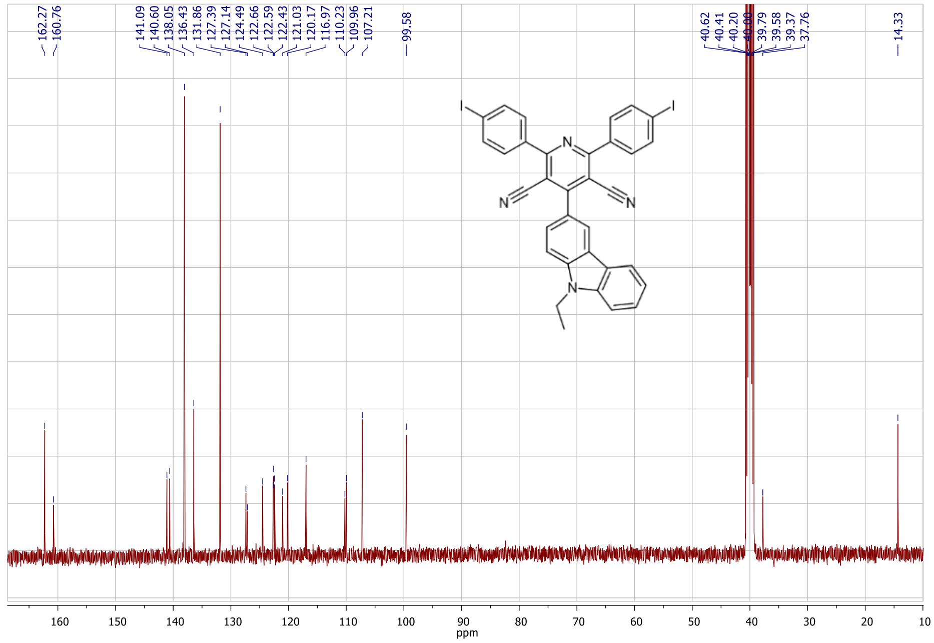

CNP-494

^{1}H NMR (400 MHz, $CDCl_3$) δ 7.87 – 7.79 (4H), 7.71 – 7.63 (4H), 7.39 – 7.30 (2H), 7.15 – 7.09 (4H), 6.95 – 6.87 (2H), 6.86 – 6.79 (4H), 3.75 (6H). ^{13}C NMR (101 MHz, $CDCl_3$) δ 161.71, 156.08, 150.41, 138.11, 136.98, 134.90, 130.02, 129.41, 127.07, 121.57, 116.20, 115.37, 114.02, 104.45, 97.59, 54.49. Chemical Formula: $C_{39}H_{26}I_2N_4O_2$, Elemental Analysis: calcd., C, 56.00; H, 3.13; N, 6.70; found C, 55.59; H, 3.10; N, 6.42. HRMS (m/z): $[M+H]^+$ calcd., 837.0218; found, 837.0205.

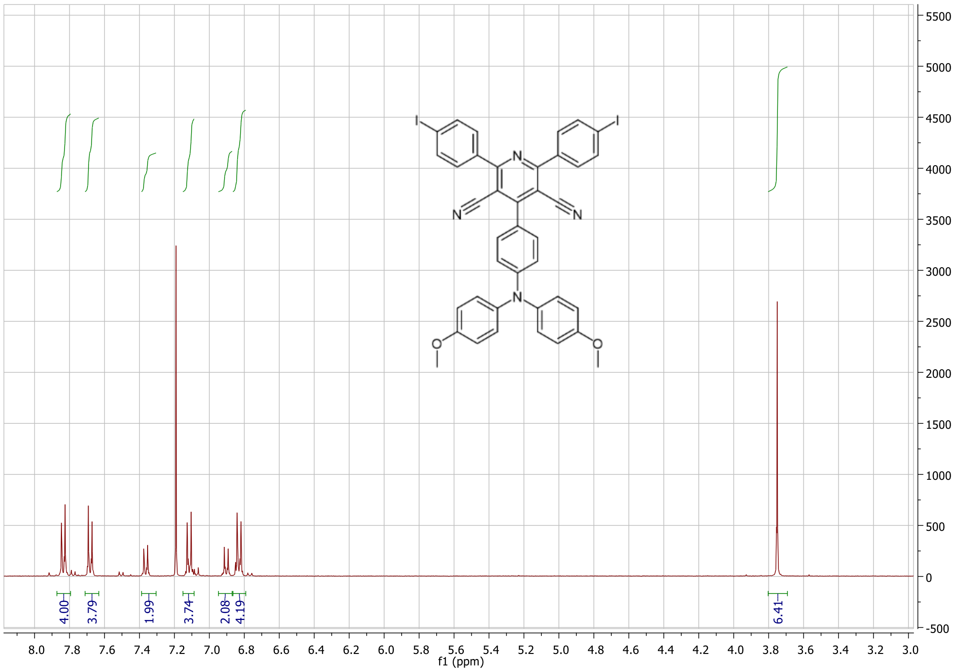

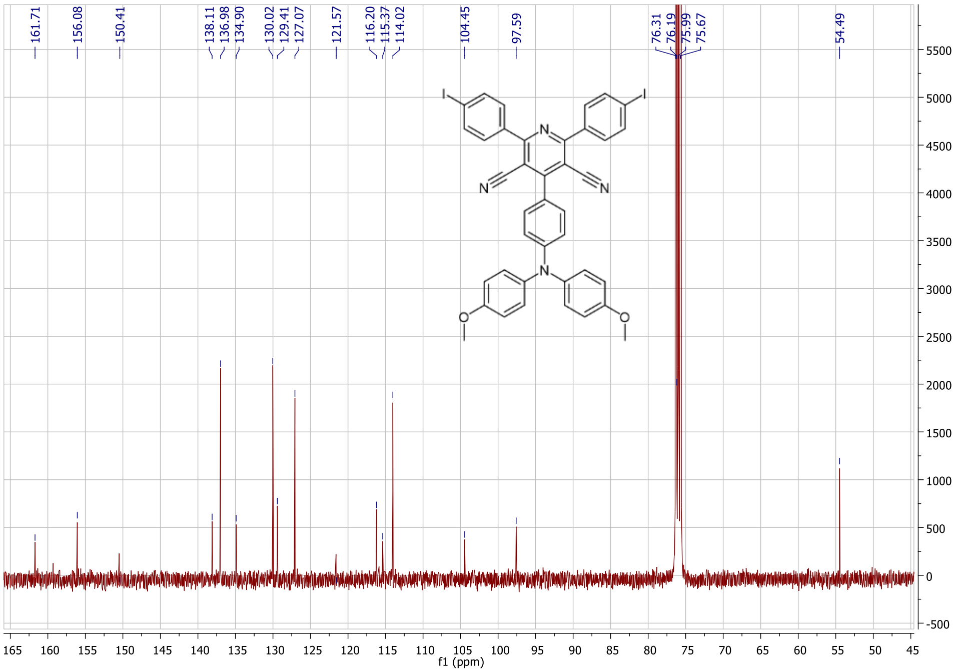

CNP-502

^{1}H NMR (400 MHz, DMSO) δ 8.06 – 8.01 (4H), 8.00 – 7.95 (2H), 7.94 – 7.86 (4H), 7.86 – 7.81 (4H), 7.41 – 7.32 (2H). ^{13}C NMR (101 MHz, DMSO) δ 162.22, 161.61, 159.47, 141.80, 138.10, 136.26, 135.76, 135.73, 133.20, 131.80, 130.49, 129.61, 129.53, 127.37, 116.53, 116.50, 116.32, 106.88, 99.71. Chemical Formula: $C_{31}H_{16}FI_2N_3$, Elemental Analysis: calcd., C, 52.94; H, 2.29; N, 5.97; found, C, 52.84; H, 2.29; N, 5.91. HRMS (m/z): [M+H]$^+$ calcd., 703.9490; found, 703.9488.

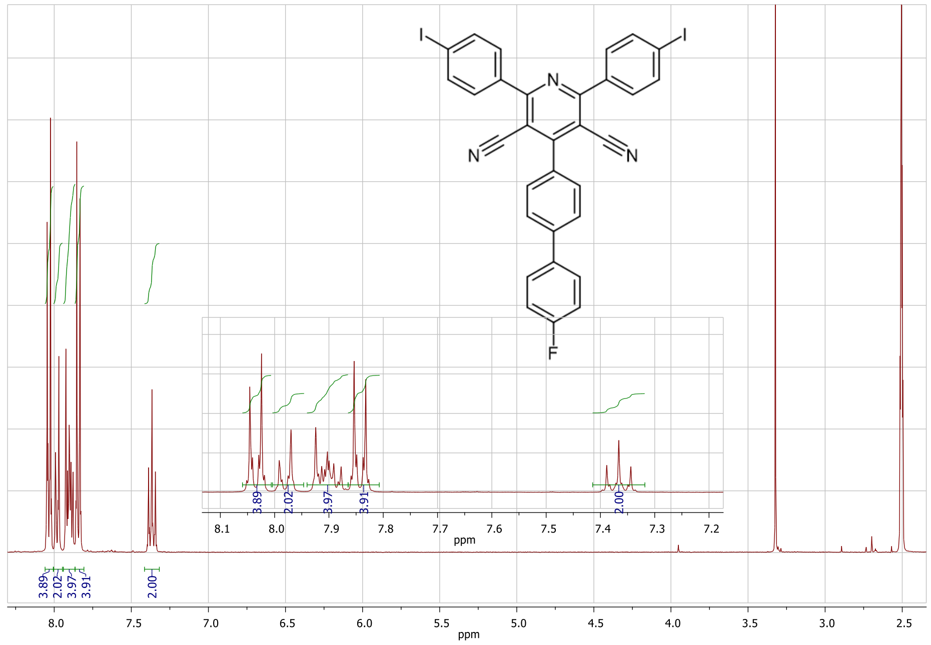

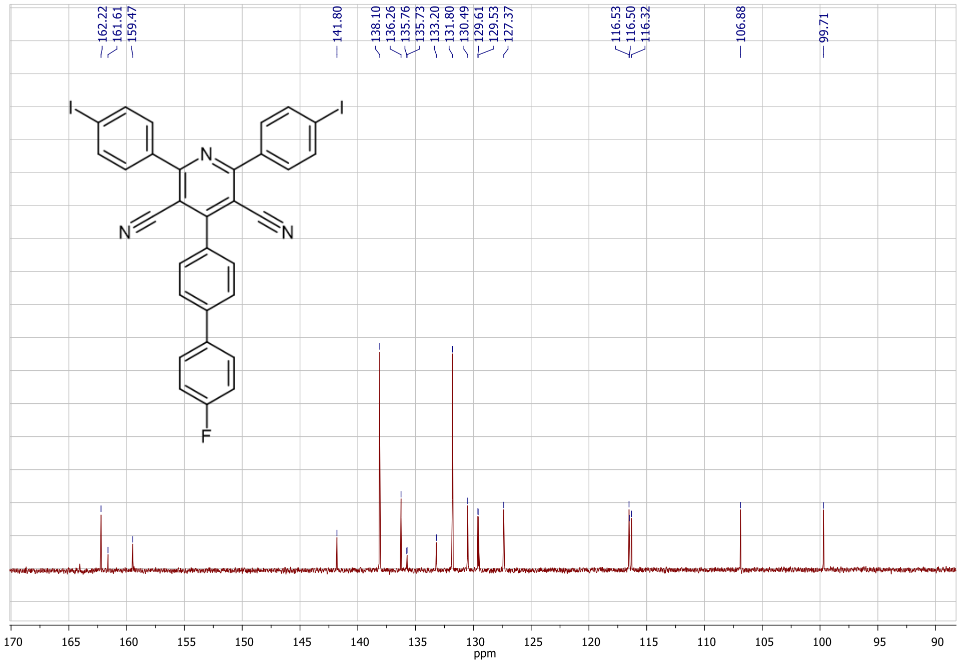

CNP-504

^{1}H NMR (400 MHz, DMSO) δ 8.38 (2H), 8.23 (1H), 8.13 (1H), 8.04 (4H), 7.85 – 7.73 (3H), 7.65 (2H), 7.45 (2H). ^{13}C NMR (101 MHz, DMSO) δ 161.37, 159.09, 140.12, 138.80, 138.12, 133.47, 131.81, 131.40, 131.24, 130.31, 129.32, 129.12, 128.19, 128.16, 127.35, 125.92, 125.26, 115.87, 108.74, 95.25. Chemical Formula: $C_{29}H_{15}I_2N_3$, Elemental Analysis: calcd., C, 52.83; H, 2.29; N, 6.37; found, C, 52.71; H, 2.26; N, 6.29. HRMS (m/z): $[M+H]^+$ calcd., 659.9428; found, 659.9415.

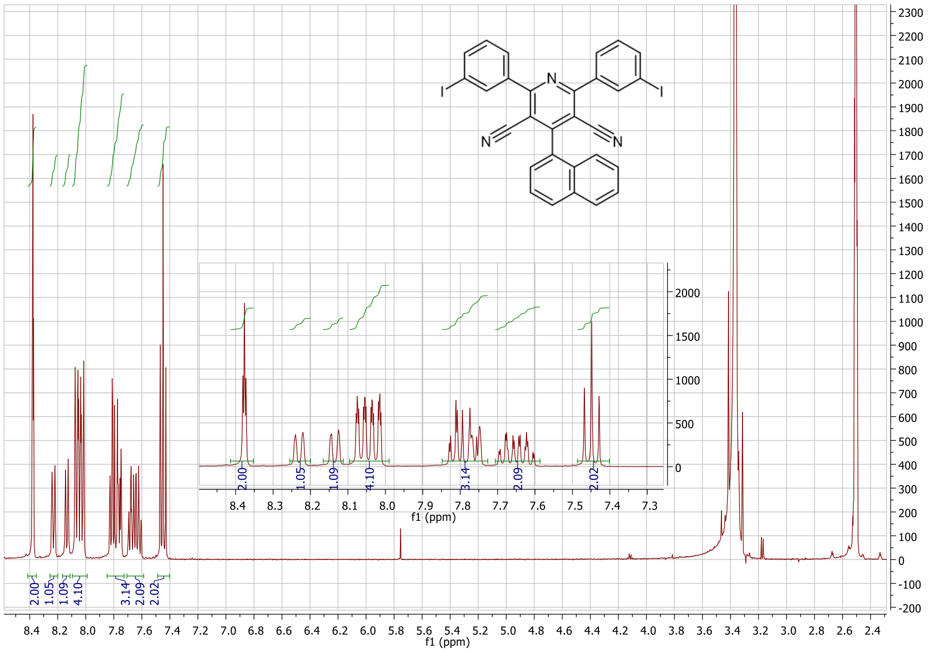

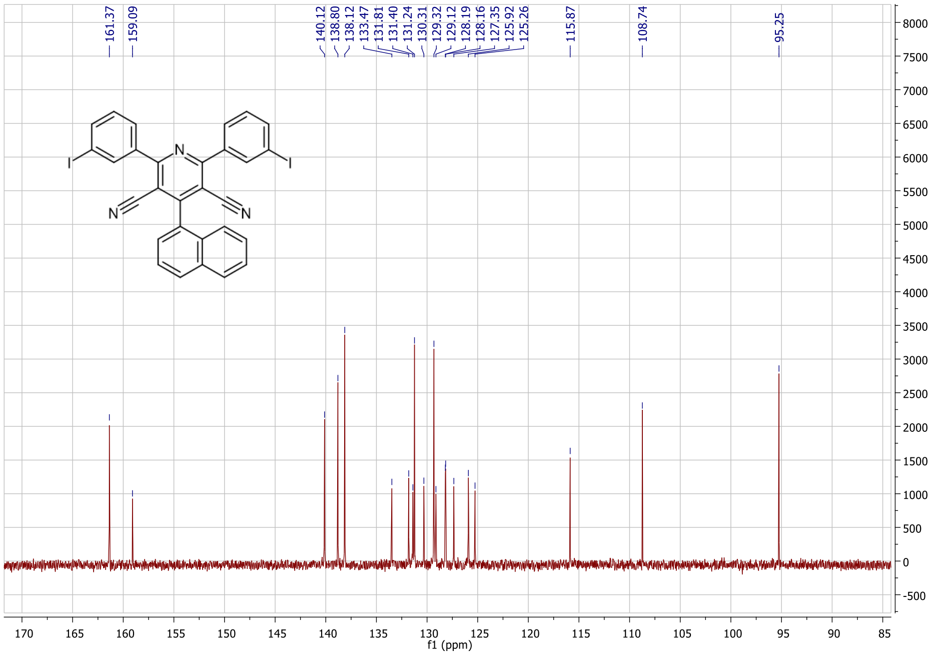

CNP-519

^{1}H NMR (400 MHz, DMSO) δ 8.70 (1H), 8.41 (2H), 8.26 (1H), 8.09 (2H), 8.03 (2H), 7.94 (2H), 7.74 (1H), 7.57 (1H), 7.45 (2H), 7.32 (1H), 4.57 (2H), 1.41 (3H). ^{13}C NMR (101 MHz, DMSO) δ 161.55, 160.41, 141.13, 140.61, 140.08, 139.05, 138.10, 131.19, 129.44, 127.48, 124.41, 122.70, 122.61, 122.44, 121.08, 120.18, 116.83, 109.97, 107.81, 95.24, 37.65, 14.34. Chemical Formula: $C_{33}H_{20}I_2N_4$, Elemental Analysis: calcd., C, 54.57; H, 2.78; N, 7.71; found, C, 54.30; H, 2.68; N, 7.65. HRMS (m/z): $[M+H]^+$ calcd., 726.9850; found, 726.9846.

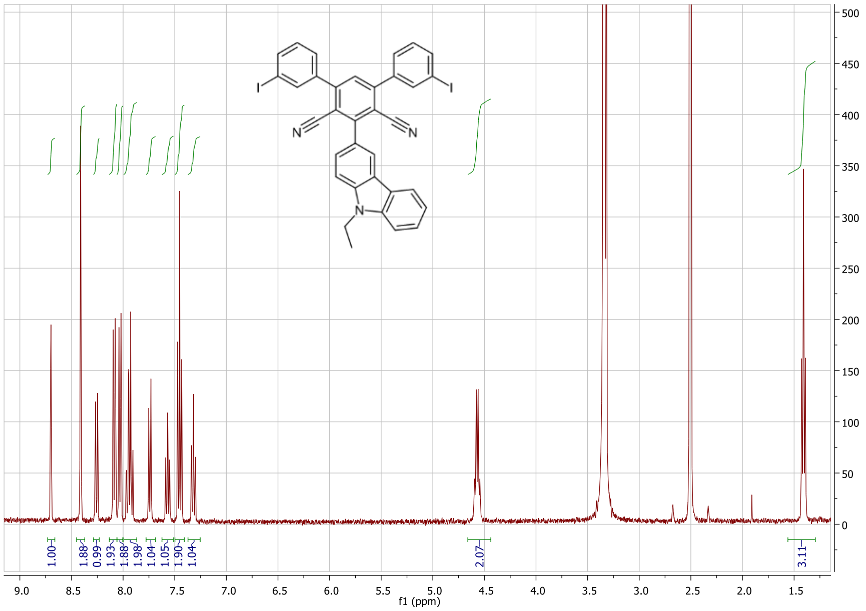

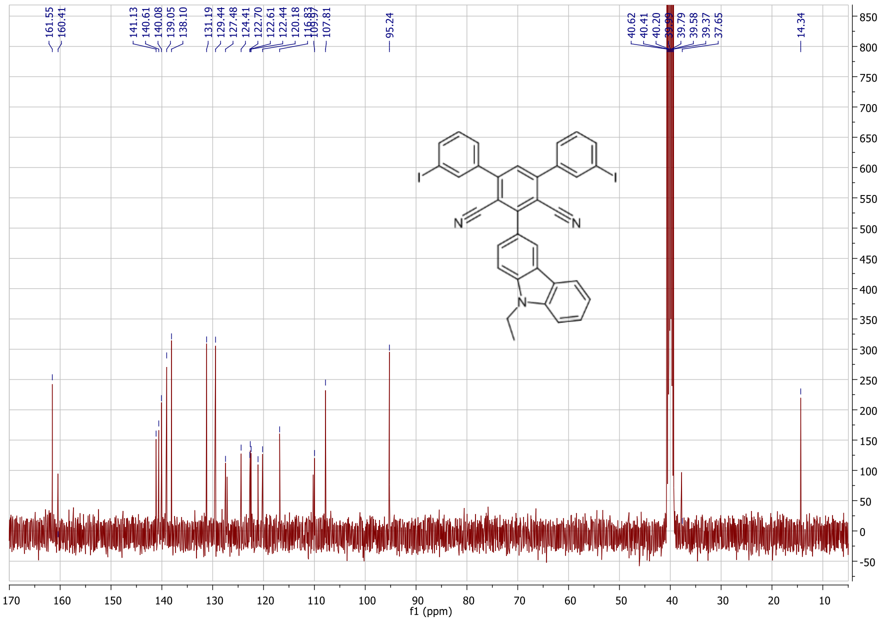

CNP-559

^{1}H NMR (400 MHz, DMSO) δ 8.45 (2H), 8.38 (2H), 8.15 – 8.07 (3H), 8.05 (5H), 8.03 – 7.99 (2H), 7.91 (2H). ^{13}C NMR (101 MHz, DMSO) δ 161.65, 158.86, 143.71, 141.07, 137.68, 134.28, 134.14, 133.47, 130.73, 130.59, 130.05, 129.73, 129.40, 128.43, 128.30, 128.00, 126.74, 126.70, 125.71, 123.01, 119.22, 116.25, 111.35, 108.02. Chemical Formula: $C_{34}H_{16}F_6N_4$, Elemental Analysis: calcd., C, 68.69; H, 2.71; N, 9.42; found C, 68.66; H, 2.60; N, 9.35. HRMS (m/z): [M+H]$^{+}$ calcd., 595.1352; found, 595.1362.

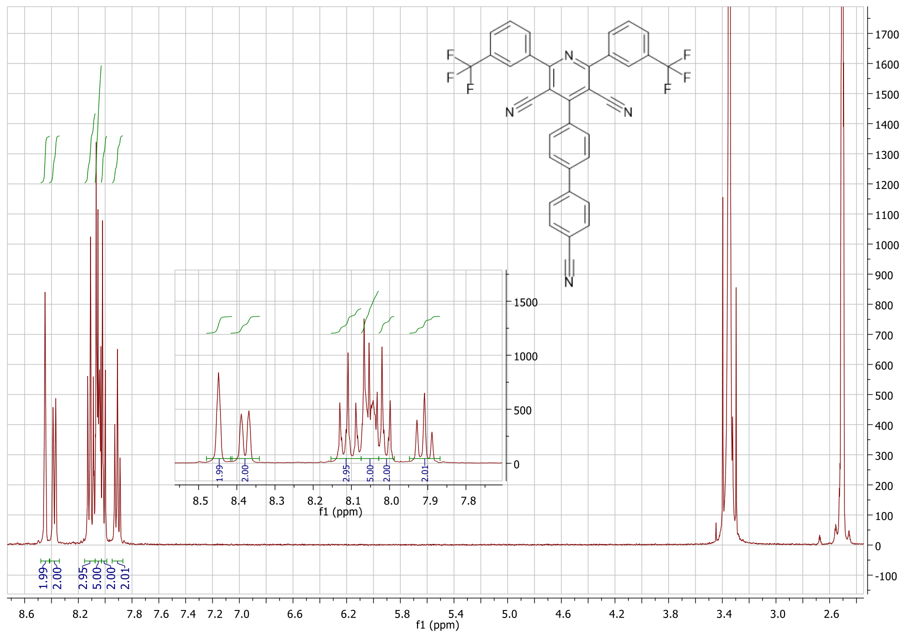

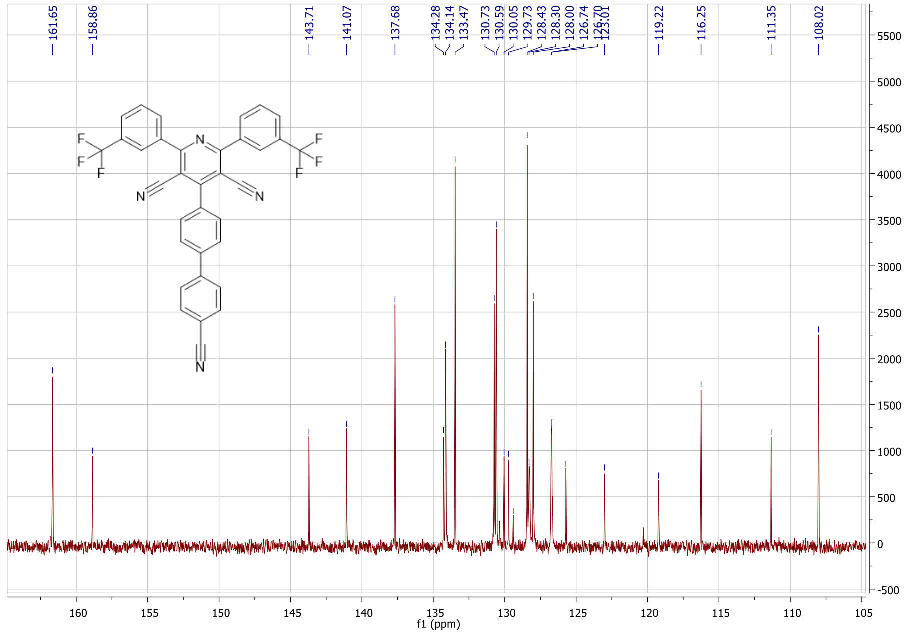

CNP-624

^{1}H NMR (400 MHz, DMSO) δ 8.71 (1H), 8.35 (1H), 7.96 – 7.89 (4H), 7.80 (1H), 7.78 – 7.69 (4H), 7.65 – 7.58 (1H), 7.58 – 7.49 (2H), 7.47 – 7.36 (2H), 6.76 – 6.66 (4H), 5.98 (4H). ^{13}C NMR (101 MHz, DMSO) δ 161.98, 161.19, 152.64, 141.42, 141.27, 136.78, 131.65, 130.84, 128.68, 127.93, 127.57, 127.38, 127.02, 123.71, 123.17, 122.92, 122.72, 121.24, 118.29, 113.54, 110.53, 110.17, 101.77. Chemical Formula: $C_{37}H_{24}N_6$, Elemental Analysis: calcd., C, 80.42; H, 4.38; N, 15.21; found, C, 79.09; H, 4.31; N, 14.86. HRMS (m/z): $[M+H]^+$ calcd., 553.2135; found, 553.2134.

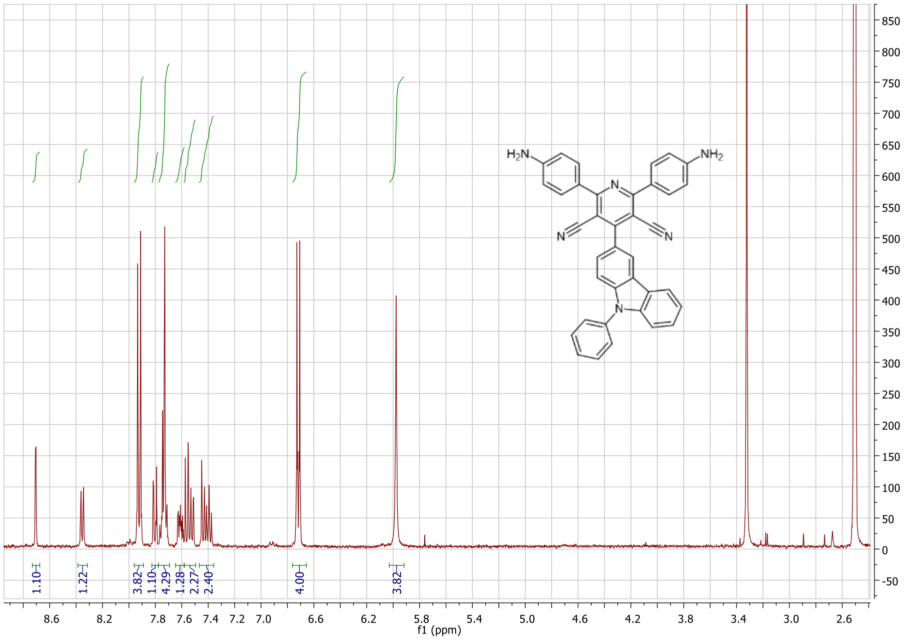

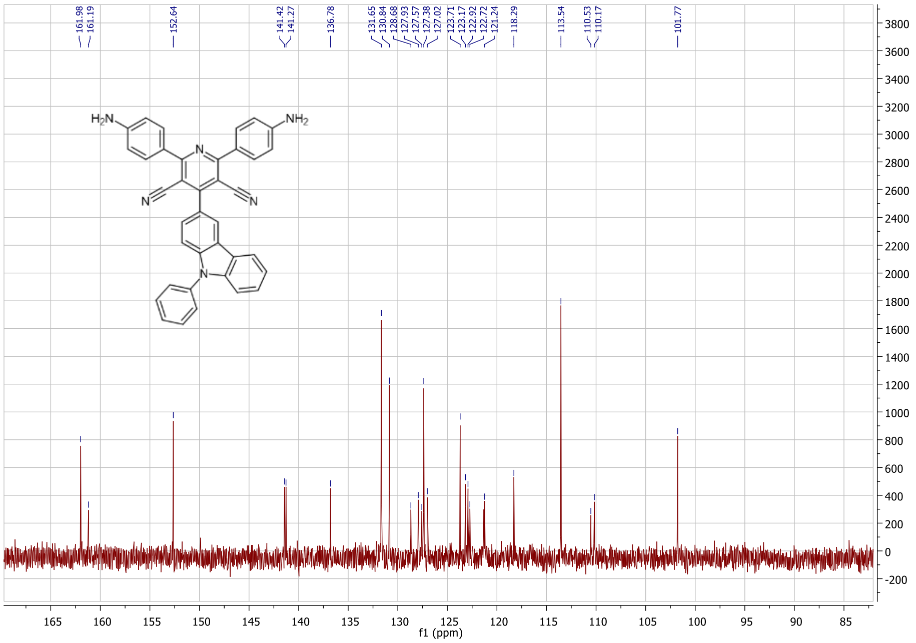

References

1. Qin, Y., Sun, R., Gianoulis, N.P. & Nocera, D.G. Photoredox Nickel-Catalyzed C–S Cross-Coupling: Mechanism, Kinetics, and Generalization. *Journal of the American Chemical Society* **143**, 2005-2015 (2021).
2. Till, N.A., Tian, L., Dong, Z., Scholes, G.D. & MacMillan, D.W.C. Mechanistic Analysis of Metallaphotoredox C–N Coupling: Photocatalysis Initiates and Perpetuates Ni(I)/Ni(III) Coupling Activity. *Journal of the American Chemical Society* **142**, 15830-15841 (2020).
